# Investigations into Simplified Analogues of the Herbicidal Natural Product (+)‐Cornexistin

**DOI:** 10.1002/chem.202300199

**Published:** 2023-05-23

**Authors:** Christian Steinborn, Aldo Tancredi, Christoph Habiger, Christina Diederich, Jan Kramer, Anna M. Reingruber, Bernd Laber, Jörg Freigang, Gudrun Lange, Dirk Schmutzler, Anu Machettira, Gilbert Besong, Thomas Magauer, David M. Barber

**Affiliations:** ^1^ Institute of Organic Chemistry and Center for Molecular Biosciences University of Innsbruck Innrain 80–82 6020 Innsbruck Austria; ^2^ Research & Development, Weed Control Bayer AG, Crop Science Division Industriepark Höchst 65926 Frankfurt am Main Germany; ^3^ Research & Development, Hit Discovery Bayer AG, Crop Science Division Alfred-Nobel-Straße 50 40789 Monheim am Rhein Germany

**Keywords:** crop protection, herbicides, natural products, structure simplification, transketolase

## Abstract

We report the design, synthesis and biological evaluation of simplified analogues of the herbicidal natural product (+)‐cornexistin. Guided by an X‐Ray co‐crystal structure of cornexistin bound to transketolase from *Zea mays*, we attempted to identify the key interactions that are necessary for cornexistin to maintain its herbicidal profile. This resulted in the preparation of three novel analogues investigating the importance of substituents that are located on the nine‐membered ring of cornexistin. One analogue maintained a good level of biological activity and could provide researchers insights in how to further optimize the structure of cornexistin for commercialization in the future.

## Graphical abstract

**(+)‐Cornexistin has long been of interest** to the crop protection community due to its interesting herbicidal profile and unique mode of action. However, the complex chemical structure of cornexistin has hindered further research into new analogues. Guided by an X‐Ray co‐crystal structure, we investigated simplified analogues of cornexistin that focused on the effects that the functional groups located on the right‐hand side of the 9‐membered ring have on herbicidal efficacy and target affinity.

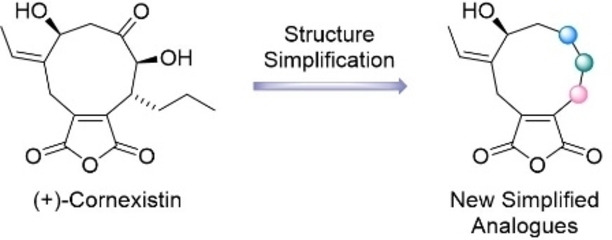

## Introduction

**Figure 1:**
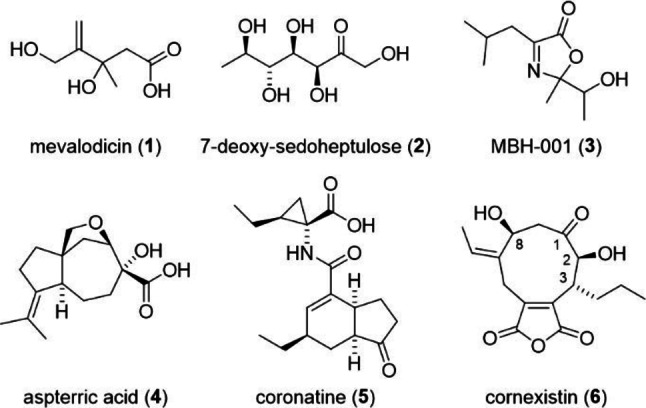
Chemical structures of the herbicidally active natural products mevalodicin (**1**), 7‐deoxy‐sedoheptulose (**2**), MBH‐001 (**3**), aspterric acid (**4**), coronatine (**5**) and cornexistin (**6**).

The search for novel biologically active natural products is very challenging and resource intensive. However, it can be extremely rewarding, with success in this endeavor often resulting in the discovery of unique chemical architectures that provide novel leads and new biological targets for both the pharmaceutical^[1]^ and agrochemical industries.^[2]^ Due to the lack of groundbreaking mode of action (MoA) innovation in the commercial herbicide market,^[3]^ research teams are more frequently turning to natural products for inspiration in the hunt for the next blockbuster herbicide that can fight resistant weeds.^[4]^ This has resulted in the disclosure of many herbicidal natural products, as well as the elucidation of several of the MoAs associated with them. Examples of herbicidal natural products that have been worked on recently include mevalocidin (**1**),^[5]^ 7‐deoxy‐sedoheptulose (**2**),^[6]^ MBH‐001 (**3**),^[7]^ aspterric acid (**4**)^[8]^ and coronatine (**5**)^[9]^ (Figure 1). One herbicidal natural product that has been of interest to the research community for some time, is the nonadride (+)‐cornexistin (**6**). First isolated in 1991 from the fungus identified as *Paecilomyces variotii* SANK 21086, cornexistin (**6**) was shown to display excellent post‐emergence efficacy against a range of weed species, whilst also exhibiting selectivity for corn.^[10]^ Because of its unique chemical structure and its promising weed control profile, cornexistin (**6**) has been the topic of several research programs over the years.^[11]^ This has included the MoA elucidation of cornexistin (**6**), i. e., its capacity at inhibiting transketolase (TK; EC.2.2.1.1)^[11f]^ which is an important enzyme in both the Calvin‐Benson‐Bassham cycle and the oxidative pentose phosphate pathway in chloroplasts.^[12]^ Additionally, many synthetic efforts were directed towards the preparation of cornexistin (**6**).^[13]^ The culmination of these efforts resulted in the first total synthesis of cornexistin (**6**) being reported in 2020.^[14]^

After first synthesizing or isolating the desired natural product, the lead structure often needs to be optimized for commercial use. This is a significant drawback of working with natural products, as typically the synthetic sequences employed are very long making the optimization of the compound a time consuming and arduous process. Therefore, it is often prudent to attempt to simplify the structure of the natural product,^[15]^ either by identifying the key functional groups required for good biological efficacy, or by implementing techniques such as bioisosteric replacement and scaffold hopping.^[16]^ Herein, we report our studies into the chemical simplification of cornexistin (**6**), which resulted in a set of novel synthetic analogues that were evaluated for their *in vitro* TK inhibition and their *in vivo* herbicidal efficacy.

## Results and Discussion

### *In vitro* target engagement

Since an X‐ray structure of *Zea mays* TK bound with thiamine pyrophosphate (TPP) cofactor (holoTK) is known from previous work (pdb accession code 1ITZ),^[17]^ and would potentially facilitate structural studies, we first assessed the inhibition of *Zea mays* TK by cornexistin (**6**) using an *in vitro* based dose‐response activity assay (Scheme S1). We confirmed *Zea mays* TK inhibition in the double‐digit μM‐range (IC_50_=50±5 μM). This is in reasonable agreement with the previously determined IC_50_ of cornexistin (**6**) towards TK from *Spinacia oleracea* (∼4.5 μM)^[11f]^ and thereby indicates that selectivity is not target based.

### X‐ray co‐crystal structure

**Figure 2:**
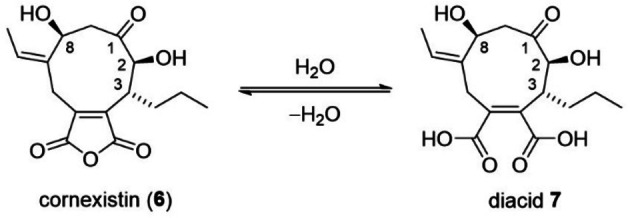
Plausible formation of diacid **7** from cornexistin (**6**) via hydrolysis of the maleic anhydride.

**Figure 3:**
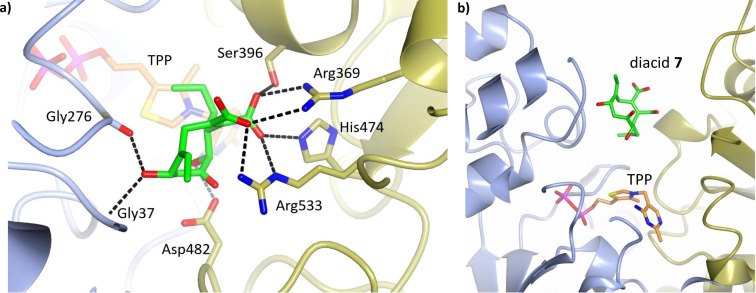
Structural analysis using X‐ray co‐crystal structure (pdb accession code 8CI0). a) Image showing the interaction of diacid **7** (shown in green) with TK; b) Image showing the significant distance between diacid **7** (shown in green) and the TPP cofactor (shown in orange) when both are bound to TK.

To better understand the molecular basis of cornexistin (**6**) binding to its target enzyme, we determined the X‐ray crystal structure of cornexistin (**6**) in complex with TK from *Zea mays* at a resolution of 1.9 Å. In TK, the TPP cofactor is bound in a deep cleft at the interface of the monomers in the dimeric enzyme.^[17]^ The X‐ray co‐crystal structure reveals that cornexistin (**6**) binds to residues from both TK monomers in its diacid form **7** (presumably arising from hydrolysis of the maleic anhydride, Figure 2) thus effectively blocking the entrance to the TK active site (Figure 3a).

The two carboxyl groups of diacid **7** interact with the sidechains from Arg369, Ser396, His474 and Arg533 of the first monomer. In addition, hydrogen bonds are observed from the C2‐hydroxyl group to the sidechain of Asp482 from the same monomer and from the C8‐hydroxyl group to mainchain atoms from Gly37 and Gly276 of the second monomer (Figure 3a). It should be noted that the shortest distance between the atoms from diacid **7** and the TPP cofactor in the crystal structure is 6.6 Å, effectively showing that they are not interacting with each other when engaging TK (Figure 3b).

### Design of simplified cornexistin analogues

**Figure 4:**
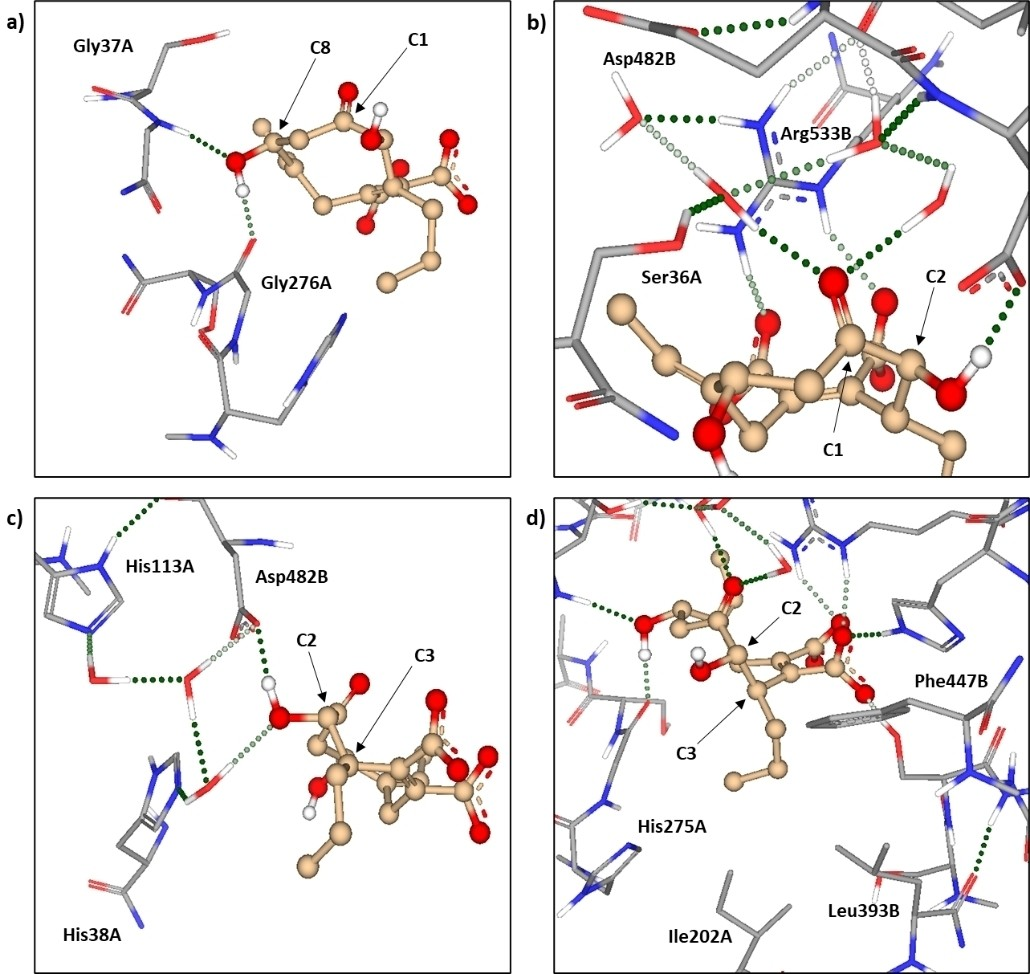
Detailed analysis of the hydrogen bonding interactions of the substituents found on the nine‐membered ring of cornexistin (**6**) with TK; a) Interaction of the C8‐hydroxyl group with the Gly37A and Gly276A residues; b) Interaction of the C1‐carbonyl group via several tetrahedrally coordinated water molecules; c) Interaction of the C2‐hydroxyl group with the Asp482B residue; d) Occupation of the C3‐propyl side chain in a hydrophobic pocket. Hydrogen bonds are drawn in dashed green lines with the darkness of the line indicating the ideality of the H‐bond geometry.

After having successfully obtained an X‐ray co‐crystal structure of cornexistin (**6**) with TK, our attention turned to designing simplified analogues that should be more synthetically viable than the parent compound. Due to the hydrogen bonding interactions of the two carboxyl groups in diacid **7**, as well as the beneficial properties that herbicidal molecules exhibit when weak acids are present in their structure,^[18]^ we decided to retain the maleic anhydride functionality in all of our designed analogues. Keeping this part of the molecule intact also increased the chance that we could retain good binding affinity for TK with our simplified analogues, as several of the hydrogen bonding interactions between the ligand and the enzyme are maintained.^[19]^ Next, we investigated the importance of the C8‐hydroxyl group that is located in the adjacent position to the alkene side chain. This hydroxyl group interacts directly with the backbone amide of Gly37A and Gly276A, thereby stabilizing the protein conformation (Figure 4a). Omitting this hydroxyl group would leave both backbone amides without a hydrogen bond partner which ought to result in a much weaker binding affinity for the corresponding compound. As a result, we postulated that we should keep this residue present in all of our novel analogues. Further examination of the C1‐carbonyl group determined that there was no direct hydrogen bond formation with the TK protein, instead it interacts with the protein via a network of tetrahedrally coordinated water molecules (Figure 4b). As the C1‐carbonyl did not exhibit any direct hydrogen bonds, we hypothesized that it may not be essential. Therefore, this functionality was deemed suitable for removal.

The importance of the C2‐hydroxyl group close to the alkyl moiety was then explored. This hydroxyl group bridges directly to the sidechain of Asp482B and via an internal water molecule to the side chain of His38A (Figure 4c). Omitting the hydroxyl group would leave one carboxylate oxygen of Asp482B without a hydrogen bonding partner and therefore should give rise to a decrease in binding affinity. Nevertheless, the interaction of the C2‐hydroxyl was determined to be less important than that of the C8‐hydroxyl group. As a result, it was also seen as a candidate for removal from the potential analogue structures. Lastly, closer investigation of the C3‐propyl side chain revealed that it provides significant stabilization via the hydrophobic effect, since the side chain is buried in a hydrophobic pocket of the protein‐protein interface (Figure 4d). Thus, omitting the C3‐propyl side chain should give rise to a weaker binding affinity and therefore it should be retained on the majority of our simplified analogues.

**Figure 5:**
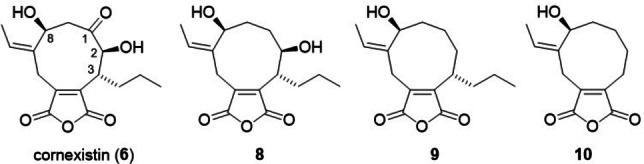
Structures of simplified analogues of cornexistin (**6**) formed by removal of the substituents present at the C1, C2 and C3 positions of the nine‐membered ring.

Taking all of this analysis into consideration, we opted for a design strategy focusing on the systematic removal of the substituents present at the C1, C2 and C3 positions of the nine‐membered ring of cornexistin (**6**), whilst retaining the maleic anhydride, the C7‐alkene group and the C8‐hydroxyl group. This resulted in three simplified analogues (compound **8** where the C1‐carbonyl has been removed, compound **9** which is without the C1‐carbonyl and the C2‐hydroxyl groups and compound **10** which has had all of the substituents from the C1, C2 and C3 positions removed, Figure 5), that could have the potential to preserve at least some of the herbicidal efficacy of the parent compound cornexistin (**6**).

### Synthesis of cornexistin analogues

**Scheme 1:**
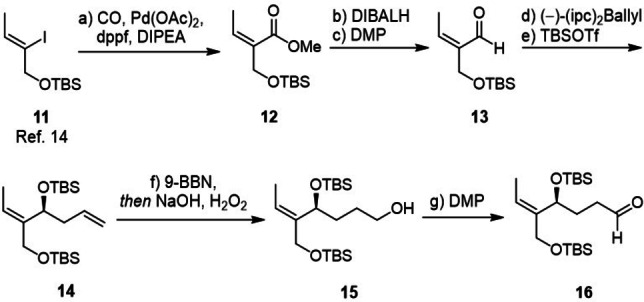
Synthesis of common intermediate **16**. Reagents and conditions: a) Pd(OAc)_2_, dppf, CO, DIPEA, MeOH, 55 °C, 67 %; b) DIBALH, CH_2_Cl_2_, −78 °C; c) DMP, CH_2_Cl_2_, 23 °C, 63 % over two steps; d) (–)‐(ipc)_2_Ballyl, Et_2_O, −78 °C, 71 % (96 % *ee*); e) TBSOTf, 2,6‐lutidine, CH_2_Cl_2_, −78 °C to 23 °C, 98 %; f) 9‐BBN, H_2_O_2_, NaOH, THF, 0 °C to 23 °C, 78 %; g) DMP, CH_2_Cl_2_, 23 °C, 84 %. 9‐BBN=9‐borabicyclo[3.3.1]nonane, DIBALH=diisobutylaluminiumhydride, DIPEA=*N,N‐*diisopropylethylamine, DMP=Dess–Martin periodinane, dppf=1,1′‐bis(diphenylphosphino)ferrocene, ipc=isopinocampheyl, TBS=*tert*‐butyldimethylsilyl, THF=tetrahydrofuran.

For the synthesis of our proposed cornexistin analogues, we planned to rely on the transformations previously employed in the synthesis of the parent natural product.^[14]^ From these studies we knew that it was essential to employ an allylic bromide for the closure of the nine‐membered ring via an intramolecular alkylation.^[20]^ Thus, the exocyclic double bond was conserved allowing us to access the derivatives from common intermediate **16**. The synthesis of the envisioned derivatives commenced with the conversion of known vinyl iodide **11** to aldehyde **13** (Scheme 1). Since **13** was found to be prone to double bond isomerization and direct carbonylation in the presence of a hydride donor resulted in the isolation of a mixture of *E*/*Z*‐isomers, **11** was first converted into the configurationally more stable methyl ester **12**. Reduction (DIBALH) and oxidation of the obtained crude allylic alcohol utilizing Dess–Martin periodinane reliably delivered aldehyde **13**. Chain elongation was achieved by Brown allylation.^[21]^ The stereochemical outcome of the reaction was confirmed by analysis of the corresponding Mosher esters and an enantiomeric excess of 96 % was determined (see Supporting Information for details). Subsequent silylation (TBSOTf, 2,6‐lutidine) of the allylic alcohol gave alkene **14** in good yields. The conversion of **14** into primary alcohol **15** employing a hydroboration/oxidation protocol (9‐BBN, then H_2_O_2_, NaOH) and subsequent oxidation gave aldehyde **16** as a branching point for the preparation of our simplified cornexistin derivatives.

**Scheme 2:**
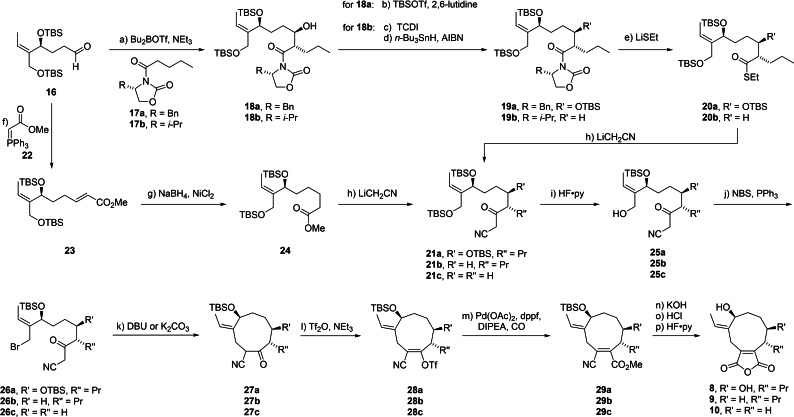
Synthesis of analogues **8**, **9** and **10**. Reagents and conditions: a) **17 a** or **17 b**, Bu_2_BOTf, NEt_3_, −78 °C to 23 °C; b) TBSOTf, 2,6‐lutidine, CH_2_Cl_2_, −78 °C to 23 °C; c) TCDI, THF, 65 °C, 75 % over two steps, (*d.r*. >20 : 1); d) *n*‐Bu_3_SnH, AIBN, toluene, 105 °C, 86 %; e) EtSH, *n*‐BuLi, THF, 0 °C, 80 % over three steps (for **20 a**), (*d.r*. >20 : 1), 84 % (for **20 b**); f) **22**, THF, 60 °C, 96 %; g) NaBH_4_, NiCl_2_, MeOH, 0 °C, 99 %; h) MeCN, *n*‐BuLi, THF, −78 °C, 66 % (for **21 a**), 89 % (for **21 b**), 88 % (for **21 c**); i) HF⋅pyridine, THF/pyridine, 0 °C, 60 % (for **25 a**), 83 % (for **25 b**), 84 % (for **25 c**); j) NBS, PPh_3_, CH_2_Cl_2_, −30 °C to −5 °C, 95 % (for **26 a**), 92 % (for **26 b**), 97 % (for **26 c**); k) DBU (for **26 a**)/K_2_CO_3_ (for **26 b** and **26 c**), MeCN, 23 °C, 42 % (for **27 a**), 65 % (for **27 b**), 38 % (for **27 c**); l) Tf_2_O, NEt_3_, −78 °C, 56 % (for **28 a**), 51 % after three cycles (for **28 b**), 86 % (for **28 c**); m) Pd(OAc)_2_, dppf, CO, DIPEA, DMF, MeOH, 55 °C, 69 % (for **29 a**), 54 % (for **29 b**), 95 % (for **29 c**); n) 10 % KOH in water, *i*‐PrOH, 70 °C; o) 0.2 m aqueous HCl, THF, 23 °C; p) HF⋅pyridine, 0 °C to 23 °C, 46 % over three steps (for **8**), 55 % over three steps (for **9**), 77 % over three steps (for **10**). AIBN=azobisisobutyronitrile, DBU=1,8‐diazabicyclo[5.4.0]undec‐7‐ene, DIPEA=*N,N‐*diisopropylethylamine, DMF=*N*,*N‐*dimethylformamide dppf=1,1′‐bis(diphenylphosphino)ferrocene, NBS=*N*‐bromosuccinimide, TCDI=thiocarbonyldiimidazole, TBS=*tert*‐butyldimethylsilyl, THF=tetrahydrofuran.

For the synthesis of derivative **8**, we continued analogously to the route utilized to access cornexistin (Scheme 2). Aldehyde **16** underwent a highly selective *syn*‐aldol reaction with the *Z*‐enolate derived from oxazolidinone **17 a** (Bu_2_BOTf, NEt_3_) and delivered alcohol **18 a** as a single diastereomer, which was in turn converted into **19 a** by silylation (TBSOTf, 2,6‐lutidine).^[22]^ In the synthesis of derivative **9**, we planned to remove the oxygen of the obtained aldol product following the Barton–McCombie protocol.^[23]^ Therefore, we decided to conduct the Evans aldol reaction with valine‐derived oxazolidinone **17 b**, reasoning that the absence of benzylic C−H bonds should minimize the risk of potential side reactions during the radical deoxygenation. The aldol reaction between aldehyde **16** and **17 b** again proceeded with excellent levels of stereocontrol giving exclusively aldol product **18 b**. Heating of **18 b** with TCDI at 65 °C then resulted in clean conversion to the Barton–McCombie precursor, which could be isolated in 75 % yield over two steps. The following deoxygenation under standard conditions (*n*‐Bu_3_SnH, AIBN, toluene, 105 °C) proceeded smoothly and gave **19 b** in 86 % yield. Cleavage of the auxiliaries in **19 a** and **19 b** was achieved by treatment with freshly prepared LiSEt and furnished the corresponding thioesters **20 a** and **20 b**, respectively. Towards analogue **10**, we subjected aldehyde **16** to Wittig olefination with ylide **22**, giving α,β‐unsaturated ester **23** in almost quantitative yield. Treatment with sodium borohydride in the presence of catalytic nickel(II) chloride effected clean conjugate reduction and delivered ester **24** in excellent yield. The obtained (thio)esters **20 a**, **20 b** and **24** were then individually subjected to the same procedure as developed for the total synthesis of cornexistin (**6**). Treatment with LiCH_2_CN proceeded smoothly and gave β‐ketonitriles **21 a**–**c** in good yields. Selective desilylation of the primary TBS‐groups was achieved by treatment with pyridine hydrofluoride in a mixture of tetrahydrofuran and pyridine at 0 °C. The obtained allylic alcohols **25 a**–**c** were subjected to Appel bromination (NBS, PPh_3_) to deliver the corresponding allylic bromides **26 a**–**c**. Treatment of **26 a** with DBU promoted the key intramolecular alkylation and gave nine‐membered carbocycle **27 a** in mediocre yields (42 %). For **26 b** and **26 c**, K_2_CO_3_ was found to deliver the best results for the ring closure. For the following triflation we observed significant differences in the reactivity among the three substrates. For **27 a** and **27 b**, which bear the propyl side chain next to the ketonitrile moiety, the conversion of the starting material seemed to stagnate after a certain time. Upon workup of the reaction mixture, the triflates were isolated together with significant amounts of unreacted starting material. Triflate **28 a** was thereby isolated in 56 % yield accompanied by 41 % of unreacted **27 a**. The triflation of **27 b** only delivered 26 % of **28 b** and 62 % of unreacted starting material. We therefore subjected the reisolated material to two additional reaction cycles, which resulted in an overall yield of 51 % and secured sufficient amounts to continue the synthesis of derivative **9**. In contrast, **27 c** lacking the propyl substituent underwent smooth triflation and delivered 86 % of the corresponding triflate **28 c** after a single cycle. The obtained triflates were subsequently subjected to carbonylation to afford methyl esters **29 a**–**c**. Treatment of the methyl esters with 10 % aqueous KOH solution in isopropanol at 70 °C resulted in formation of the corresponding imidates, which were sufficiently pure to be used without further purification. Stirring with 0.2 m aqueous hydrochloric acid in tetrahydrofuran effected conversion into the respective anhydrides and finally complete desilylation (pyridine hydrofluoride) furnished cornexistin analogues **8**, **9** and **10** in good yields over three steps (46 %–77 %).

### Biological and biochemical evaluation of cornexistin analogues

With suitable quantities of the cornexistin analogues **8**, **9** and **10** in hand, we embarked on assessing their herbicidal efficacy in greenhouse trials using a variety of potted weed and crop species. We first evaluated the herbicidal efficacy of our synthesized analogues in the warm season segment using post‐emergence application and cornexistin (**6**) for comparison (Table 1). At dose rates of 1280 and 320 grams/hectare (g/ha), cornexistin (**6**) was shown to have broad herbicidal efficacy against the selected grass and dicot species, as well as having excellent selectivity for *Zea mays*. Pleasingly, analogue **8** exhibited a similar level of herbicidal efficacy at 1280 g/ha as that of cornexistin (**6**), whilst also retaining selectivity for *Zea mays*. However, the broad herbicidal profile of analogue **8** was reduced when testing at the lower dosage of 320 g/ha. Good levels of control for the weed species *Setaria viridis*, *Abutilon theophrasti* and *Amaranthus retroflexus* did remain, but efficacy against the grass species *Digitaria sanguinalis* and *Echinochloa crus‐galli* was lost. Unfortunately, the analogues **9** and **10** performed poorly in this trial, with only moderate efficacy against the weed species *Abutilon theophrasti* (analogue **9**) and *Amaranthus retroflexus* (analogue **10**) being observed. We next tested how our simplified analogues performed in the cold season segment, again using post‐emergence application (Table 1). Cornexistin (**6**) displayed broad herbicidal efficacy at 1280 g/ha, with nearly complete control of all of the weed species evaluated. The result was similar at 320 g/ha, but there was a slight reduction in efficacy against the grass weeds *Alopecurus myosuroides*, *Avena fatua* and *Lolium rigidum*. Analogue **8** showed a broad level of herbicidal efficacy in the cold season spectrum at 1280 g/ha, with only a weakness against the dicot weed *Matricaria inodora*. When compound **8** was tested at 320 g/ha a significant reduction of herbicidal efficacy against the monocot weeds was observed. In contrast, the control of the dicot weeds *Kochia scoparia*, *Veronica persica* and *Viola tricolor* was mostly maintained. However, almost no efficacy was observed for *Matricaria inodora*. Analogue **9** exhibited a decent level of control in the cold season dicot spectrum at the highest dose rate (1280 g/ha). However, this was vastly reduced when the dose rate of lowered to 320 g/ha, with only limited efficacy against *Viola tricolor* remaining. In the monocot spectrum compound **9** had minimal effects even at the highest dosage. Analogue **10** was almost completely inactive, bar some minor observable damage against the dicot weeds that were tested at 1280 g/ha.

**Table 1:** Greenhouse post‐emergent herbicidal activity of cornexistin (**6**) and analogues **8**, **9** and **10** in the warm and cold season segments

		Herbicidal activity warm season segment								Herbicidal activity cold season segment							
Compound	Dosage [g/ha]	DIGSA	ECHCG	SETVI	ABUTH	AMARE	PHBPU	POLCO	ZEAMX	ALOMY	AVEFA	LOLRI	KCHSC	MATIN	VERPE	VIOTR	TRZAS
cornexistin (**6**)	1280	5	4	5	5	5	5	5	–	5	5	5	5	5	5	5	5
compound **8**	1280	4	4	4	5	5	5	4	–	5	4	4	5	3	5	5	4
compound **9**	1280	–	1	1	3	1	2	–	–	–	–	–	4	1	4	4	–
compound **10**	1280	–	–	–	1	3	–	–	–	–	–	–	1	1	1	1	–
cornexistin (**6**)	320	4	4	5	5	5	4	4	–	4	3	4	5	4	5	5	3
compound **8**	320	–	–	4	5	4	3	1	–	3	–	1	5	–	5	4	–
compound **9**	320	–	–	1	–	–	–	–	–	–	–	–	1	–	1	3	–
compound **10**	320	–	–	–	–	–	–	–	–	–	–	–	1	–	–	–	–

Rating scale: “5”=100 % inhibition, “4”=80 %–99 % inhibition, “3”=60 %–79 % inhibition, “2”=40 %–59 % inhibition, “1”=20 %–39 % inhibition and “–”=<20 % inhibition. Abbreviations: *Digitaria sanguinalis* (DIGSA), *Echinochloa crus‐galli* (ECHCG), *Setaria viridis* (SETVI), *Abutilon theophrasti* (ABUTH), *Amaranthus retroflexus* (AMARE), *Ipomoea purpurea* (PHBPU), *Polygonum convolvulus* (POLCO), *Zea mays* (ZEAMX), *Alopecurus myosuroides* (ALOMY), *Avena fatua* (AVEFA), *Lolium rigidum* (LOLRI), *Kochia scoparia* (KCHSC), *Matricaria inodora* (MATIN), *Veronica persica* (VERPE), *Viola tricolor* (VIOTR) and *Triticum aestivum* (TRZAS). The color coding for the crop species ZEAMX and TRZAS is inverted to reflect that less inhibition of the crop species is desired.

The greenhouse data are corroborated by the *in vitro* biochemical data that we obtained. Similar to the determination of a dose‐response curve for cornexistin (**6**), the inhibitory potency of the newly synthesized cornexistin analogues (compounds **8**, **9** and **10**) were determined (Table 2 and Figure S1a). The assay data indicate more than a 30‐fold reduction in inhibitory potency for compound **8**, which is missing the C1‐carbonyl group of cornexistin (**6**). Even further reduced inhibitory potency is observed for compounds **9** and **10** with IC_50_ values well above 5 mM (Table 2 and Figure S1) which is in good agreement with the absence of, or weak herbicidal efficacy for these compounds in greenhouse trials. We further analyzed the influence of cornexistin (**6**) and its analogues (compounds **8**, **9** and **10**) on protein stability as a measure for interaction between the compounds and the enzyme using nano differential scanning fluorimetry (nanoDSF). Whilst cornexistin (**6**) and the most effective analogue **8** show a significant stabilization of the protein compared to holoTK in the absence of any compound, compounds **9** and **10** fail to stabilize the enzyme (Table 2 and Figure S1b). This is indicative of reduced or absent binding affinity of the compounds towards TK.

**Table 2:** *In vitro* biochemical analysis of cornexistin (**6**) and the analogues **8**, **9** and **10** against TK.

	Enzymatic assay	NanoDSF
Compound	IC_50_ [μM]	Δ*T*_M_ [°C]
cornexistin (**6**)	50±5	2.5±0.3
compound **8**	1660±150	3.1±0.3
compound **9**	>5000	0.7±0.2
compound **10**	>5000	0.2±0.2

Abbreviations: IC_50_=half maximal inhibitory concentration; DSF=Differential scanning fluorimetry.

## Discussion

When all of this data is processed together, they illustrate that whilst removal of the C1‐carbonyl has only minor detrimental effects on compound binding and efficacy (compound **6** vs. compound **8**), the removal of the C2‐hydroxyl group of cornexistin (**6**) virtually abolishes binding and biological efficacy of the molecule (compounds **6** and **8** vs. **9** and **10**). This observation suggests that the C2‐hydroxyl functionality is crucial for interactions of the compounds with TK. This is no surprise as the crystal structure showed that the C1‐carbonyl group establishes less important contacts to residues in the binding site. In contrast, the C2‐hydroxyl group is prominently involved in a hydrogen bonding network to Asp482 from the first monomer and two adjacent water molecules that in turn are near the reactive ylide of the TPP cofactor. Therefore, the removal of this functional group has a massive detrimental effect on the binding affinity and the *in vivo* efficacy. The additional removal of the C3‐propyl functionality (compounds **6**, **8** and **9** vs. **10**) likely further weakens the binding to TK by decreasing the hydrophobic effect. From our studies we can conclude that any simplified analogue of cornexistin (**6**) that retains good herbicidal efficacy can have the C1‐carbonyl removed. However, the C2‐hydroxyl group and the C3‐propyl group most likely need to be included in the final compound structure. In our study we have not investigated the effects that removal of the C7‐alkenyl group, the C8‐hydroxyl group or the anhydride/diacid functionality can have on herbicidal efficacy and TK target affinity. We postulate that this work may inspire other research groups to continue to investigate novel analogues of the natural product cornexistin (**6**) or stimulate activities in finding completely new inhibitors of TK.

## Conclusions

In summary, we have designed and synthesized three simplified analogues of the herbicidal natural product (+)‐cornexistin (**6**). Guided by an X‐ray co‐crystal structure of cornexistin bound to transketolase from *Zea mays*, we identified the key hydrogen bonding interactions of the compound with the target protein and used this data for the design of our analogues. Using a convergent synthetic route, we were able to access three analogues from the designed compounds that focused on the removal of the substituents in the C1, C2 and C3 positions of the nine‐membered ring. Analysis of these three compounds showed that analogue **8**, that is without the carbonyl group in the C1 position, retained a good level of *in vivo* herbicidal efficacy in both the cold and warm season segments at a dose rate of 1280 g/ha. It also displayed target affinity for transketolase, albeit with a significantly reduced IC_50_ value compared to cornexistin. Our studies show that herbicidally active simplified analogues of cornexistin can be prepared, which we hope will stimulate other research activities to further validate the inhibition of transketolase as a herbicidal mode of action, as well as enabling it as being an innovative crop protection solution of the future.

## Conflict of interest

The authors declare no conflict of interest.

1

## Supporting information

As a service to our authors and readers, this journal provides supporting information supplied by the authors. Such materials are peer reviewed and may be re‐organized for online delivery, but are not copy‐edited or typeset. Technical support issues arising from supporting information (other than missing files) should be addressed to the authors.

Supporting Information

## Data Availability

The data that support the findings of this study are available in the supplementary material of this article.
