# Supplementary material for: Investigations into Simplified Analogues of the Herbicidal Natural Product (+)‐Cornexistin
Source: Chemistry. 2023 May 23;29(39):e202300199. doi: 10.1002/chem.202300199 (PMC7614749; doi:10.1002/chem.202300199)
Supplement: Supplementary file 1 — Supporting Information [file CHEM-29-0-s001.pdf]

Chemistry–A European Journal

Supporting Information

Investigations into Simplified Analogues of the Herbicidal Natural Product (+)-Cornexistin

Christian Steinborn, Aldo Tancredi, Christoph Habiger, Christina Diederich, Jan Kramer, Anna M. Reingruber, Bernd Laber, Jörg Freigang, Gudrun Lange, Dirk Schmutzler, Anu Machettira, Gilbert Besong, Thomas Magauer,* and David M. Barber*

Table of contents

Figure S1 2

Scheme S1 2

Table S1 3

General experimental details 4

Synthesis and characterization data 7

- **Synthesis of aldehyde 16** 7
- **Synthesis of derivative 8** 16
- **Synthesis of derivative 9** 26
- **Synthesis of derivative 10** 37

References 46

^{1}H and ^{13}C NMR spectra 47

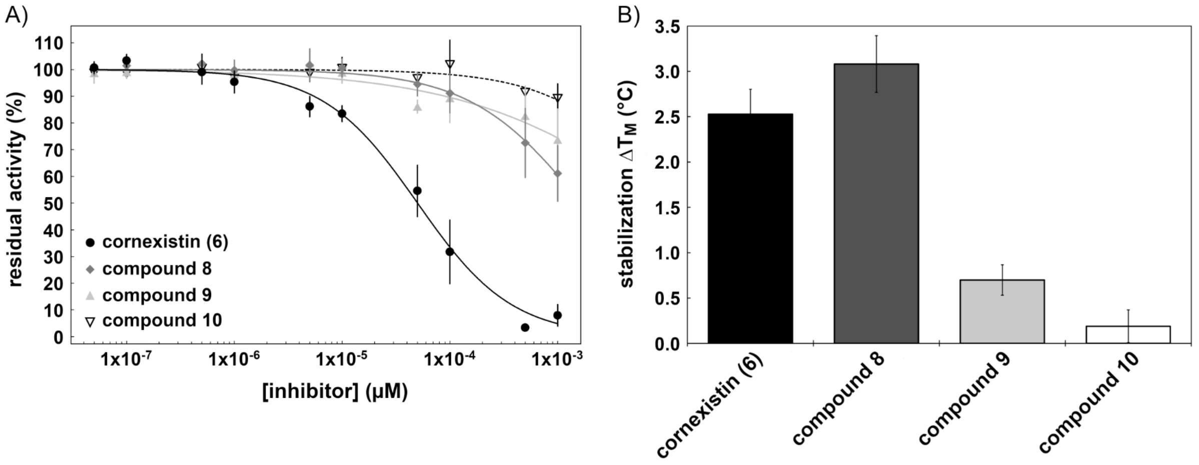

Figure S1. *In vitro* biochemical analysis of cornexistin (**6**) and its analogues (compounds **8**, **9** and **10**). a) Dose-response curves for transketolase from *Zea mays* titrated with cornexistin (**6**, ●), compound **8** (♦), compound **9** (▲) or compound **10** (▽). b) Thermal stabilization plot of transketolase from *Zea mays* in presence of cornexistin (**6**, ■), compound **8** (■), compound **9** (■) or compound **10** (□). The temperature difference relative to the melting temperature of the enzyme at the corresponding DMSO concentration (1 % (v/v) for compound **6**, **8** and **10**, 5 % (v/v) for compound **9**) is plotted.

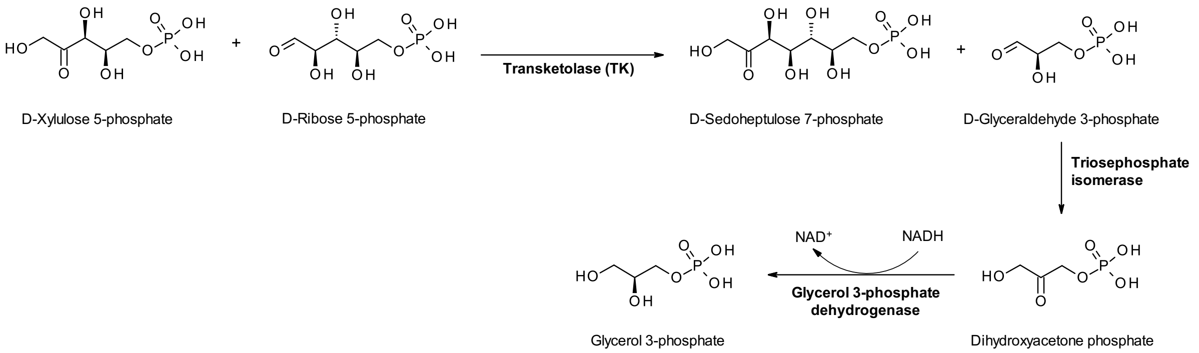

Scheme S1. Reaction path of the coupled enzymatic activity assay. As readout of the enzymatic assay the decrease in UV absorbance at 340 nm resulting from NADH consumption is monitored.

Table S1. X-ray data and structure refinement statistics.

Space group	$P3_121$
Cell axes a, b, c (Å)	135.31, 135.31, 201.44
Resolution (Å)	20-1.9 (2.00-1.90)
No. of observation	947 697 (50 979)
No. of unique reflections	157 602 (18 870)
R_{merge} (%)	6.4 (17.5)
I/σ(I)	19.0 (4.9)
Completeness (%)	94.4 (78.6)
R_{cryst} (%)	15.3 (19.4)
R_{free} (%)	18.4 (25.0)

Values in parentheses denote the highest resolution shell

General experimental details

Protein preparation

Transketolase from *Zea mays* (UniProt Q7SIC9 (TKTC_MAIZE)) was expressed and purified using standard protocols. In brief, the protein was overexpressed in *E. coli* BL21 (DE3) cells from a pET21a-derived plasmid encoding for an N-terminal hexahistidine tag, a tobacco etch virus protease cleavage site and the protein of interest lacking the first 55 amino acids corresponding to the chloroplast transit peptide. The protein was purified by single-step immobilized Ni^{2+}-affinity chromatography in 50 mM TRIS/HCl pH7.5, 20 mM NaCl, 5 mM $MgCl_2$, 50 µM TPP (+ 300 mM imidazole) followed by proteolytic cleavage of the N-terminal tag and a second immobilized Ni^{2+}-affinity chromatography step using the same buffers. Purified protein in 50 mM TRIS/HCl pH7.5, 200 mM NaCl, 5 mM $MgCl_2$, 50 µM TPP was concentrated, and the concentration determined using a NanoDrop™ Lite spectrophotometer (Thermo Scientific) with the absorption at 280 nm equal to 1 mg/mL protein and a conversion factor of 1.267 (accounting for molecular weight and specific extinction coefficient of TK). After addition of 10% (v/v) glycerol, aliquots of TK were snap-frozen in liquid nitrogen and stored at −80 °C until further usage.

Enzymatic assay

Dose-response curves for cornexistin (**6**) and its analogues (compounds **8**, **9** and **10**) were determined in a coupled enzymatic activity assay (Scheme S1) following NADH depletion at 340 nm for 30 minutes using a CLARIOstar® microplate reader (BMG LABTECH GmbH) with 96-well polystyrene flat-bottom microplates (Greiner Bio-One GmbH) at room temperature. Either compound was titrated in the range from 50 µM to 1 mM in 200 µL reaction volume, the mixture further containing both substrates, D-xylulose 5-phosphate and D-ribose 5-phosphate, each at 2 mM final concentration in assay buffer (50 mM sodium phosphate pH7.0, 2 mM $MgCl_2$, 50 mM NaCl, 10% (v/v) glycerol, 10 µM TPP, 2.5 mM NADH). The reaction was initiated by addition of an enzymatic mixture containing TK at 20 nM (active site) and the coupled enzymes triosephosphate isomerase and glycerol 3-phosphate dehydrogenase in excess each at 0.04 U/µL final concentration. All experiments were performed in triplicate and inhibition parameters were derived by fitting to a dose-response 4-parameter logistic model using the XLfit add-in (IDBS) in Microsoft Excel (Microsoft Corporation).

Protein crystallization and data collection

Expression and purification of TK for crystallization experiments followed published protocols.[1] Crystals were grown from 28 % (w/v) PEG 3350, 250 mM ammonium acetate at 18°C by sitting drop vapor diffusion. Before data collection, a crystal was soaked with reservoir solution containing 1 mM cornexistin (**6**) for 30 minutes. Diffraction data were collected on a D8 Venture diffraction system from Bruker. The structure was solved by molecular replacement with MOLREP[2] using the complex of TK

from *Zea mays* with TPP (pdb accession code 1ITZ)[1] as a search model. Manual model building and refinement were iteratively performed with Coot[3] and RefMac5.[4] Data collection and refinement statistics are shown in Table S1. The coordinates and structure factors of the refined model are available for the RCSB Protein Data Bank under accession code 8CI0.

Thermal stability assay

A Prometheus NT.48 instrument (NanoTemper Technolgies) was used to determine melting temperatures of TK from *Zea mays* in absence and presence of cornexistin (**6**) or its analogues (compounds **8**, **9** and **10**). The manufacturer's provided standard capillaries were filled with 10 µL sample containing 2.5 µM (active site) TK and 1 mM cornexistin (**6**) or either of the cornexistin analogues (**8**, **9** and **10**) in nanoDSF buffer (50 mM sodium phosphate pH7.0, 2 mM $MgCl_2$, 50 mM NaCl, 10% (v/v) glycerol, 10 µM TPP, 1 % (v/v) DMSO (5 % (v/v) DMSO for compound **9**)) and placed on the sample holder. As control, sample solutions containing only protein in the respective nanoDSF buffer (1 or 5 % (v/v) DMSO) were used. A temperature gradient of 1 °C/min ranging from 15 to 95 °C was applied, the change of the intrinsic protein fluorescence at 330 and 350 nm was monitored and the ratio 350/330 nm was calculated. The resulting relative melting curves allowed determination of the melting temperatures from their inflection points using the manufacturer's provided software tool. The differences in the melting temperatures relative to the respective DMSO controls were calculated and plotted using Microsoft Excel (Microsoft Corporation).

Herbicidal greenhouse trials

Seeds of mono- and dicotyledonous weed plants and crop plants were sown, in plastic or organic planting pots in sandy loam, covered with soil and grown in a greenhouse under controlled growth conditions. Two to three weeks after sowing, the test plants were sprayed in the single-leaf stage with the test compounds, formulated in the form of emulsifiable concentrates (EC), onto the green plant parts as an emulsion, with the addition of 0.5% of an additive, at an application rate of 600 liters of water/ha. The test plants were placed in the greenhouse for three weeks under optimum growth conditions and then the effect of the compounds was assessed visually in comparison with untreated controls.

General organic synthesis methods

All reactions were carried out with magnetic stirring, and if moisture or air sensitive, under nitrogen or argon atmosphere using standard Schlenk techniques in oven-dried glassware (120 °C oven temperature). If required glassware was further dried under vacuum with a heat-gun at 650 °C. External bath thermometers were used to record all reaction temperatures. Low temperature reactions were carried out in a Dewar vessel filled with acetone/dry ice (T between –78 °C and 0 °C) or distilled water/ice (0 °C). High temperature reactions were conducted using a heated silicon oil bath

or a metal block in reaction vessels equipped with a reflux condenser or in a pressure tube. Tetrahydrofuran (THF) was distilled over sodium/potassium alloy prior to use. All other solvents were purchased from Acros Organics as 'extra dry' reagents. All other reagents with a purity > 95% were obtained from commercial sources (Sigma Aldrich, Acros, Alfa Aesar and others) and used without further purification unless otherwise stated.

Flash column chromatography (FCC) was carried out with Merck silica gel 60 (0.040–0.063 mm). Analytical thin layer chromatography (TLC) was carried out using Merck silica gel 60 F254 glass-backed plates or aluminum foils and visualized under UV light at 254 nm. Staining was performed with ceric ammonium molybdate (CAM), an aqueous potassium permanganate solution or by staining with an aqueous anisaldehyde solution and subsequent heating.

NMR spectra (^{1}H NMR, ^{13}C NMR and ^{19}F NMR) were recorded in deuterated chloroform ($CDCl_3$), deuterated methanol (CD_3OD) or deuterated benzene (C_6D_6) on a Bruker Avance III HD 400 MHz spectrometer equipped with a CryoProbe™, a Bruker Avance Neo 400 MHz spectrometer, an Agilent 500 DD2 500 MHz spectrometer or a Bruker Avance II 600 MHz spectrometer and are reported as follows: chemical shift δ in ppm (multiplicity, coupling constant *J* in Hz, number of protons) for ^{1}H NMR spectra and chemical shift δ in ppm for ^{13}C NMR spectra. Multiplicities are abbreviated as follows: s = singlet, d = doublet, t = triplet, q = quartet, p = quintet, br = broad, m = multiplet, or combinations thereof. Residual solvent peaks of $CDCl_3$ (δ_H = 7.26 ppm, δ_C = 77.16 ppm), CD_3OD (δ_H = 3.31 ppm, δ_C = 49.00 ppm) and C_6D_6 (δ_H = 7.16 ppm, δ_C = 128.06ppm) were used as internal reference. NMR spectra of key intermediates were assigned using information ascertained from 2D-NMR experiments.

High resolution mass spectra (HRMS) were recorded on Thermo Scientific™ LTQ Orbitrap XL™ Hybrid Ion Trap-Orbitrap Mass Spectrometer at the Institute of Organic Chemistry and Center for Molecular Biosciences, University of Innsbruck.

Infrared spectra (IR) were recorded from 4000 cm^{-1} to 450 cm^{-1} on a Bruker™ ALPHA FT-IR Spectrometer. Samples were prepared as a neat film or a film by evaporation of a solution in $CDCl_3$.

Optical rotation values were recorded on a Schmidt+Haensch UniPol L1000 Peltier polarimeter. The specific rotation is calculated as follows: $[\alpha]_{\lambda}^{T} = \frac{\alpha \times 100}{c \times d}$. Thereby, the wavelength λ is reported in nm and the measuring temperature in °C. α represents the recorded optical rotation, *c* the concentration of the analyte in 10 mg/mL and *d* the length of the cuvette in dm. Thus, the specific rotation is given in $10^{-1}\cdot deg\cdot cm^2\,g^{-1}$. Use of the sodium *D* line (λ = 589 nm) is indicated by *D* instead of the wavelength in nm. The sample concentration as well as the solvent is reported in the relevant section of the experimental part.

All yields are isolated, unless otherwise specified.

Synthesis and characterization data

Synthesis of aldehyde 16

Methyl ester 12

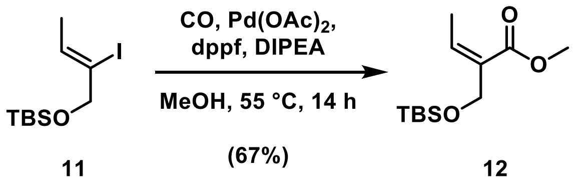

11 12

Note: Methanol was degassed via freeze-pump-thaw (three cycles) prior to use.

Palladium(II) acetate (360 mg, 1.60 mmol, 10.0 mol%), 1,1'-bis(diphenylphosphino)-ferrocene (888 mg, 1.60 mmol, 10.0 mol%) and *N,N*-diisopropylethylamine (2.80 mL, 16.0 mmol, 1.00 equiv) were sequentially added to a solution of **11** (5.00 g, 16.0 mmol, 1 equiv) in methanol (160 mL). The flask was fitted with a balloon filled with carbon monoxide gas, and the solution was sparged with carbon monoxide for 10 minutes. The black reaction mixture was then heated to 55 °C under a carbon monoxide atmosphere. After 14 h, water (300 mL) and dichloromethane (300 mL) were added to the reaction mixture. The layers were separated, and the aqueous phase was extracted with dichloromethane (2 × 200 mL). The combined organic phases were dried over magnesium sulfate and the dried solution was filtrated. The filtrate was concentrated, and the residue was purified by flash column chromatography on silica gel (5% diethyl ether in pentane) to give **12** (2.63 g, 67%) as a colorless oil.

TLC (10% diethyl ether in pentane): R_f = 0.64 (UV, CAM).
^{1}H-NMR (400 MHz, $CDCl_3$): δ = 6.35 (qt, *J* = 7.3, 1.6 Hz, 1H), 4.34 – 4.28 (m, 2H), 3.74 (s, 3H), 2.04 (dt, *J* = 7.3, 1.6 Hz, 3H), 0.91 (s, 9H), 0.07 (s, 6H) ppm.
^{13}C-NMR (101 MHz, $CDCl_3$): δ = 167.3, 137.3, 131.5, 63.5, 51.2, 26.1, 18.5, 15.5, –5.2 ppm.
IR (Diamond-ATR, neat): 2956, 2929, 2856, 1764, 1251, 1202, 1101, 836, 776, 671.
HRMS (ESI) calc. for $C_{12}H_{24}NaO_3Si^+$ $[M+Na]^+$: 267.1387; found: 267.1381.

Aldehyde 13

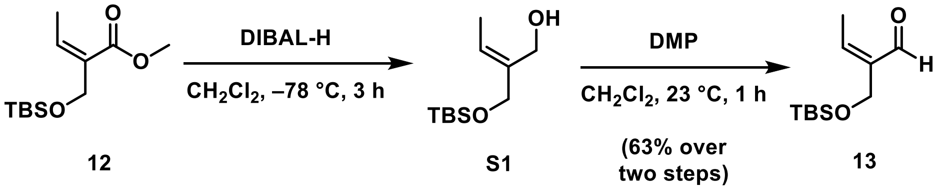

A solution of diisobutylaluminum hydride (1.00 M in dichloromethane, 41.6 mL, 41.6 mmol, 2.50 equiv) was added slowly to a solution of **12** (4.07 g, 16.7 mmol, 1 equiv) in dichloromethane (170 mL) at –78 °C. After 3 h, the reaction mixture was poured into an aqueous saturated sodium potassium tartrate solution (250 mL) and the mixture was stirred vigorously for 1 h. The layers were separated and the aqueous phase was extracted with dichloromethane (2 × 150 mL). The combined organic phases were dried over magnesium sulfate and the dried solution was filtrated. The filtrate was concentrated to give crude alcohol **S1** which was used in the following oxidation step without further purification.

Dess–Martin periodinane (8.03 g, 18.4 mmol, 1.10 equiv) was added to a solution of crude **S1** (assumed 16.7 mmol, 1 equiv) in dichloromethane (170 mL) at 23 °C. After 1 h, water (200 mL) was added to the reaction mixture. The layers were separated and the aqueous phase was extracted with dichloromethane (2 × 150 mL). The combined organic phases were dried over magnesium sulfate and the dried solution was filtrated. The filtrate was concentrated and the residue was purified by flash column chromatography on silica gel (8% diethyl ether in pentane) to give **13** (2.26 g, 63% over two steps) as a colorless oil.

TLC (15% ethyl acetate in cyclohexane): R_f = 0.53 (UV, Permanganate)

^{1}H-NMR (400 MHz, $CDCl_3$): δ = 10.15 (s, 1H), 6.82 (*q*, *J* = 7.7 Hz, 1H), 4.29 (s, 2H), 2.15 (d, *J* = 7.7 Hz, 3H), 0.89 (s, 9H), 0.05 (s, 6H) ppm.

^{13}C-NMR (101 MHz, $CDCl_3$): δ = 190.1, 142.9, 139.2, 60.4, 26.0, 18.5, 12.9, –5.4.

IR (Diamond-ATR, neat): 2954, 2929, 2857, 1674, 1252,1222, 1108, 834, 775, 671.

HRMS (ESI) calc. for $C_{11}H_{22}NaO_2Si^+$ $[M+Na]^+$: 237.1287; found: 237.1249.

Alcohol S2

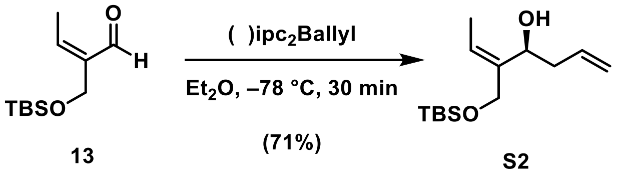

A solution of (−)-Ipc_2B(allyl)borane (1.00 M in pentane, 13.3 mL, 13.3 mmol, 1.10 equiv) was added slowly to a solution of **13** (2.61 g, 12.2 mmol, 1 equiv) in diethyl ether (50 mL) at –78 °C. After 30 min, the reaction mixture was warmed to 23 °C, the solvent was evaporated and the residue was redissolved in pentane (27 mL). Ethanolamine (2.23 mL, 36.5 mmol, 3.00 equiv) was added to the resulting solution and the reaction mixture was stirred vigorously. After 12 h, the mixture was filtered through a plug of Celite and the filter cake was thoroughly washed with pentane (75 mL). The filtrate was concentrated, and the residue was purified by flash column chromatography on silica gel (10% diethyl ether in pentane) to give **S2** (2.21 g, 71%) as a colorless oil.

TLC (20% diethyl ether in pentane): R_f = 0.31 (UV, CAM).
^{1}H-NMR (400 MHz, $CDCl_3$): δ = 5.80 (ddt, *J* = 17.2, 10.2, 7.1 Hz, 1H), 5.61 – 5.50 (m, 1H), 5.16 – 4.98 (m, 2H), 4.64 (q, *J* = 6.7 Hz, 1H), 4.41 (dt, *J* = 11.8, 1.3 Hz, 1H), 4.10 (d, *J* = 11.8 Hz, 1H), 3.25 (d, *J* = 6.4 Hz, 1H), 2.57 – 2.43 (m, 1H), 2.39 – 2.25 (m, 1H), 1.69 – 1.64 (m, 3H), 0.91 (s, 9H), 0.10 (s, 3H), 0.09 (s, 3H) ppm.
^{13}C-NMR (101 MHz, $CDCl_3$): δ = 138.7, 135.2, 124.1, 117.1, 69.7, 66.7, 41.1, 26.0, 18.3, 13.2, –5.3, –5.3 ppm.
IR (Diamond-ATR, neat): 3443, 2954, 2929, 2857, 1254, 1094, 1052, 1004, 836, 776 ppm.
HRMS (ESI) calc. for $C_{14}H_{28}NaO_2Si^+$ $[M+Na]^+$: 279.1751; found: 279.1748.
$[\alpha]_D^{20}$: –13.5 (c = 0.45, CH_2Cl_2).

(*R*)-Mosher ester S3

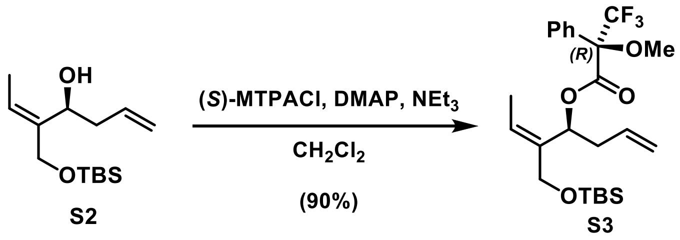

N,N-Dimethylaminopyridine (962 µg, 7.80 µmol, 0.200 equiv) and triethylamine (16.3 µL, 117 µmol, 3.00 equiv) was added to a solution of **S2** (10.0 mg, 39.0 µmol, 1 equiv) and (*S*)-(–)-α-methoxy-α-(trifluoromethyl)phenylacetyl chloride (11.1 µL, 58.5 µmol, 1.50 equiv) in dichloromethane (1mL) at 23 °C. After 16 h, saturated aqueous sodium bicarbonate solution (10 mL) and dichloromethane (10 mL) were added. The layers were separated and the aqueous layer was extracted with dichloromethane (2 × 10 mL). The combined organic phases were dried over magnesium sulfate and the dried solution was filtrated. The filtrate was concentrated and the residue was purified by flash column chromatography on silica gel (40% diethyl ether in pentane) to give **S3** (16.6 mg, 90%) as a colorless oil.

TLC (40% diethyl ether in pentane): R_f = 0.34 (UV, CAM).
^{1}H–NMR (400 MHz, $CDCl_3$): δ = 7.52 – 7.47 (m, 2H), 7.41 – 7.34 (m, 3H), 5.86 (dd, *J* = 8.8, 5.8 Hz, 1H), 5.81 – 5.68 (m, 2H), 5.17 – 5.07 (m, 2H), 4.02 (dt, *J* = 13.1, 1.3 Hz, 1H), 3.90 (dt, *J* = 13.1, 1.4 Hz, 1H), 3.56 (d, *J* = 1.3 Hz, 3H), 2.70 – 2.61 (m, 1H), 2.52 – 2.44 (m, 1H), 1.69 (dt, *J* = 7.1, 1.5 Hz, 3H), 0.87 (s, 9H), –0.01 (s, 6H) ppm.
^{13}C-NMR (101 MHz, $CDCl_3$): δ = 165.8, 135.3, 133.5, 132.4, 129.5, 128.3, 127.3 (d, *J* = 1.5 Hz), 125.2, 123.4 (q, *J* = 288.6 Hz), 118.2, 84.5 (q, *J* = 27.6 Hz), 74.2, 63.3, 55.7 (d, *J* = 1.4 Hz), 37.7, 25.9, 18.3, 13.2, –5.5, –5.5 ppm.
^{19}F-NMR (376 MHz, $CDCl_3$) δ = 71.4 ppm.
IR (Diamond–ATR, neat): 2953, 2928, 2856, 1747, 1254, 1186, 1170, 1081, 838, 777 cm^{-1}.
HRMS (ESI) calc. for $C_{24}H_{35}F_3NaO_4Si^+$ $[M+Na]^+$: 495.2149; found: 495.2147.
$[\alpha]_D^{20}$: 32.1 (c = 0.047, CH_2Cl_2).

(*S*)-Mosher ester S4

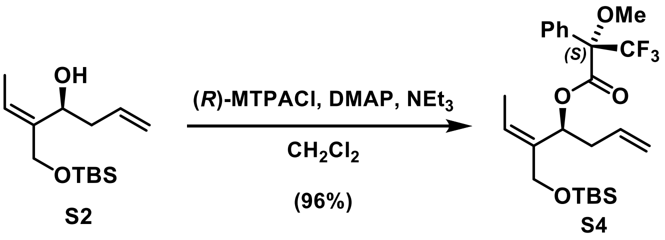

N,N-Dimethylaminopyridine (972 µg, 7.88 µmol, 0.200 equiv) and triethylamine (16.4 µL, 118 µmol, 3.00 equiv) was added to a solution of **S2** (10.1 mg, 39.4 µmol, 1 equiv) and (*R*)-(–)-α-methoxy-α-(trifluoromethyl)phenylacetyl chloride (11.2 µL, 59.1 µmol, 1.50 equiv) in dichloromethane (1mL) at 23 °C. After 16 h, saturated aqueous sodium bicarbonate solution (10 mL) and dichloromethane (10 mL) were added. The layers were separated and the aqueous layer was extracted with dichloromethane (2 × 10 mL). The combined organic phases were dried over magnesium sulfate and the dried solution was filtrated. The filtrate was concentrated and the residue was purified by flash column chromatography on silica gel (40% diethyl ether in pentane) to give **S4** (17.8 mg, 96%) as a colorless oil.

TLC (40% diethyl ether in pentane): R_f = 0.35 (UV, CAM).
^{1}H–NMR (400 MHz, $CDCl_3$): δ = 7.51 – 7.45 (m, 2H), 7.42 – 7.34 (m, 3H), 5.93 (dd, *J* = 8.5, 6.1 Hz, 1H), 5.83 – 5.75 (m, 1H), 5.72 – 5.60 (m, 1H), 5.10 – 5.00 (m, 2H), 4.18 (dt, *J* = 13.3, 1.4 Hz, 1H), 4.08 (dt, *J* = 13.4, 1.5 Hz, 1H), 3.50 (d, *J* = 1.3 Hz, 3H), 2.63 – 2.54 (m, 1H), 2.46 – 2.38 (m, 1H), 1.76 (dt, *J* = 7.2, 1.5 Hz, 3H), 0.89 (s, 9H), 0.03 (s, 6H) ppm.
^{13}C-NMR (101 MHz, $CDCl_3$): δ = 165.9, 135.3, 133.0, 132.4, 129.5, 128.3, 127.5 (d, *J* = 1.5 Hz), 125.1, 123.4 (d, *J* = 288.5 Hz), 118.2, 84.6 (q, *J* = 27.9 Hz), 73.7, 63.3, 55.3 (d, *J* = 1.8 Hz), 37.6, 25.9, 18.3, 13.3, –5.4, –5.5 ppm.
^{19}F-NMR (376 MHz, $CDCl_3$) δ = 71.3 ppm.
IR (Diamond–ATR, neat): 2953, 2929, 2857, 1746, 1253, 1185, 1170, 1106, 1082, 838 cm^{-1}.
HRMS (ESI) calc. for $C_{24}H_{35}F_3NaO_4Si^+$ $[M+Na]^+$: 495.2149; found: 495.2142.
$[\alpha]_D^{20}$: –22.5 (c = 0.12, CH_2Cl_2).

Mosher ester analysis of S3 and S4

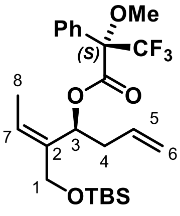

S4

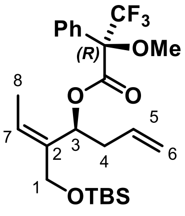

S3

Table S2. Mosher ester analysis of alcohol **S2**.

Position of H	(*S*)-Mosher ester S4	(*R*)-Mosher ester S3	$\Delta\delta = \delta_S - \delta_R$
**1a**	4.18	4.02	0.16
**1b**	4.08	3.90	0.18
**3**	5.93	5.86	0.07
**4a**	2.59	2.65	–0.06
**4b**	2.42	2.48	–0.06
**5**	5.65	5.74	–0.09
**6**	5.04	5.11	–0.07
**7**	5.79	5.74	0.05
**8**	1.76	1.69	0.07

Analysis of the shift differences Δδ shows that the protons at carbons 1, 7 and 8 show positive values, while the difference for the protons at carbons 4, 5 and 6 is negative. In accordance with the reported model, this confirms that the allylation delivers alcohol **S2** as the expected (*S*)-enantiomer.[5]

Determination of the *ee* of alcohol S2

The enantiomeric excess of alcohol **S2** was determined by integration of the ^{19}F-signal of the CF_3-group of the obtained Mosher esters.

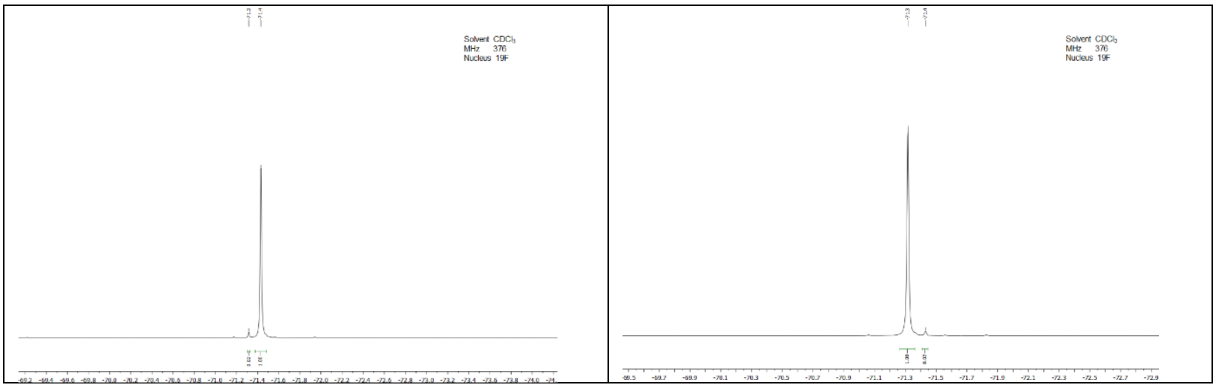

Figure S2. ^{19}F-NMR spectrum of (*R*)-Mosher ester **S3** (left) and (*S*)-Mosher ester **S4** (right)

Integration of the ^{19}F signals, shows a ratio of 50:1 between the desired (*S*),(*R*)-diastereomer and the undesired (*R*),(*R*)-diastereomer for **S3**. The same ratio between the desired (*S*),(*S*)-diastereomer and the undesired (*R*),(*S*)-diastereomer is observed for **S4**. This corresponds to an enantiomeric excess of 96%.

Silyl ether 14

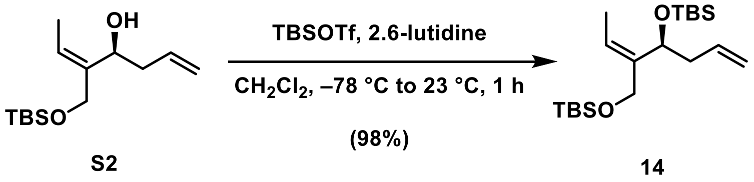

Tert-butyldimethylsilyl trifluoromethanesulfonate (4.35 mL, 18.6 mmol, 1.20 equiv) was added to a solution of **S2** (3.97 g, 15.5 mmol, 1 equiv) and 2,6-lutidine (3.31 mL, 27.9 mmol, 1.80 equiv) in dichloromethane (150 mL) at –78 °C. After 20 min, the reaction mixture was allowed to warm to 23 °C. After 40 min, water (200 mL) and dichloromethane (100 mL) were added. The layers were separated and the aqueous phase was extracted with dichloromethane (2 × 100 mL). The combined organic phases were dried over magnesium sulfate and the dried solution was filtrated. The filtrate was concentrated and the residue was purified by flash column chromatography on silica gel (5% diethyl ether in pentane) to give **14** (5.64 g, 98%) as a colorless oil.

TLC (5% diethyl ether in pentane): R_f = 0.73 (CAM).
^{1}H-NMR (400 MHz, $CDCl_3$): δ = 5.77 (ddt, *J* = 17.3, 10.1, 7.2 Hz, 1H), 5.59 – 5.50 (m, 1H), 5.08 – 4.96 (m, 2H), 4.58 (dd, *J* = 7.9, 5.8 Hz, 1H), 4.28 – 4.12 (m, 2H), 2.43 – 2.32 (m, 1H), 2.28 – 2.19 (m, 1H), 1.65 (dt, *J* = 7.1, 1.7 Hz, 3H), 0.92 (s, 9H), 0.88 (s, 9H), 0.07 (s, 6H), 0.04 (s, 3H), –0.01 (s, 3H).
^{13}C-NMR (101 MHz, $CDCl_3$): δ = 140.6, 135.8, 118.6, 116.7, 70.2, 62.8, 41.9, 26.2, 26.0, 18.6, 18.3, 13.1, –4.7, –4.8, –5.2, –5.2.
IR (Diamond-ATR, neat): 2955, 2929, 2857, 1641, 1252, 1067, 1005, 912, 831, 772.
HRMS (ESI) calc. for $C_{20}H_{42}NaO_2Si_2^+$ $[M+Na]^+$: 393.2621; found: 393.2611.
$[\alpha]_D^{20}$: –19.2 (c = 0.99, CH_2Cl_2).

Alcohol 15

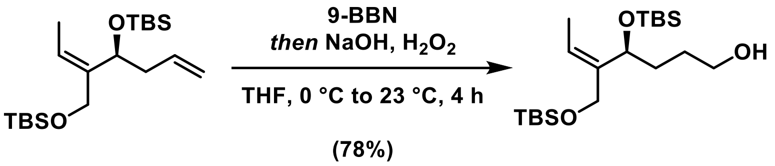

14 15

A solution of 9-borabicyclo[3.3.1]nonane (0.50 M in tetrahydrofuran, 23.2 mL, 11.6 mmol, 1.50 equiv) was added to a solution of **14** (2.87 g, 7.74 mmol, 1 equiv) in tetrahydrofuran (80 mL) at 0 °C. After 16 h, the reaction mixture was allowed to warm to 23 °C. After 1 h, the reaction mixture was recooled to 0 °C and aqueous solutions of sodium hydroxide (10wt.%, 32 mL) and hydrogen peroxide (30wt.%, 13 mL) were added sequentially under vigorous stirring. After 4 h, water (150 mL) and dichloromethane (150 mL) were added. The layers were separated and the aqueous phase was extracted with dichloromethane (2 × 100 mL). The combined organic phases were dried over magnesium sulfate and the dried solution was filtrated. The filtrate was concentrated and the residue was purified by flash column chromatography on silica gel (35% diethyl ether in pentane) to give **15** (2.36 g, 78%) as a colorless oil.

TLC (20% diethyl ether in pentane): R_f = 0.19 (CAM).
^{1}H-NMR (400 MHz, $CDCl_3$): δ = 5.55 (q, *J* = 7.0 Hz, 1H), 4.56 (dd, *J* = 7.4, 5.6 Hz, 1H), 4.22 (dt, *J* = 13.9, 1.6 Hz, 1H), 4.14 (dt, *J* = 13.9, 1.6 Hz, 1H), 3.63 (t, *J* = 6.2 Hz, 2H), 1.74 – 1.61 (m, 5H), 1.61 – 1.45 (m, 3H), 0.91 (s, 9H), 0.88 (s, 9H), 0.06 (s, 6H), 0.05 (s, 3H), –0.01 (s, 3H) ppm.
^{13}C-NMR (101 MHz, $CDCl_3$): δ = 140.9, 118.9, 70.1, 63.1, 62.8, 33.4, 29.5, 26.2, 26.0, 18.6, 18.3, 13.1, –4.7, –4.9, –5.2, –5.2.ppm.
IR (Diamond-ATR, neat): 3307, 2955, 2929, 2857, 1743, 1472, 1252, 1057, 836, 775.
HRMS (ESI) calc. for $C_{20}H_{44}NaO_3Si_2^+$ $[M+Na]^+$: 411.2721; found: 411.2720.
$[\alpha]_D^{20}$: –21.4 (c = 0.68, CH_2Cl_2).

Aldehyde 16

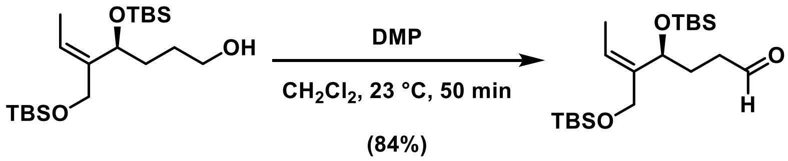

15 16

Dess-Martin periodinane (5.41 g, 12.4 mmol, 1.20 equiv) was added to a solution of **15** (4.01 g, 10.3 mmol, 1 equiv) in dichloromethane (100 mL) at 23 °C. After 50 min, water (100 mL) was added to the reaction mixture. The layers were separated and the aqueous phase was extracted with dichloromethane (2 × 75 mL). The combined organic phases were dried over magnesium sulfate and the dried solution was filtrated. The filtrate was concentrated and the residue was purified by flash column chromatography on silica gel (10% diethyl ether in pentane) to give **16** (3.36 g, 84%) as a colorless oil.

TLC (10% diethyl ether in pentane): R_f = 0.50 (UV, CAM).
^{1}H-NMR (400 MHz, $CDCl_3$): δ = 9.77 (t, *J* = 1.5 Hz, 1H), 5.58 (q, *J* = 7.1 Hz, 1H), 4.58 (dd, *J* = 8.1, 5.8 Hz, 1H), 4.26 – 4.18 (m, 1H), 4.16 – 4.07 (m, 1H), 2.49 – 2.42 (m, 2H), 2.03 – 1.89 (m, 1H), 1.87 – 1.74 (m, 1H), 1.66 (dt, *J* = 7.1, 1.4 Hz, 3H), 0.91 (s, 9H), 0.88 (s, 9H), 0.06 (s, 6H), 0.04 (s, 3H), –0.02 (s, 3H) ppm.
^{13}C-NMR (101 MHz, $CDCl_3$): δ = 202.6, 140.3, 120.1, 69.3, 62.9, 40.8, 29.4, 26.1, 25.9, 18.6, 18.3, 13.2, –4.8, –5.0, –5.2, –5.2 ppm.
IR (Diamond-ATR, neat): 2954, 2929, 2857, 1728, 1472, 1252, 1086, 1005, 834, 774.
HRMS (ESI) calc. for $C_{20}H_{42}NaO_3Si_2^+$ $[M+Na]^+$: 409.2565; found: 409.2565.
$[\alpha]_D^{20}$: –17.8 (c = 0.53, CH_2Cl_2).

Synthesis of derivative 8

Alcohol 18a

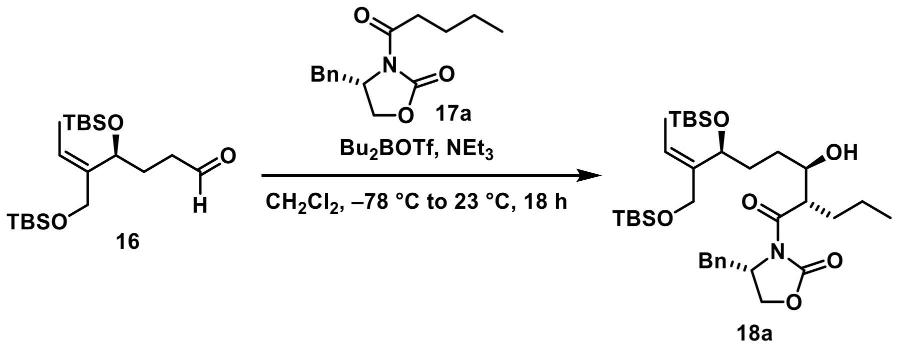

A solution of dibutylboryl trifluoromethanesulfonate (1 M in dichloromethane, 4.94 mL, 4.94 mmol, 1.18 equiv) was added to a solution of **17a** (1.21 g, 4.61 mmol, 1.10 equiv) in dichloromethane (50 mL) at –78 °C. After 15 min, triethylamine (728 µL, 5.24 mmol, 1.25 equiv) was added to the orange solution. After 15 min, the solution was allowed to warm to 0 °C. After 1 h, the reaction mixture was cooled to –78 °C and a solution of **16** (1.61 g, 4.19 mmol, 1 equiv) in dichloromethane (30 mL) was added. After complete addition, the reaction mixture was allowed to slowly warm to 23 °C. After 18 h, the reaction mixture was cooled to 0 °C and 3 mL of a 2:1 mixture of pH = 7 phosphate buffer/methanol and 3 mL of a 2:1 mixture of hydrogen peroxide (30% in water)/methanol were subsequently added. After 1 h, water (70 mL) was added and the layers were separated. The aqueous phase was extracted with dichloromethane (2 × 50 mL). The combined organic phases were dried over magnesium sulfate and the dried solution was filtrated. The filtrate was concentrated to give crude aldol product **18a**, which was clean enough to be used in the following step without further purification.

TLC (30% diethyl ether in pentane): R_f = 0.16 (UV, CAM).

Silyl ether 19a

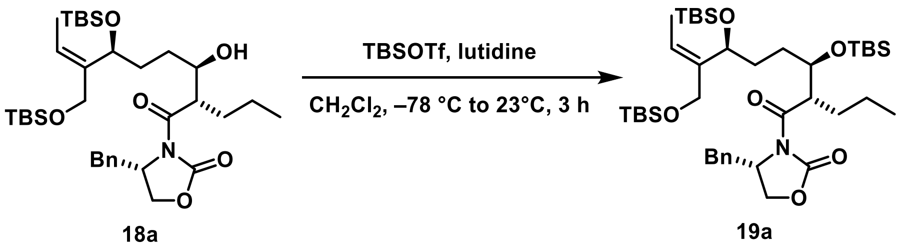

Tert-butyldimethylsilyl trifluoromethanesulfonate (1.23 mL, 5.24 mmol, 1.25 equiv) was added dropwise to a solution of **18a** (assumed 4.19 mmol) and 2,6-lutidine (996 µL, 8.38 mmol, 2.00 equiv) in dichloromethane (60 mL) at –78 °C. After 30 min, the reaction mixture was allowed to warm to 23 °C. After 2.5 h, water (100 mL) was added and the layers were separated. The aqueous phase was extracted with dichloromethane (2 × 70 mL). The combined organic phases were dried over magnesium sulfate and the dried solution was filtrated. The filtrate was concentrated and the residue was filtered over a short plug of silica gel eluting with 10% diethyl ether in pentane. Evaporation of the solvent gave **19a** which was used in the following step without further purification

TLC (10% diethyl ether in pentane): R_f = 0.78 (UV, CAM).

Thioester 20a

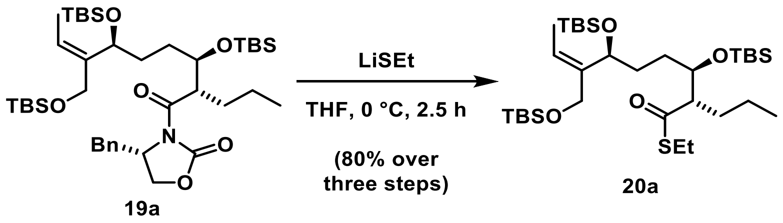

A solution of *n*-butyllithium (2.50 M in hexanes, 5.70 mL, 14.2 mmol, 3.40 equiv) was added dropwise to a solution of ethanethiol (1.57 mL, 1.31 mmol, 5.00 equiv) in tetrahydrofuran (70 mL) at –78 °C. After 5 min, the white suspension was allowed to warm to 0 °C. After 20 min, a solution of **19a** (assumed 4.19 mmol) in tetrahydrofuran (10 mL) was added to the reaction mixture. After 2.5 h, aqueous saturated ammonium chloride solution (100 mL) and dichloromethane (100 mL) were added to the slightly yellow mixture. The layers were separated and the aqueous phase was extracted with dichloromethane (2 × 75 mL). The combined organic phases were dried over magnesium sulfate and the dried solution was filtrated. The filtrate was concentrated and the residue was purified by flash column chromatography on silica gel (5% diethyl ether in pentane) to give **20a** (2.17 g, 80% over three steps) as a yellowish oil.

TLC (5% diethyl ether in pentane): R_f = 0.78 (UV, CAM).
^{1}H–NMR (400 MHz, $CDCl_3$): δ = 5.51 (q, *J* = 7.0 Hz, 1H), 4.46 (dd, *J* = 8.1, 5.2 Hz, 1H), 4.18 (s, 2H), 3.82 (dt, *J* = 7.2, 4.7 Hz, 1H), 2.85 (qd, *J* = 7.4, 1.2 Hz, 2H), 2.68 (ddd, *J* = 10.0, 7.3, 4.1 Hz, 1H), 1.71 – 1.48 (m, 8H), 1.33 (dtdd, *J* = 18.2, 12.7, 6.2, 3.8 Hz, 2H), 1.28 – 1.17 (m, 1H), 1.23 (t, *J* = 7.4 Hz, 3H), 0.91 (s, 9H), 0.90 – 0.86 (s + t + s, 9H + 3H +9H), 0.05 (s, 9H), 0.04 (s, 3H), 0.02 (s, 3H), –0.03 (s, 3H) ppm.
^{13}C–NMR (101 MHz, $CDCl_3$): δ = 201.9, 141.1, 117.4, 73.4, 70.5, 62.3, 59.6, 31.9, 31.7, 31.7, 26.2, 26.1, 26.0, 23.4, 20.8, 18.6, 18.3, 18.3, 14.9, 14.3, 13.0, –4.1, –4.3, –4.8, –4.9, –5.2, –5.2 ppm.
IR (Diamond–ATR, neat): 2956, 2929, 2857, 1685, 1472, 1254, 1104, 1071, 836, 774 cm^{-1}.
HRMS (ESI) calc. for $C_{33}H_{70}NaO_4SSi_3^+$ $[M+Na]^+$: 669.4195; found: 669.4186.
$[\alpha]_D^{20}$: 0.6 (c = 0.69, CH_2Cl_2).

β–keto nitrile 21a

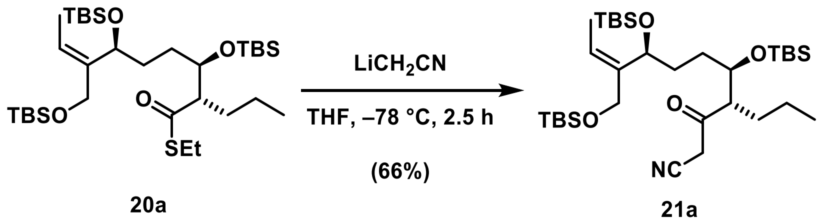

A solution of *n*-butyllithium (2.50 M in hexanes, 4.82 mL, 12.8 mmol, 3.60 equiv) was added dropwise to a solution of acetonitrile (706 µL, 13.4 mmol, 4.00 equiv) in tetrahydrofuran (80 mL) at –78 °C. After 30 min, a solution of **20a** (2.17 g, 3.35 mmol, 1 equiv) in tetrahydrofuran (10 mL) was added to the reaction mixture. After 2.5 h, aqueous saturated ammonium chloride solution (150 mL) and dichloromethane (150 mL) were added to the yellow reaction mixture. The layers were separated and the aqueous phase was extracted with dichloromethane (2 × 100 mL). The combined organic phases were dried over magnesium sulfate and the dried solution was filtrated. The filtrate was concentrated and the residue was purified by flash column chromatography on silica gel (25% diethyl ether in pentane) to give **21a** (1.39 g, 66%) as a yellowish oil.

TLC (20% diethyl ether in pentane): R_f = 0.18 (UV, CAM).
^{1}H–NMR (400 MHz, $CDCl_3$): δ = 5.52 (q, *J* = 7.1 Hz, 1H), 4.43 (dd, *J* = 8.5, 4.7 Hz, 1H), 4.19 – 4.12 (m, 2H), 3.81 – 3.70 (m, 2H), 3.49 (d, *J* = 19.2 Hz, 1H), 2.81 (dt, *J* = 8.6, 4.0 Hz, 1H), 1.80 – 1.62 (m, 2H), 1.63 (dt, *J* = 7.2, 1.6 Hz, 3H), 1.58 – 1.40 (m, 2H), 1.31 – 1.20 (m, 3H), 1.08 – 0.95 (m, 1H), 0.91 (s, 9H), 0.91 – 0.86 (s + s + t, 9H + 9H + 3H), 0.13 (s, 3H), 0.08 (s, 3H), 0.06 (s, 6H), 0.04 (s, 3H), –0.02 (s, 3H) ppm.
^{13}C–NMR (101 MHz, $CDCl_3$): δ = 199.5, 140.8, 118.2, 114.0, 74.5, 70.3, 62.6, 58.0, 34.6, 34.0, 30.5, 30.1, 26.2, 26.0, 25.9, 21.3, 18.7, 18.3, 18.2, 14.2, 13.0, –4.0, –4.5, –4.7, –4.9, –5.2 ppm.
IR (Diamond–ATR, neat): 2956, 2930, 2857, 1727, 1683, 1472, 1463, 1255, 836, 775 cm^{-1}.
HRMS (ESI) calc. for $C_{33}H_{67}NNaO_4Si_3^+$ $[M+Na]^+$: 648.4270; found: 648.4283.
$[\alpha]_D^{20}$: 57.7 (c = 0.35, CH_2Cl_2).

Alcohol 25a

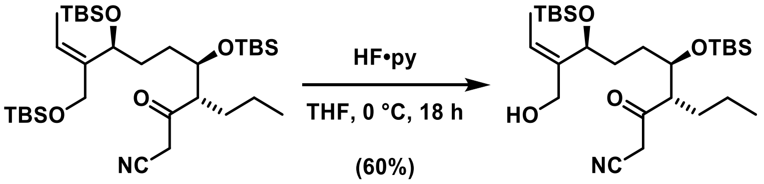

21a 25a

Pyridine hydrofluoride (≈70% hydrogen fluoride, ≈30% pyridine, 988 µL, 54.3 mmol, 25.0 equiv) was added to a solution of **21a** (1.36 g, 2.17 mmol, 1 equiv) and pyridine (8.80 mL, 109 mmol, 50.0 equiv) in tetrahydrofuran (18 mL) at 0 °C. After 18 h, aqueous saturated sodium hydrogen carbonate solution (50 mL) and dichloromethane (50 mL) were added to the reaction mixture. The layers were separated and the aqueous phase was extracted with dichloromethane (2 × 50 mL). The combined organic phases were dried over magnesium sulfate and the dried solution was filtrated. The filtrate was concentrated and the residue was purified by flash column chromatography on silica gel (60% diethyl ether in pentane) to give **25a** (837 mg, 60%) as a colorless oil.

TLC (60% diethyl ether in pentane): R_f = 0.21 (UV, CAM).

^{1}H–NMR (400 MHz, $CDCl_3$): δ = 5.53 (q, *J* = 7.0 Hz, 1H), 4.61 – 4.52 (m, 1H), 4.19 (dd, *J* = 12.6, 4.4 Hz, 1H), 4.08 (dd, *J* = 12.5, 7.2 Hz, 1H), 3.82 – 3.72 (m, 2H), 3.48 (d, *J* = 19.3 Hz, 1H), 2.83 (dt, *J* = 8.7, 4.1 Hz, 1H), 2.41 (dd, *J* = 7.2, 4.5 Hz, 1H), 1.78 – 1.49 (m, 7H), 1.33 – 1.15 (m, 3H), 1.08 – 0.95 (m, 1H), 0.92 – 0.88 (s + t + s, 9H + 3H +9H), 0.14 (s, 3H), 0.09 (s, 6H), 0.03 (s, 3H).

^{13}C–NMR (101 MHz, $CDCl_3$): δ = 200.0, 140.7, 123.3, 114.3, 74.6, 71.3, 65.8, 58.2, 35.0, 34.2, 30.7, 30.4, 26.3, 26.2, 21.5, 18.5 (2 carbons), 14.6, 13.5, –3.7, –4.2, –4.4, –4.5 ppm.

IR (Diamond–ATR, neat): 3441, 2956, 2930, 2858, 1726, 1472, 1256, 1070, 836, 776 cm^{-1}.

HRMS (ESI) calc. for $C_{27}H_{53}NNaO_4Si_2^+$ $[M+Na]^+$: 534.3405; found: 534.3401.

$[\alpha]_D^{20}$: 71.2 (c = 0.34, CH_2Cl_2).

Bromide 26a

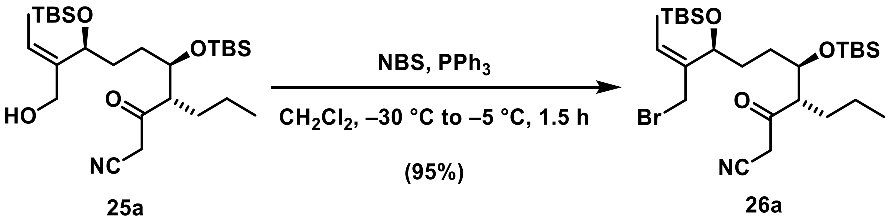

N-Bromosuccinimide (300 mg, 1.67 mmol, 1.30 equiv) was added to a solution of **25a** (833 mg, 1.29 mmol, 1 equiv) and triphenylphosphine (497 mg, 1.80 mmol, 1.40 equiv) in dichloromethane (15 mL) at –30 °C. The reaction mixture was allowed to slowly warm to –5 °C. After 1.5 h, water (30 mL) was added to the reaction mixture. The layers were separated and the aqueous phase was extracted with dichloromethane (2 × 20 mL). The combined organic phases were dried over magnesium sulfate and the dried solution was filtrated. The filtrate was concentrated and the residue was purified by flash column chromatography on silica gel (10% diethyl ether in pentane) to give **26a** (701 mg, 95%) as a slightly yellow oil.

TLC (10% diethyl ether in pentane): R_f = 0.41 (UV, CAM).
^{1}H–NMR (400 MHz, $CDCl_3$): δ = 5.80 (q, *J* = 7.1 Hz, 1H), 4.44 (t, *J* = 6.3 Hz, 1H), 4.20 (d, *J* = 9.9 Hz, 1H), 3.89 (d, *J* = 9.9 Hz, 1H), 3.84 – 3.73 (m, 2H), 3.50 (d, *J* = 19.3 Hz, 1H), 2.83 (dt, *J* = 8.6, 4.0 Hz, 1H), 1.79 – 1.59 (m, 7H), 1.33 – 1.18 (m, 3H), 1.14 – 1.01 (m, 1H), 0.92 – 0.87 (s + t + s, 9H + 3H + 9H), 0.14 (s, 3H), 0.11 (s, 3H), 0.05 (s, 3H), –0.04 (s, 3H).ppm.
^{13}C–NMR (101 MHz, $CDCl_3$): δ = 199.6, 139.4, 128.9, 114.0, 74.3, 70.4, 58.9, 34.6, 34.4, 33.2, 30.5, 30.1, 26.1, 25.9, 21.3, 18.3, 18.2, 14.3, 13.8, –4.0, –4.4, –4.7, –4.9 ppm.
IR (Diamond–ATR, neat): 2956, 2930, 2857, 1726, 1472, 1256, 1073, 862, 836, 776 cm^{-1}.
HRMS (ESI) calc. for $C_{27}H_{52}BrNNaO_3Si_2^+$ $[M+Na]^+$: 596.2561; found: 596.2556.
$[\alpha]_D^{20}$: 80.0 (c = 0.28, CH_2Cl_2).

Nine-membered carbocycle 27a

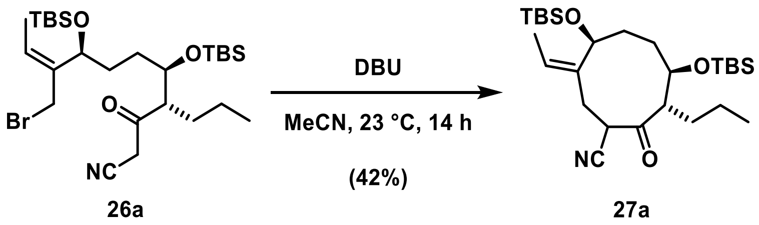

1,8-Diazabicyclo[5.4.0]-undec-7-ene (308 µL, 2.04 mmol, 2.00 equiv) was added to a solution of **26a** (587 mg, 1.02 mmol, 1 equiv) in acetonitrile (40 mL) at 23 °C. After 14 h, aqueous saturated ammonium chloride solution (80 mL) and dichloromethane (80 mL) were added to the reaction mixture. The layers were separated and the aqueous phase was extracted with dichloromethane (2 × 50 mL). The combined organic phases were dried over magnesium sulfate and the dried solution was filtrated. The filtrate was concentrated and the residue was purified by flash column chromatography on silica gel (5% diethyl ether in pentane) to give a diastereomeric mixture of **27a** (214 mg, 42%) as a yellowish oil.

TLC (10% diethyl ether in pentane): R_f = 0.61 (UV, CAM).

Triflate 28a

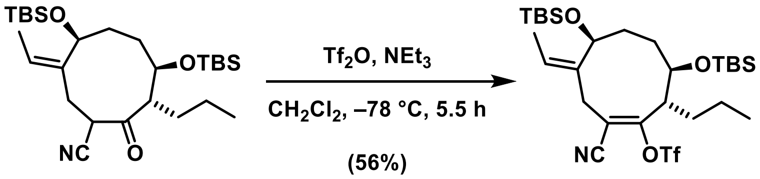

27a **28a**

Triethylamine (324 µL, 2.33 mmol, 5.00 equiv) was added dropwise to a solution of **27a** (230 mg, 466 µmol, 1 equiv) in dichloromethane (10 mL) at –78 °C. After 30 min, triflic anhydride (271 µL, 1.63 mmol, 3.50 equiv) was added dropwise. After 5.5 h, an aqueous saturated sodium hydrogen carbonate solution (20mL) was added to the reaction mixture. The layers were separated and the aqueous phase was extracted with dichloromethane (2 × 15 mL). The combined organic phases were dried over magnesium sulfate and the dried solution was filtrated. The filtrate was concentrated and the residue was purified by flash column chromatography on silica gel (5% diethyl ether in pentane) to give **28a** (163 mg, 56%) as a colorless oil, alongside with unreacted starting material **27a** (40.9 mg, 41%).

TLC (10% diethyl ether in pentane): R_f = 0.81 (UV, CAM).
^{1}H–NMR (400 MHz, $CDCl_3$): δ = 5.86 (q, *J* = 7.0 Hz, 1H), 4.78 (t, *J* = 5.9 Hz, 1H), 3.81 (dd, *J* = 10.2, 4.4 Hz, 1H), 3.47 (td, *J* = 10.9, 3.6 Hz, 1H), 3.35 (d, *J* = 14.7 Hz, 1H), 2.79 (d, *J* = 14.8 Hz, 1H), 1.94 – 1.67 (m, 4H), 1.65 (d, *J* = 7.7 Hz, 3H), 1.48 – 1.38 (m, 1H), 1.38 – 1.23 (m, 2H), 1.22 – 1.12 (m, 1H), 0.92 (t, *J* = 7.3 Hz, 3H), 0.91 (s, 9H), 0.88 (s, 9H), 0.08 (s, 3H), 0.06 (s, 6H), 0.03 (s, 3H) ppm.
^{13}C–NMR (101 MHz, $CDCl_3$): δ = 160.1, 136.6, 127.2, 118.5 (q, *J* = 320.6 Hz), 116.0, 111.4, 69.9, 69.7, 45.8, 30.2, 29.8, 28.8, 27.8, 26.0, 26.0, 20.8, 18.3, 18.3, 14.4, 13.6, –4.4, –4.6, –4.8, –4.9 ppm.
^{19}F–NMR (101 MHz, $CDCl_3$): δ = –72.6 ppm.
IR (Diamond–ATR, neat): 2956, 2930, 2858, 1410, 1255, 1214, 1134, 1062, 836, 776 cm^{-1}.
HRMS (ESI) calc. for $C_{28}H_{50}F_3NNaO_5SSi_2^+$ $[M+Na]^+$: 648.2793; found: 648.2798.
$[\alpha]_D^{20}$: 60.1 (c = 0.67, CH_2Cl_2).

Methyl Ester 29a

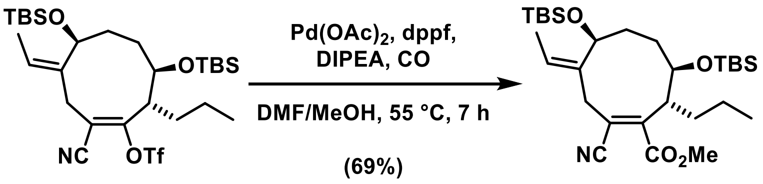

28a 29a

Note: Methanol and N,N-dimethylformamide was degassed via freeze-pump-thaw (three cycles) prior to use.

Palladium(II) acetate (15.4 mg, 68.7 µmol, 20.0 mol%), 1,1'-bis(diphenylphosphino)-ferrocene (38.1 mg, 68.7 µmol, 20.0 mol%) and *N,N*-diisopropylethylamine (60.0 µL, 343 µmol, 1.00 equiv) were sequentially added to a solution of **28a** (215 mg, 343 µmol, 1 equiv) in a mixture of *N*,*N*-dimethylformamide (7 mL) and methanol (1 mL). The flask was fitted with a balloon filled with carbon monoxide gas, and the solution was sparged with carbon monoxide for 10 minutes. The red-brownish reaction mixture was then heated to 55 °C under a carbon monoxide atmosphere. After 7 h, water (30 mL) and dichloromethane (30 mL) were added to the reaction mixture. The layers were separated, and the aqueous phase was extracted with dichloromethane (2 × 30 mL). The combined organic phases were dried over magnesium sulfate and the dried solution was filtrated. The filtrate was concentrated, and the residue was purified by flash column chromatography on silica gel (5% diethyl ether in pentane) to give **29a** (126 mg, 69%) as a colorless oil.

TLC (10% diethyl ether in pentane): R_f = 0.56 (UV, CAM).
^{1}H–NMR (400 MHz, $CDCl_3$): δ = 5.85 (q, *J* = 7.0 Hz, 1H), 4.73 (t, *J* = 6.0 Hz, 1H), 4.01 (dd, *J* = 9.9, 4.9 Hz, 1H), 3.83 (s, 3H), 3.46 (d, *J* = 14.1 Hz, 1H), 3.28 (td, *J* = 10.4, 3.3 Hz, 1H), 2.75 (d, *J* = 14.2 Hz, 1H), 1.97 – 1.87 (m, 1H), 1.84 – 1.75 (m, 1H), 1.76 – 1.58 (m, 5H), 1.36 – 1.25 (m, 1H), 1.22 – 1.08 (m, 3H), 0.91 (s, 9H), 0.88 (s, 9H), 0.86 (t, *J* = 7.1 Hz, 3H), 0.07 (s, 3H), 0.05 (s, 6H), 0.02 (s, 3H) ppm.
^{13}C–NMR (101 MHz, $CDCl_3$): δ = 167.1, 150.3, 137.1, 126.6, 119.2, 118.8, 71.6, 69.9, 52.5, 45.2, 31.8, 31.4, 28.6, 28.1, 26.1, 26.0, 21.3, 18.4, 18.3, 14.5, 13.6, –4.2, –4.5, –4.6, –4.9 ppm.
IR (Diamond–ATR, neat): 2953, 2931, 2858, 1726, 1472, 1279, 1254, 1211, 837, 780 cm^{-1}.
HRMS (ESI) calc. for $C_{29}H_{53}NNaO_4Si_2^+$ $[M+Na]^+$: 558.3405; found: 558.3403.
$[\alpha]_D^{20}$: 105.0 (c = 0.28, CH_2Cl_2).

Derivative 8

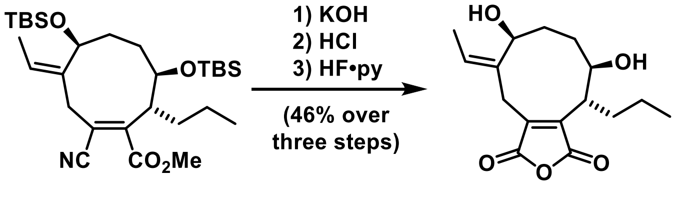

29a 8

A solution of potassium hydroxide (10wt.% in water, 1.25 mL) was added to a solution of **29a** (126 mg, 235 µmol, 1 equiv) in *iso*-propanol (12.5 mL) and the mixture was heated to 70 °C. After 2 h, the reaction mixture was allowed to cool to 23 °C and aqueous saturated ammonium chloride solution (35 mL) and dichloromethane (35 mL) were added. The layers were separated and the aqueous phase was extracted with dichloromethane (2 × 30 mL). The combined organic phases were dried over magnesium sulfate and the dried solution was filtrated. The filtrate was concentrated to give crude **S5**, which was used in the next step without further purification.

Aqueous hydrochloric acid (0.2 M, 1.2 mL) was added to a solution of crude **S5** (assumed 235 µmol, 1 equiv) in tetrahydrofuran (12 mL) at 23 °C. After 45 min, aqueous hydrochloric acid (0.2 M, 30 mL) and dichloromethane (30 mL) were added to the reaction mixture. The layers were separated and the aqueous phase was extracted with dichloromethane (2 × 30 mL). The combined organic phases were dried over magnesium sulfate and the dried solution was filtrated. The filtrate was concentrated to give crude anhydride **S6** which was used in the following deprotection step without further purification.

Pyridine hydrofluoride (≈70% hydrogen fluoride, ≈30% pyridine, 489 µL, 118.8 mmol, 80.0 equiv) was added to a solution of crude **S6** (assumed 235 µmol) in tetrahydrofuran (5 mL) at 0 °C and the reaction was allowed to warm to 23 °C. After 18 h, aqueous hydrochloric acid (0.2 M, 20 mL) and dichloromethane (20 mL) were added to the reaction mixture. The layers were separated and the aqueous phase was extracted with dichloromethane (2 × 20 mL). The combined organic phases were dried over magnesium sulfate and the dried solution was filtrated. The filtrate was concentrated and the residue was purified by flash column chromatography on silica gel (diethyl ether) to give **8** (32.0 mg, 46% over three steps) as a colorless oil.

TLC (diethyl ether): R_f = 0.23 (UV, CAM).

^{1}H–NMR (400 MHz, $CDCl_3$): δ = 5.94 (q, *J* = 7.3 Hz, 1H), 4.75 (dd, *J* = 9.5, 4.9 Hz, 1H), 3.90 (d, *J* = 9.5 Hz, 1H), 3.32 – 3.17 (m, 3H), 2.14 (dddd, *J* = 13.4, 11.3, 9.5, 4.8 Hz, 1H), 2.02 – 1.79 (m, 4H), 1.77 (br, 1H), 1.70 (d, *J* = 7.1 Hz, 3H), 1.61 (br, 1H), 1.38 – 1.07 (m, 3H), 0.92 (t, *J* = 7.3 Hz, 3H) ppm.

^{13}C–NMR (101 MHz, $CDCl_3$): δ = 165.8, 164.8, 146.3, 142.1, 135.3, 131.7, 71.3, 68.9, 41.4, 29.9, 28.7, 25.7, 25.0, 21.8, 14.1, 13.6 ppm.

IR (Diamond–ATR, neat): 3381, 2954, 2928, 2872, 1761, 1456, 1272, 1017, 926, 764 cm^{-1}.

HRMS (ESI) calc. for $C_{16}H_{22}NaO_5^+$ $[M+Na]^+$: 317.1359; found: 317.1359.

$[\alpha]_D^{20}$: 7.0 (c = 0.20, CH_2Cl_2).

Synthesis of derivative 9

Alcohol 18b

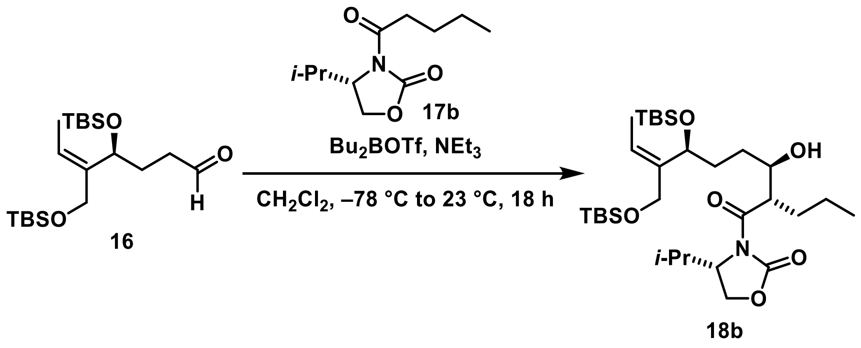

A solution of dibutylboryl trifluoromethanesulfonate (1 M in dichloromethane, 3.36 mL, 3.36 mmol, 1.30 equiv) was added to a solution of **17b** (607 mg, 2.85 mmol, 1.10 equiv) in dichloromethane (30 mL) at 0 °C. After 10 min, triethylamine (629 µL, 4.53 mmol, 1.75 equiv) was added to the orange solution. After 10 min, the solution was cooled to –78 °C and a solution of **16** (1.00 g, 2.59 mmol, 1 equiv) in dichloromethane (4 mL) was added to the reaction mixture. After complete addition, the reaction mixture was allowed to slowly warm to 23 °C. After 18 h, the reaction mixture was cooled to 0 °C and 0.6 mL of a 2:1 mixture of pH = 7 phosphate buffer/methanol and 0.6 mL of a 2:1 mixture of hydrogen peroxide (30% in water)/methanol were subsequently added. After 1 h, water (50 mL) was added and the layers were separated. The aqueous phase was extracted with dichloromethane (2 × 30 mL). The combined organic phases were dried over magnesium sulfate and the dried solution was filtrated. The filtrate was concentrated to give crude aldol product **18b**, which was clean enough to be used in the following step without further purification.

TLC (25% diethyl ether in pentane): R_f = 0.18 (UV, CAM).
^{1}H-NMR (400 MHz, $CDCl_3$): δ = 5.59 – 5.46 (m, 1H), 4.58 – 4.45 (m, 2H), 4.27 – 4.16 (m, 4H), 4.11 (dt, *J* = 9.8, 4.2 Hz, 1H), 3.79 (dt, *J* = 9.3, 3.7 Hz, 1H), 2.50 (d, *J* = 3.3 Hz, 1H), 2.37 (pd, *J* = 7.0, 3.7 Hz, 1H), 1.88 – 1.72 (m, 1H), 1.70 – 1.54 (m, 6H), 1.46 – 1.24 (m, 4H), 0.94 – 0.84 (m, 27H), 0.05 (s, 6H), 0.04 (s, 3H), –0.02 (s, 3H) ppm.
^{13}C-NMR (101 MHz, $CDCl_3$): δ = 176.1, 154.3, 140.9, 118.3, 73.0, 70.3, 63.0, 62.5, 58.8, 47.7, 33.7, 30.7, 29.9, 28.4, 26.2, 26.0, 20.9, 18.6, 18.3, 18.2, 14.6, 14.4, 13.1, –4.7, –4.9, –5.2 ppm.
IR (Diamond–ATR, neat): 3476, 2957, 2929, 2857, 1784, 1697, 1251, 1202, 1094, 836 cm^{-1}.
HRMS (ESI) calc. for $C_{31}H_{61}NNaO_6Si_2^+$ $[M+Na]^+$: 633.3930; found: 633.3922.
$[\alpha]_D^{20}$: 27.4 (c = 0.31, CH_2Cl_2).

Deoxygenation precursor S7

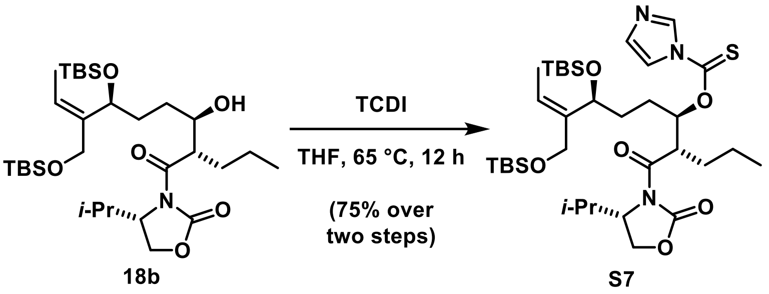

1,1'-Thiocarbonyldiimidazole (2.31 g, 12.9 mmol, 5.00 equiv) was added to a solution of crude aldol product **18b** (assumed 2.59 mmol, 1 equiv) in tetrahydrofuran (40 mL) and the resulting yellow suspension was heated to 65 °C. After 12 h, the reaction mixture was cooled to 23 °C, concentrated and the residue was purified by flash column chromatography on silica gel (40% diethyl ether in pentane) to give **S7** (1.38 g, 75% over two steps) as an amorphous colorless solid.

TLC (50% diethyl ether in pentane): R_f = 0.25 (UV, CAM).
^{1}H NMR (300 MHz, $CDCl_3$): δ = 8.29 (q, *J* = 1.3 Hz, 1H), 7.58 (t, *J* = 1.5 Hz, 1H), 7.03 (dd, *J* = 1.7, 0.9 Hz, 1H), 6.00 (dt, *J* = 8.7, 4.4 Hz, 1H), 5.59 – 5.48 (m, 1H), 4.50 (dd, *J* = 8.1, 5.2 Hz, 1H), 4.33 (tt, *J* = 7.7, 3.6 Hz, 2H), 4.28 – 4.09 (m, 4H), 2.32 (dtq, *J* = 9.2, 6.1, 3.1, 2.3 Hz, 1H), 2.04 – 1.15 (m, 11H), 0.97 – 0.82 (m, 27H), 0.03 (s, 6H), 0.03 (s, 3H), -0.03 (s, 3H).
^{13}C NMR (75 MHz, $CDCl_3$): δ = 184.5, 172.4, 154.1, 140.3, 137.1, 131.0, 119.4, 118.0, 83.7, 69.7, 63.5, 62.6, 59.0, 46.0, 32.7, 28.9, 28.6, 28.5, 26.1, 25.9, 20.7, 18.6, 18.3, 18.1, 14.8, 14.2, 13.2, –4.7, –4.9, –5.2 ppm.
IR (Diamond–ATR, neat): 2957, 2929, 2856, 1781, 1700, 1388, 1285, 1251, 1250, 1229, 886 cm^{-1}.
HRMS (ESI) calc. for $C_{35}H_{63}N_3NaO_6SSi_2^+$ $[M+Na]^+$: 732.3868; found: 732.3855.
$[\alpha]_D^{20}$: 25.1 (c = 0.37, CH_2Cl_2).

Deoxygenation product 19b

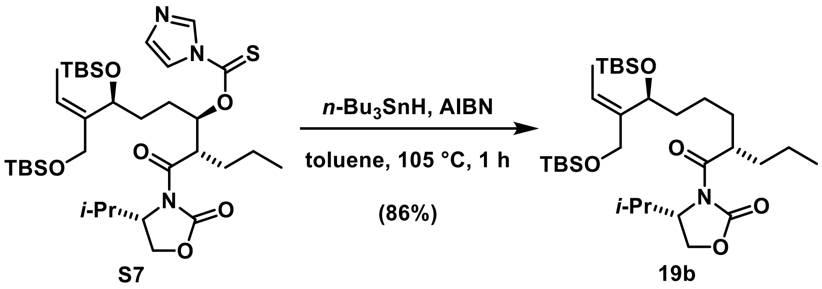

Azobisisobutyronitrile (80.9 mg, 493 µmmol, 0.500 equiv) was added to a solution of **S7** (700 mg, 986 µmol, 1 equiv) and tributyltin hydride (685 µL, 2.46 mmol, 2.50 equiv) in toluene (9 mL) and the resulting solution was heated to 105 °C. After 1 h, the reaction mixture was cooled to room temperature, evaporated and the residue was purified by flash column chromatography on silica gel (2% diethyl ether in pentane until tin impurities were removed, then 7% diethyl ether in pentane) to give **19b** (494 mg, 86%) as a colorless oil.

TLC (20% diethyl ether): R_f = 0.64 (UV, CAM).
^{1}H-NMR (400 MHz, $CDCl_3$): δ = 5.58 – 5.45 (m, 1H), 4.54 – 4.41 (m, 2H), 4.27 – 4.14 (m, 4H), 3.85 (tt, *J* = 8.2, 5.4 Hz, 1H), 2.36 (dtt, *J* = 10.7, 7.0, 3.8 Hz, 1H), 1.77 – 1.15 (m, 13H), 0.93 – 0.83 (m, 27H), 0.05 (s, 6H), 0.02 (s, 3H), –0.03 (s, 3H) ppm.
^{13}C-NMR (101 MHz, $CDCl_3$) δ = 177.1, 153.8, 140.9, 118.2, 70.0, 63.0, 62.5, 58.7, 42.6, 37.3, 35.2, 32.2, 28.5, 26.2, 26.0, 24.1, 20.4, 18.6, 18.3, 18.2, 14.7, 14.3, 13.1, –4.7, –4.9, –5.2 ppm.
IR (Diamond–ATR, neat): 2956, 2930, 2857, 1784, 1700, 1386, 1201, 1090, 836, 775 cm^{-1}.
HRMS (ESI) calc. for $C_{31}H_{61}NNaO_5Si_2^+$ $[M+Na]^+$: 606.3980; found: 606.3975.
$[\alpha]_D^{20}$: 27.4 (c = 0.34, CH_2Cl_2).

Thioester 20b

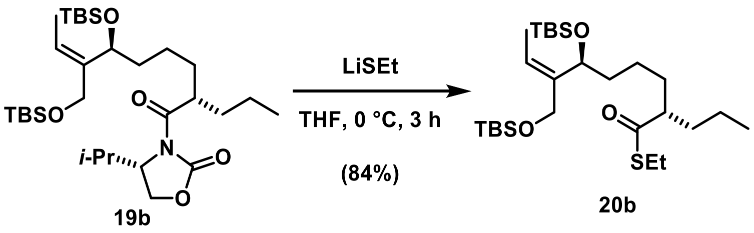

A solution of *n*-butyllithium (2.50 M in hexanes, 6.85 mL, 17.1 mmol, 4.00 equiv) was added dropwise to a solution of ethanethiol (1.60 mL, 21.4 mmol, 5.00 equiv) in tetrahydrofuran (75 mL) at –78 °C. After 5 min, the white suspension was allowed to warm to 0 °C. After 20 min, a solution of **19b** (2.49 g, 4.28 mmol, 1 equiv) in tetrahydrofuran (10 mL) was added to the reaction mixture. After 3 h, aqueous saturated ammonium chloride solution (150 mL) and dichloromethane (120 mL) were added to the slightly yellow reaction mixture. The layers were separated and the aqueous phase was extracted with dichloromethane (2 × 100 mL). The combined organic phases were dried over magnesium sulfate and the dried solution was filtrated. The filtrate was concentrated and the residue was purified by flash column chromatography on silica gel (5% diethyl ether in pentane) to give an **20b** (1.85 g, 84%) as a yellowish oil.

TLC (5% diethyl ether in pentane): R_f = 0.59 (UV, CAM).
^{1}H-NMR (400 MHz, $CDCl_3$): δ = 5.52 (qt, *J* = 7.1, 1.6 Hz, 1H), 4.49 (dd, *J* = 8.2, 4.8 Hz, 1H), 4.24 – 4.08 (m, 2H), 2.85 (q, *J* = 7.5 Hz, 2H), 2.51 (tt, *J* = 9.2, 4.6 Hz, 1H), 1.70 – 1.51 (m, 6H), 1.48 – 1.16 (m, 7H), 1.23 (t, *J* = 7.4 Hz, 3H), 0.94 – 0.84 (m, 21H), 0.05 (s, 6H), 0.02 (s, 3H), –0.03 (s, 3H) ppm.
^{13}C-NMR (101 MHz, $CDCl_3$): δ = 203.8, 141.0, 118.2, 70.0, 62.6, 54.5, 37.1, 35.4, 33.2, 26.2, 26.0, 24.1, 23.2, 20.6, 18.6, 18.3, 15.0, 14.2, 13.1, –4.7, –4.9, –5.2, –5.2 ppm.
IR (Diamond–ATR, neat): 2956, 2930, 2857, 1688, 1462, 1211, 1074, 1006, 836, 775 cm^{-1}.
HRMS (ESI) calc. for $C_{27}H_{56}NaO_3SSi_2^+$ $[M+Na]^+$: 539.3381; found: 539.3378.
$[\alpha]_D^{20}$: 6.0 (c = 0.33, CH_2Cl_2).

β–keto nitrile 21b

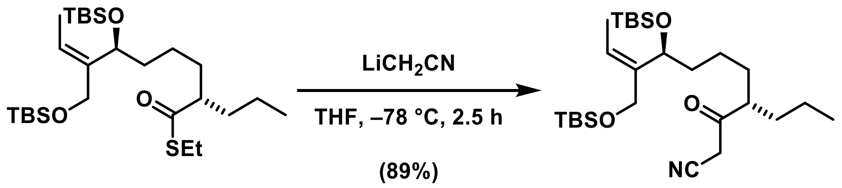

20b **21b**

A solution of *n*-butyllithium (2.50 M in hexanes, 4.32 mL, 10.8 mmol, 3.00 equiv) was added dropwise to a solution of acetonitrile (658 µL, 12.6 mmol, 3.50 equiv) in tetrahydrofuran (80 mL) at –78 °C. After 30 min, a solution of **20b** (1.86 g, 3.60 mmol, 1 equiv) in tetrahydrofuran (10 mL) was added to the reaction mixture. After 2.5 h, aqueous saturated ammonium chloride solution (150 mL) and dichloromethane (150 mL) were added to the yellow reaction mixture. The layers were separated and the aqueous phase was extracted with dichloromethane (2 × 100 mL). The combined organic phases were dried over magnesium sulfate and the dried solution was filtrated. The filtrate was concentrated and the residue was purified by flash column chromatography on silica gel (25% diethyl ether in pentane) to give **21b** (1.59 g, 89%) as a yellowish oil.

TLC (10% diethyl ether in pentane): R_f = 0.19 (UV, CAM).
^{1}H-NMR (400 MHz, $CDCl_3$) δ = 5.51 (s, 1H), 4.50 (dd, *J* = 8.0, 5.5 Hz, 1H), 4.16 (dtt, *J* = 15.6, 14.0, 1.8 Hz, 2H), 3.44 (s, 2H), 2.65 (tq, *J* = 7.9, 5.3 Hz, 1H), 1.68 – 1.11 (m, 13H), 0.94 – 0.85 (m, 21H), 0.06 (s, 6H), 0.03 (s, 3H), –0.02 (s, 3H) ppm.
^{13}C-NMR (101 MHz, $CDCl_3$) δ = 201.2, 140.8, 119.0, 113.8, 69.8, 62.7, 51.7, 37.1, 33.6, 31.7, 31.4, 26.2, 25.9, 23.9, 20.6, 18.6, 18.3, 14.2, 13.2, –4.7, –4.9, –5.2, –5.2 ppm.
IR (Diamond–ATR, neat): 2955, 2930, 2857, 1727, 1463, 1361, 1254, 1005, 836, 775 cm^{-1}.
HRMS (ESI) calc. for $C_{27}H_{53}NNaO_3Si_2^+$ $[M+Na]^+$: 518.3456; found: 518.3454.
$[\alpha]_D^{20}$: –1.12 (c = 0.27, CH_2Cl_2).

Alcohol 25b

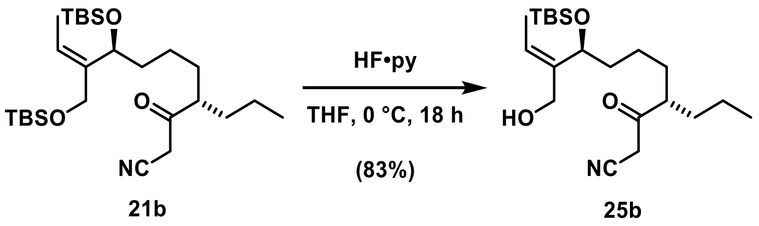

Pyridine hydrofluoride (≈70% hydrogen fluoride, ≈30% pyridine, 1.46 mL, 80.2 mmol, 25.0 equiv) was added to a solution of **21b** (1.59 g, 3.21 mmol, 1 equiv) and pyridine (13.0 mL, 160 mmol, 50.0 equiv) in tetrahydrofuran (26 mL) at 0 °C. After 18 h, aqueous saturated sodium hydrogen carbonate solution (50 mL) and dichloromethane (60 mL) were added to the reaction mixture. The layers were separated and the aqueous phase was extracted with dichloromethane (2 × 50 mL). The combined organic phases were dried over magnesium sulfate and the dried solution was filtrated. The filtrate was concentrated and the residue was purified by flash column chromatography on silica gel (60% diethyl ether in pentane) to give **25b** (1.02 g, 83%) as a colorless oil.

TLC (50% diethyl ether in pentane): R_f = 0.22 (UV, CAM).
^{1}H-NMR (400 MHz, $CDCl_3$): δ = 5.54 (dddd, *J* = 8.0, 7.0, 5.9, 1.0 Hz, 1H), 4.61 (t, *J* = 7.0 Hz, 1H), 4.21 (d, *J* = 12.7 Hz, 1H), 4.04 (d, *J* = 12.6 Hz, 1H), 3.45 (s, 2H), 2.66 (tt, *J* = 7.9, 5.6 Hz, 1H), 2.50 (s, 1H), 1.72 – 1.05 (m, 13H), 0.93 – 0.85 (t + s, 3H + 9H), 0.07 (s, 3H), 0.03 (s, 3H) ppm.
^{13}C-NMR (101 MHz, $CDCl_3$): δ = 201.1, 140.1, 123.2, 113.8, 70.6, 65.4, 51.5, 36.9, 33.6, 31.6, 31.2, 25.9, 23.5, 20.6, 18.2, 14.2, 13.3, –4.7, –4.8 ppm.
IR (Diamond–ATR, neat): 3446, 2956, 2931, 2858, 1725, 1463, 1253, 1069, 836, 776 cm^{-1}.
HRMS (ESI) calc. for $C_{21}H_{39}NNaO_3Si^+$ $[M+Na]^+$: 404.2591; found: 404.2586.
$[\alpha]_D^{20}$: –1.91 (c = 0.31, CH_2Cl_2).

Bromide 26b

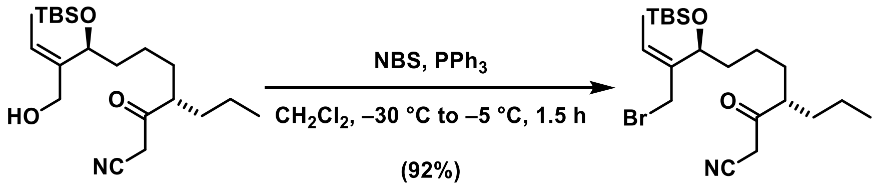

25b 26b

N-Bromosuccinimide (625 mg, 3.47 mmol, 1.30 equiv) was added to a solution of **25b** (1.02 g, 2.67 mmol, 1 equiv) and triphenylphosphine (1.03 g, 3.74 mmol, 1.40 equiv) in dichloromethane (30 mL) at –30 °C. The reaction mixture was allowed to slowly warm to –5 °C. After 1.5 h, water (50 mL) was added to the reaction mixture. The layers were separated and the aqueous phase was extracted with dichloromethane (2 × 30 mL). The combined organic phases were dried over magnesium sulfate and the dried solution was filtrated. The filtrate was concentrated and the residue was purified by flash column chromatography on silica gel (10% diethyl ether in pentane) to give **36b** (1.09 g, 92%) as a slightly yellow oil.

TLC (10% diethyl ether in pentane): R_f = 0.22 (UV, CAM).
^{1}H-NMR (400 MHz, $CDCl_3$): δ = 5.82 (q, *J* = 7.1 Hz, 1H), 4.50 (dd, *J* = 7.7, 6.3 Hz, 1H), 4.19 (d, *J* = 10.0 Hz, 1H), 3.90 (d, *J* = 10.0 Hz, 1H), 3.50 – 3.43 (m, 2H), 2.67 (tt, *J* = 7.9, 5.6 Hz, 1H), 1.79 – 1.16 (m, 13H), 0.91 (td, *J* = 7.3, 1.3 Hz, 3H), 0.87 (s, 9H), 0.05 (s, 3H), –0.03 (s, 3H) ppm.
^{13}C-NMR (101 MHz, $CDCl_3$): δ = 201.1, 138.9, 129.2, 113.8, 69.9, 51.6, 37.2, 33.5, 33.3, 31.6, 31.1, 25.9, 23.7, 20.6, 18.2, 14.2, 13.8, –4.7, –4.9 ppm.
IR (Diamond–ATR, neat): 2955, 2930, 2857, 1726, 1463, 1251, 1210, 1073, 837, 776 cm^{-1}.
HRMS (ESI) calc. for $C_{21}H_{38}BrNNaO_3Si^+$ $[M+Na]^+$: 466.1747; found: 466.1744.
$[\alpha]_D^{20}$: 29.0 (c = 0.29, CH_2Cl_2).

Nine-membered carbocycle 27b

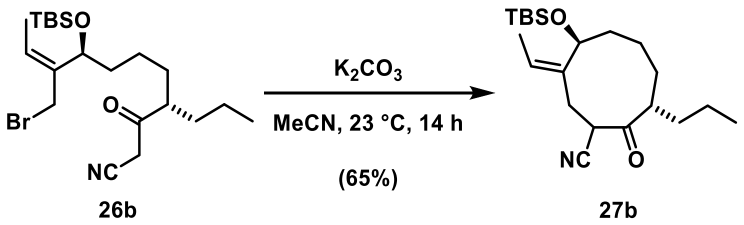

Potassium carbonate (847 mg, 6.13 mmol, 2.50 equiv) was added to a solution of **26b** (1.09 g, 2.45 mmol, 1 equiv) in acetonitrile (84 mL) at 23 °C. After 14 h, aqueous saturated ammonium chloride solution (150 mL) and dichloromethane (150 mL) were added to the reaction mixture. The layers were separated and the aqueous phase was extracted with dichloromethane (2 × 100 mL). The combined organic phases were dried over magnesium sulfate and the dried solution was filtrated. The filtrate was concentrated and the residue was purified by flash column chromatography on silica gel (8% diethyl ether in pentane) to give a diastereomeric mixture of **27b** (579 mg, 65%) as a yellowish oil.

TLC (10% diethyl ether in pentane): R_f = 0.45 (UV, CAM).

Triflate 28b

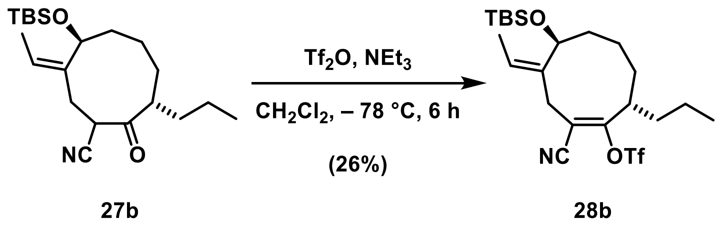

Triethylamine (695 µL, 4.98 mmol, 5.00 equiv) was added dropwise to a solution of **27b** (362 mg, 996 µmol, 1 equiv) in dichloromethane (34 mL) at –78 °C. After 30 min, triflic anhydride (590 µL, 3.48 mmol, 3.50 equiv) was added dropwise. After 6 h, an aqueous saturated sodium hydrogen carbonate solution (50mL) was added to the reaction mixture. The layers were separated and the aqueous phase was extracted with dichloromethane (2 × 40 mL). The combined organic phases were dried over magnesium sulfate and the dried solution was filtrated. The filtrate was concentrated and the residue was purified by flash column chromatography on silica gel (5% diethyl ether in pentane) to give **28b** (126 mg, 26%) as a colorless oil, alongside with unreacted starting material **27b** (223 mg, 62%).

An overall yield of 51% was achieved, when unreacted starting material was resubjected to the reaction condition for two additional times.

TLC (10% diethyl ether in pentane): R_f = 0.67 (UV, CAM).
^{1}H NMR (400 MHz, $CDCl_3$): δ = 5.78 (qt, *J* = 7.1, 1.3 Hz, 1H), 4.82 (ddd, *J* = 5.7f, 4.1, 1.4 Hz, 1H), 3.64 (td, *J* = 10.9, 4.6 Hz, 1H), 3.41 (d, *J* = 14.5 Hz, 1H), 2.74 (dt, *J* = 14.6, 1.2 Hz, 1H), 2.01 – 1.90 (m, 1H), 1.65 – 1.02 (m, 12H), 0.94 – 0.84 (m, 12H), 0.08 (s, 3H), 0.06 (s, 3H) ppm.
^{13}C-NMR (101 MHz, $CDCl_3$): δ = 161.9, 137.2, 126.5, 118.5 (q, *J* = 320.5 Hz), 116.0, 110.1, 70.4, 39.2, 35.3, 33.9, 31.4, 31.1, 25.9, 21.1, 20.9, 18.3, 14.3, 13.6, –4.7, –5.0 ppm.
^{19}F–NMR (101 MHz, $CDCl_3$): δ = –72.6 ppm.
IR (Diamond–ATR, neat): 2956, 2931, 2860, 1410, 1214, 1135, 1069, 911, 837, 777 cm^{-1}.
HRMS (ESI) calc. for $C_{22}H_{36}F_3NNaO_4SSi^+$ $[M+Na]^+$: 518.1979; found: 518.1974.
$[\alpha]_D^{20}$: 91.1 (c = 0.28, CH_2Cl_2).

Methyl Ester 29b

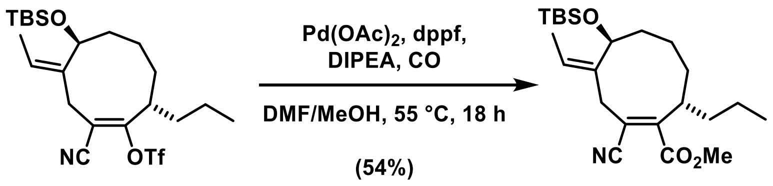

28b 29b

Note: Methanol and N,N-dimethylformamide was degassed via freeze-pump-thaw (three cycles) prior to use.

Palladium(II) acetate (18.6 mg, 82.7 µmol, 20.0 mol%), 1,1'-bis(diphenylphosphino)-ferrocene (45.9 mg, 82.7 µmol, 20.0 mol%) and *N,N*-diisopropylethylamine (72.3 µL, 414 µmol, 1.00 equiv) were sequentially added to a solution of **28b** (205 mg, 414 µmol, 1 equiv) in a mixture of *N*,*N*-dimethylformamide (9 mL) and methanol (1.5 mL). The flask was fitted with a balloon filled with carbon monoxide gas, and the solution was sparged with carbon monoxide for 10 minutes. The red-brownish reaction mixture was then heated to 55 °C under a carbon monoxide atmosphere. After 18 h, water (40 mL) and dichloromethane (30 mL) were added to the reaction mixture. The layers were separated, and the aqueous phase was extracted with dichloromethane (2 × 30 mL). The combined organic phases were dried over magnesium sulfate and the dried solution was filtrated. The filtrate was concentrated, and the residue was purified by flash column chromatography on silica gel (5% diethyl ether in pentane) to give **29b** (90 mg, 54%) as a colorless oil.

TLC (10% diethyl ether in pentane): R_f = 0.43 (UV, CAM).
^{1}H-NMR (400 MHz, $CDCl_3$): δ = 5.76 (qt, *J* = 7.0, 1.3 Hz, 1H), 4.85 – 4.75 (m, 1H), 3.82 (s, 3H), 3.54 – 3.42 (m, 2H), 2.69 (dt, *J* = 13.9, 1.2 Hz, 1H), 1.94 – 1.84 (m, 1H), 1.77 – 1.65 (m, 1H), 1.59 (dd, *J* = 7.1, 0.9 Hz, 3H), 1.57 – 1.17 (m, 7H), 1.06 – 0.96 (m, 1H), 0.91 – 0.83 (m, 12H), 0.07 (s, 3H), 0.03 (s, 3H) ppm.
^{13}C-NMR (101 MHz, $CDCl_3$): δ = 166.9, 152.0, 137.9, 125.5, 119.2, 117.8, 70.6, 52.3, 38.9, 36.0, 35.5, 33.4, 31.9, 25.9, 21.4, 21.3, 18.3, 14.5, 13.6, –4.7, –5.0 ppm.
IR (Diamond–ATR, neat): 2955, 2930, 2857, 1731, 1420, 1209, 1070, 835, 777, 743 cm^{-1}.
HRMS (ESI) calc. for $C_{23}H_{39}NNaO_3Si^+$ $[M+Na]^+$: 428.2591; found: 428.2589.
$[\alpha]_D^{20}$: 97.5 (c = 0.16, CH_2Cl_2).

Derivative 9

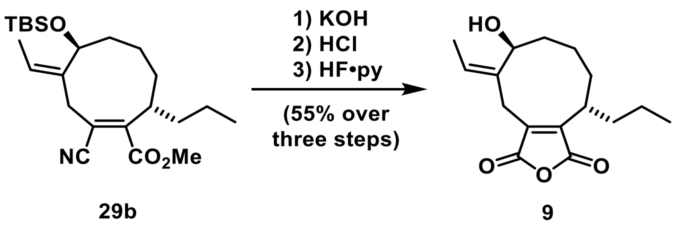

A solution of potassium hydroxide (10wt.% in water, 0.9 mL) was added to a solution of **29b** (90.1 mg, 222 µmol, 1 equiv) in *iso*-propanol (9 mL) and the mixture was heated to 70 °C. After 2.5 h, the reaction mixture was allowed to cool to 23 °C and aqueous saturated ammonium chloride solution (25 mL) and dichloromethane (30 mL) were added. The layers were separated and the aqueous phase was extracted with dichloromethane (2 × 20 mL). The combined organic phases were dried over magnesium sulfate and the dried solution was filtrated. The filtrate was concentrated to give crude **S8** which was used in the next step without further purification.

Aqueous hydrochloric acid (0.2 M, 0.9 mL) was added to a solution of crude **S8** (assumed 222 µmol, 1 equiv) in tetrahydrofuran (9 mL) at 23 °C. After 45 min, aqueous hydrochloric acid (0.2 M, 20 mL) and dichloromethane (20 mL) were added to the reaction mixture. The layers were separated and the aqueous phase was extracted with dichloromethane (2 × 20 mL). The combined organic phases were dried over magnesium sulfate and the dried solution was filtrated. The filtrate was concentrated to give crude anhydride **S9** which was used in the following deprotection step without further purification.

Pyridine hydrofluoride (≈70% hydrogen fluoride, ≈30% pyridine, 404 µL, 15.5 mmol, 70.0 equiv) was added to a solution of crude **S9** (assumed 0.222 mmol) in tetrahydrofuran (5 mL) at 0 °C and the reaction was allowed to warm to 23 °C. After 18 h, aqueous hydrochloric acid (0.2 M, 20 mL) and dichloromethane (20 mL) were added to the reaction mixture. The layers were separated and the aqueous phase was extracted with dichloromethane (2 × 20 mL). The combined organic phases were dried over magnesium sulfate and the dried solution was filtrated. The filtrate was concentrated and the residue was purified by flash column chromatography on silica gel (60% diethyl ether in pentane) to give **9** (34.1 mg, 55% over three steps) as a colorless oil.

TLC (60% diethyl ether in pentane): R_f = 0.24 (UV, CAM).

^{1}H-NMR (400 MHz, $CDCl_3$): δ = 5.98 (q, *J* = 7.1 Hz, 1H), 4.69 (dd, *J* = 10.1, 5.6 Hz, 1H), 3.20 (s, 2H), 3.17 – 3.08 (m, 1H), 2.07 (dtd, *J* = 13.5, 8.9, 6.4 Hz, 1H), 1.99 – 1.85 (m, 1H), 1.74 – 1.17 (m, 11H), 0.91 (t, *J* = 7.3 Hz, 3H), 0.59 (dddd, *J* = 15.1, 8.1, 5.9, 3.6 Hz, 1H) ppm.

^{13}C-NMR (101 MHz, $CDCl_3$): δ = 166.1, 164.7, 145.9, 143.7, 136.5, 132.0, 69.0, 34.5, 33.6, 31.4, 29.5, 24.4, 21.7, 20.3, 14.1, 13.4 ppm.

IR (Diamond–ATR, neat): 3380, 2928, 2872, 1759, 1466, 1218, 1016, 920, 886, 764 cm^{-1}.

HRMS (ESI) calc. for $C_{16}H_{22}NaO_4^+$ $[M+Na]^+$: 301.1410; found: 301.1405.

$[\alpha]_D^{20}$: 18.3 (c = 0.63, CH_2Cl_2).

Synthesis of derivative 10

Methyl Ester 23

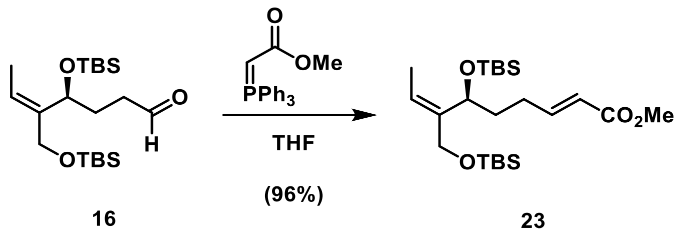

16 23

To a solution of aldehyde **16** (910 mg, 2.35 mmol, 1 eq) in dry THF (20 mL) at 23 °C was added (methoxycarbonylmethylene)triphenylphosphorane (1.60 g, 4.71 mmol, 2.00 eq) in one portion. The reaction mixture was heated to 60 °C for 12 h. After cooling down, the reaction mixture was concentrated under reduced pressure and the crude product was purified by flash column chromatography on silica gel (3% to 6% ethyl acetate in petroleum ether) to give methyl ester **23** (963 mg, 92%) as a colorless oil.

TLC (10% diethyl ether in pentane): R_f = 0.50 (UV, CAM).

^{1}H-NMR (400 MHz, Chloroform-*d*): δ = 6.98 (dt, *J* = 15.7, 6.8 Hz, 1H), 5.82 (dt, *J* = 15.7, 1.6 Hz, 1H), 5.61 – 5.51 (m, 1H), 4.54 (dd, *J* = 8.1, 5.6 Hz, 1H), 4.16 (qt, *J* = 13.9, 1.7 Hz, 2H), 3.72 (s, 3H), 2.34 – 2.09 (m, 2H), 1.85 – 1.72 (m, 1H), 1.65 (dt, *J* = 7.1, 1.7 Hz, 3H), 1.63 – 1.57 (m, 1H), 0.91 (s, 9H), 0.88 (s, 9H), 0.06 (s, 6H), 0.04 (s, 3H), –0.01 (s, 3H) ppm.

^{13}C-NMR (101 MHz, Chloroform-*d*): δ = 167.4, 149.6, 140.6, 121.1, 119.6, 69.6, 62.9, 51.6, 35.5, 29.1, 26.2, 26.0, 18.7, 18.4, 13.3, –4.6, –4.8, –5.1, –5.1 ppm.

IR (Diamond–ATR, neat): 2954, 2929, 2857, 1729, 1659, 1472, 1254, 1073, 836, 775 cm^{-1}.

HRMS (ESI) calc. for $C_{23}H_{46}NNaO_4Si_2^+$ $[M+Na]^+$: 465.2827; found: 465.2823.

$[\alpha]_D^{20}$: –10.3 (c = 0.30, CH_2Cl_2).

Methyl Ester 24

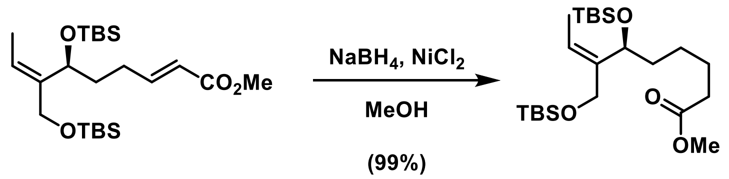

23 24

To a solution of methyl ester **23** (963 mg, 2.17 mmol, 1 eq) in MeOH (22 mL) was added $NiCl_2$ (39.4 mg, 0.30 mmol, 0.14 eq), followed by $NaBH_4$ (239 mg, 6.30 mmol, 2.90 eq) portion-wise at 0 °C. After 1.5 h, aqueous saturated ammonium chloride solution (15 mL) and ethyl acetate (30 mL) were added to the reaction mixture. The layers were separated and the aqueous phase was extracted with ethyl acetate (2 × 20 mL). The combined organic phases were dried over magnesium sulfate and the dried solution was filtrated. The filtrate was concentrated to give crude **24** as a white cloudy oil (960 mg, 99%) which was used in the next step without further purification.

TLC (3% ethyl acetate in petroleum ether): R_f = 0.35 (UV, CAM).
^{1}H-NMR (400 MHz, Chloroform-*d*): δ = 5.57 – 5.48 (m, 1H), 4.52 (dd, *J* = 7.9, 5.4 Hz, 1H), 4.23 – 4.11 (m, 2H), 3.65 (s, 3H), 2.29 (t, *J* = 7.6 Hz, 2H), 1.69 – 1.55 (m, 6H), 1.50 – 1.33 (m, 2H), 1.30 – 1.18 (m, 1H), 0.91 (s, 9H), 0.87 (s, 9H), 0.05 (s, 6H), 0.03 (s, 3H), –0.02 (s, 3H) ppm.
^{13}C-NMR (101 MHz, $CDCl_3$): δ = 174.3, 140.9, 118.3, 69.9, 62.6, 51.6, 36.8, 34.3, 26.2, 25.9, 25.7, 25.1, 18.6, 18.3, 13.1, –4.7, –4.9, –5.2, –5.2 ppm.
IR (Diamond–ATR, neat): 2953, 2930, 2857, 1743, 1463, 1361, 1253, 1091, 836, 775 cm^{-1}.
HRMS (ESI) calc. for $C_{23}H_{48}NaO_4Si_2^+$ $[M+Na]^+$: 467.2983; found: 467.2983.
$[\alpha]_D^{20}$: –3.03 (c = 0.35, CH_2Cl_2).

β–keto nitrile 21c

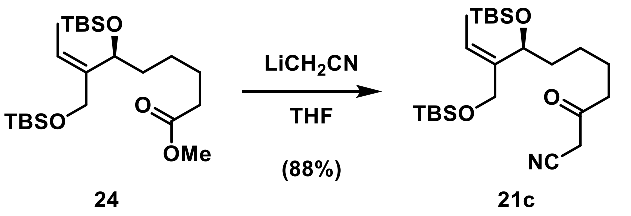

A solution of *n*-butyllithium (2.50 M in hexanes, 3.13 mL, 7.82 mmol, 3.60 equiv) was added dropwise to a solution of acetonitrile (451 µL, 8.69 mmol, 4.00 equiv) in tetrahydrofuran (48 mL) at –78 °C. After 45 min, a solution of **24** (936 mg, 2.17 mmol, 1 equiv) in tetrahydrofuran (4 mL) was added to the reaction mixture. After 1 h, aqueous saturated ammonium chloride solution (50 mL) and ethyl acetate (75 mL) were added to the yellow reaction mixture. The layers were separated and the aqueous phase was extracted with ethyl acetate (2 × 50 mL). The combined organic phases were dried over magnesium sulfate and the dried solution was filtrated. The filtrate was concentrated and the residue was purified by flash column chromatography on silica gel (9% to 14% ethyl acetate in petroleum ether) to give **21c** (845 mg, 88%) as a yellowish oil.

TLC (9% ethyl acetate in petroleum ether): R_f = 0.16 (UV, CAM).
^{1}H-NMR (400 MHz, Chloroform-*d*): δ = 5.58 – 5.48 (m, 1H), 4.51 (dd, *J* = 7.9, 5.5 Hz, 1H), 4.23 – 4.08 (m, 2H), 3.43 (s, 2H), 2.60 (t, *J* = 7.4 Hz, 2H), 1.68 – 1.57 (m, 6H), 1.52 – 1.31 (m, 2H), 1.32 – 1.18 (m, 1H), 0.91 (s, 9H), 0.87 (s, 9H), 0.06 (s, 6H), 0.03 (s, 3H), –0.02 (s, 3H) ppm.
^{13}C-NMR (101 MHz, Chloroform-*d*): δ = 197.5, 140.8, 118.7, 113.9, 69.8, 62.7, 42.3, 36.8, 32.1, 26.1, 25.9, 25.5, 23.5, 18.6, 18.3, 13.1, –4.7, –4.9, –5.2, –5.2 ppm.
IR (Diamond–ATR, neat): 2953, 2929, 2857, 1733, 1472, 1361, 1253, 1092, 836, 775 cm^{-1}.
HRMS (ESI) calc. for $C_{24}H_{47}NNaO_3Si_2^+$ $[M+Na]^+$: 476.2987; found: 476.2986.
$[\alpha]_D^{20}$: –4.69 (c = 0.32, CH_2Cl_2).

Alcohol 25c

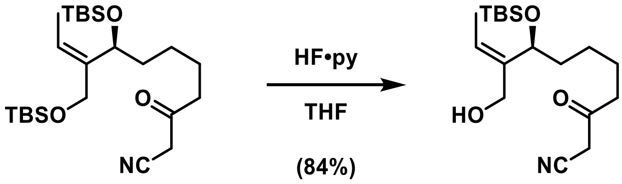

21c 25c

Pyridine hydrofluoride (≈70% hydrogen fluoride, ≈30% pyridine, 1.40 mL, 11.2 mmol, 6.00 equiv) was added to a solution of **21c** (847 mg, 1.87 mmol, 1 equiv) and pyridine (7.56 mL, 93.32 mmol, 50.0 equiv) in tetrahydrofuran (12 mL) at 0 °C. After 5 h, aqueous saturated sodium hydrogen carbonate solution (20 mL) and ethyl acetate (30 mL) were added to the reaction mixture. The layers were separated and the aqueous phase was extracted with ethyl acetate (2 × 30 mL). The combined organic phases were dried over magnesium sulfate and the dried solution was filtrated. The filtrate was concentrated and the residue was purified by flash column chromatography on silica gel (25% to 33% ethyl acetate in petroleum ether) to give **25c** (530 mg, 84%) as a colorless oil.

TLC (25% ethyl acetate in petroleum ether): R_f = 0.13 (UV, CAM).
^{1}H-NMR (400 MHz, Chloroform-*d*): δ = 5.55 (qd, *J* = 7.0, 1.1 Hz, 1H), 4.64 (t, *J* = 7.0 Hz, 1H), 4.25 (dt, *J* = 12.6, 1.2 Hz, 1H), 4.04 (d, *J* = 12.5 Hz, 1H), 3.43 (s, 2H), 2.61 (t, *J* = 7.3 Hz, 2H), 2.55 – 2.24 (bs, 1H), 1.76 – 1.52 (m, 7H), 1.41 – 1.27 (m, 1H), 1.26 – 1.12 (m, 1H), 0.88 (s, 9H), 0.08 (s, 3H), 0.04 (s, 3H) ppm.
^{13}C-NMR (101 MHz, Chloroform-*d*): δ = 197.4, 140.2, 123.2, 113.9, 70.7, 65.5, 42.1, 36.6, 32.1, 25.9, 25.1, 23.4, 18.2, 13.2, –4.7, –4.8 ppm.
IR (Diamond–ATR, neat): 3448, 2929, 2857, 1731, 1667, 1463, 1253, 1065, 836, 776 cm^{-1}.
HRMS (ESI) calc. for $C_{18}H_{33}NNaO_3Si^+$ $[M+Na]^+$: 362.2122; found: 362.2120.
$[\alpha]_D^{20}$: –5.80 (c = 0.29, CH_2Cl_2).

Bromide 26c

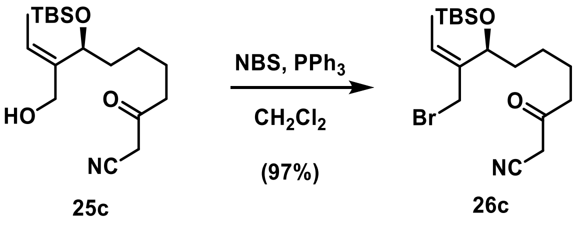

N-Bromosuccinimide (546 mg, 3.07 mmol, 1.30 equiv) was added to a solution of **25c** (801 mg, 2.36 mmol, 1 equiv) and triphenylphosphine (866 mg, 3.30 mmol, 1.40 equiv) in dichloromethane (25 mL) at –30 °C. The reaction mixture was allowed to slowly warm to –5 °C. After 2 h, water (30 mL) was added to the reaction mixture. The layers were separated and the aqueous phase was extracted with dichloromethane (2 × 30 mL). The combined organic phases were dried over magnesium sulfate and the dried solution was filtrated. The filtrate was concentrated and the residue was purified by flash column chromatography on silica gel (15% ethyl acetate in petroleum ether) to give **26c** (920 mg, 97%) as a slightly yellow oil.

TLC (25% ethyl acetate in petroleum ether): R_f = 0.23 (UV, CAM).
^{1}H-NMR (400 MHz, Chloroform-*d*): δ = 5.82 (q, *J* = 7.1 Hz, 1H), 4.52 (t, *J* = 6.9 Hz, 1H), 4.19 (d, *J* = 10.0, 1H), 3.90 (d, *J* = 10.0 Hz, 1H), 3.44 (s, 2H), 2.62 (t, *J* = 7.3 Hz, 2H), 1.81 – 1.72 (m, 1H), 1.69 (d, *J* = 7.2 Hz, 3H), 1.68 – 1.59 (m, 3H), 1.47 – 1.19 (m, 2H), 0.87 (s, 9H), 0.04 (s, 3H), –0.03 (s, 3H) ppm.
^{13}C-NMR (101 MHz, Chloroform-*d*): δ = 197.5, 139.0, 129.0, 113.9, 70.0, 42.2, 36.9, 33.3, 32.1, 25.9, 25.3, 23.3, 18.2, 13.8, –4.7, –4.9 ppm.
IR (Diamond–ATR, neat): 2952, 2930, 2857, 1732, 1472, 1404, 1253, 1073, 837, 776 cm^{-1}.
HRMS (ESI) calc. for $C_{18}H_{32}BrNNaO_2Si^+$ $[M+Na]^+$: 424.1278; found: 424.1287.
$[\alpha]_D^{20}$: 33.41 (c = 0.29, CH_2Cl_2).

Nine-membered carbocycle 27c

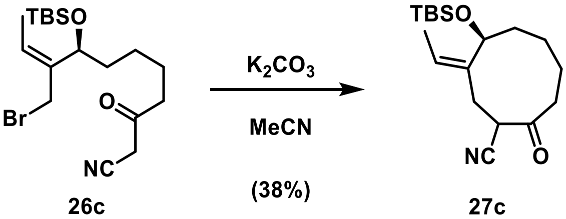

26c 27c

Potassium carbonate (1.26 g, 9.14 mmol, 4.00 equiv) was added to a solution of **26c** (920 mg, 2.28 mmol, 1 equiv) in acetonitrile (80 mL) at 23 °C. After 48 h, aqueous saturated ammonium chloride solution (150 mL) and dichloromethane (150 mL) were added to the reaction mixture. The layers were separated and the aqueous phase was extracted with dichloromethane (2 × 100 mL). The combined organic extracts were dried over magnesium sulfate and the dried solution was filtrated. The filtrate was concentrated and the residue was purified by flash column chromatography on silica gel (5% ethyl acetate in petroleum ether) to give a diastereomeric mixture of **27c** (277 mg, 38%) as a colorless oil.

TLC (9% ethyl acetate in petroleum ether): R_f = 0.15 (UV, CAM).
IR (Diamond–ATR, neat): 2953, 2930, 2856, 1719, 1447, 1251, 1065, 878, 836, 776 cm^{-1}.
HRMS (ESI) calc. for $C_{18}H_{31}NNaO_2Si^+$ $[M+Na]^+$: 344.2016; found: 344.2014.

Triflate 28c

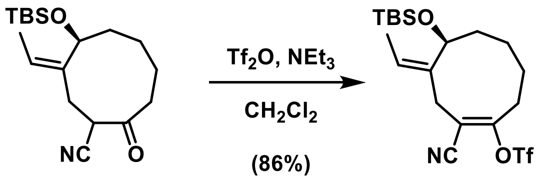

27c **28c**

Triethylamine (600 µL, 4.31 mmol, 5.00 equiv) was added dropwise to a solution of **27c** (277 mg, 861 µmol, 1 equiv) in dichloromethane (40 mL) at –78 °C. After 30 min, a solution of triflic anhydride (1.00 M in CH_2Cl_2, 3.01 mL, 3.01 mmol, 3.50 equiv) was added dropwise. After 5 h, an aqueous saturated sodium hydrogen carbonate solution (50mL) was added to the reaction mixture. The layers were separated and the aqueous phase was extracted with dichloromethane (2 × 40 mL). The combined organic phases were dried over magnesium sulfate and the dried solution was filtrated. The filtrate was concentrated and the residue was purified by flash column chromatography on silica gel (5% ethyl acetate in petroleum ether) to give **28c** (336 mg, 86%) as a colorless oil.

TLC (5% ethyl acetate in petroleum ether): R_f = 0.36 (UV, CAM).
^{1}H-NMR (400 MHz, Chloroform-*d*): δ = 5.69 (qd, *J* = 7.1, 1.3 Hz, 1H), 4.78 (d, *J* = 5.5 Hz, 1H), 3.34 (d, *J* = 15.3 Hz, 1H), 3.20 – 3.08 (m, 1H), 2.81 (d, *J* = 15.4, 1.2 Hz, 1H), 2.71 – 2.60 (m, 1H), 1.87 – 1.76 (m, 1H), 1.67 – 1.59 (m, 7H), 1.23 – 1.07 (m, 1H), 0.90 (s, 9H), 0.07 (s, 3H), 0.05 (s, 3H) ppm.
^{13}C-NMR (101 MHz, Chloroform-*d*): δ = 160.2, 138.1, 126.1, 118.4 (q, *J* = 320.3 Hz), 115.6, 108.5, 69.8, 34.9, 32.2, 29.2, 26.0, 24.5, 19.8, 18.3, 13.8, –4.8, –4.9 ppm.
^{19}F-NMR (376 MHz, Chloroform-*d*): δ = –73.8 ppm.
IR (Diamond–ATR, neat): 2953, 2932, 2859, 1424, 1215, 1138, 1101, 911, 837, 779 cm^{-1}.
HRMS (ESI) calc. for $C_{19}H_{30}F_3NNaO_4SSi^+$ $[M+Na]^+$: 476.1509; found: 476.1506.
$[\alpha]_D^{20}$: 56.29 (c = 0.29, CH_2Cl_2).

Methyl Ester 29c

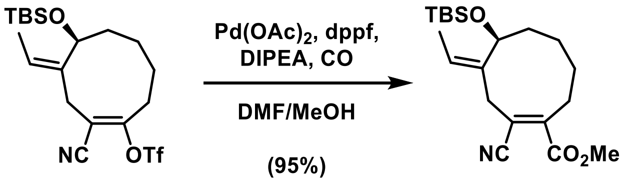

28c 29c

Note: Methanol and N,N-dimethylformamide were degassed via freeze-pump-thaw (three cycles) prior to use.

Palladium(II) acetate (11.3 mg, 50.4 µmol, 15.0 mol%), 1,1'-bis(diphenylphosphino)-ferrocene (28.0 mg, 50.4 µmol, 15.0 mol%) and *N*,*N*-diisopropylethylamine (57.8 µL, 336 µmol, 1.00 equiv) were sequentially added to a solution of **28c** (152 mg, 336 µmol, 1 equiv) in a mixture of *N*,*N*-dimethylformamide (10 mL) and methanol (1.6 mL). The flask was fitted with a balloon filled with carbon monoxide gas, and the solution was sparged with carbon monoxide for 10 minutes. The red-brownish reaction mixture was then heated to 55 °C under a carbon monoxide atmosphere. After 5 h, water (20 mL) and dichloromethane (30 mL) were added to the reaction mixture. The layers were separated, and the aqueous phase was extracted with dichloromethane (2 × 30 mL). The combined organic extracts were washed sequentially with sodium hydrogencarbonate, ammonium chloride and eventually brine. The organic phase was dried over magnesium sulfate and the dried solution was filtrated. The filtrate was concentrated, and the residue was purified by flash column chromatography on silica gel (3% ethyl acetate in petroleum ether) to give **29c** (116 mg, 95%) as a colorless oil.

TLC (9% ethyl acetate in petroleum ether): R_f = 0.26 (UV, CAM).
^{1}H-NMR (400 MHz, Chloroform-*d*): δ 5.82 = (q, *J* = 6.9 Hz, 1H), 4.75 (t, *J* = 6.1 Hz, 1H), 3.83 (s, 3H), 3.46 (d, *J* = 14.5 Hz, 1H), 2.97 (ddd, *J* = 13.1, 10.7, 4.6 Hz, 1H), 2.85 (d, *J* = 15.4 Hz, 1H), 2.59 (dt, *J* = 13.1, 5.0 Hz, 1H), 1.89 – 1.76 (m, 1H), 1.63 (d, *J* = 7.1 Hz, 3H), 1.60 – 1.42 (m, 4H), 1.02 – 0.91 (m, 1H), 0.89 (s, 9H), 0.06 (s, 3H), 0.03 (s, 3H) ppm.
^{13}C-NMR (101 MHz, Chloroform-*d*): δ = 166.2, 146.1, 138.2, 126.0, 120.5, 119.6, 70.4, 52.6, 34.5, 34.3, 27.0, 26.0, 25.9, 20.1, 18.3, 13.7, – 4.7, – 4.9 ppm.
IR (Diamond–ATR, neat): 2952, 2929, 2857, 1727, 1388, 1231, 1183, 1054, 835, 776 cm^{-1}.
HRMS (ESI) calc. for $C_{20}H_{33}NNaO_3Si^+$ $[M+Na]^+$: 386.2122; found: 386.2118.
$[\alpha]_D^{20}$: 65.64 (c = 0.26, CH_2Cl_2).

Derivative 10

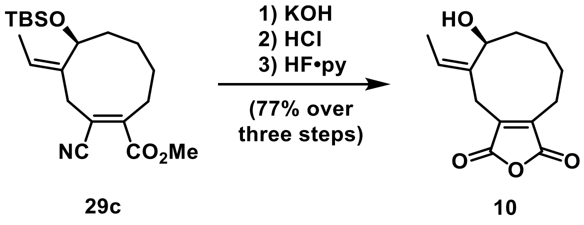

A solution of potassium hydroxide (10 wt.% in water, 1.0 mL) was added to a solution of **29c** (100 mg, 275 µmol, 1 equiv) in *iso*-propanol (12 mL) and the mixture was heated to 70 °C. After 3 h, the reaction mixture was allowed to cool to 23 °C and aqueous saturated ammonium chloride solution (25 mL) and dichloromethane (30 mL) were added. The layers were separated and the aqueous phase was extracted with dichloromethane (2 × 20 mL). The combined organic phases were dried over magnesium sulfate and the dried solution was filtrated. The filtrate was concentrated to give crude **S10** which was used in the next step without further purification.

Aqueous hydrochloric acid (0.2 M, 1.30 mL) was added to a solution of crude **S10** (assumed 275 µmol, 1 equiv) in tetrahydrofuran (12 mL) at 23 °C. After 45 min, aqueous hydrochloric acid (0.2 M, 10 mL) and dichloromethane (20 mL) were added to the reaction mixture. The layers were separated and the aqueous phase was extracted with dichloromethane (2 × 20 mL). The combined organic phases were dried over magnesium sulfate and the dried solution was filtrated. The filtrate was concentrated to give crude anhydride **S11** as a yellowish oil which was used in the following deprotection step without further purification.

Pyridine hydrofluoride (≈70% hydrogen fluoride, ≈30% pyridine, 500 µL, 3.90 mmol, 14.0 equiv) was added to a solution of crude **S11** (assumed 275 µmol) in tetrahydrofuran (8 mL) at 0 °C and the reaction was allowed to warm to 23 °C. After 18 h, aqueous hydrochloric acid (2 M, 20 mL) and dichloromethane (20 mL) were added to the reaction mixture. The layers were separated and the aqueous phase was extracted with dichloromethane (2 × 20 mL). The combined organic phases were dried over magnesium sulfate and the dried solution was filtrated. The filtrate was concentrated and the residue was purified by flash column chromatography on silica gel (25% ethyl acetate in petroleum ether) to give **10** (50.0 mg, 77% over three steps) as a colorless oil.

TLC (33% ethyl acetate in petroleum ether): R_f = 0.17 (UV, CAM).

^{1}H-NMR (400 MHz, Chloroform-*d*): δ = 5.89 (q, *J* = 7.1 Hz, 1H), 4.74 (dd, *J* = 10.2, 5.3 Hz, 1H), 3.31 (d, *J* = 15.1 Hz, 1H), 3.16 (d, 15.4 Hz, 1H), 2.76 – 2.60 (m, 2H), 1.86 – 1.73 (m, 2H), 1.71 (d, *J* = 7.0 Hz, 3H), 1.68 – 1.53 (m, 4H), 0.88 – 0.73 (m, 1H) ppm.

^{13}C-NMR (101 MHz, Chloroform-*d*): δ = 166.6, 166.4, 144.6, 141.6, 136.3, 130.8, 68.8, 29.1, 25.2, 24.6, 20.6, 19.6, 13.3 ppm.

IR (Diamond–ATR, neat): 3392, 2928, 1763, 1451, 1276, 1040, 980, 923, 802, 775 cm^{-1}.

HRMS (ESI) calc. for $C_{13}H_{15}O_4^-$ [M-H]$^+$: 235.0976; found: 235.0974.

$[\alpha]_D^{20}$: 43 (c = 0.2, CH_2Cl_2).

References

1) S. Gerhardt, S. Echt, M. Busch, J. Freigang, G. Auerbach, G. Bader, W. F. Martin, A. Bacher, R. Huber, M. Fischer, Plant Physiol. **2003**, 132(4), 1941-1949.
2) A. Vagin, A. Teplyakov, *Acta Crystallogr. D* **2010**, 66, 22-25.
3) P. Emsley, K. Cowtan, *Acta Crystallogr. D* **2004**, 60, 2126-2132.
4) G. N. Murshudov, P. Skubak, A. A. Lebedev, N. S. Pannu, R. A. Steiner, R. A. Nicholls, M. D. Winn, F. Long, A. A. Vagin, *Acta Crystallogr. D* **2011**, 67, 355-367.
5) T. R. Hoye, C. S Jeffrey, F. Shao, *Nature Protocols* **2007**, *2*, 2451–2458.

^{1}H and ^{13}C NMR spectra

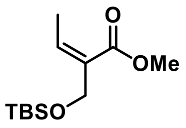

12

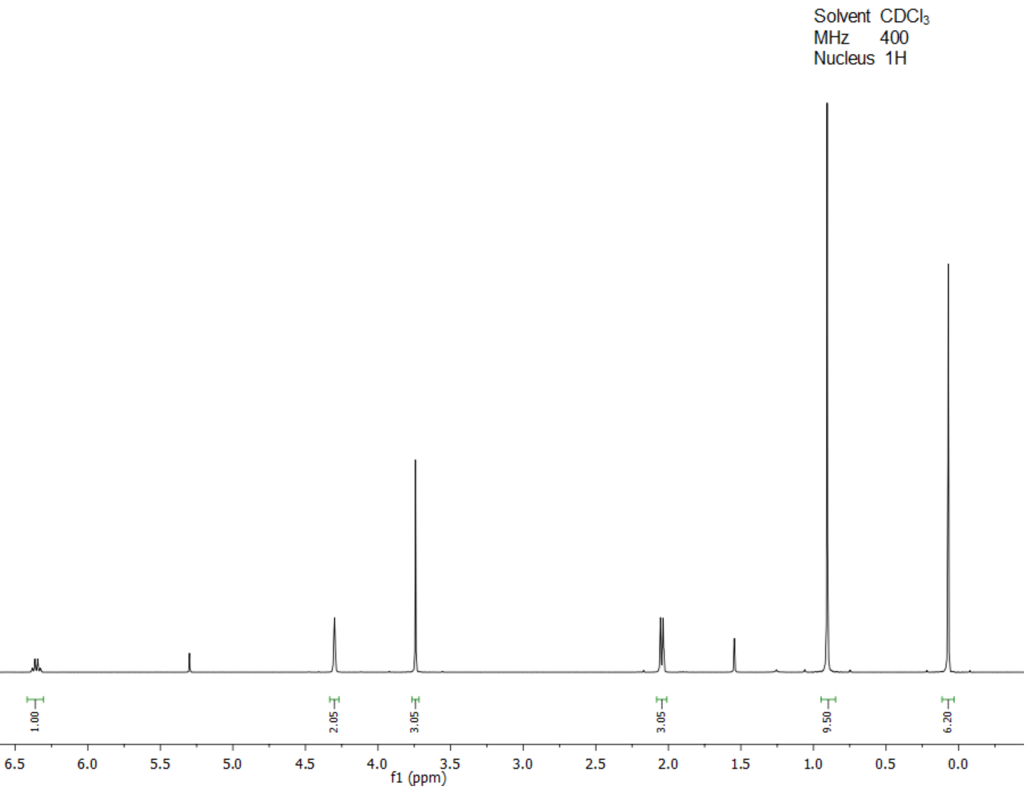

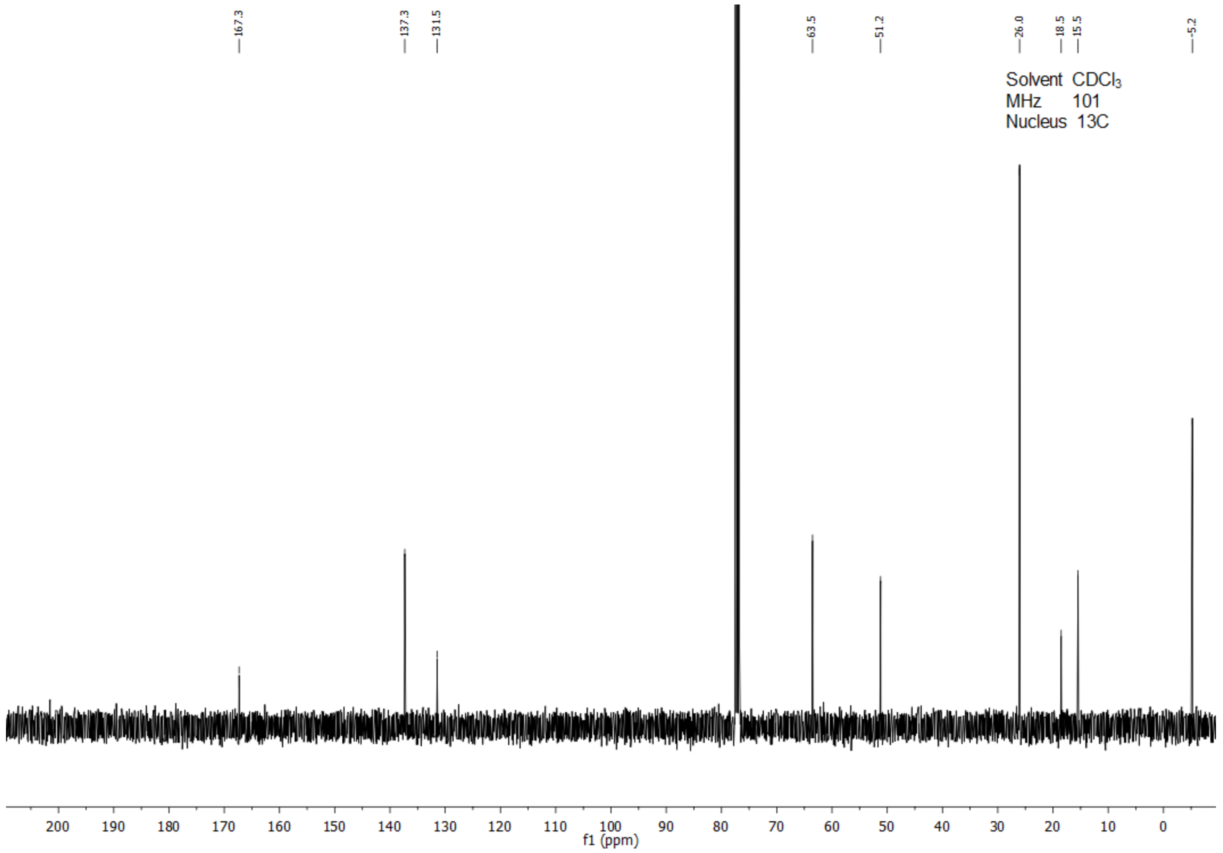

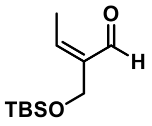
O
H
TBSO

13

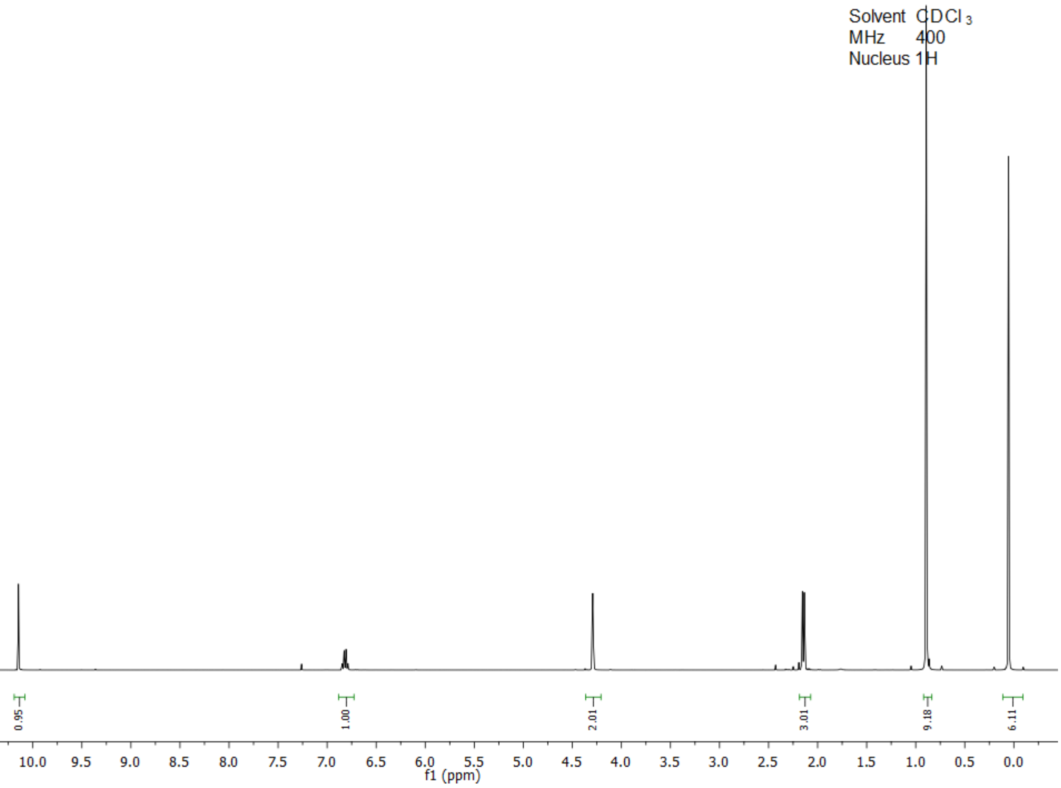
Solvent CDCl 3
MHz 400
Nucleus 1H
0.95
1.00
2.01
3.01
9.18
6.11
11.5 11.0 10.5 10.0 9.5 9.0 8.5 8.0 7.5 7.0 6.5 6.0 5.5 5.0 4.5 4.0 3.5 3.0 2.5 2.0 1.5 1.0 0.5 0.0
f1 (ppm)

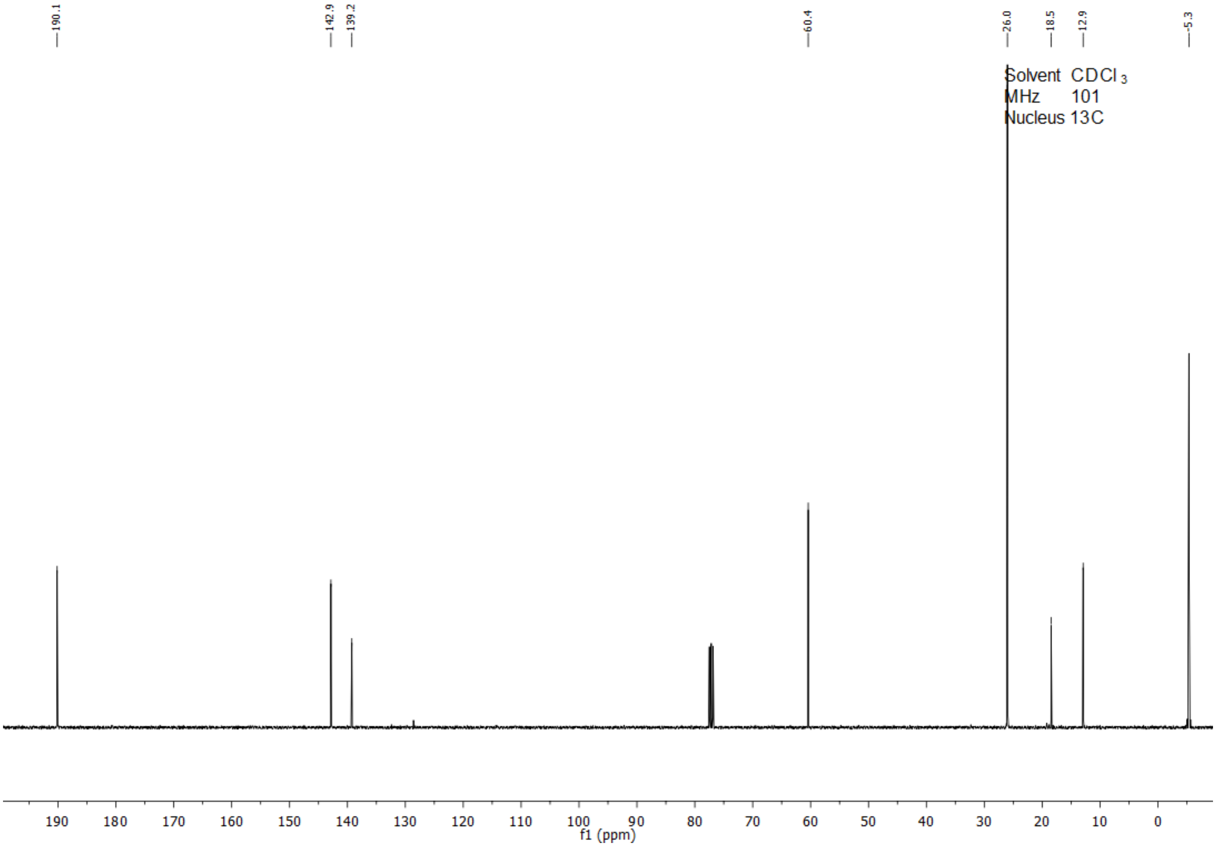
190.1
142.9
139.2
60.4
26.0
18.5
12.9
-5.3
Solvent CDCl 3
MHz 101
Nucleus 13C
190 180 170 160 150 140 130 120 110 100 90 80 70 60 50 40 30 20 10 0
f1 (ppm)

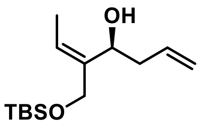
OH
TBSO

S2

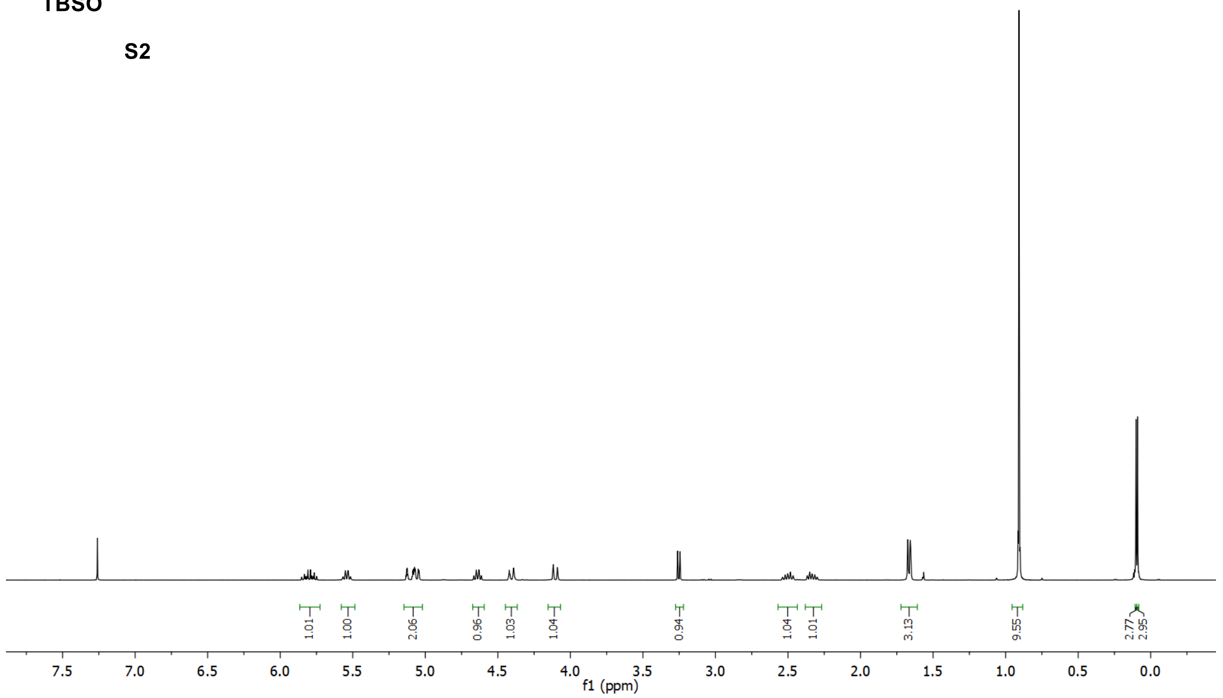
Solvent CDCl3
MHz 400
Nucleus 1H
7.5
7.0
6.5
6.0
5.5
5.0
4.5
4.0
3.5
3.0
2.5
2.0
1.5
1.0
0.5
0.0
f1 (ppm)

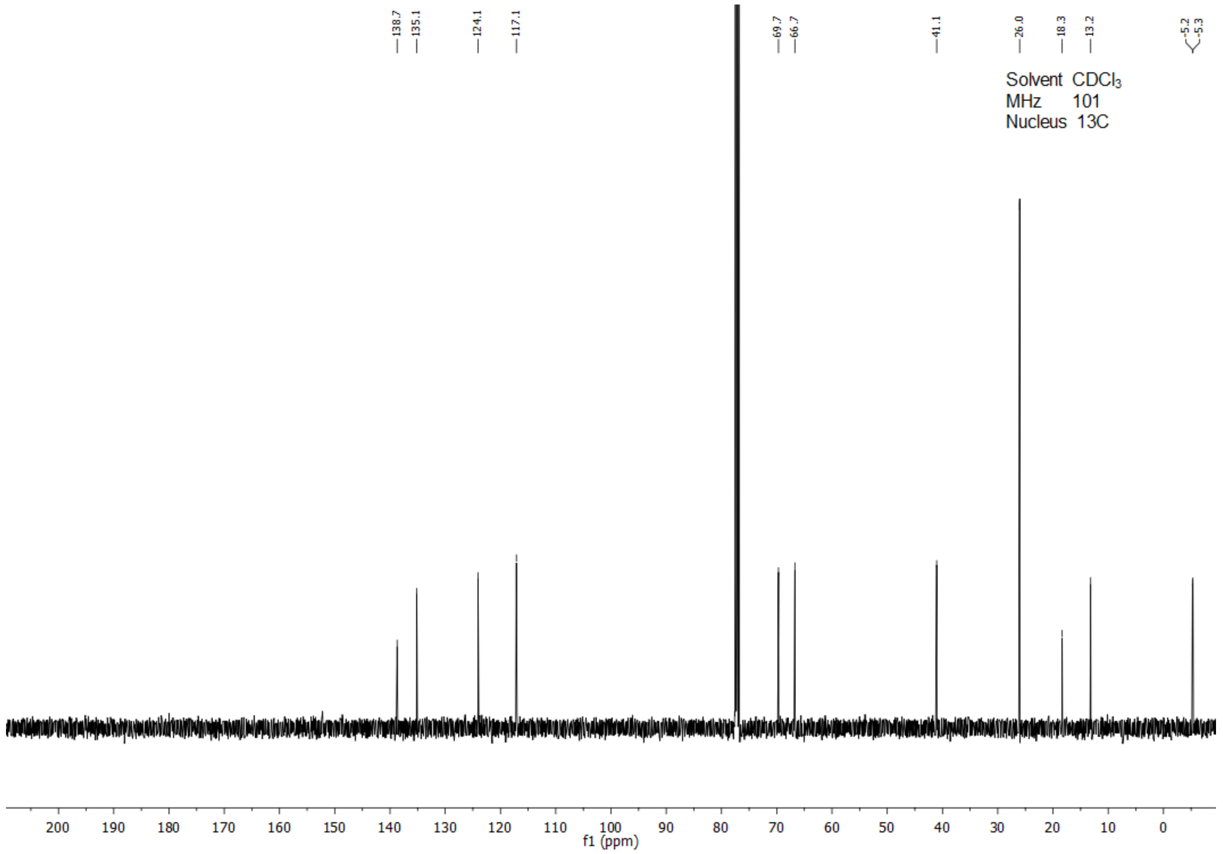
138.7
135.1
124.1
117.1
69.7
66.7
41.1
26.0
18.3
13.2
-5.2
-5.3
Solvent CDCl3
MHz 101
Nucleus 13C
200
190
180
170
160
150
140
130
120
110
100
90
80
70
60
50
40
30
20
10
0
f1 (ppm)

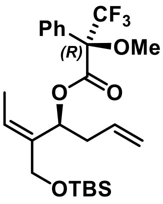
Ph
CF3
(R)
OMe
O
O
OTBS

S3

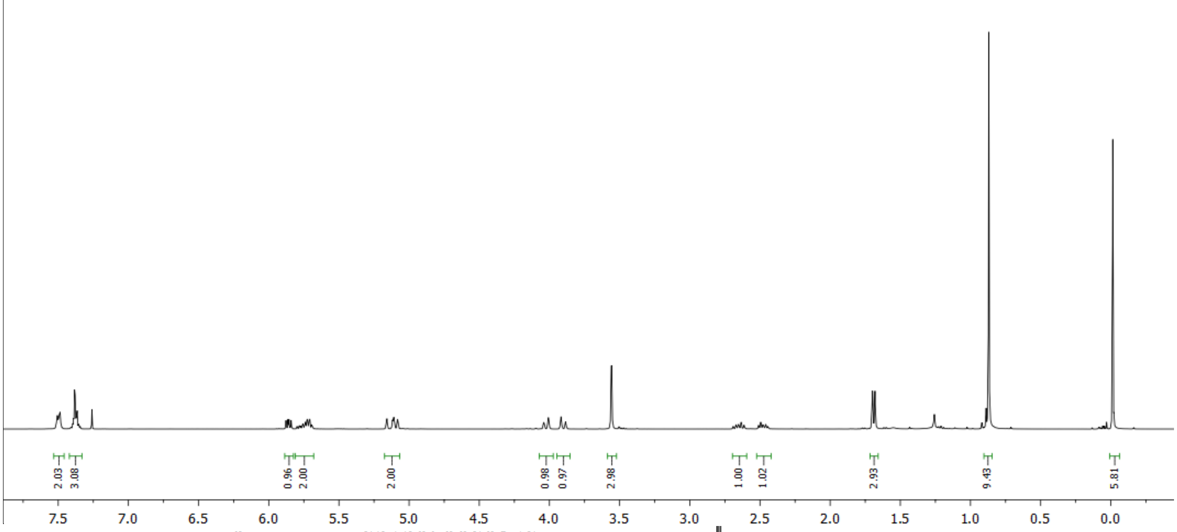
Solvent CDCl3
MHz 400
Nucleus 1H
2.03
3.08
0.96
2.00
2.00
0.98
0.97
2.98
1.00
1.02
2.93
9.43
5.81

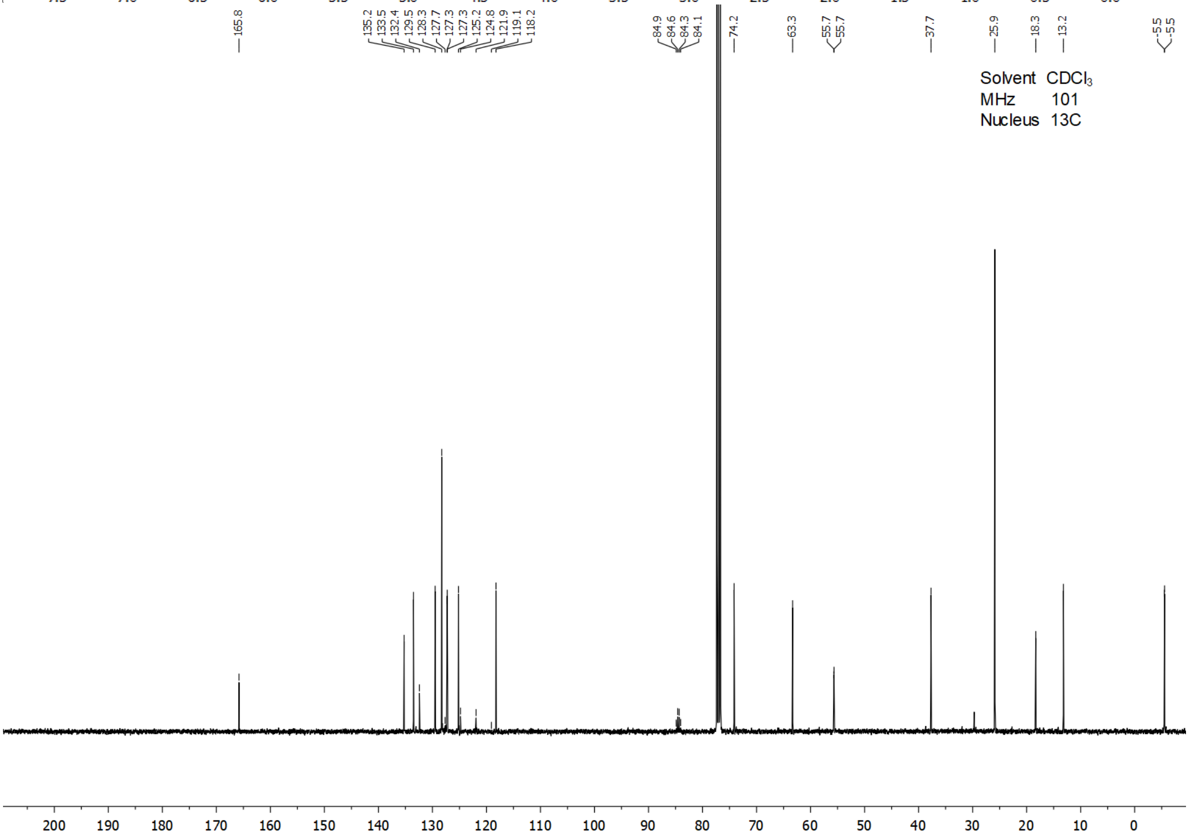
165.8
135.2
133.5
132.4
129.5
128.3
127.7
127.3
127.3
125.2
124.8
121.9
119.1
118.2
84.9
84.6
84.3
84.1
74.2
63.3
55.7
55.7
37.7
25.9
18.3
13.2
-5.5
-5.5
Solvent CDCl3
MHz 101
Nucleus 13C

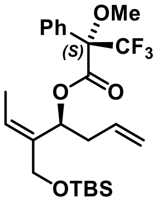
OMe
Ph
(S)
CF_3
O
O
OTBS

S4

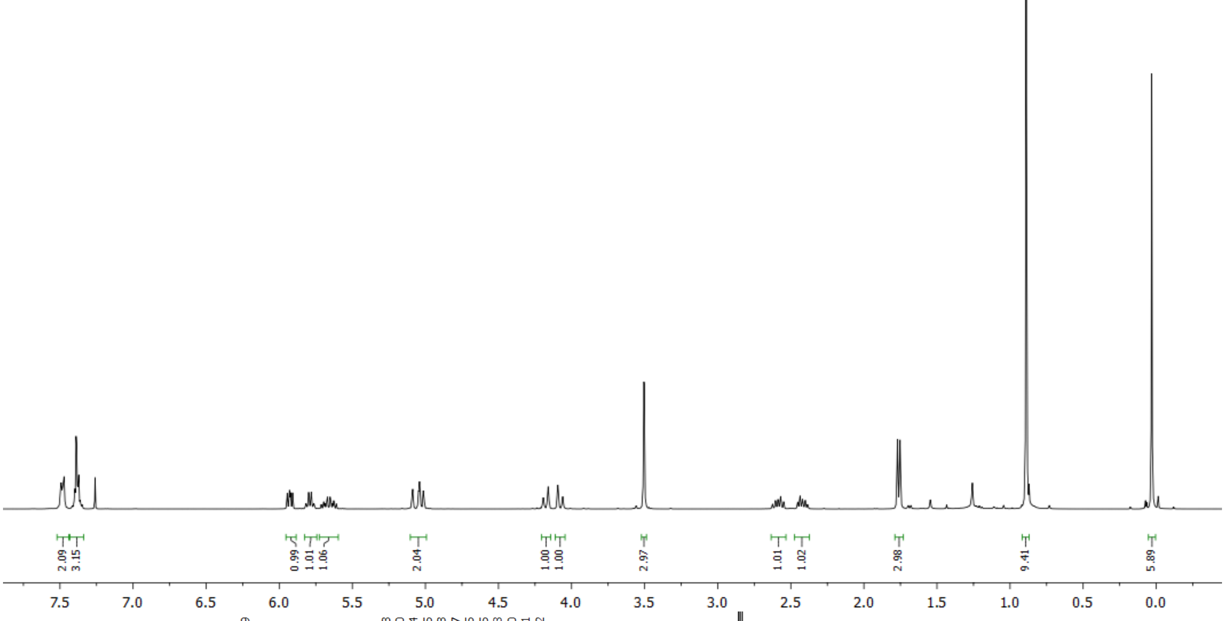
Solvent CDCl3
MHz 400
Nucleus 1H
2.09 3.15 0.99 1.01 1.06 2.04 1.00 1.00 2.97 1.01 1.02 2.98 9.41 5.89
7.5 7.0 6.5 6.0 5.5 5.0 4.5 4.0 3.5 3.0 2.5 2.0 1.5 1.0 0.5 0.0

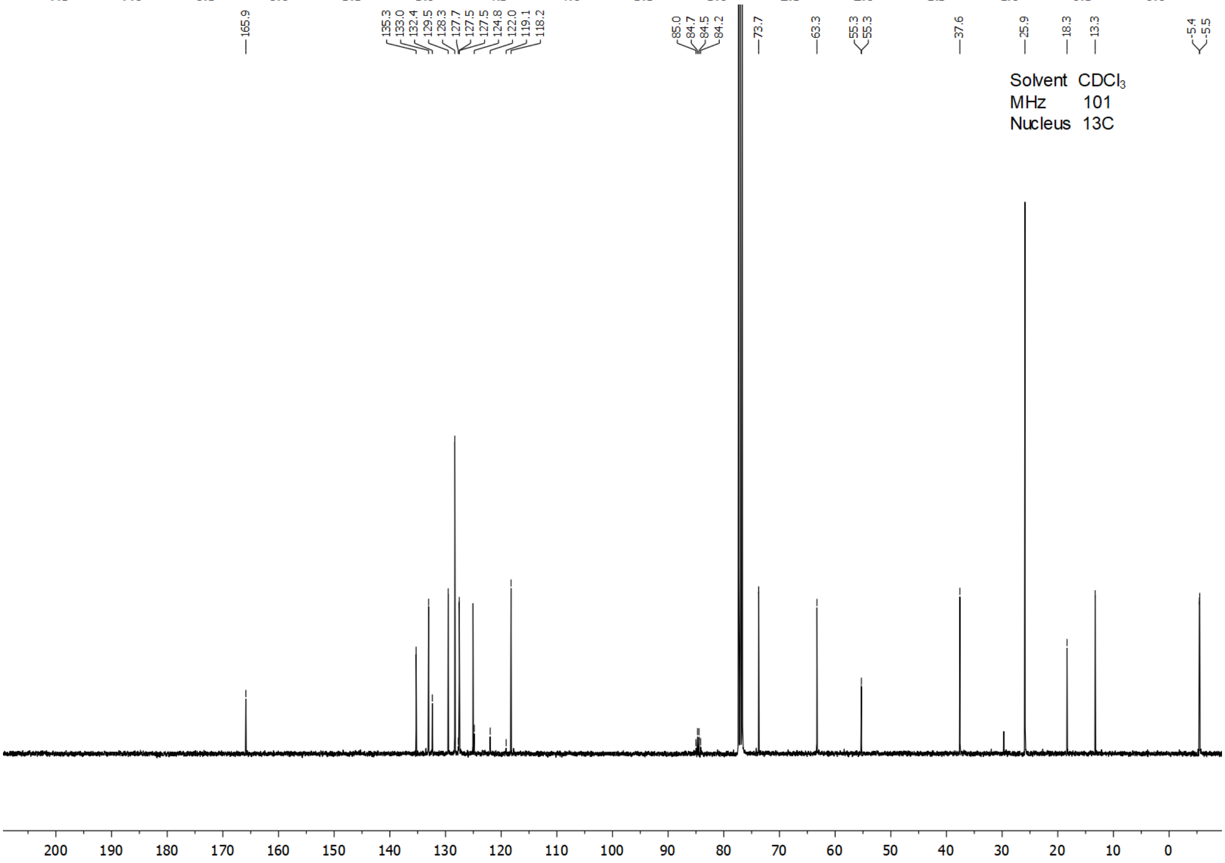
165.9
135.3 133.0 132.4 129.5 128.3 127.7 127.5 127.5 124.8 122.0 119.1 118.2
85.0 84.7 84.5 84.2
73.7
63.3
55.3 55.3
37.6
25.9
18.3
13.3
-5.4 -5.5
Solvent CDCl3
MHz 101
Nucleus 13C
200 190 180 170 160 150 140 130 120 110 100 90 80 70 60 50 40 30 20 10 0

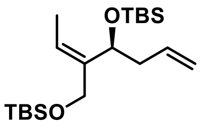
OTBS
TBSO

14

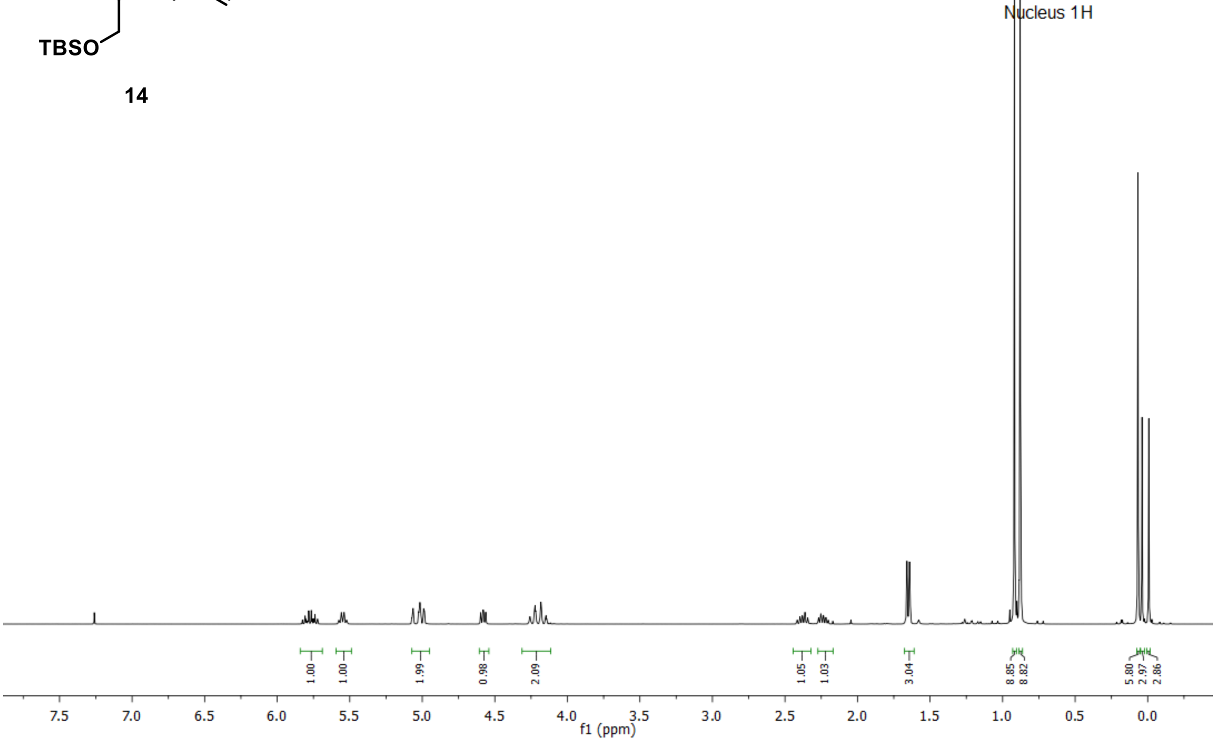
Solvent CDCl3
MHz 400
Nucleus 1H
1.00
1.00
1.99
0.98
2.09
1.05
1.03
3.04
8.85
8.82
5.80
2.97
2.86
f1 (ppm)

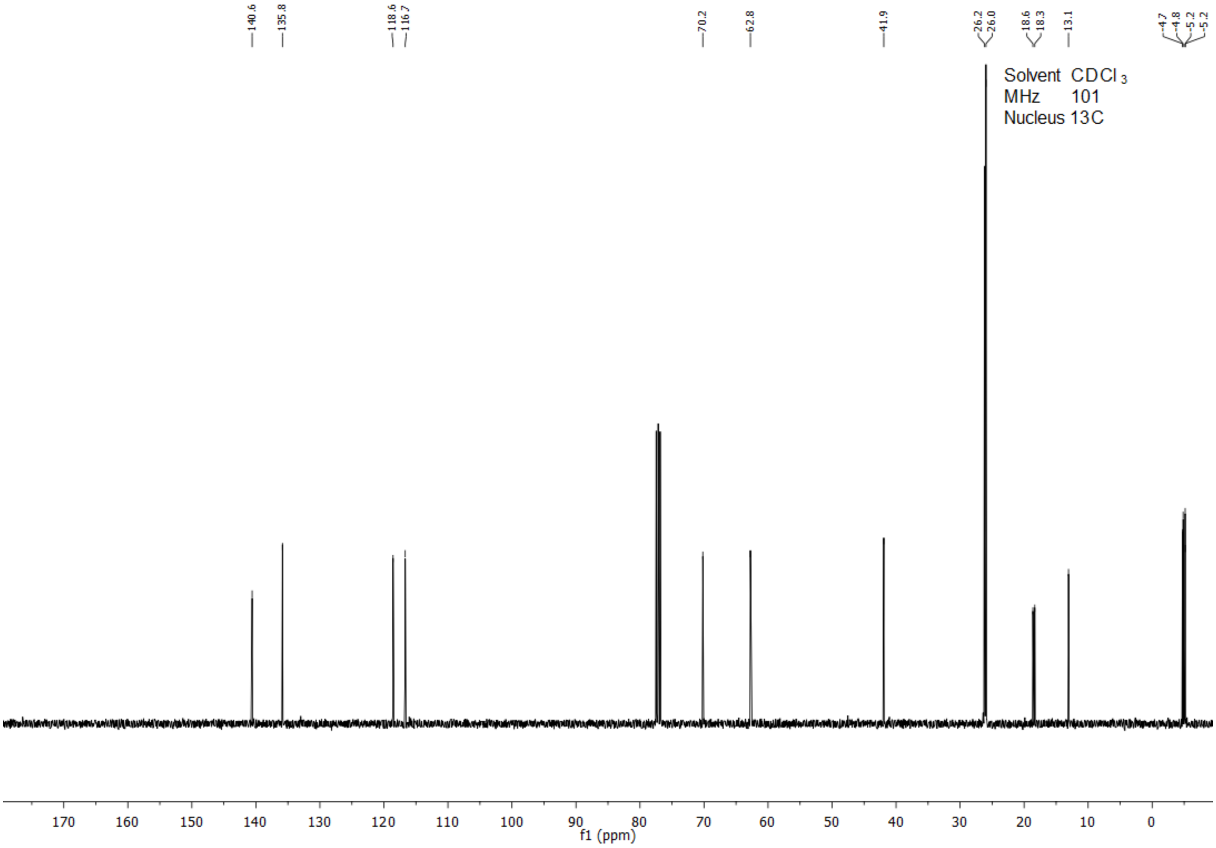
140.6
135.8
118.6
116.7
70.2
62.8
41.9
26.2
26.0
18.6
18.3
13.1
-4.7
-4.8
-5.2
-5.2
Solvent CDCl3
MHz 101
Nucleus 13C
f1 (ppm)

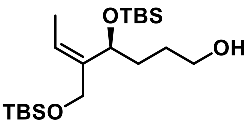
OTBS
OH
TBSO

15

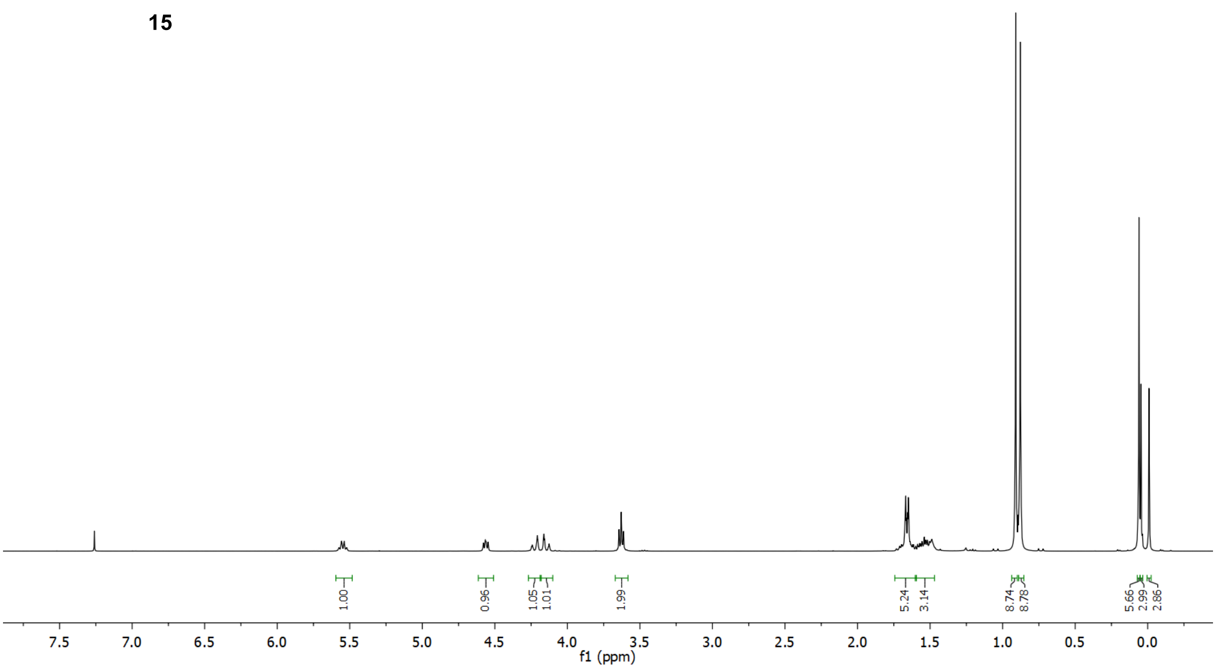
Solvent CDCl3
MHz 400
Nucleus 1H
1.00
0.96
1.05
1.01
1.99
5.24
3.14
8.74
8.78
5.66
2.99
2.86
f1 (ppm)

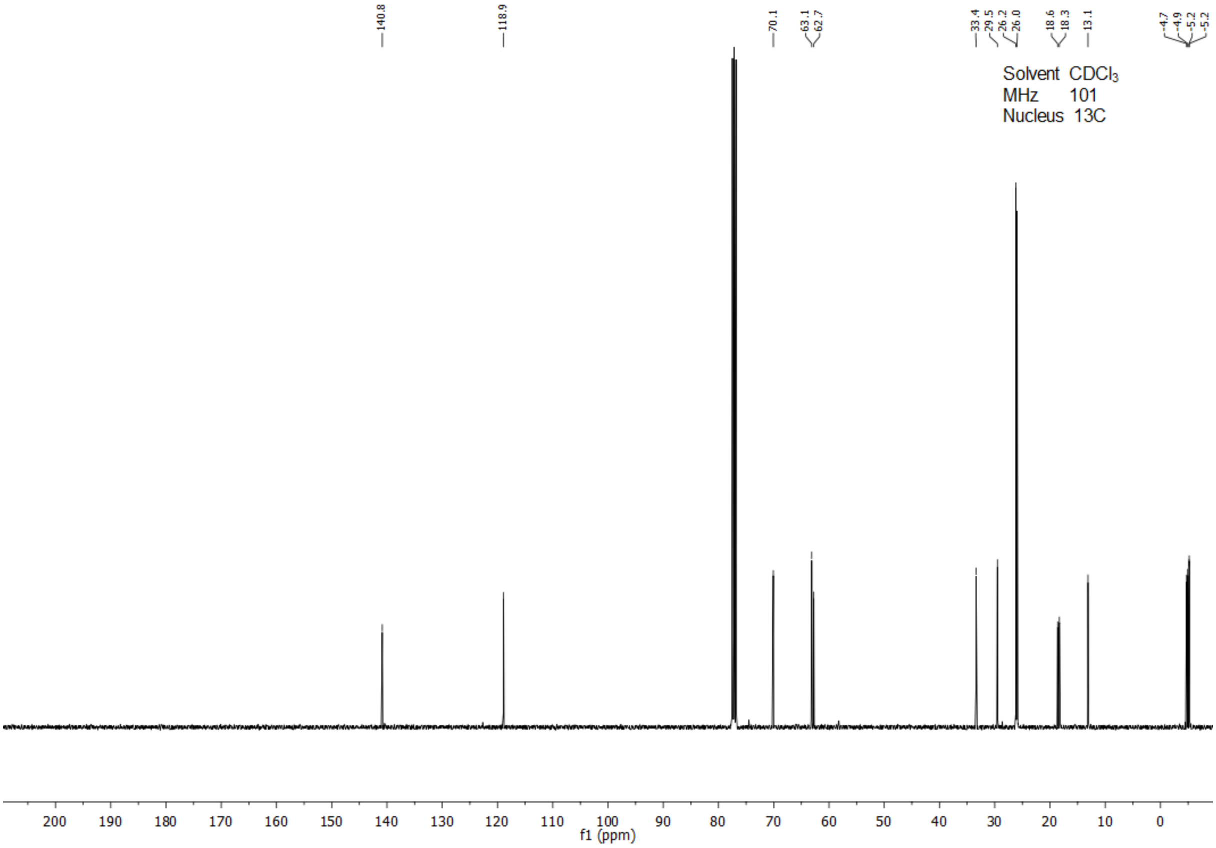
140.8
118.9
70.1
63.1
62.7
33.4
29.5
26.2
26.0
18.6
18.3
13.1
-4.7
-4.9
-5.2
-5.2
Solvent CDCl3
MHz 101
Nucleus 13C
f1 (ppm)

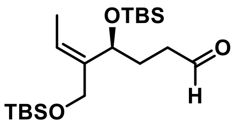
OTBS
O
H
TBSO

16

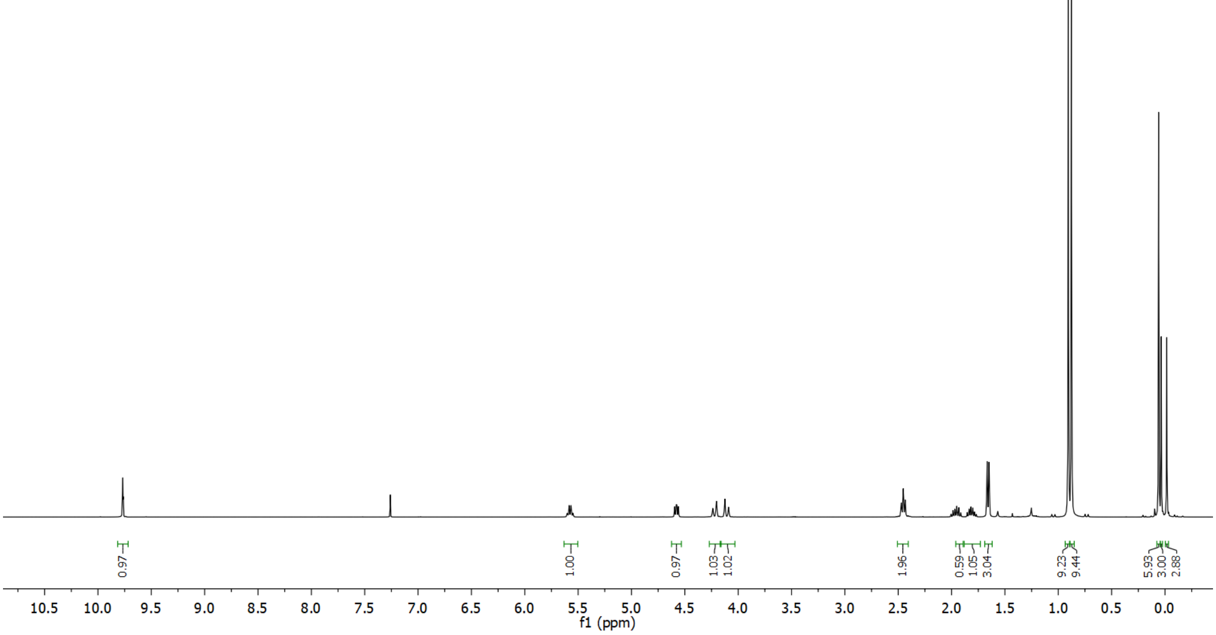
Solvent CDCl3
MHz 400
Nucleus 1H
0.97
1.00
0.97
1.03
1.02
1.96
0.59
1.05
3.04
9.23
9.44
5.93
3.00
2.88
10.5 10.0 9.5 9.0 8.5 8.0 7.5 7.0 6.5 6.0 5.5 5.0 4.5 4.0 3.5 3.0 2.5 2.0 1.5 1.0 0.5 0.0
f1 (ppm)

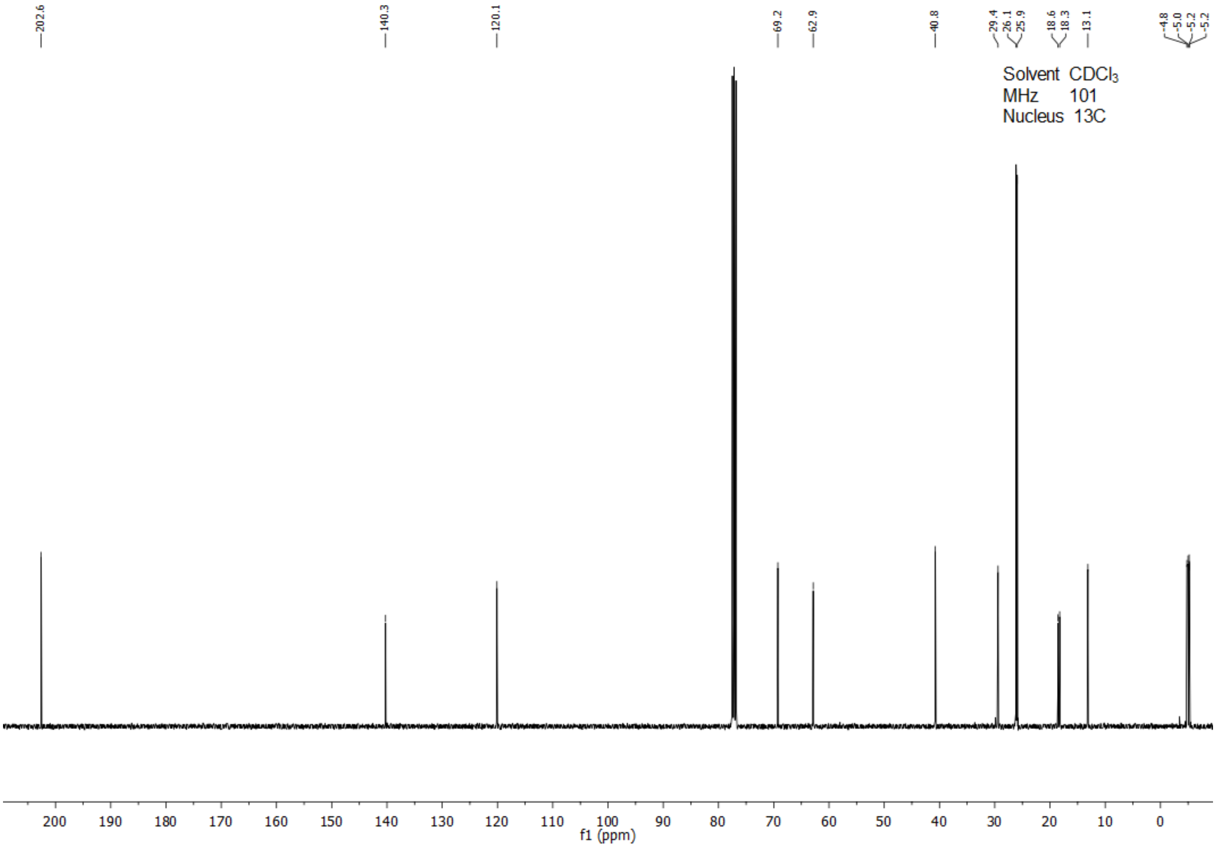
202.6
140.3
120.1
69.2
62.9
40.8
29.4
26.1
25.9
18.6
18.3
13.1
-4.8
-5.0
-5.2
-5.2
Solvent CDCl3
MHz 101
Nucleus 13C
200 190 180 170 160 150 140 130 120 110 100 90 80 70 60 50 40 30 20 10 0
f1 (ppm)

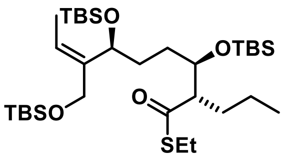
TBSO
OTBS
TBSO
O
SEt

20a

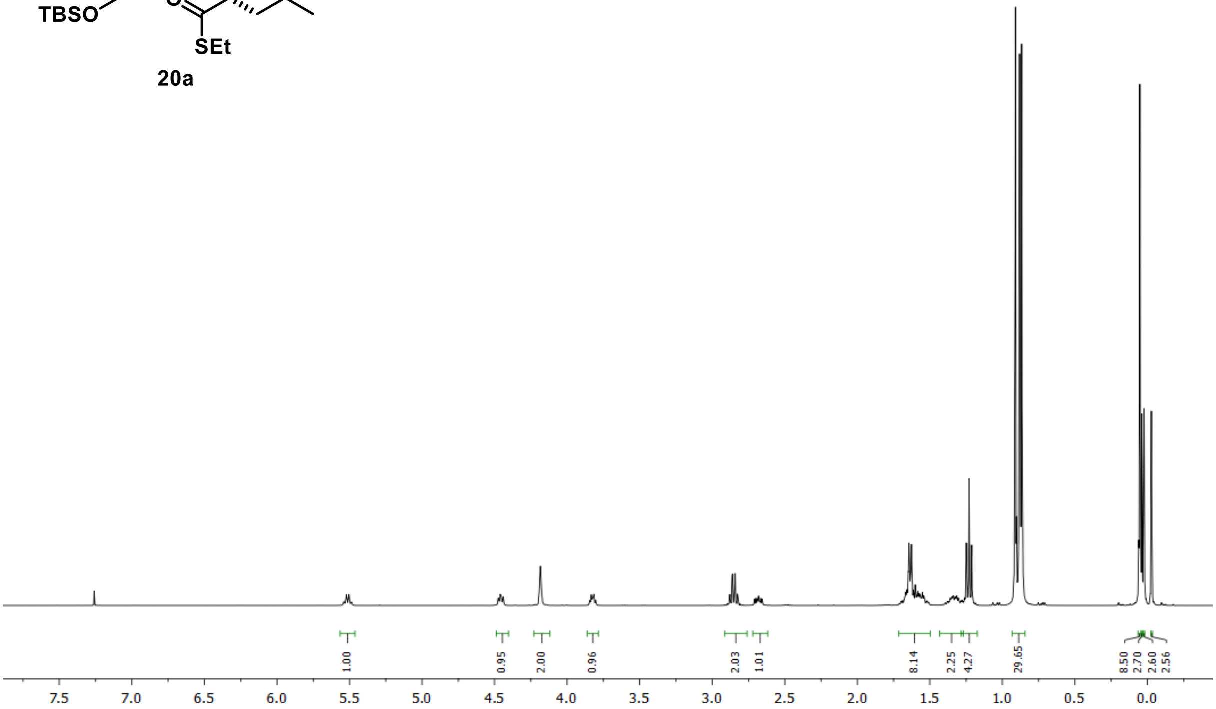
Solvent CDCl3
MHz 400
Nucleus 1H

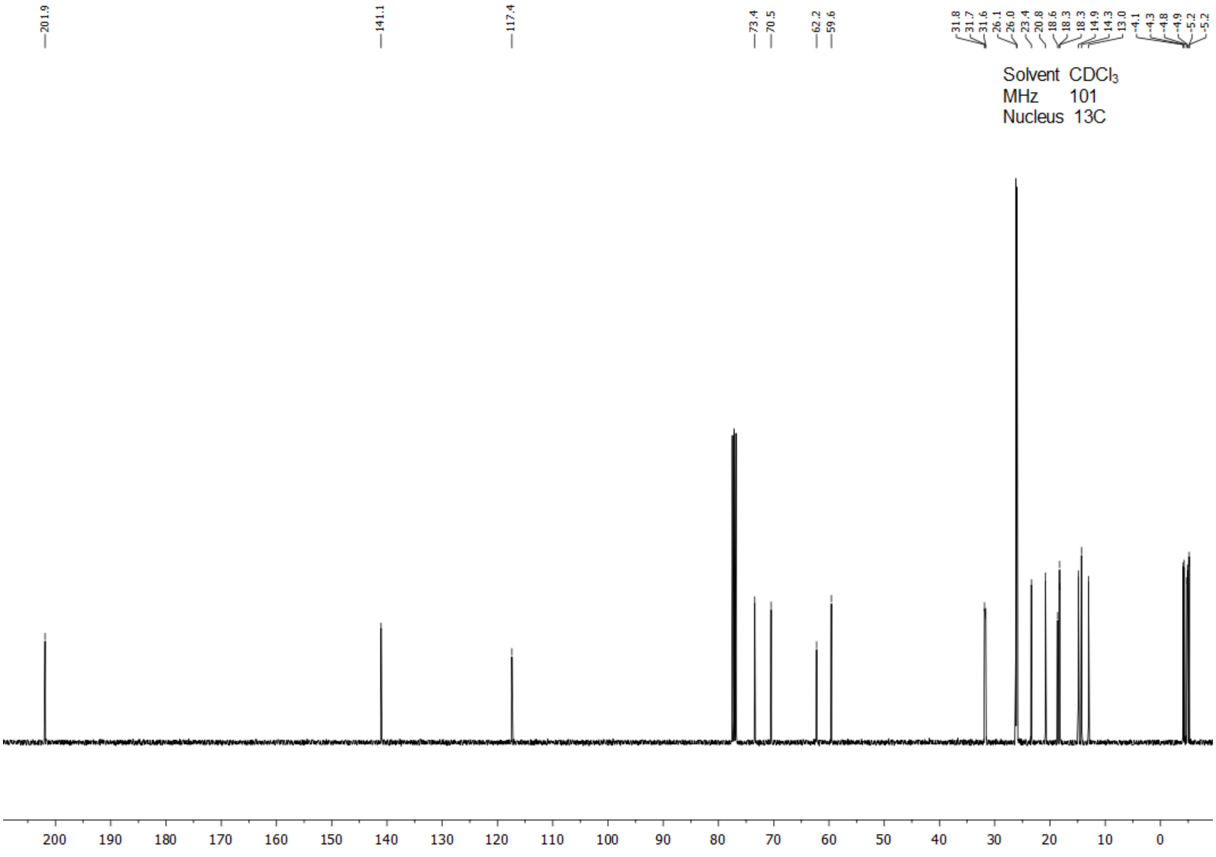
Solvent CDCl3
MHz 101
Nucleus 13C

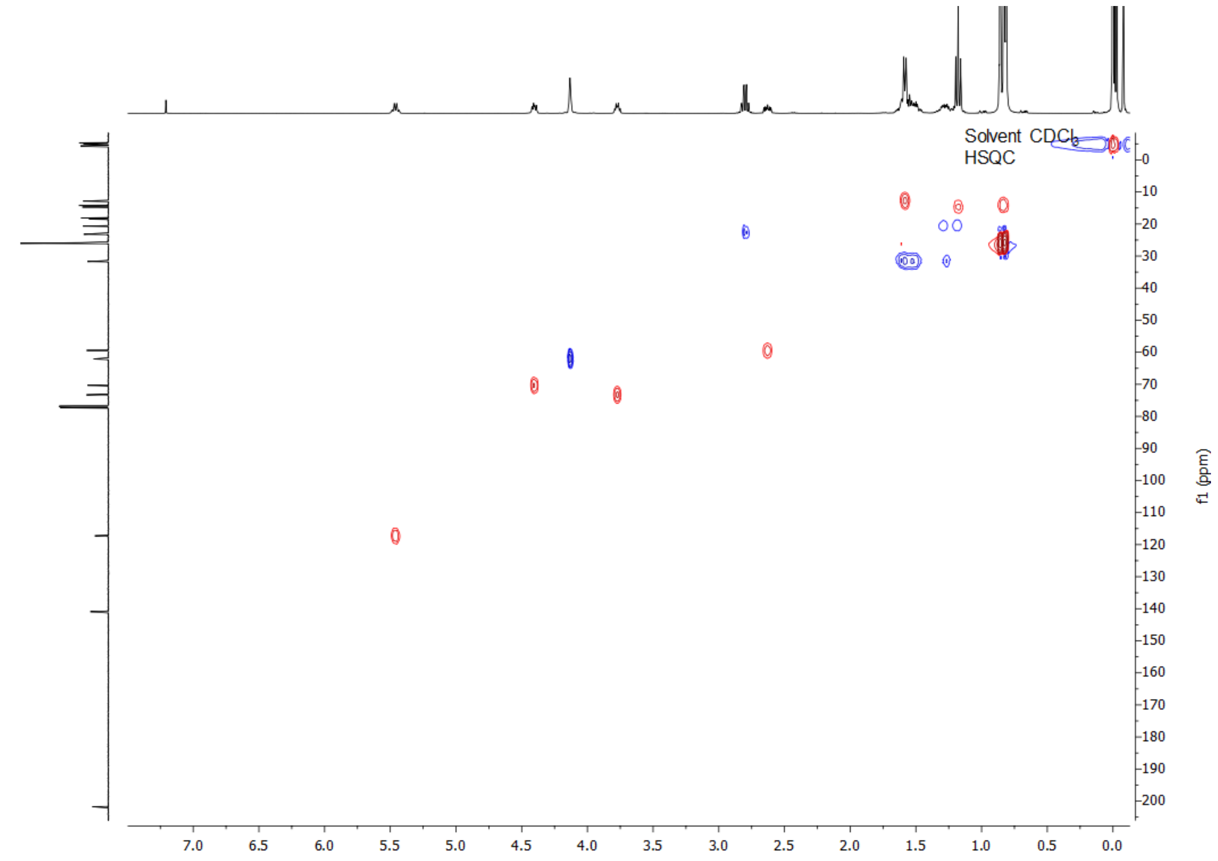
Solvent CDCl3
HSQC
f1 (ppm)

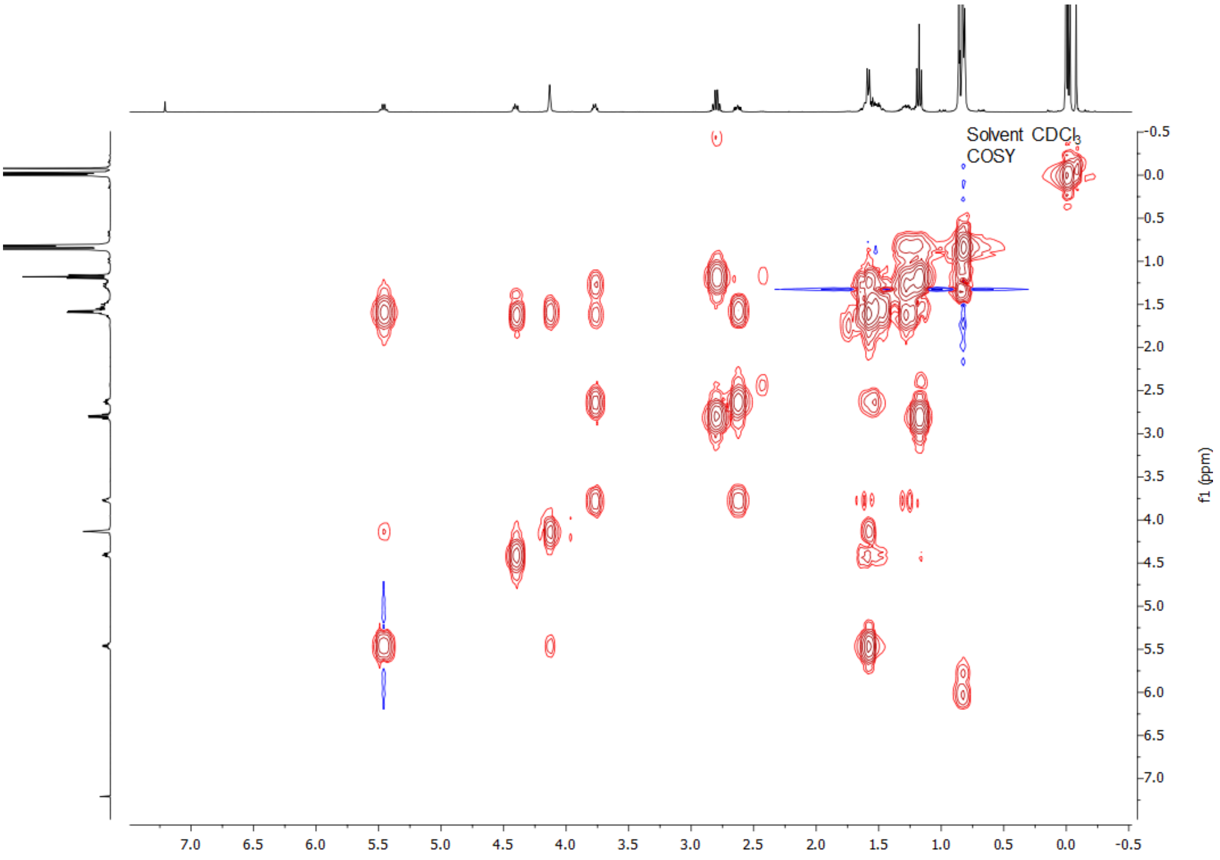
Solvent CDCl3
COSY
f1 (ppm)

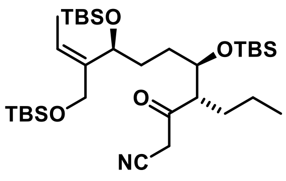
TBSO
OTBS
TBSO
O
NC

21a

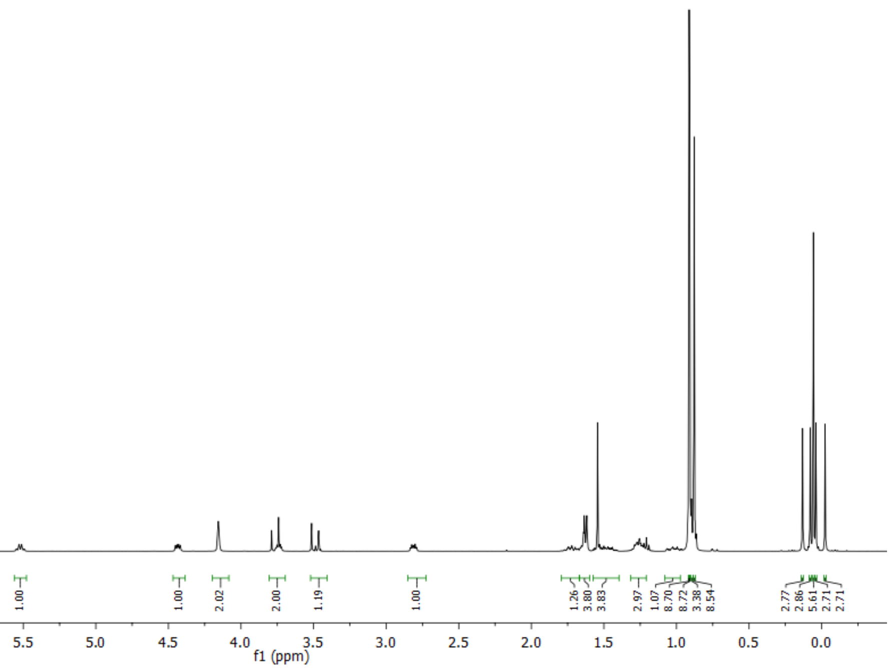
Solvent CDCl3
MHz 400
Nucleus 1H
f1 (ppm)

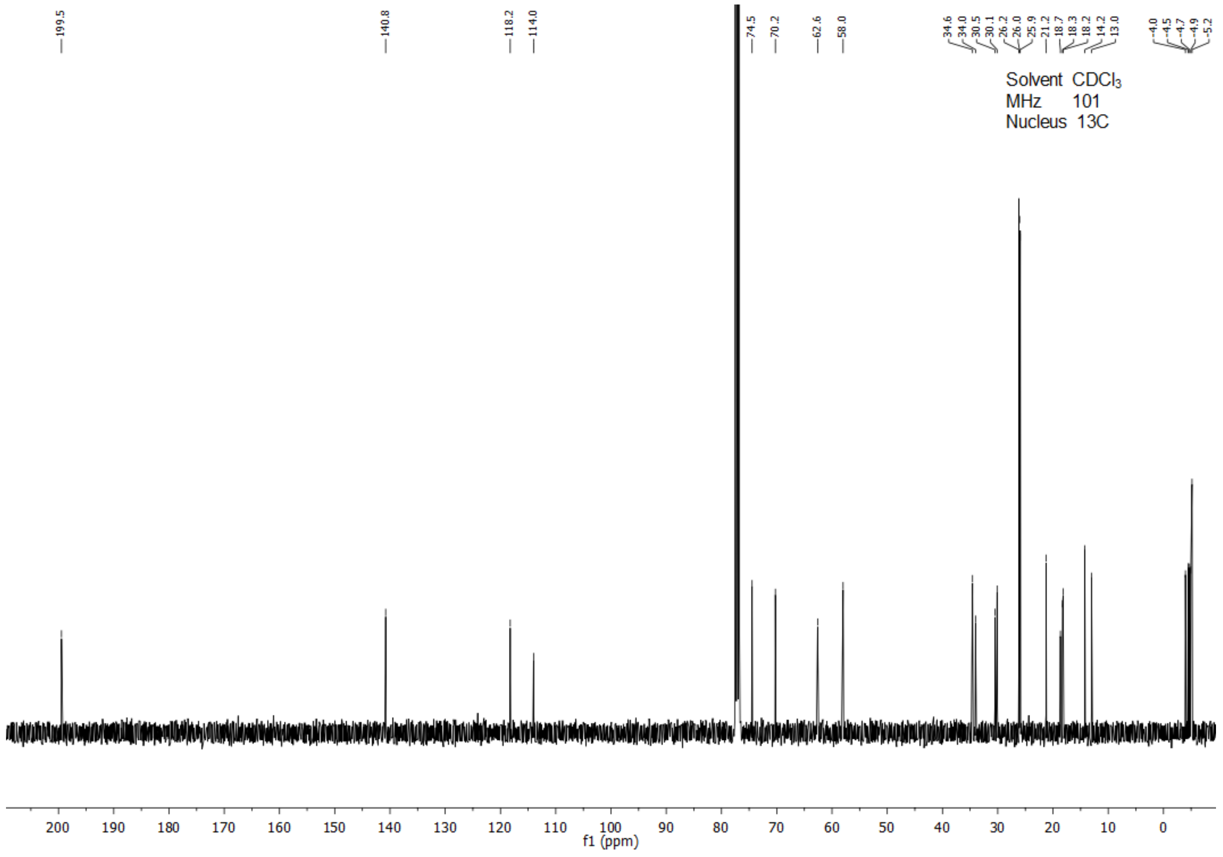
199.5
140.8
118.2
114.0
74.5
70.2
62.6
58.0
34.6
34.0
30.5
30.1
26.2
26.0
25.9
21.2
18.7
18.3
18.2
14.2
13.0
-4.0
-4.5
-4.7
-4.9
-5.2
Solvent CDCl3
MHz 101
Nucleus 13C
f1 (ppm)

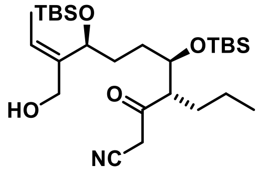
TBSO
OTBS
O
HO
NC

25a

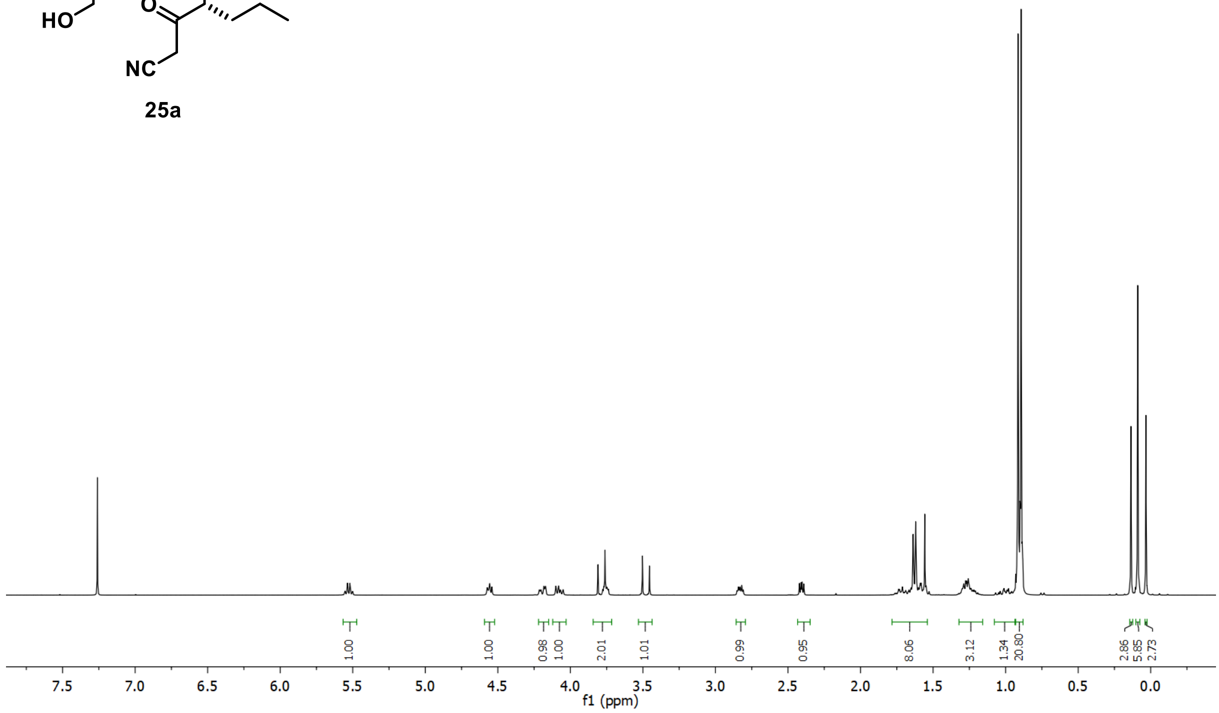
Solvent CDCl3
MHz 400
Nucleus 1H
f1 (ppm)

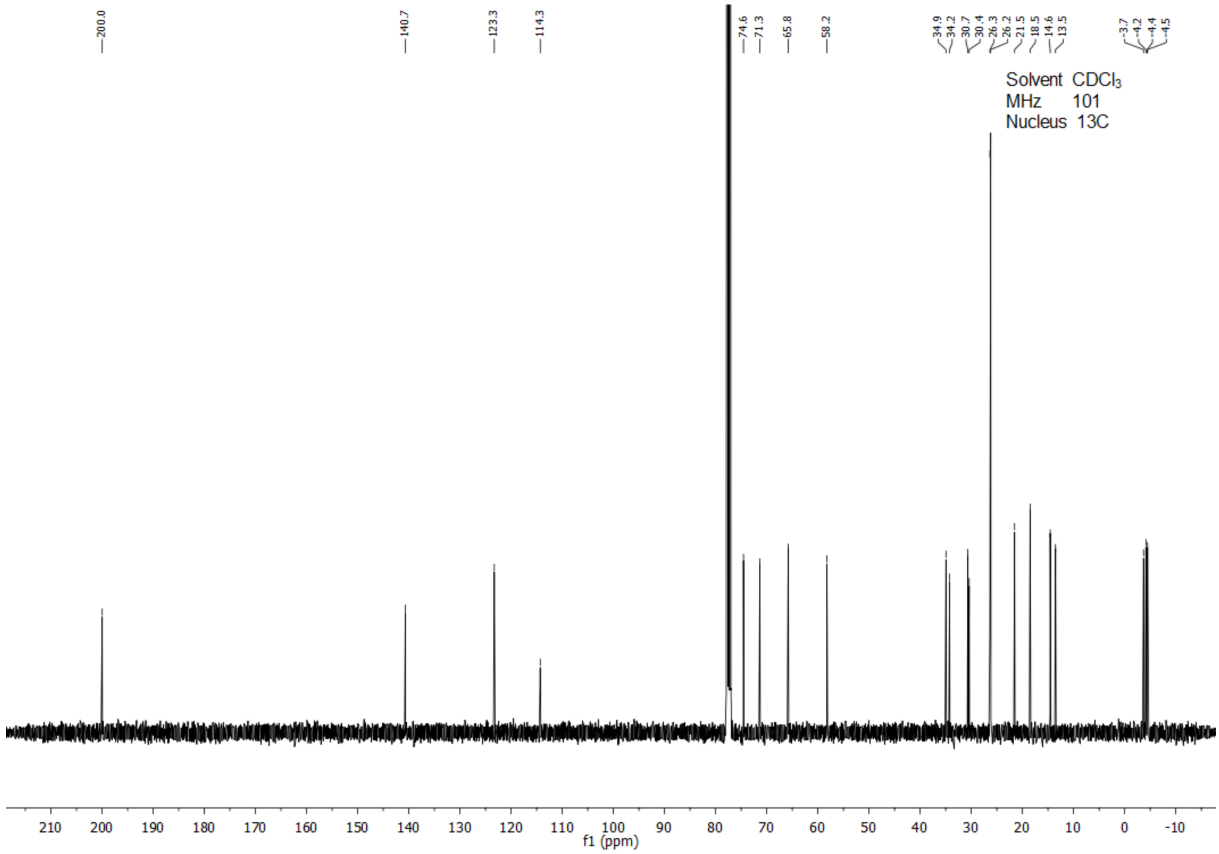
Solvent CDCl3
MHz 101
Nucleus 13C
f1 (ppm)

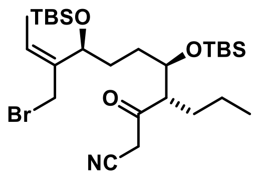
TBSO
OTBS
O
Br
NC

26a

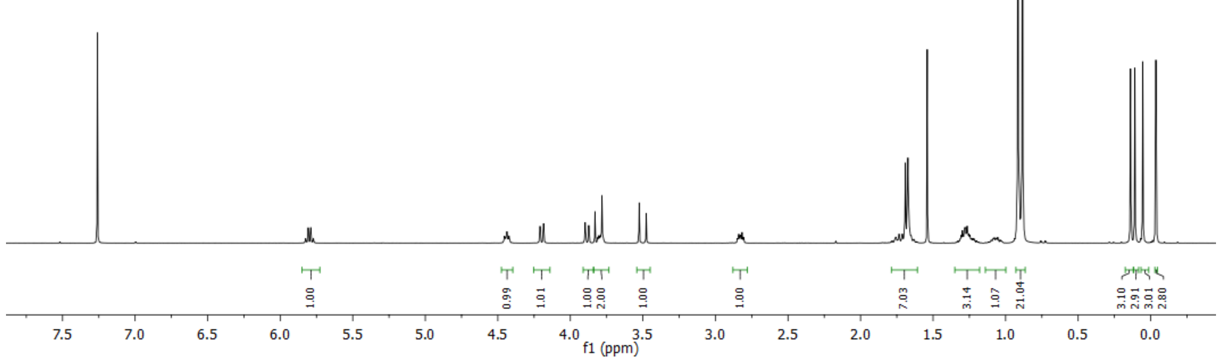
Solvent CDCl3
MHz 400
Nucleus 1H
1.00
0.99
1.01
1.00
2.00
1.00
1.00
7.03
3.14
1.07
21.04
3.10
2.91
3.01
2.80
f1 (ppm)

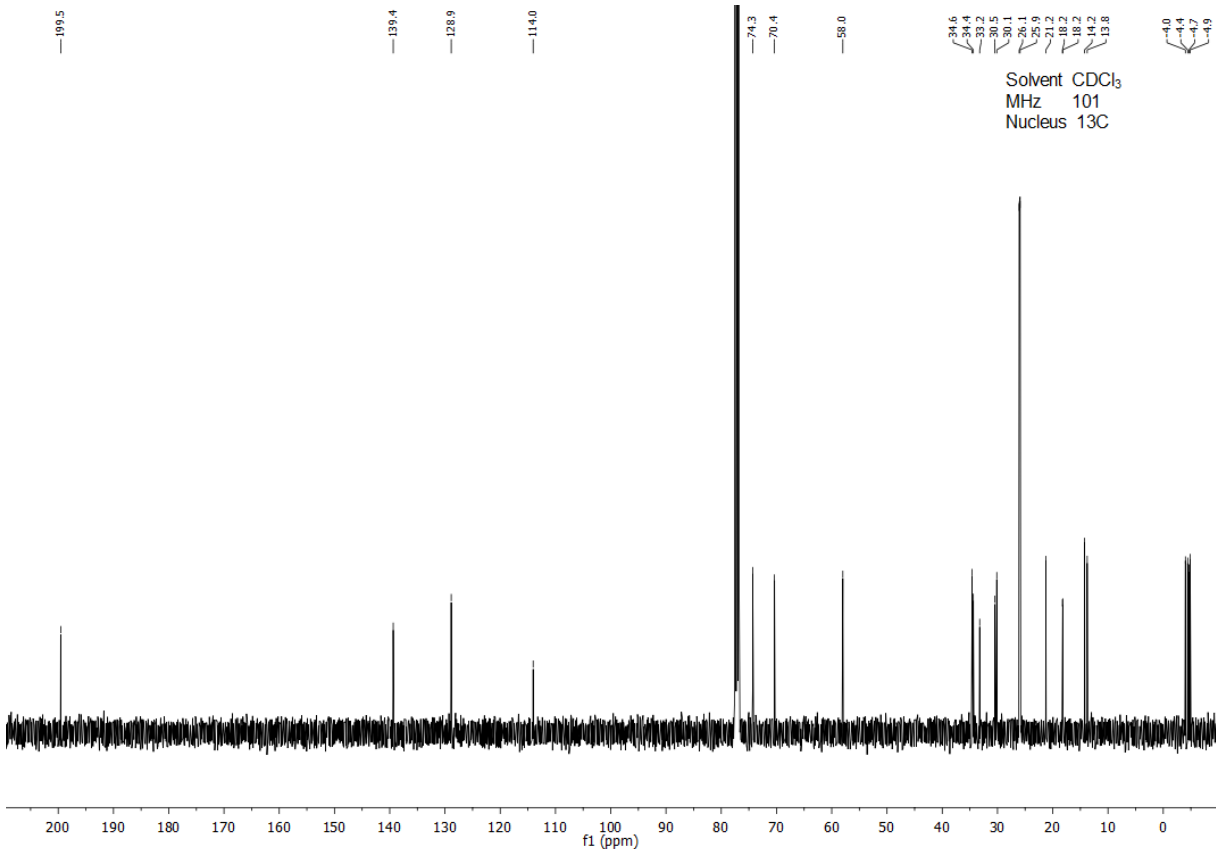
199.5
139.4
128.9
114.0
74.3
70.4
58.0
34.6
34.4
33.2
30.5
30.1
26.1
25.9
21.2
18.2
18.2
14.2
13.8
-4.0
-4.4
-4.7
-4.9
Solvent CDCl3
MHz 101
Nucleus 13C
f1 (ppm)

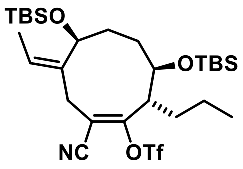
TBSO
OTBS
NC
OTf

28a

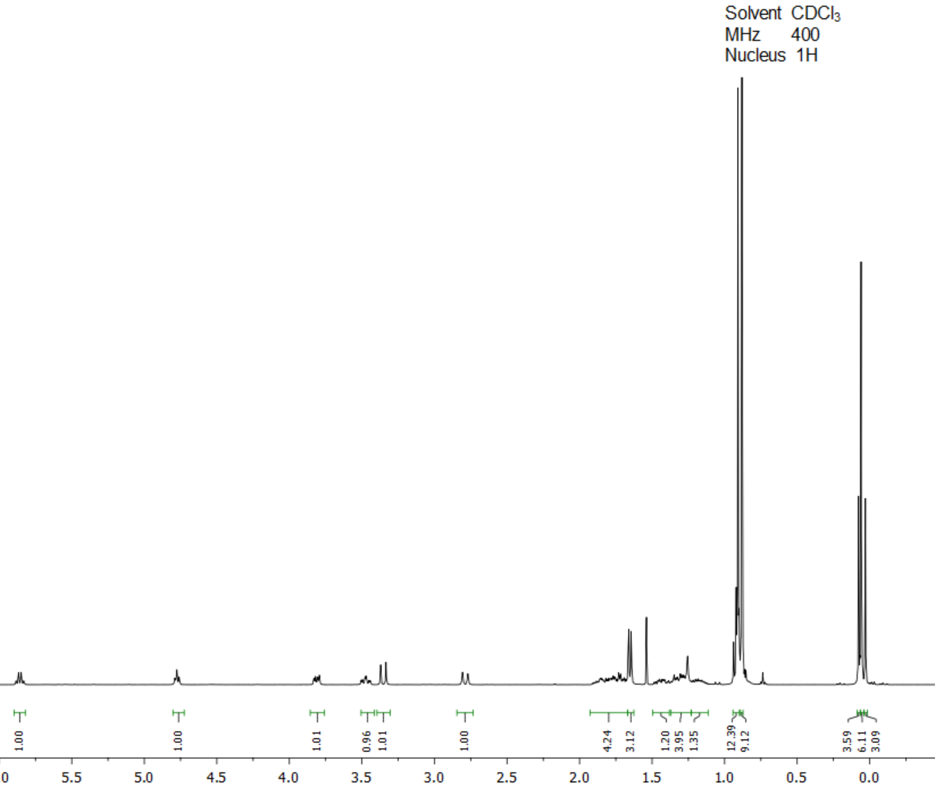
Solvent CDCl3
MHz 400
Nucleus 1H

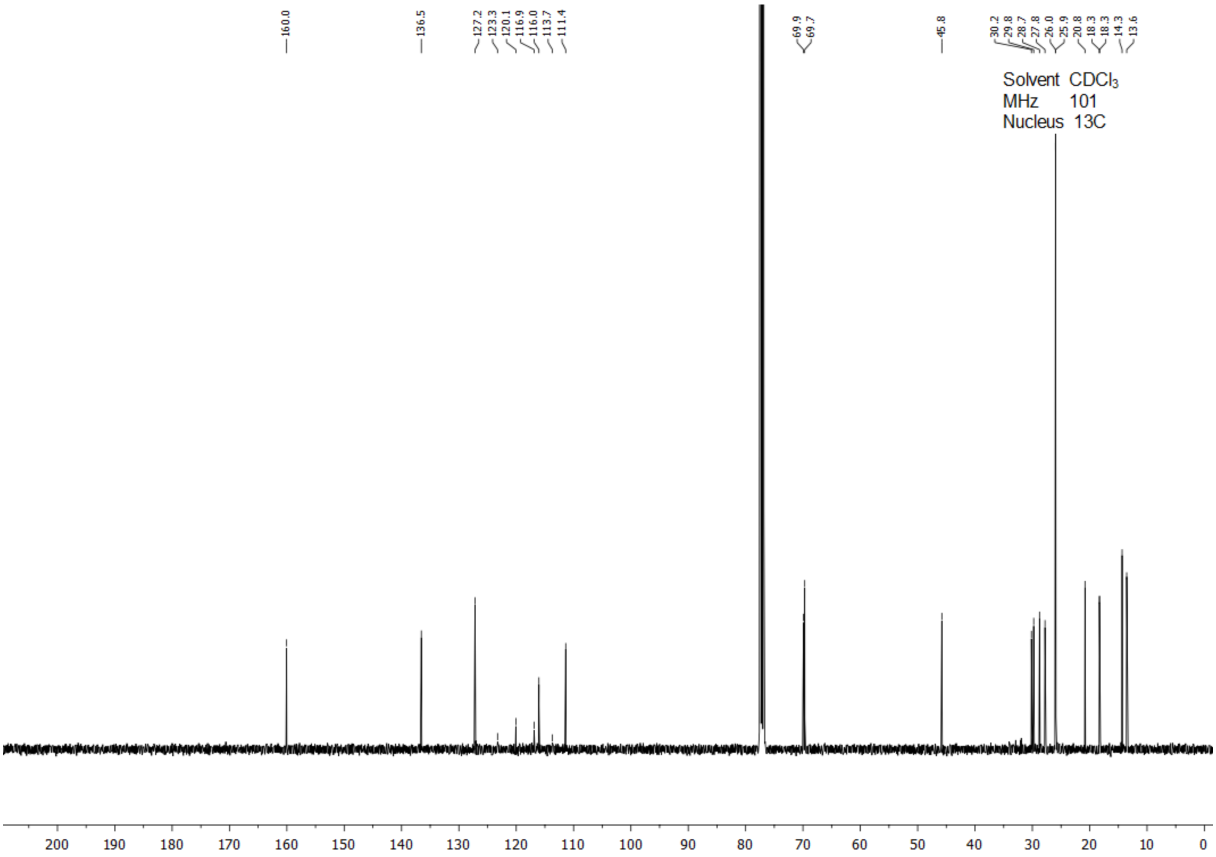
Solvent CDCl3
MHz 101
Nucleus 13C

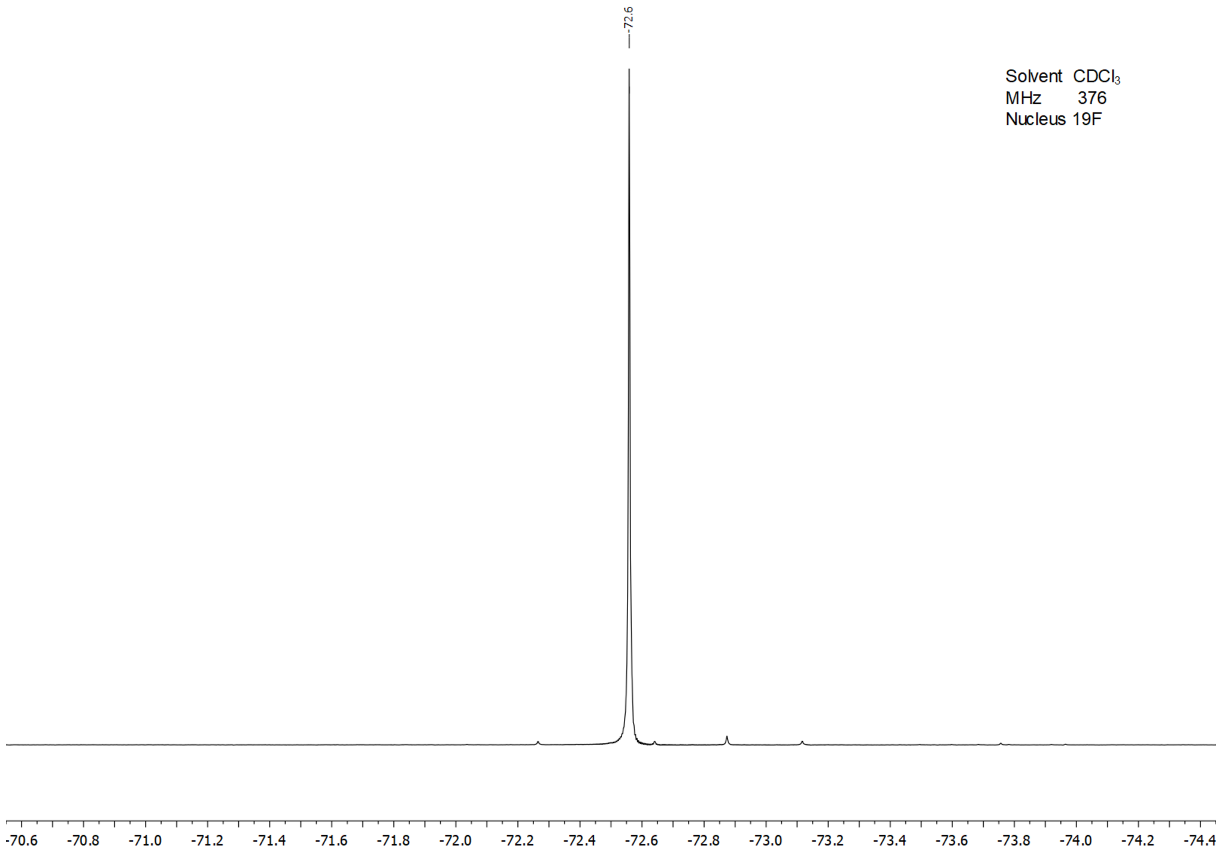
-72.6
Solvent CDCl3
MHz 376
Nucleus 19F
-70.6
-70.8
-71.0
-71.2
-71.4
-71.6
-71.8
-72.0
-72.2
-72.4
-72.6
-72.8
-73.0
-73.2
-73.4
-73.6
-73.8
-74.0
-74.2
-74.4

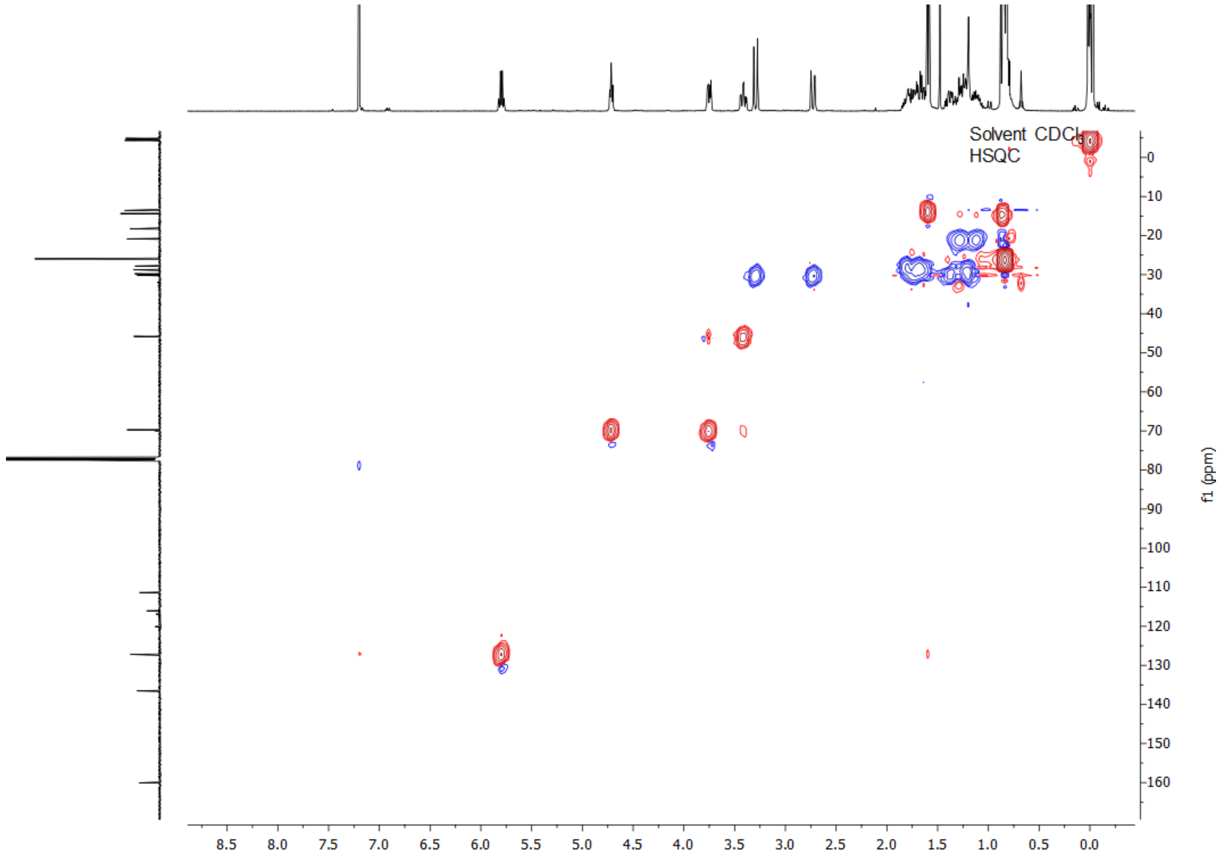
Solvent CDCl3
HSQC
f1 (ppm)
8.5
8.0
7.5
7.0
6.5
6.0
5.5
5.0
4.5
4.0
3.5
3.0
2.5
2.0
1.5
1.0
0.5
0.0

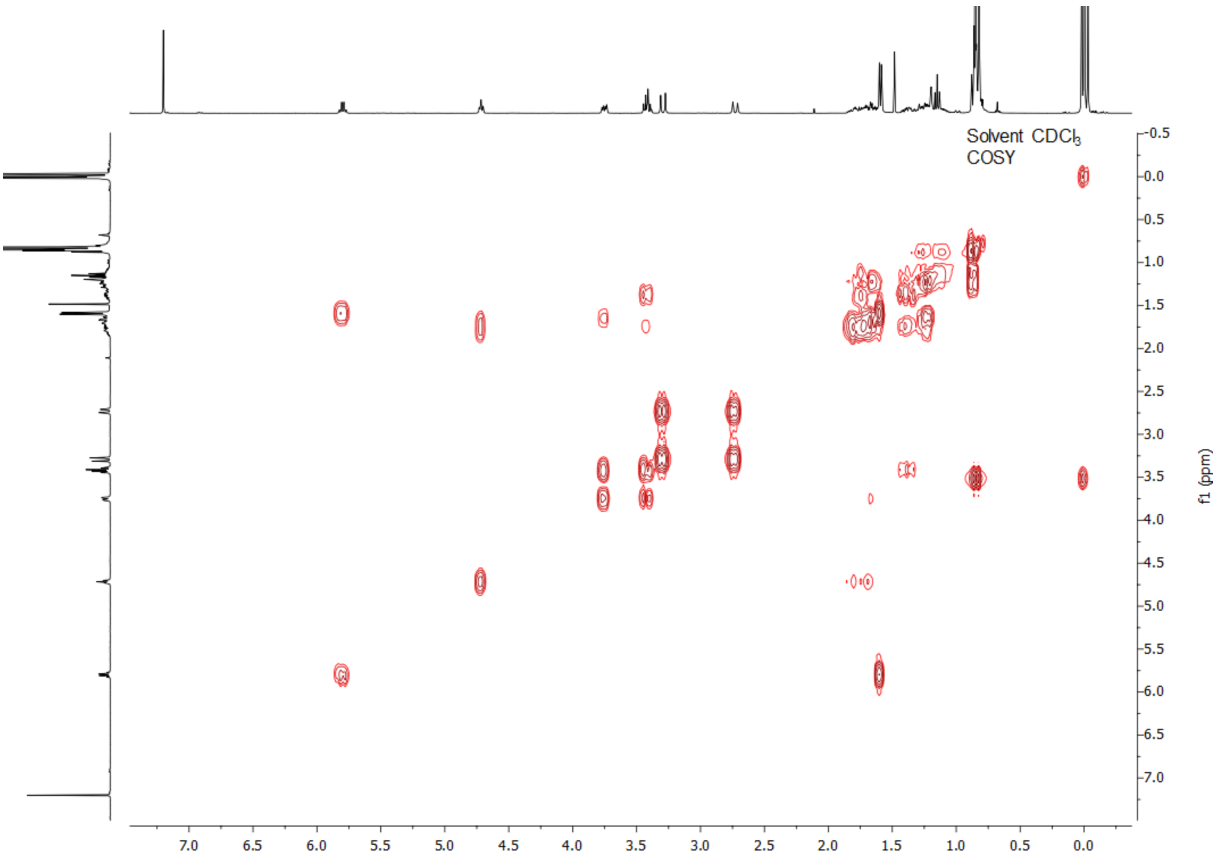
Solvent CDCl3
COSY
f1 (ppm)
-0.5
0.0
0.5
1.0
1.5
2.0
2.5
3.0
3.5
4.0
4.5
5.0
5.5
6.0
6.5
7.0
7.0
6.5
6.0
5.5
5.0
4.5
4.0
3.5
3.0
2.5
2.0
1.5
1.0
0.5
0.0

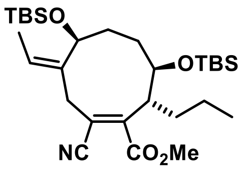
TBSO
OTBS
NC
CO_2Me

29a

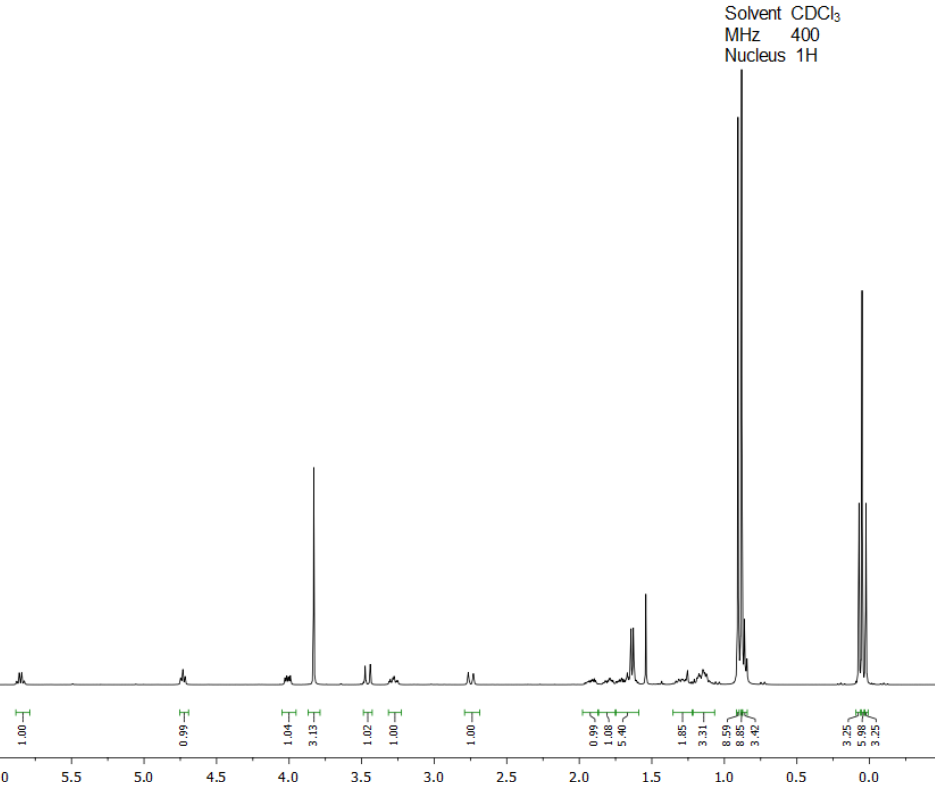
Solvent CDCl$_3$
MHz 400
Nucleus 1H

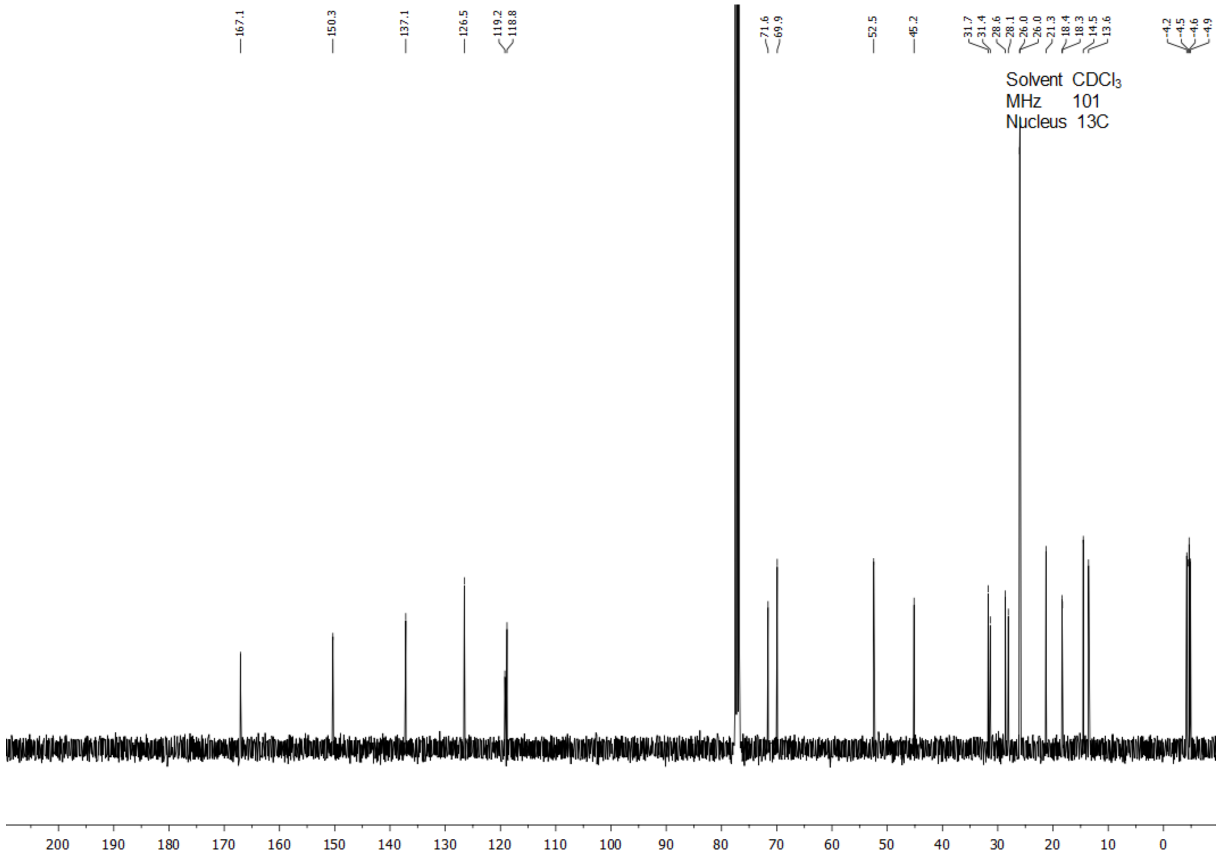
167.1
150.3
137.1
126.5
119.2
118.8
71.6
69.9
52.5
45.2
31.7
31.4
28.6
28.1
26.0
26.0
21.3
18.4
18.3
14.5
13.6
-4.2
-4.5
-4.6
-4.9
Solvent CDCl$_3$
MHz 101
Nucleus 13C

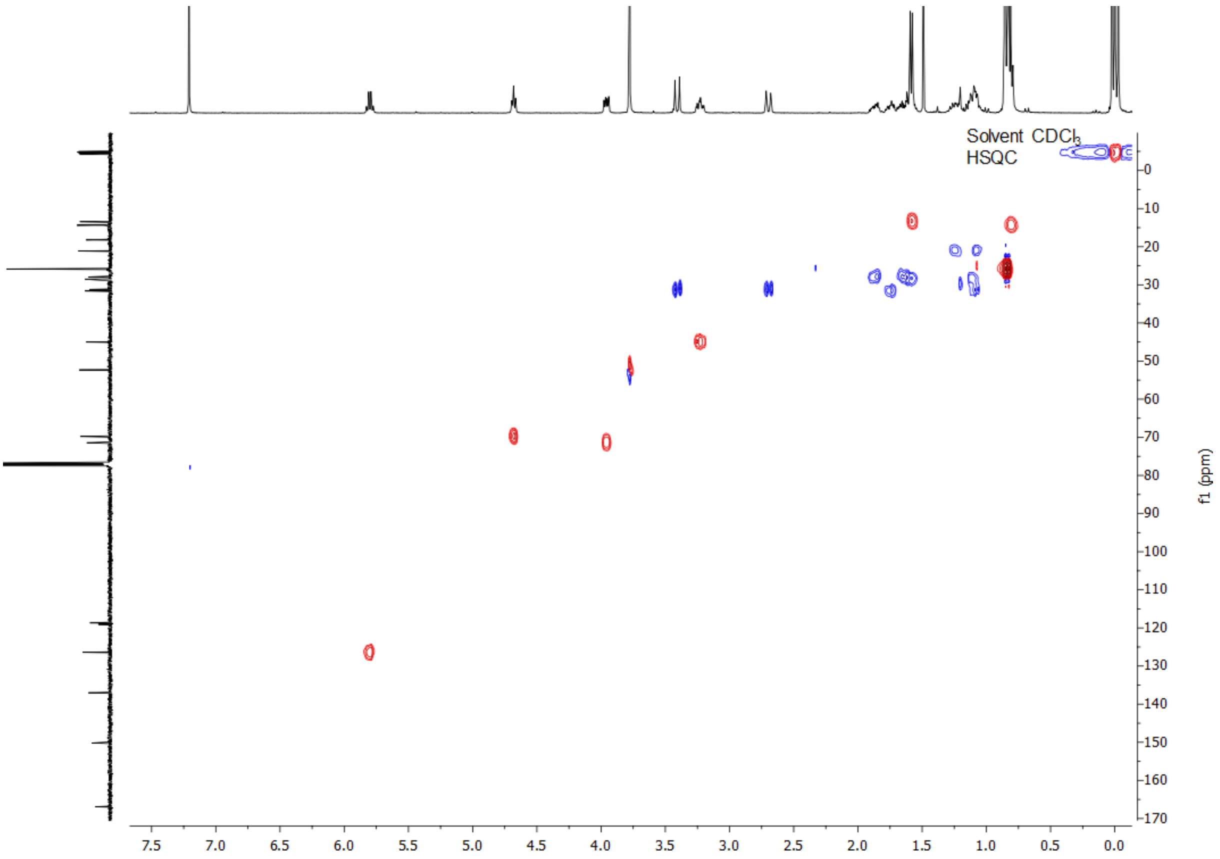
Solvent CDCl3
HSQC
f1 (ppm)

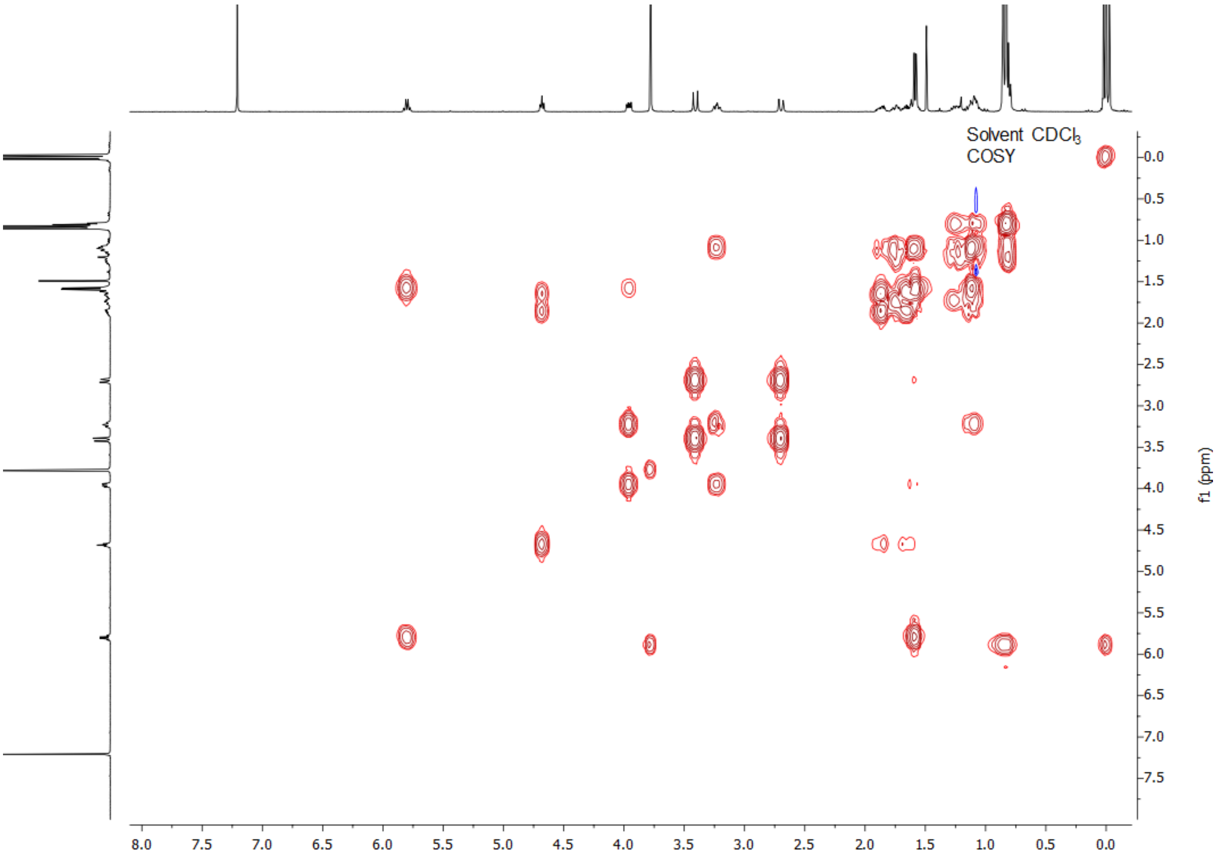
Solvent CDCl3
COSY
f1 (ppm)

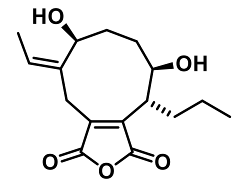
HO
OH
O
O
O

8

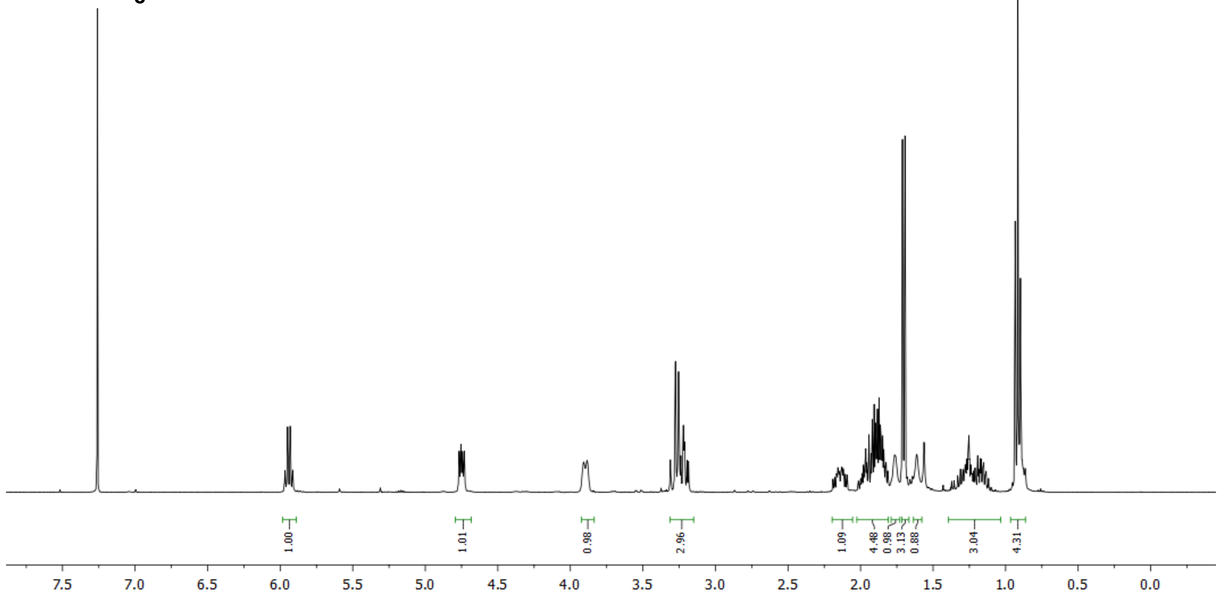
Solvent CDCl3
MHz 400
Nucleus 1H
1.00
1.01
0.98
2.96
1.09
4.48
0.98
3.13
0.88
3.04
4.31
7.5
7.0
6.5
6.0
5.5
5.0
4.5
4.0
3.5
3.0
2.5
2.0
1.5
1.0
0.5
0.0

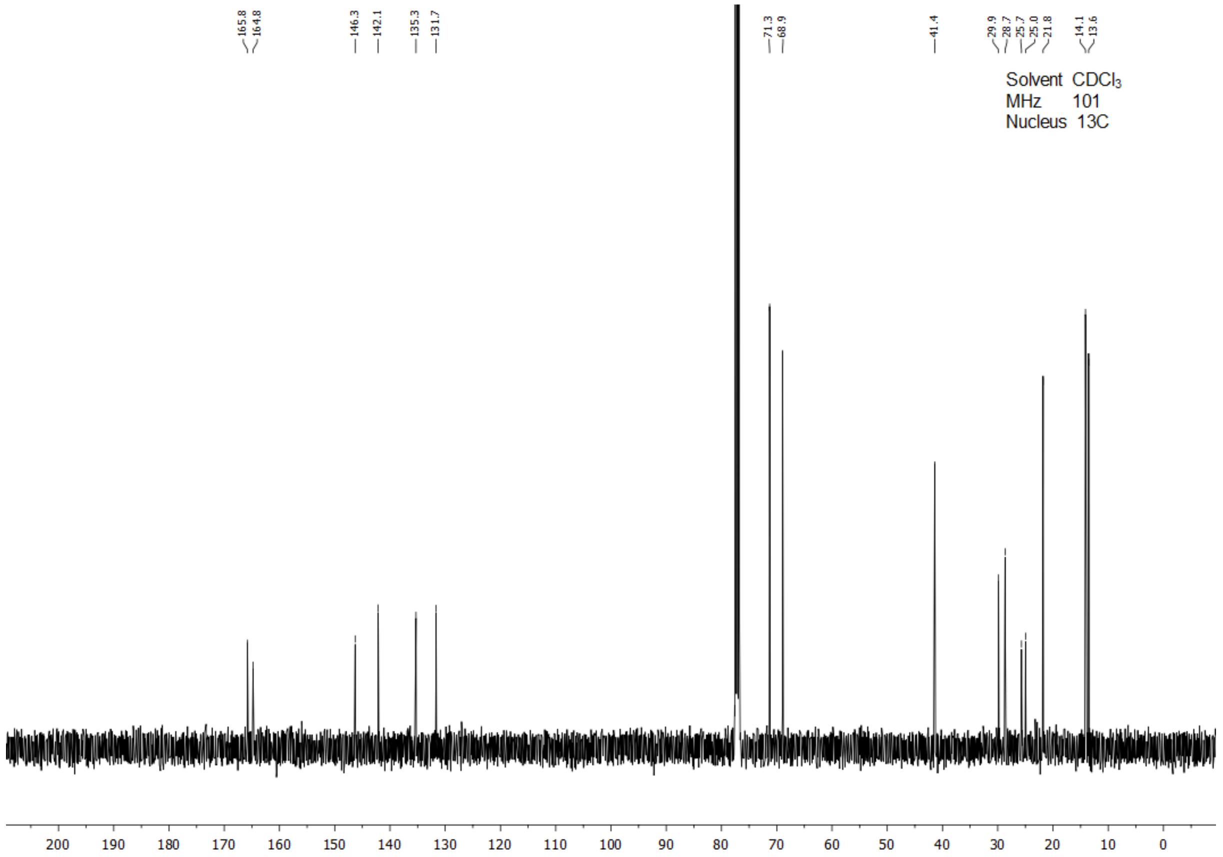
165.8
164.8
146.3
142.1
135.3
131.7
71.3
68.9
41.4
29.9
28.7
25.7
25.0
21.8
14.1
13.6
Solvent CDCl3
MHz 101
Nucleus 13C
200
190
180
170
160
150
140
130
120
110
100
90
80
70
60
50
40
30
20
10
0

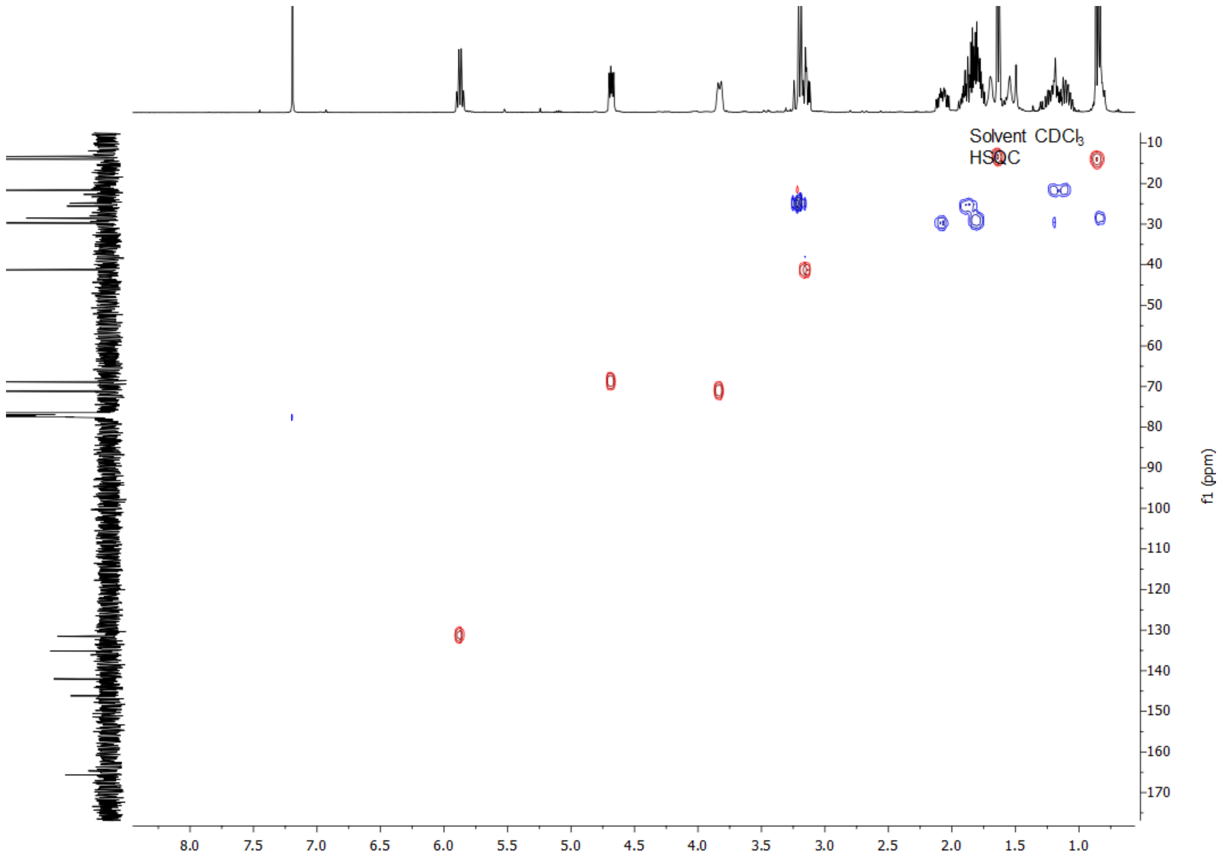
Solvent CDCl3
HSQC
f1 (ppm)

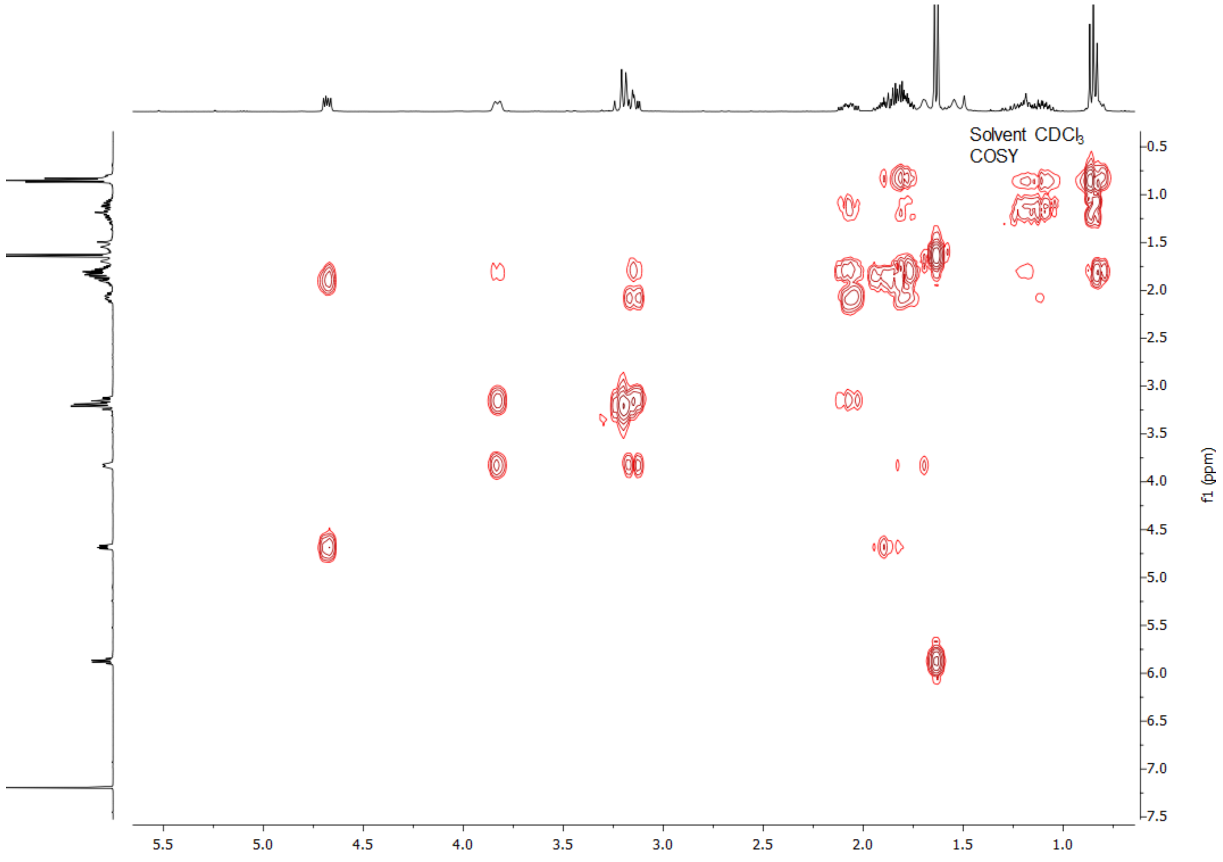
Solvent CDCl3
COSY
f1 (ppm)

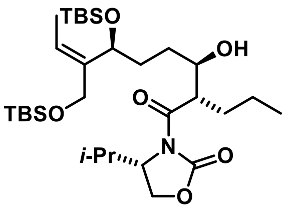
TBSO
OH
TBSO
O
N
i-Pr
O
O

18b

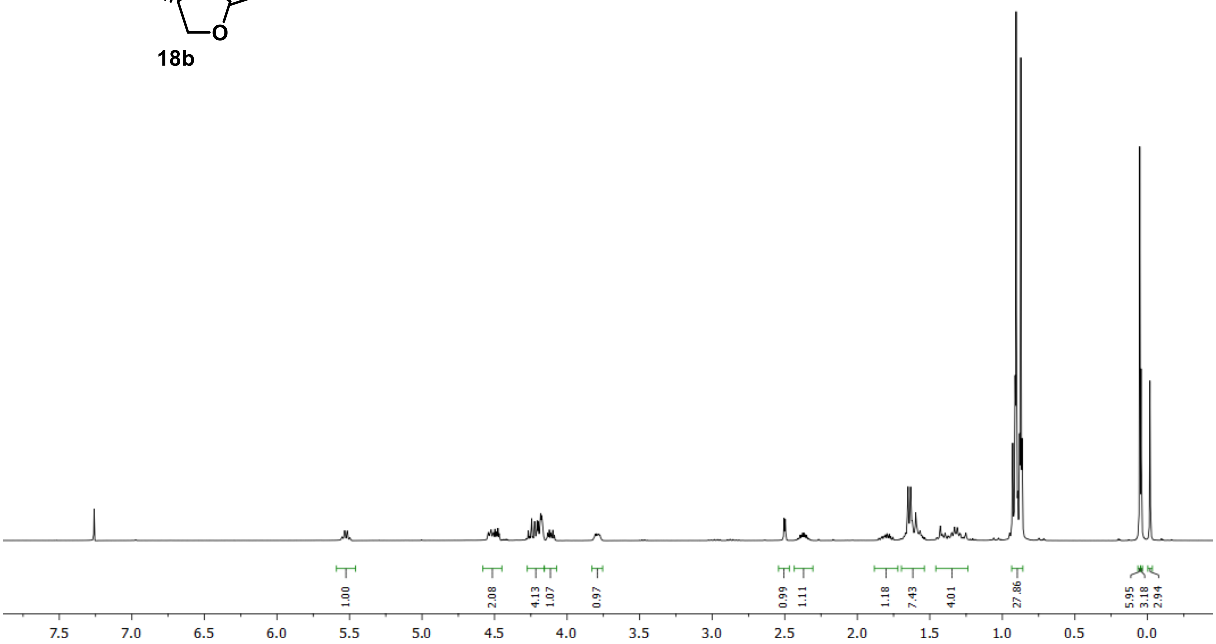
Solvent CDCl3
MHz 400
Nucleus 1H
1.00
2.08
4.13
1.07
0.97
0.99
1.11
1.18
7.43
4.01
27.86
5.95
3.18
2.94

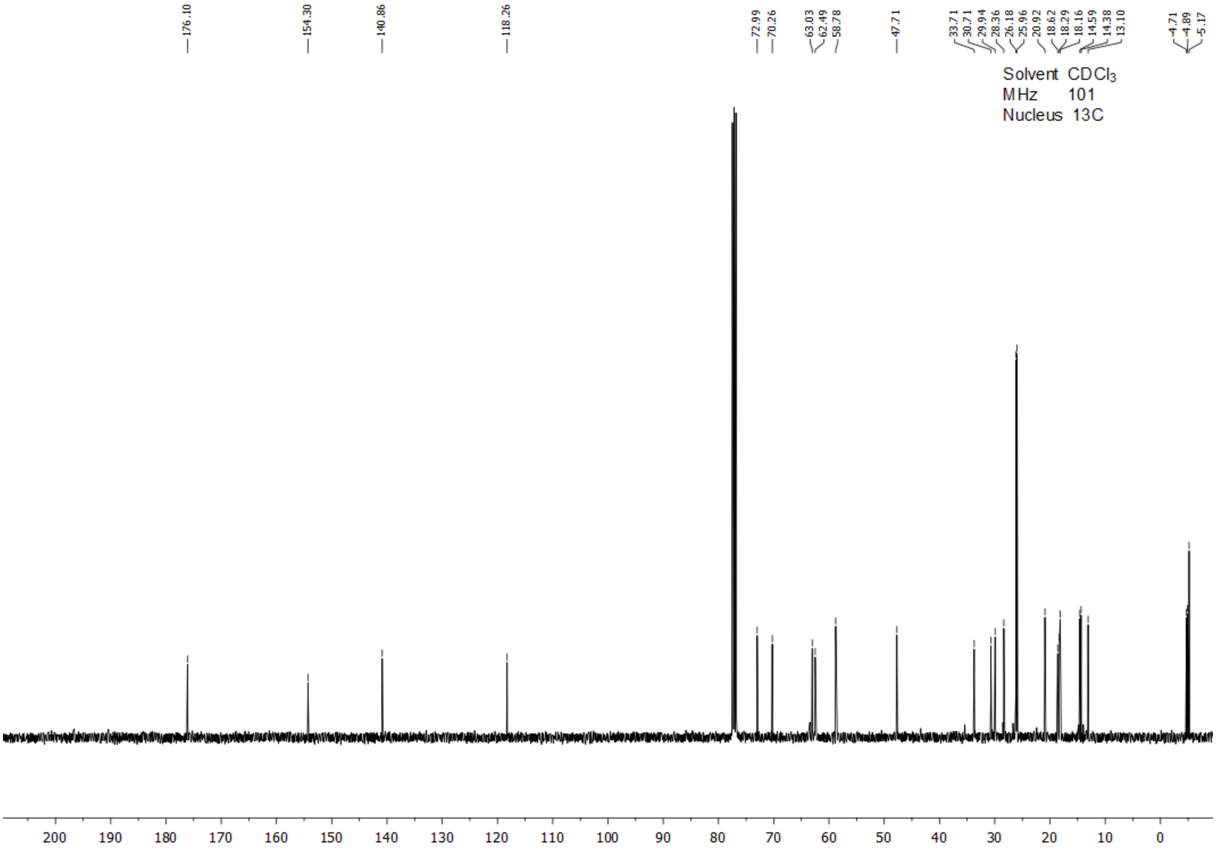
176.10
154.30
140.86
118.26
72.99
70.26
63.03
62.49
58.78
47.71
33.71
30.71
29.94
28.36
26.18
25.96
20.92
18.62
18.29
18.16
14.59
14.38
13.10
-4.71
-4.89
-5.17
Solvent CDCl3
MHz 101
Nucleus 13C

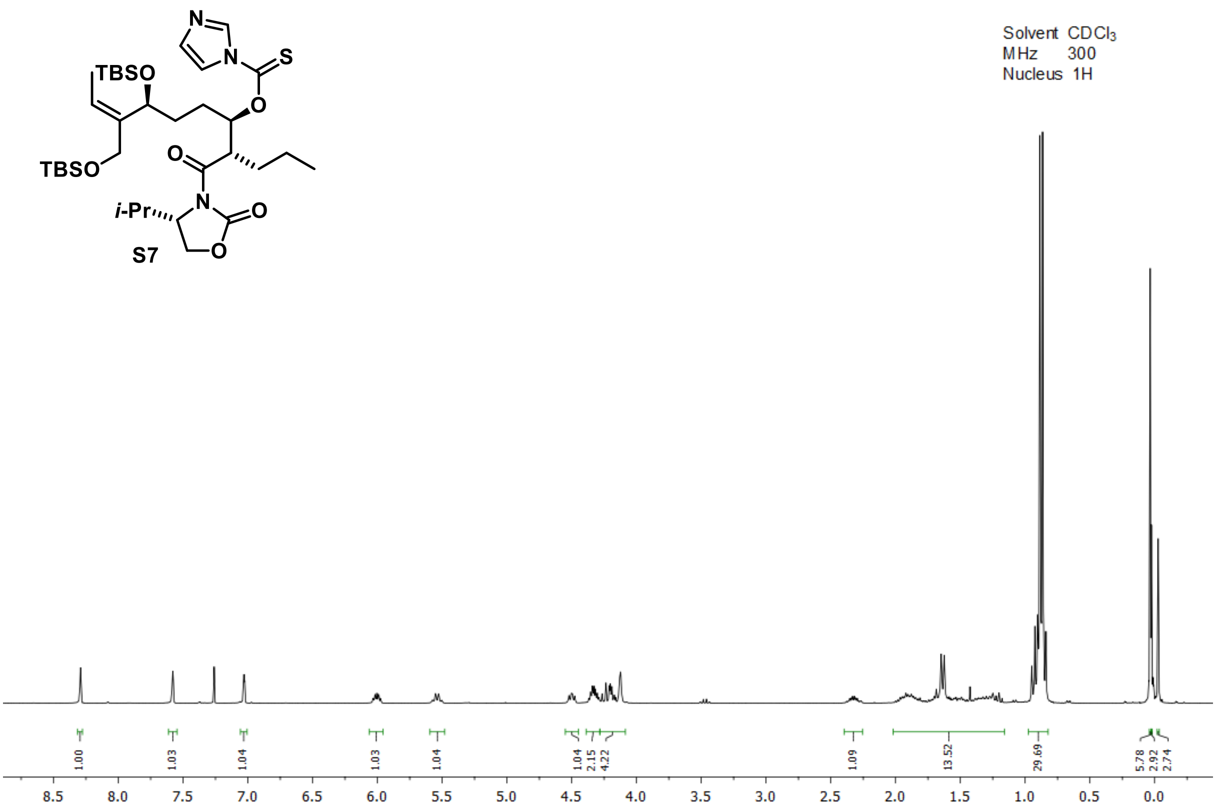

TBSO
TBSO
N
N
S
O
O
N
O
O
i-Pr
S7
Solvent CDCl3
MHz 300
Nucleus 1H
1.00
1.03
1.04
1.03
1.04
1.04
2.15
4.22
1.09
13.52
29.69
5.78
2.92
2.74
8.5 8.0 7.5 7.0 6.5 6.0 5.5 5.0 4.5 4.0 3.5 3.0 2.5 2.0 1.5 1.0 0.5 0.0

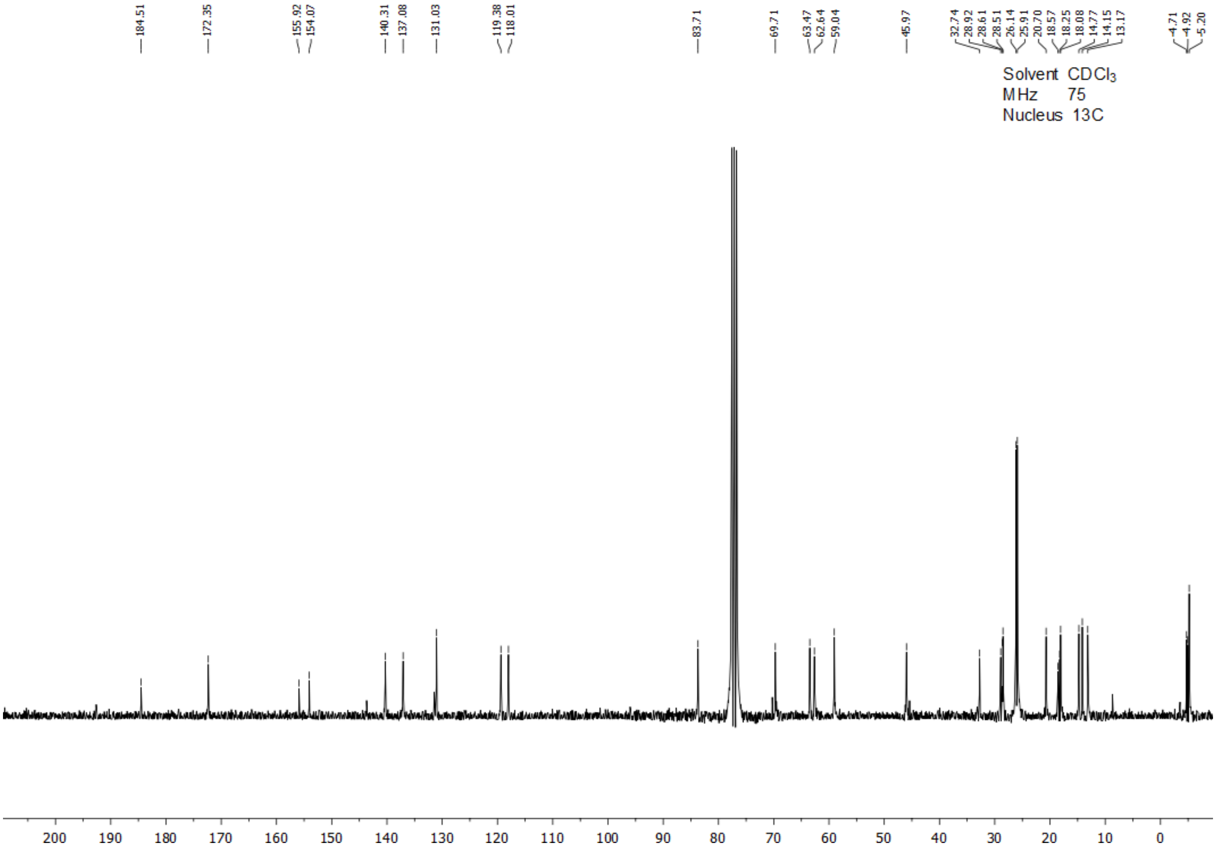

184.51
172.35
155.92
154.07
140.31
137.08
131.03
119.38
118.01
83.71
69.71
63.47
62.64
59.04
45.97
32.74
28.92
28.61
28.51
26.14
25.91
20.70
18.57
18.25
18.08
14.77
14.15
13.17
-4.71
-4.92
-5.20
Solvent CDCl3
MHz 75
Nucleus 13C
200 190 180 170 160 150 140 130 120 110 100 90 80 70 60 50 40 30 20 10 0

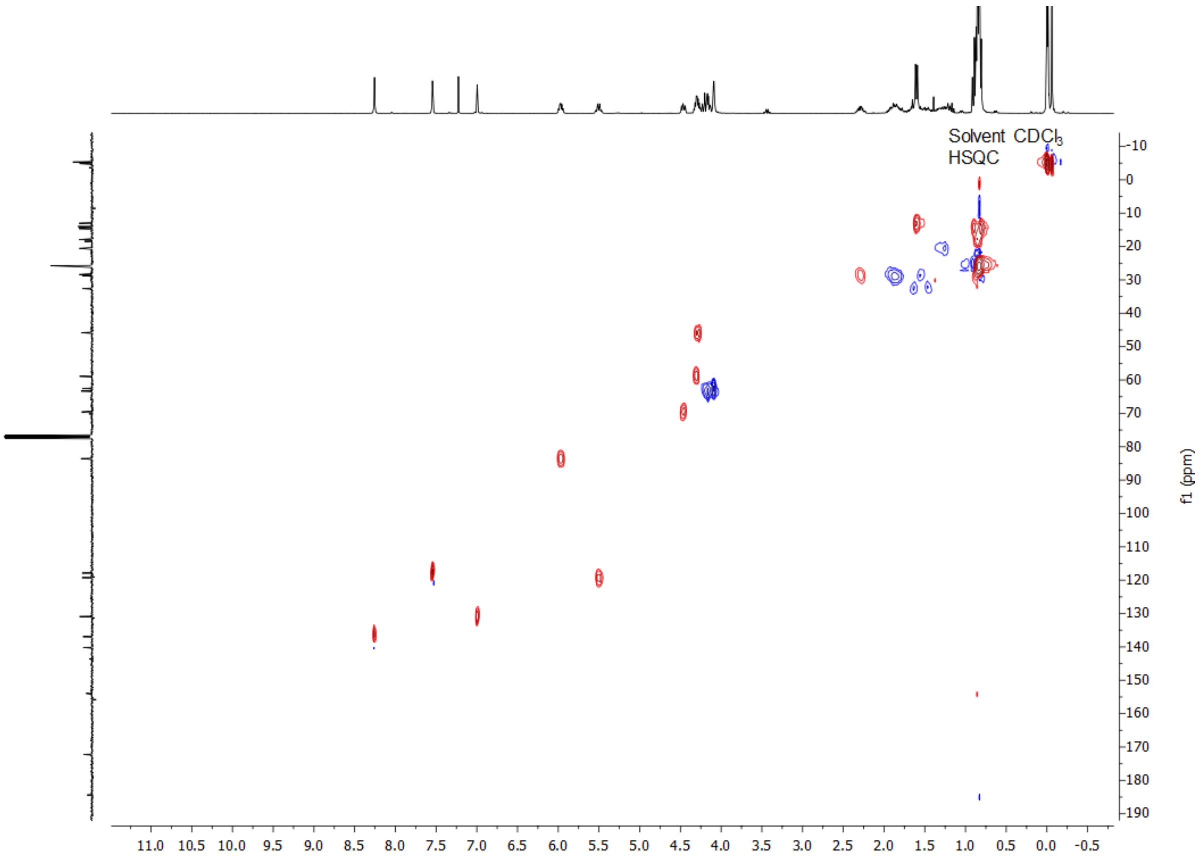
Solvent CDCl3
HSQC
f1 (ppm)

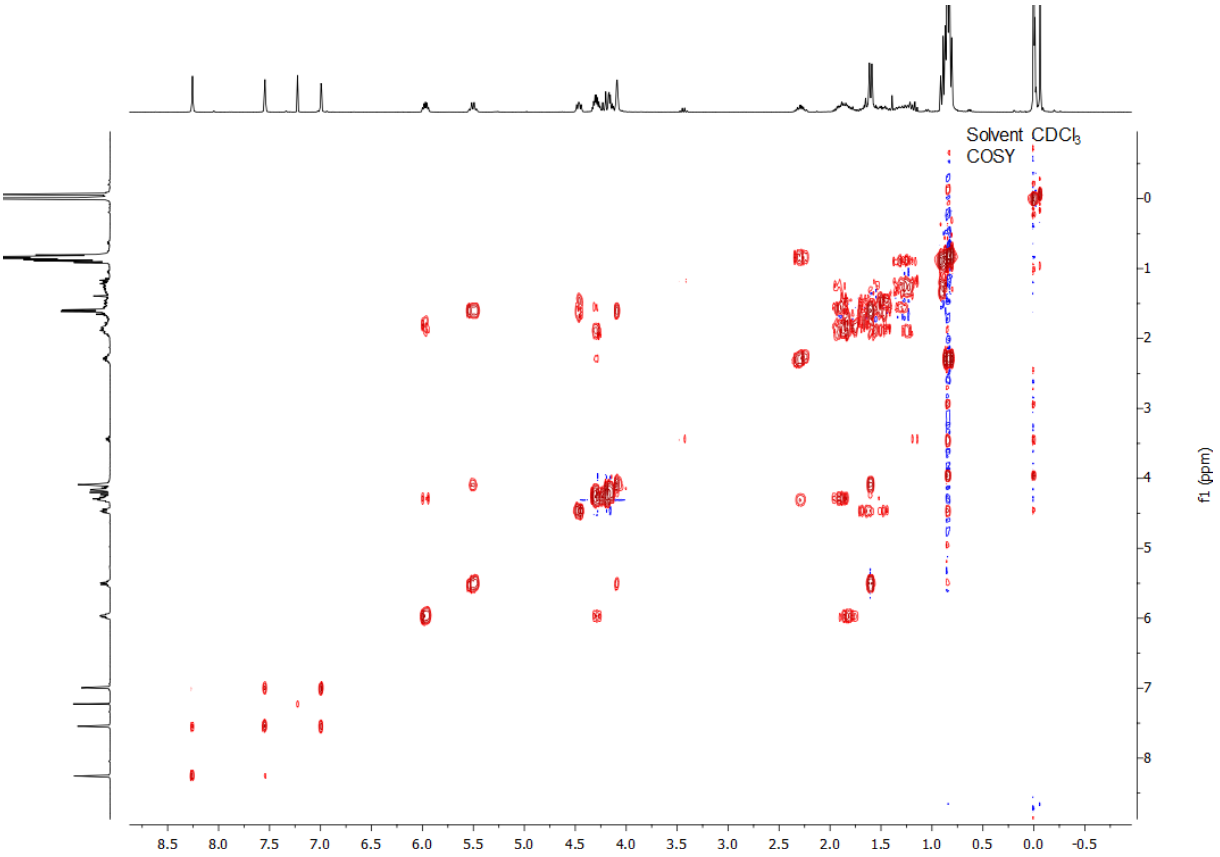
Solvent CDCl3
COSY
f1 (ppm)

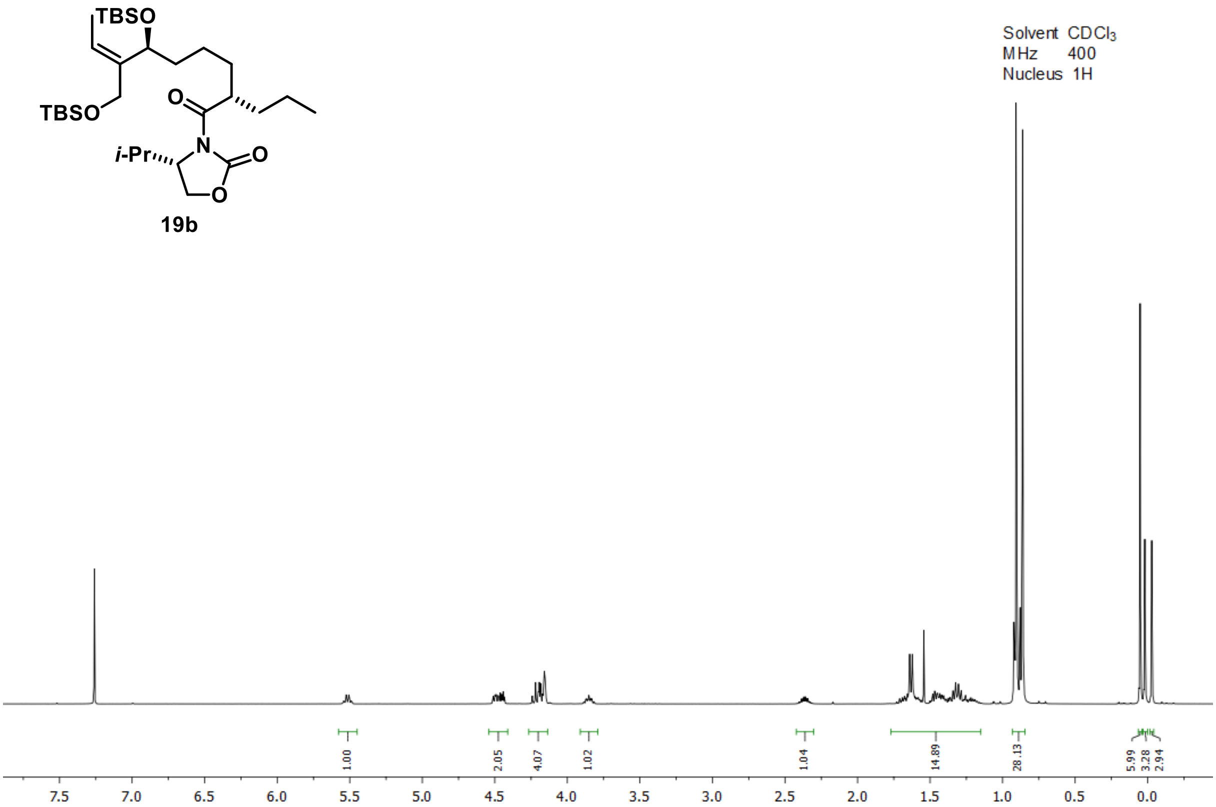
TBSO
TBSO
O
N
O
i-Pr
O
19b
Solvent CDCl3
MHz 400
Nucleus 1H
1.00
2.05
4.07
1.02
1.04
14.89
28.13
5.99
3.28
2.94
7.5
7.0
6.5
6.0
5.5
5.0
4.5
4.0
3.5
3.0
2.5
2.0
1.5
1.0
0.5
0.0

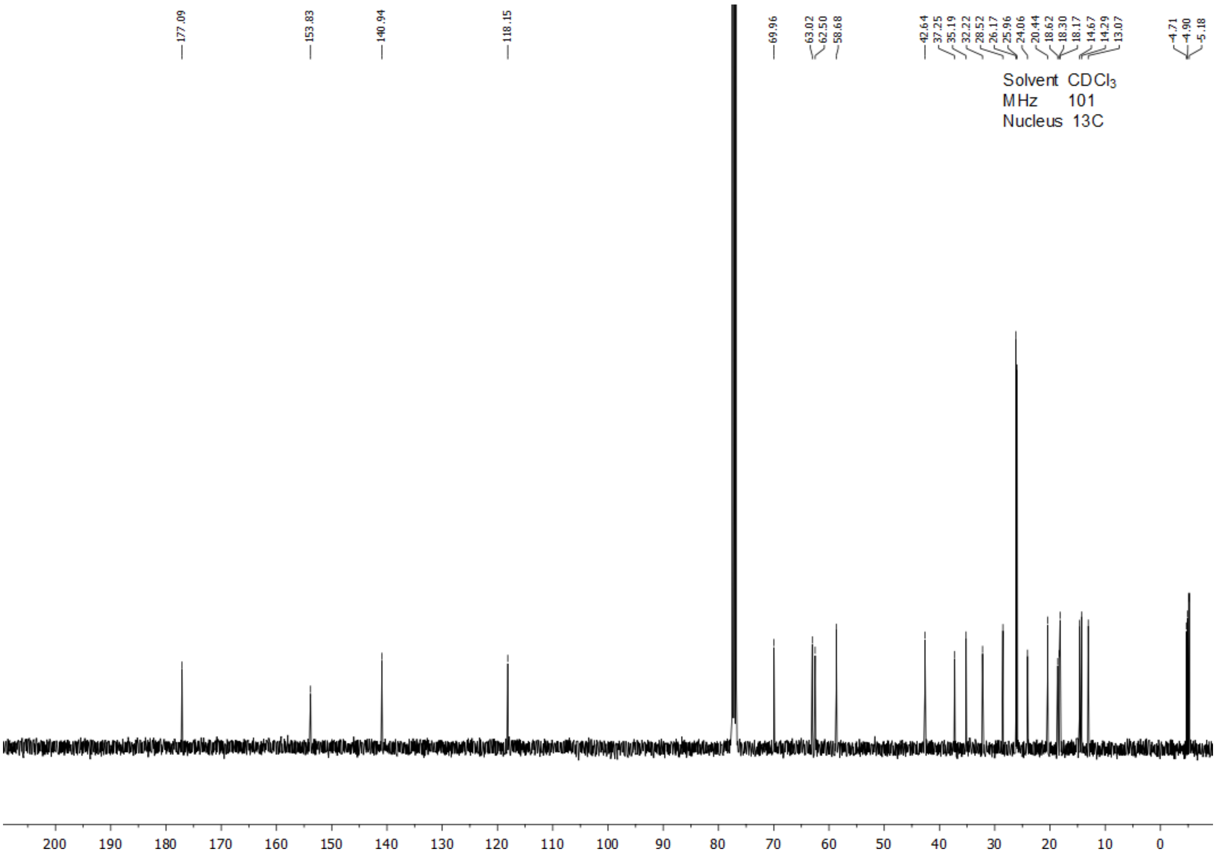
177.09
153.83
140.94
118.15
69.96
63.02
62.50
58.68
42.64
37.25
35.19
32.22
28.52
26.17
25.96
24.06
20.44
18.62
18.30
18.17
14.67
14.29
13.07
-4.71
-4.90
-5.18
Solvent CDCl3
MHz 101
Nucleus 13C
200
190
180
170
160
150
140
130
120
110
100
90
80
70
60
50
40
30
20
10
0

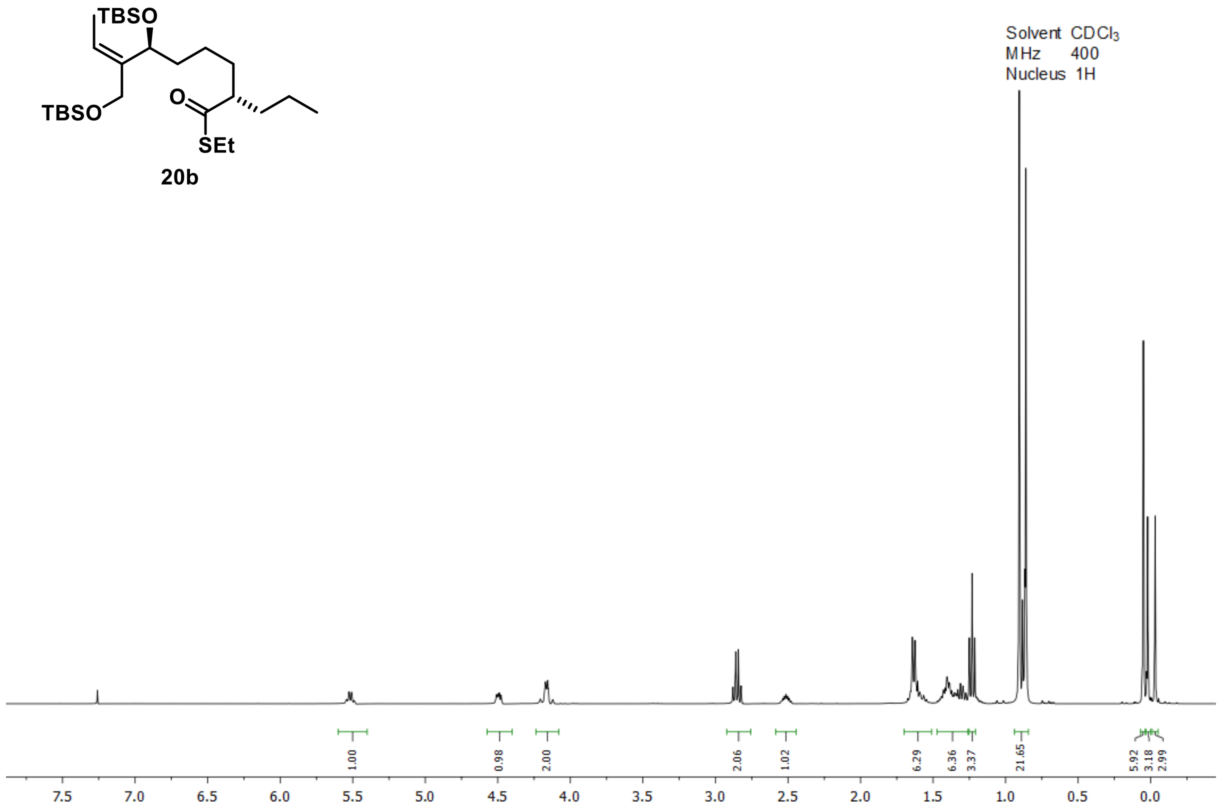
TBSO
TBSO
O
SEt
20b
Solvent CDCl3
MHz 400
Nucleus 1H

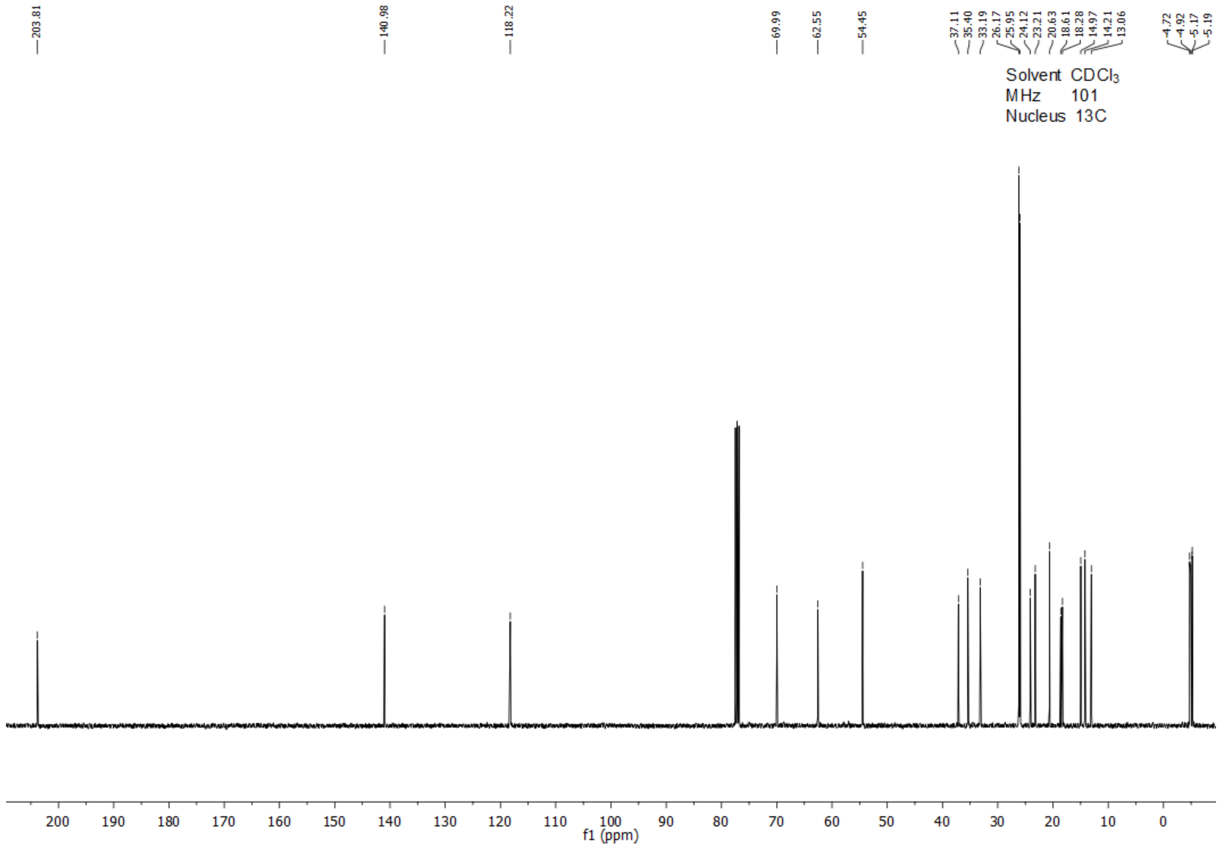
Solvent CDCl3
MHz 101
Nucleus 13C
f1 (ppm)

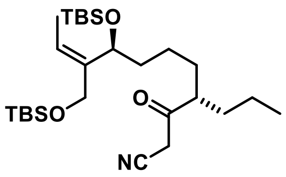
TBSO
TBSO
O
NC

21b

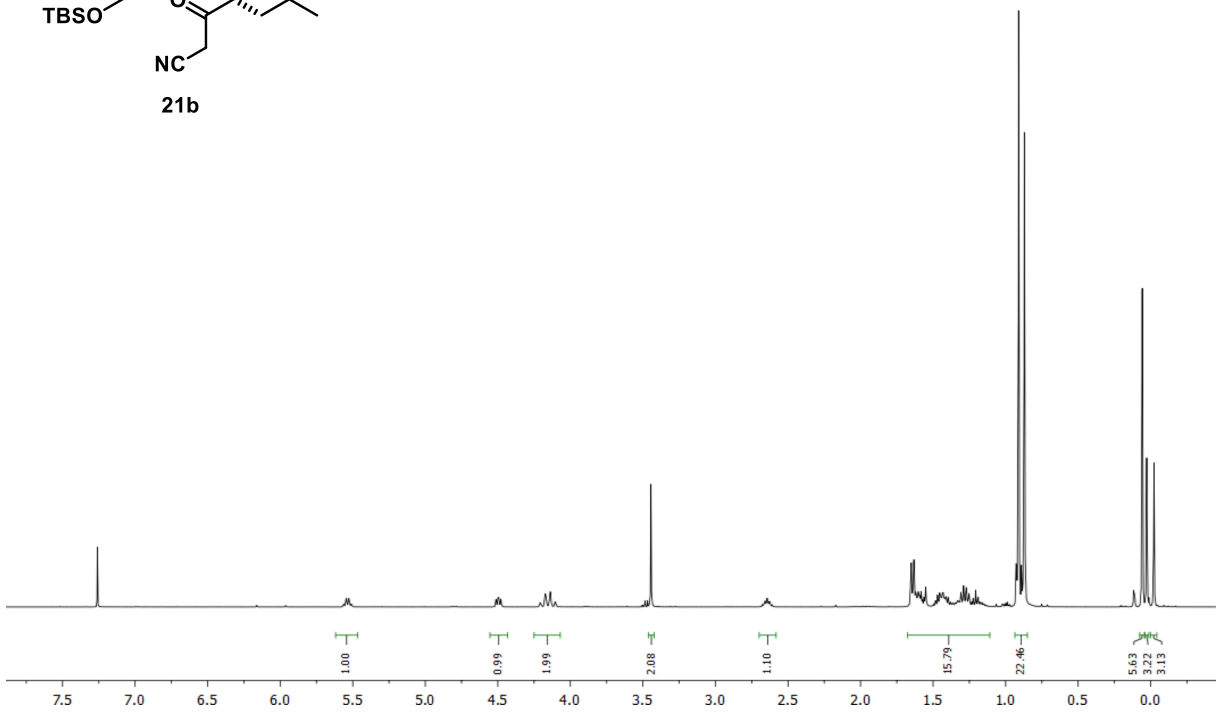
Solvent CDCl3
MHz 400
Nucleus 1H
1.00
0.99
1.99
2.08
1.10
15.79
22.46
5.63
3.22
3.13
7.5 7.0 6.5 6.0 5.5 5.0 4.5 4.0 3.5 3.0 2.5 2.0 1.5 1.0 0.5 0.0

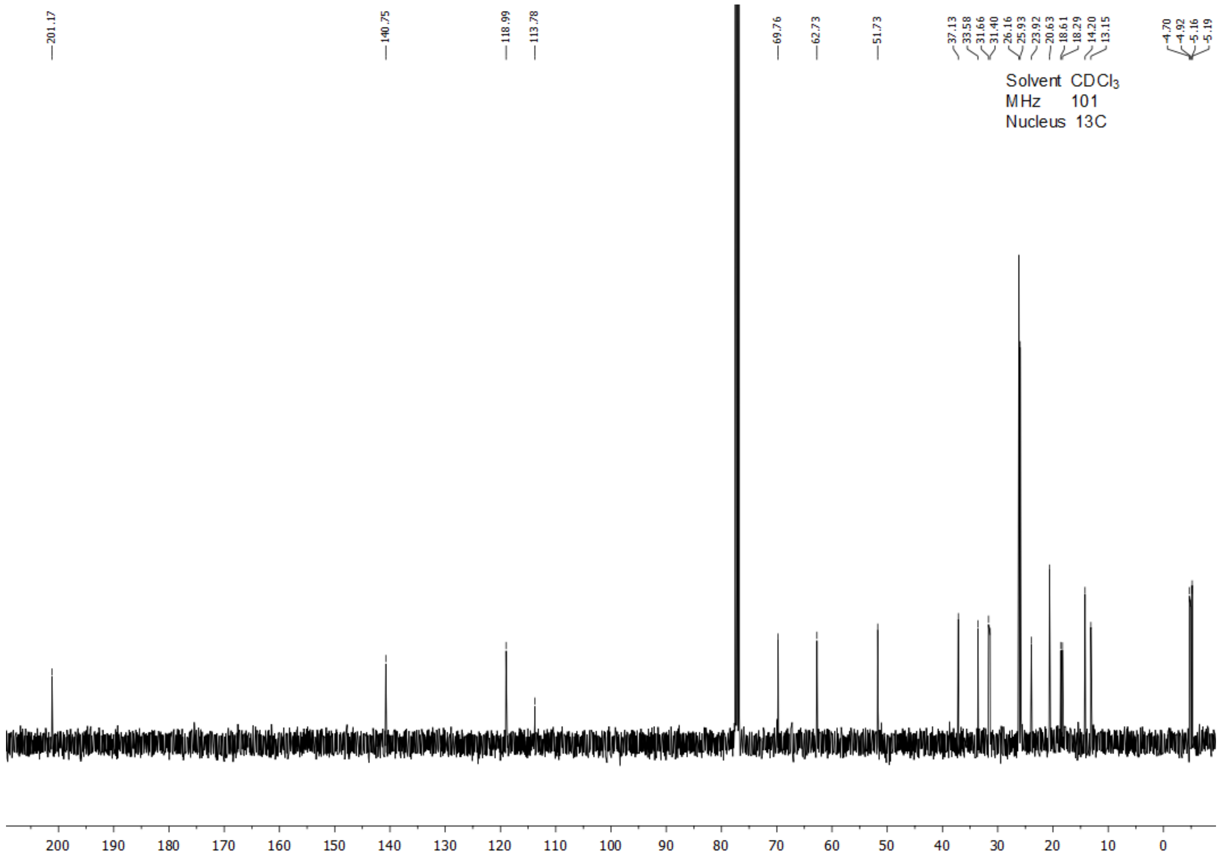
201.17
140.75
118.99
113.78
69.76
62.73
51.73
37.13
33.58
31.66
31.40
26.16
25.93
23.92
20.63
18.61
18.29
14.20
13.15
-4.70
-4.92
-5.16
-5.19
Solvent CDCl3
MHz 101
Nucleus 13C
200 190 180 170 160 150 140 130 120 110 100 90 80 70 60 50 40 30 20 10 0

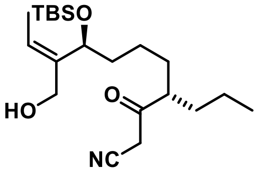
TBSO
HO
O
NC

25b

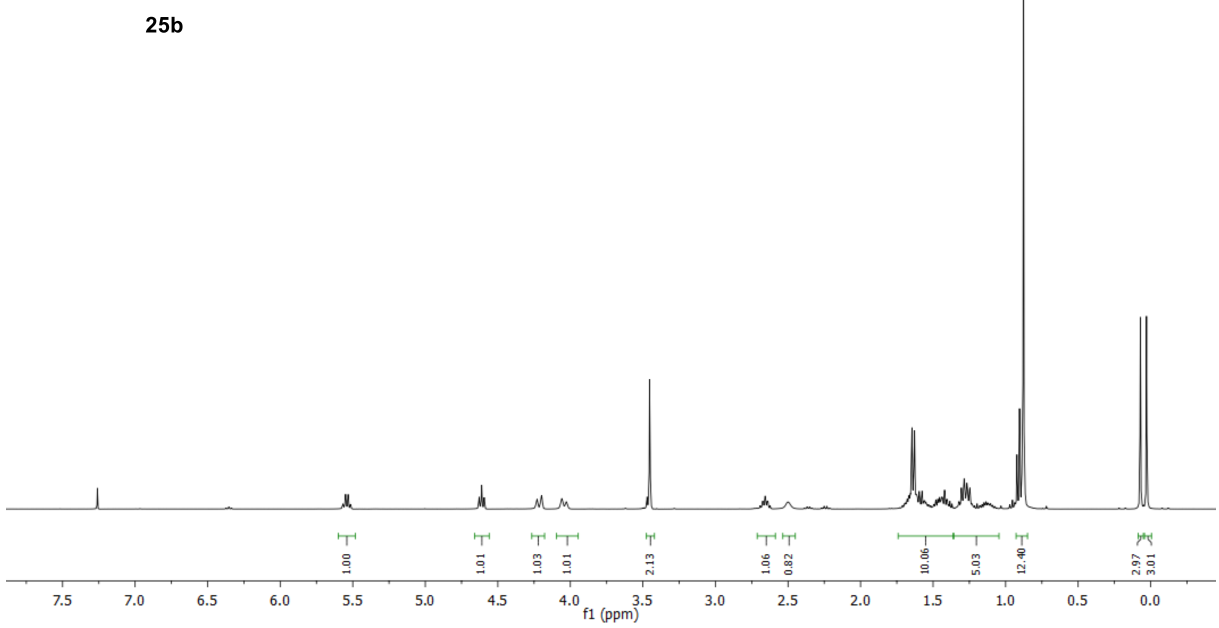
Solvent CDCl3
MHz 400
Nucleus 1H
1.00
1.01
1.03
1.01
2.13
1.06
0.82
10.06
5.03
12.40
2.97
3.01
f1 (ppm)

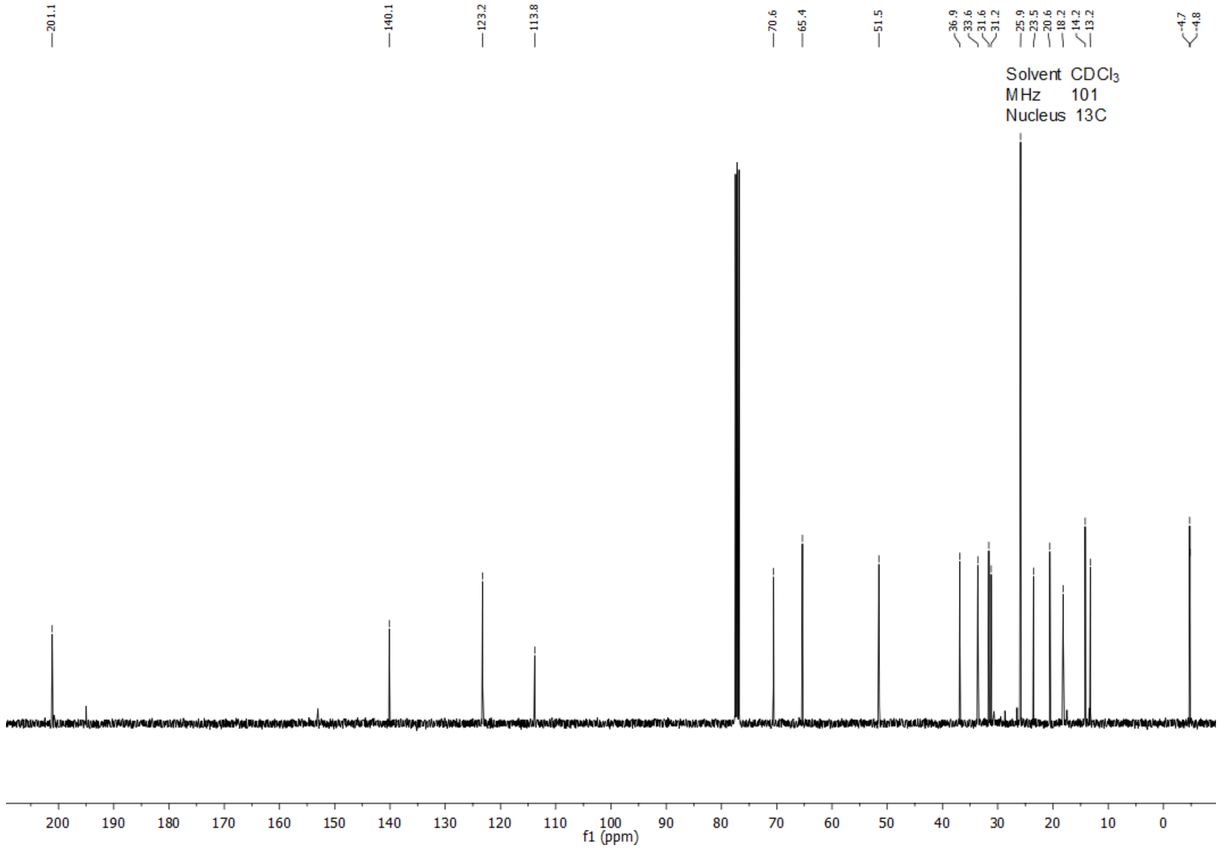
201.1
140.1
123.2
113.8
70.6
65.4
51.5
36.9
33.6
31.6
31.2
25.9
23.5
20.6
18.2
14.2
13.2
-4.7
-4.8
Solvent CDCl3
MHz 101
Nucleus 13C
f1 (ppm)

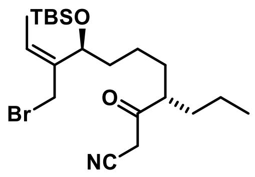
TBSO
Br
O
NC

26b

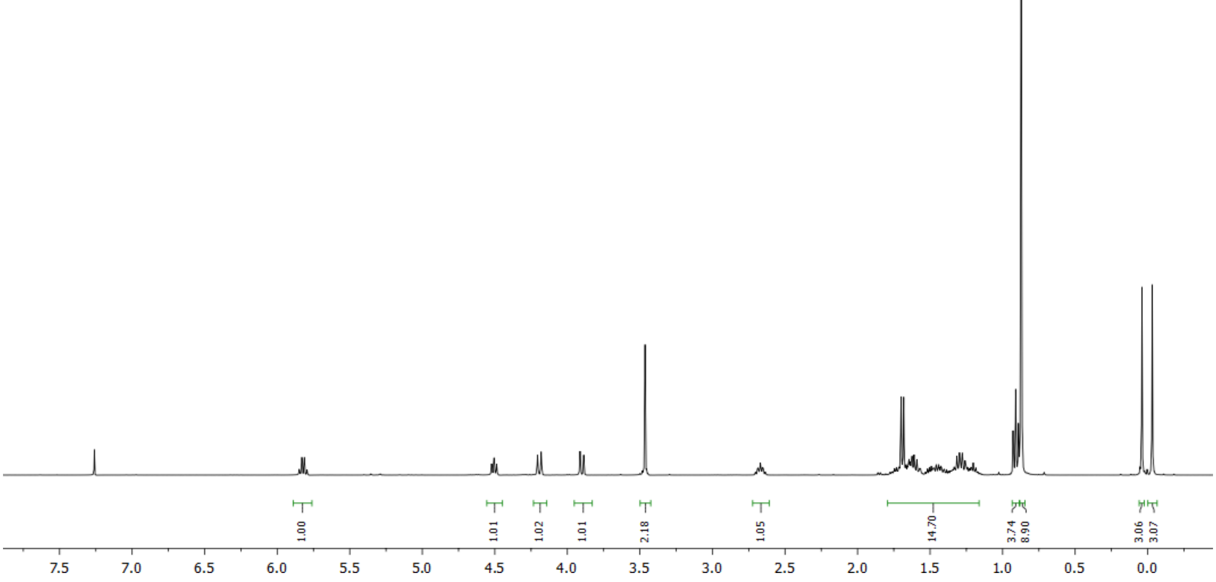
Solvent CDCl3
MHz 400
Nucleus 1H
1.00
1.01
1.02
1.01
2.18
1.05
14.70
3.74
8.90
3.06
3.07
7.5 7.0 6.5 6.0 5.5 5.0 4.5 4.0 3.5 3.0 2.5 2.0 1.5 1.0 0.5 0.0

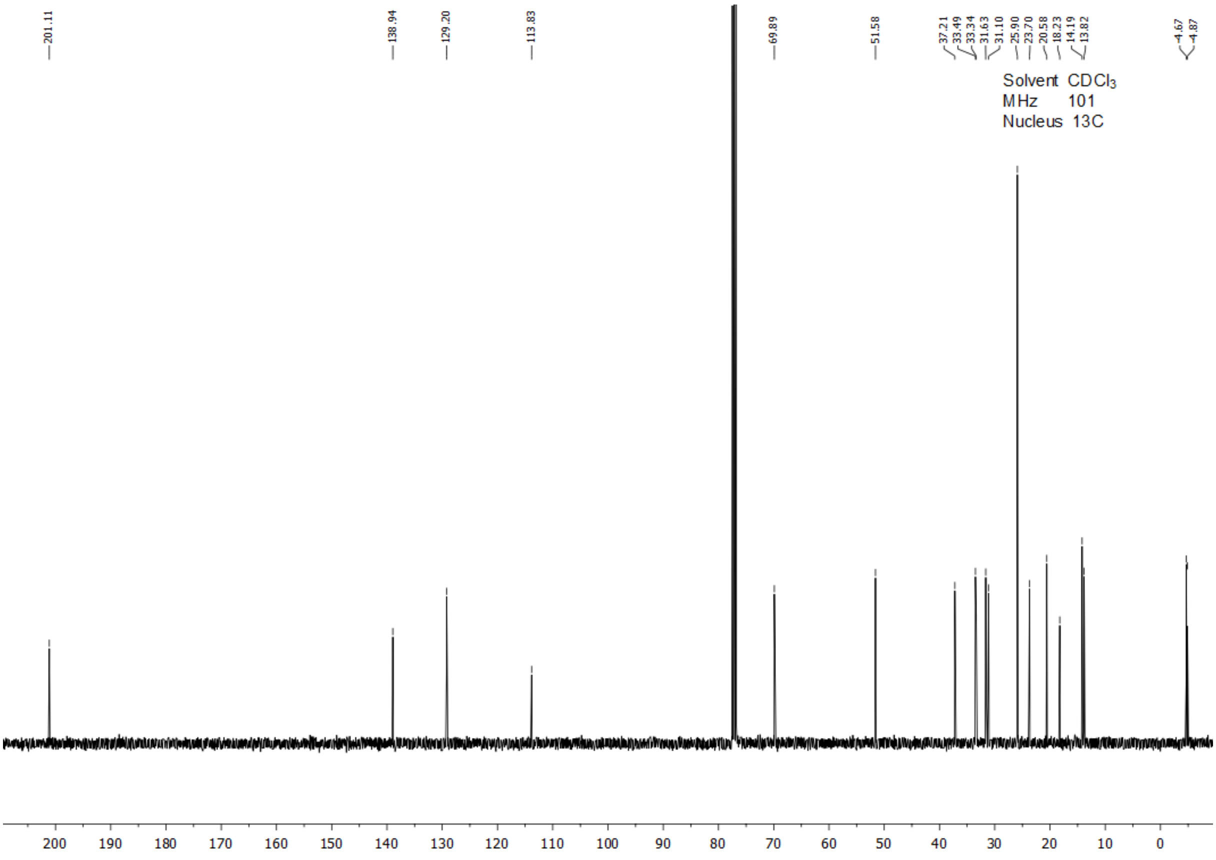
201.11
138.94
129.20
113.83
69.89
51.58
37.21
33.49
33.34
31.63
31.10
25.90
23.70
20.58
18.23
14.19
13.82
-4.67
-4.87
Solvent CDCl3
MHz 101
Nucleus 13C
200 190 180 170 160 150 140 130 120 110 100 90 80 70 60 50 40 30 20 10 0

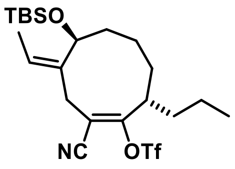
TBSO
NC
OTf

28b

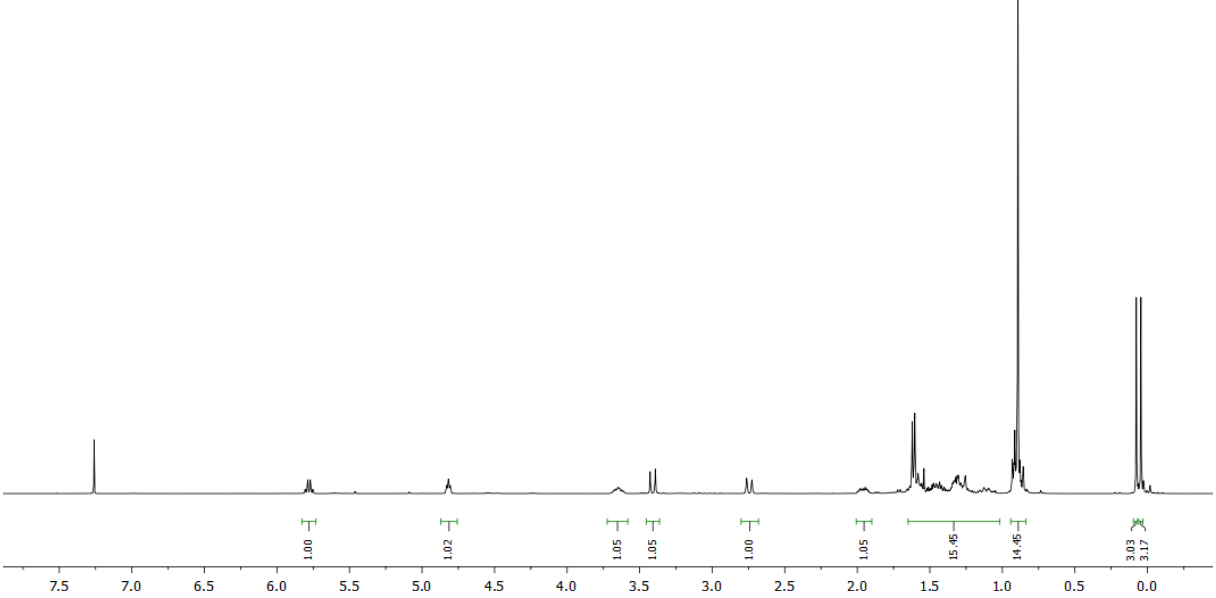
Solvent CDCl3
MHz 400
Nucleus 1H

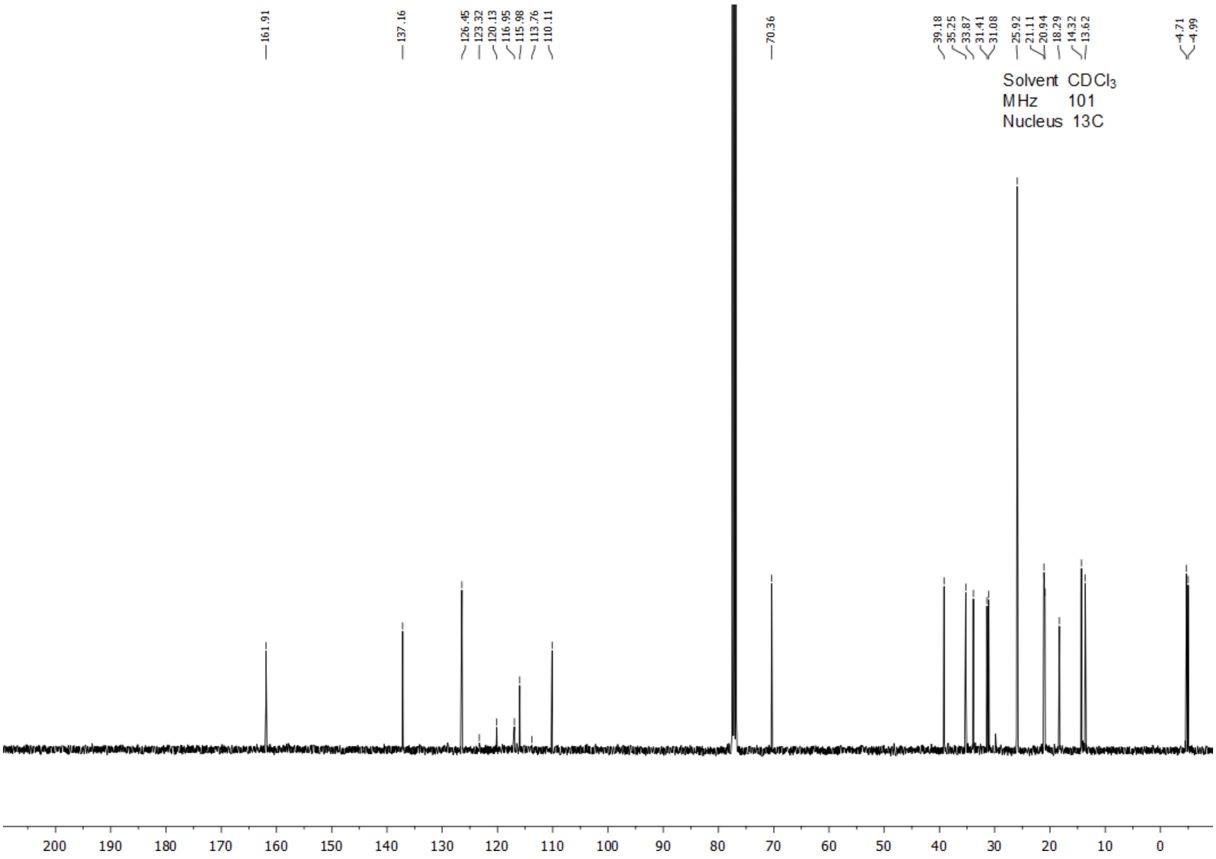
Solvent CDCl3
MHz 101
Nucleus 13C

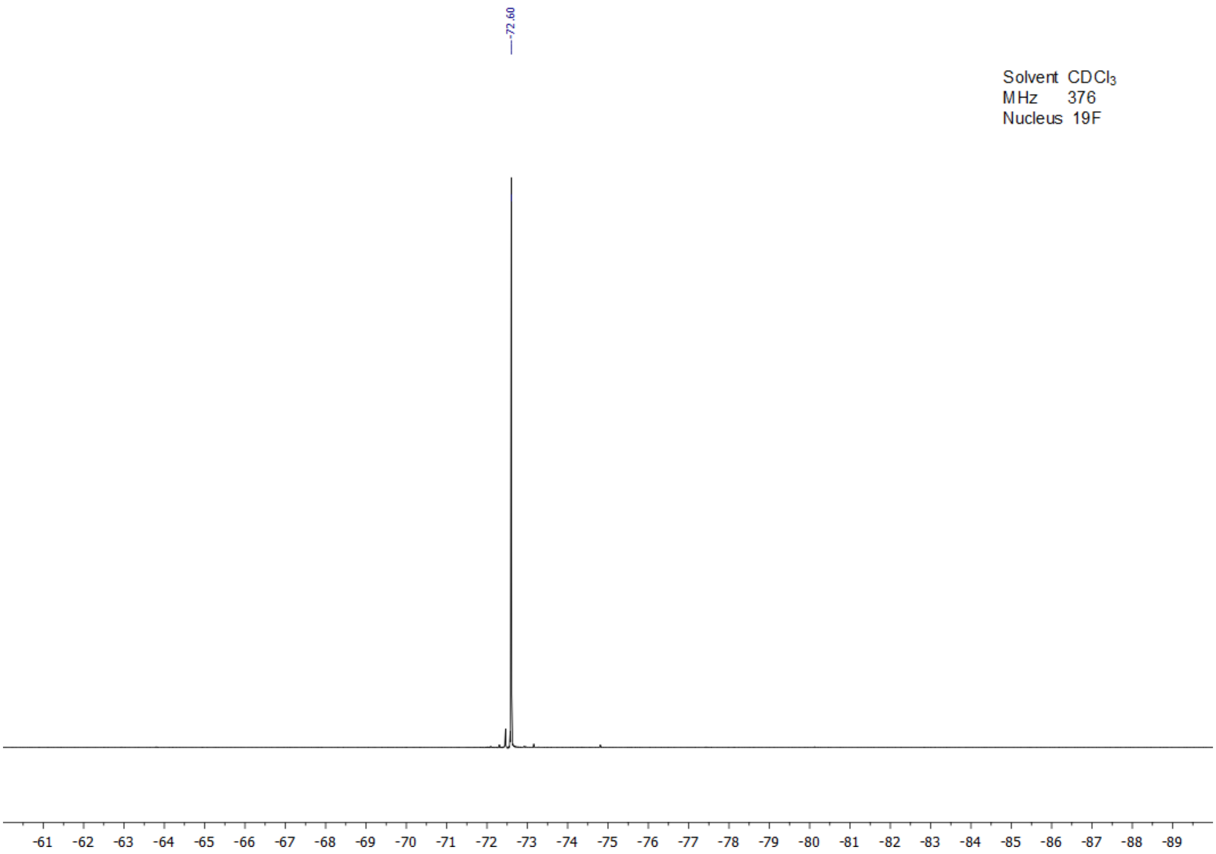

-72.60
Solvent CDCl3
MHz 376
Nucleus 19F
-61 -62 -63 -64 -65 -66 -67 -68 -69 -70 -71 -72 -73 -74 -75 -76 -77 -78 -79 -80 -81 -82 -83 -84 -85 -86 -87 -88 -89

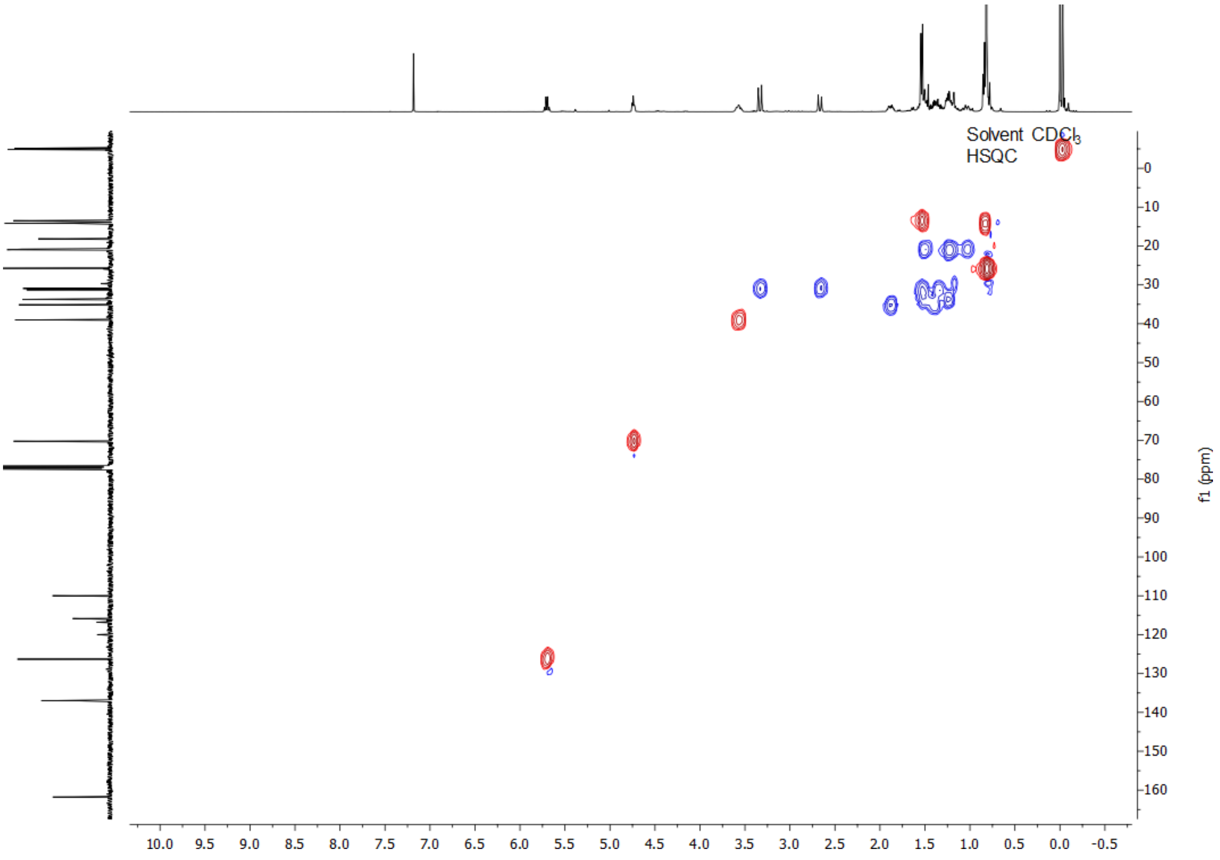

Solvent CDCl3
HSQC
f1 (ppm)
0 10 20 30 40 50 60 70 80 90 100 110 120 130 140 150 160
10.0 9.5 9.0 8.5 8.0 7.5 7.0 6.5 6.0 5.5 5.0 4.5 4.0 3.5 3.0 2.5 2.0 1.5 1.0 0.5 0.0 -0.5

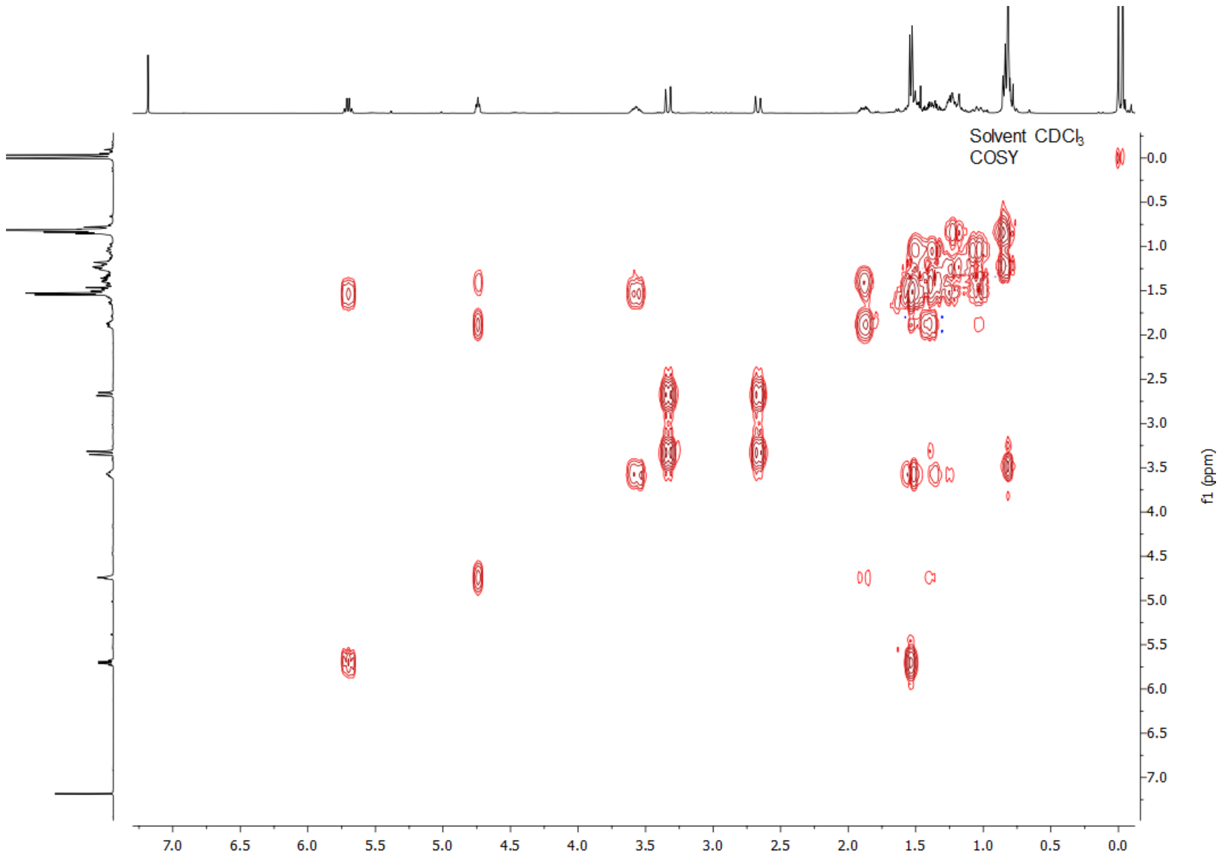
Solvent CDCl3
COSY
f1 (ppm)
0.0
0.5
1.0
1.5
2.0
2.5
3.0
3.5
4.0
4.5
5.0
5.5
6.0
6.5
7.0

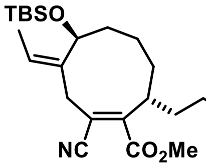
TBSO
NC
CO2Me

29b

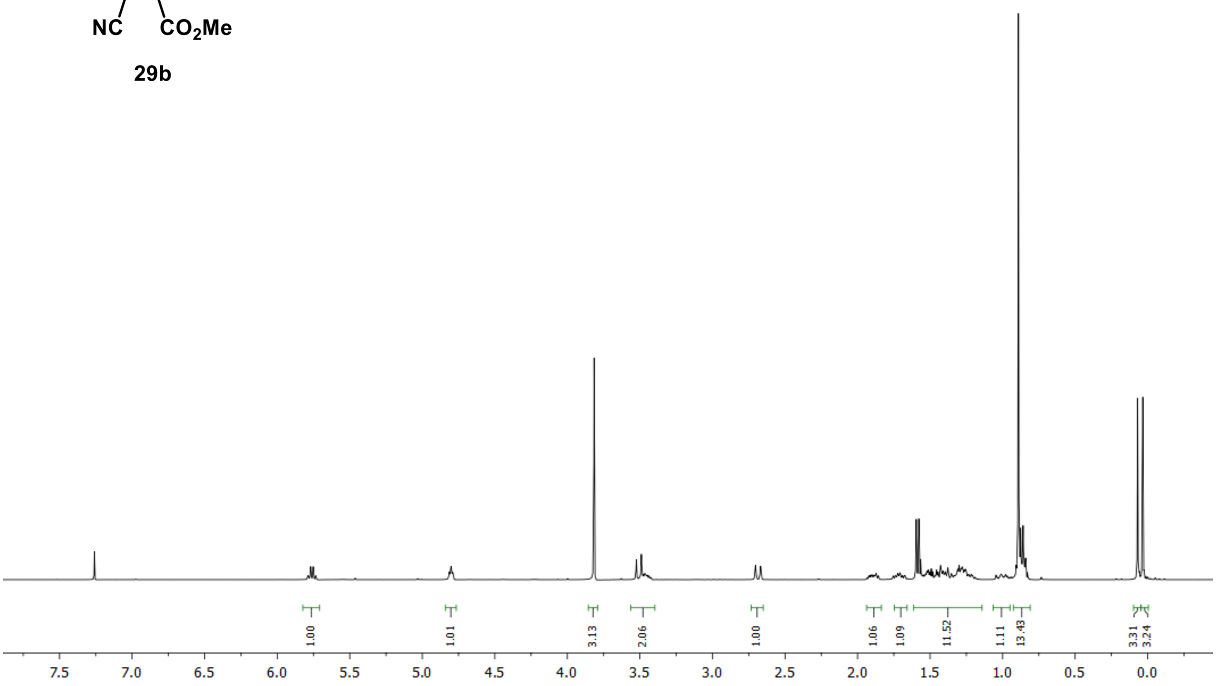
Solvent CDCl3
MHz 400
Nucleus 1H
1.00
1.01
3.13
2.06
1.00
1.06
1.09
11.52
1.11
13.43
3.31
3.24
7.5 7.0 6.5 6.0 5.5 5.0 4.5 4.0 3.5 3.0 2.5 2.0 1.5 1.0 0.5 0.0

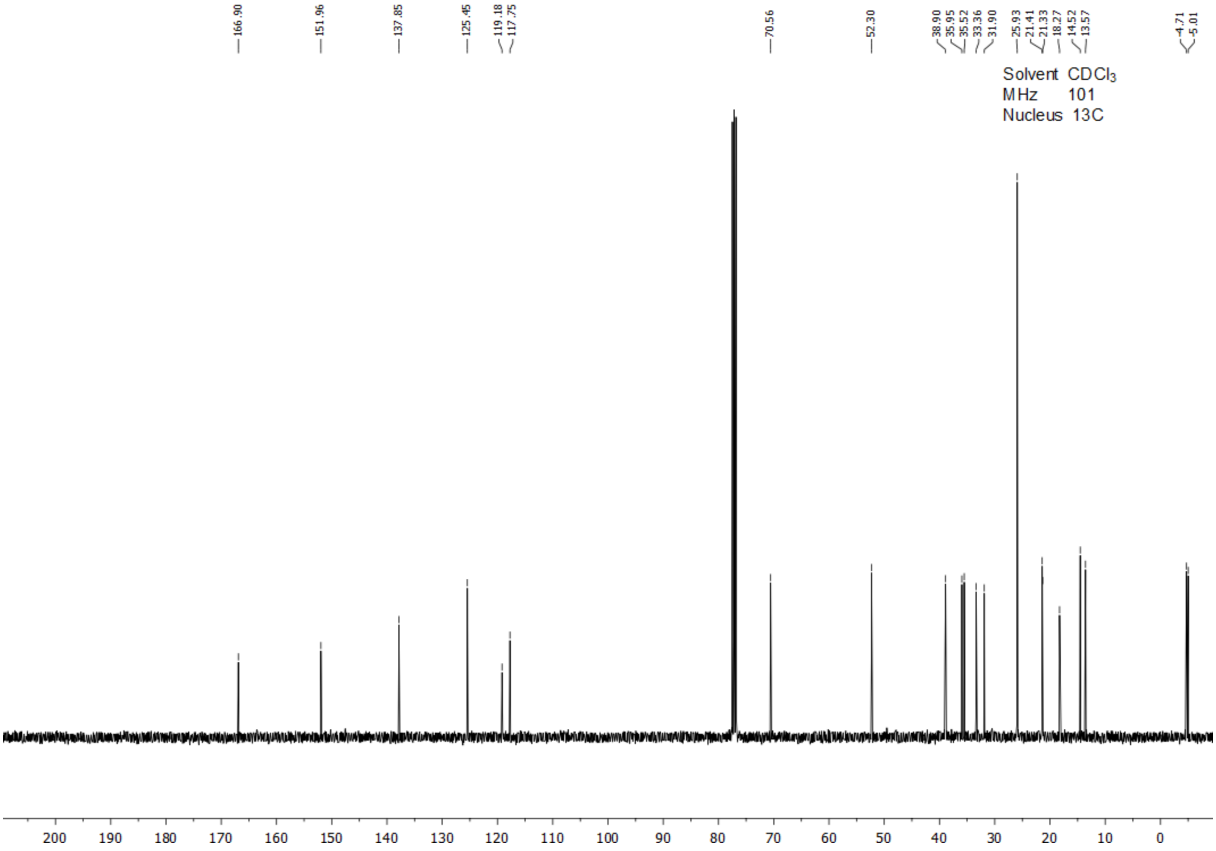
166.90
151.96
137.85
125.45
119.18
117.75
70.56
52.30
38.90
35.95
35.52
33.36
31.90
25.93
21.41
21.33
18.27
14.52
13.57
-4.71
-5.01
Solvent CDCl3
MHz 101
Nucleus 13C
200 190 180 170 160 150 140 130 120 110 100 90 80 70 60 50 40 30 20 10 0

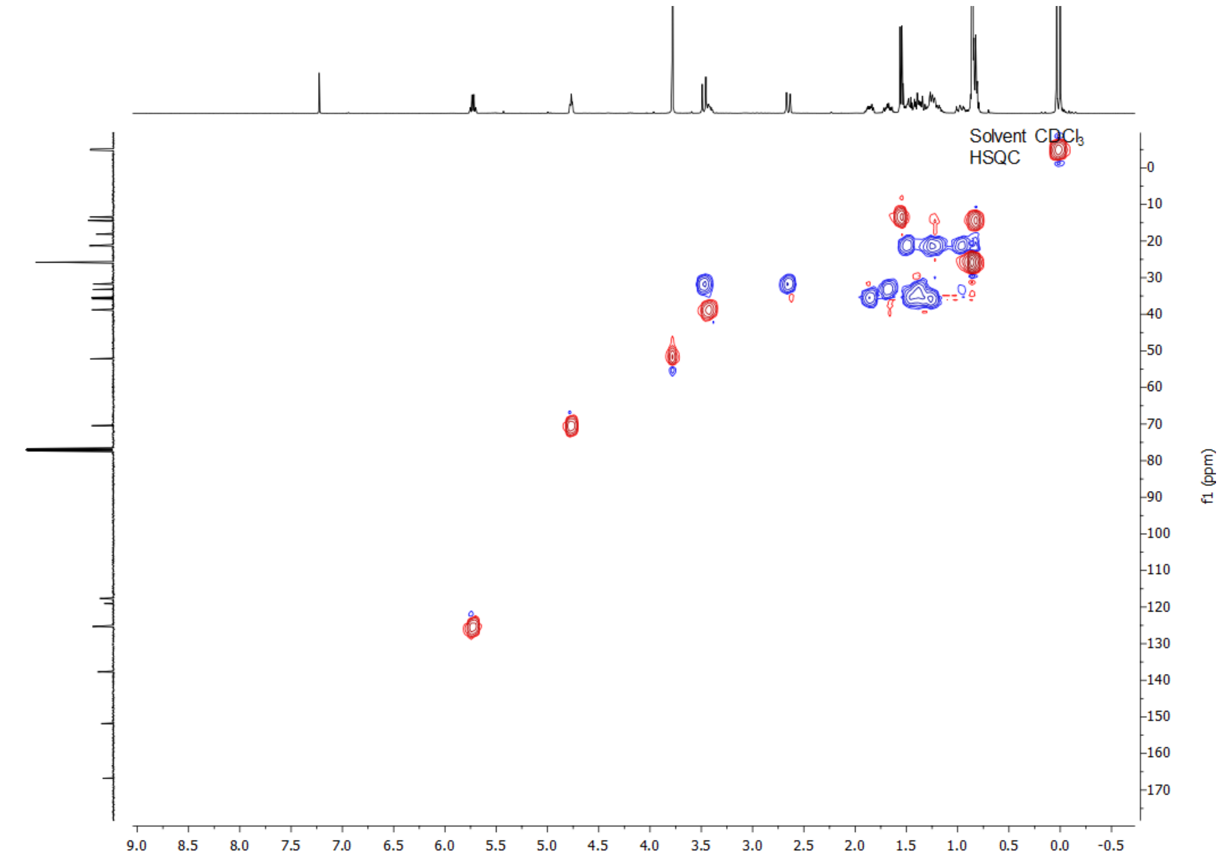
Solvent CDCl3
HSQC
f1 (ppm)

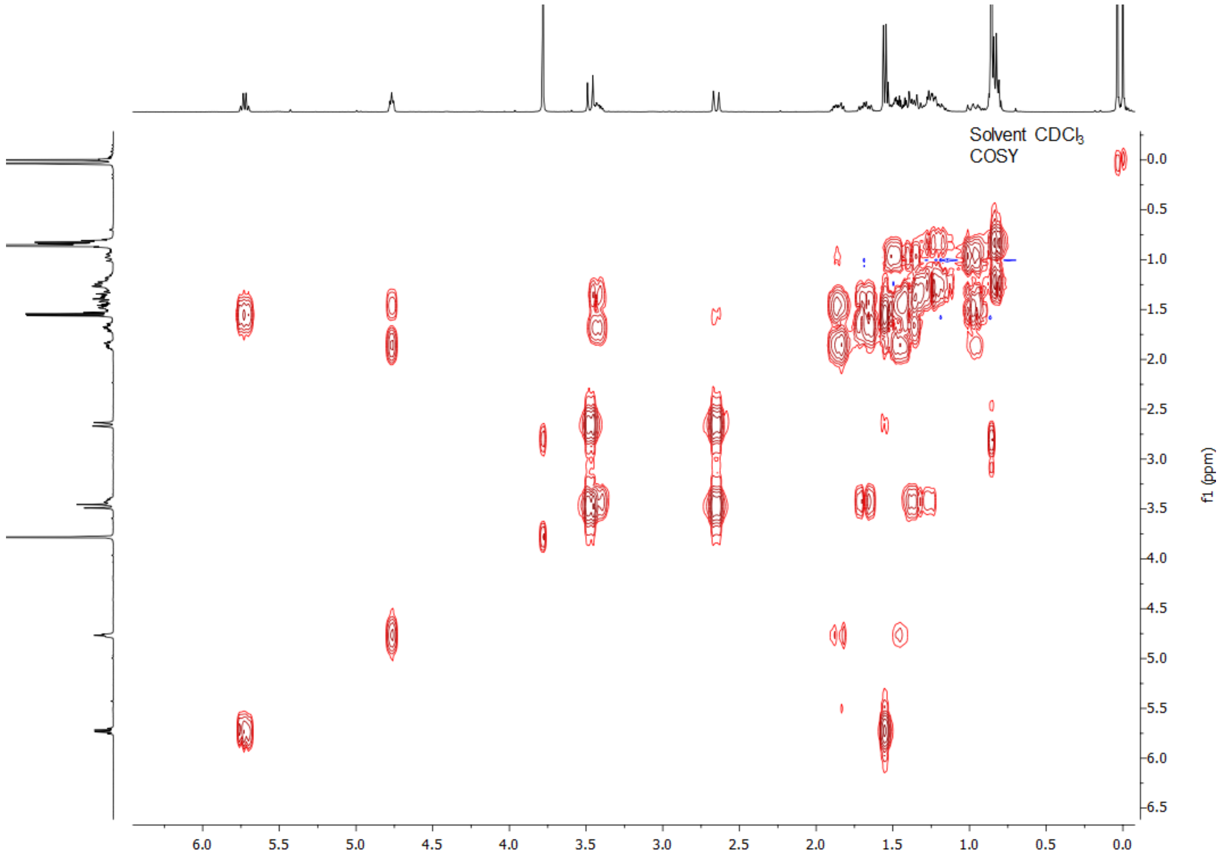
Solvent CDCl3
COSY
f1 (ppm)

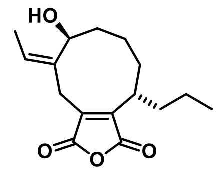
HO
O
O
O

9

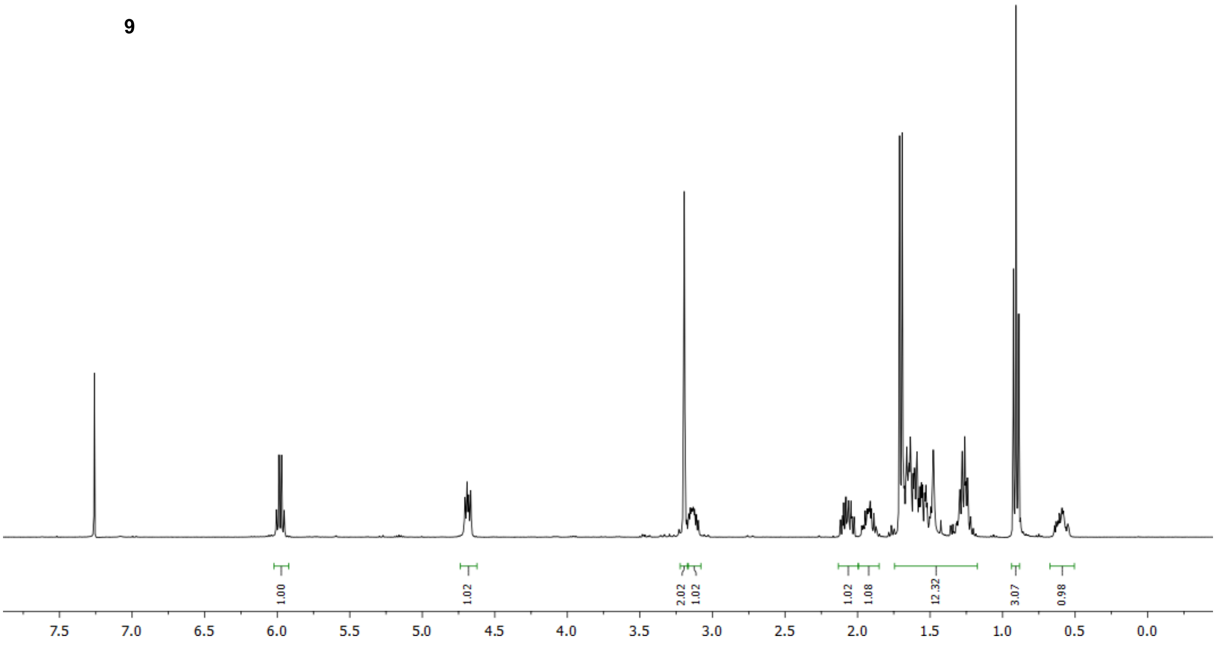
Solvent CDCl3
MHz 400
Nucleus 1H
1.00
1.02
2.02
1.02
1.02
1.08
12.32
3.07
0.98
7.5
7.0
6.5
6.0
5.5
5.0
4.5
4.0
3.5
3.0
2.5
2.0
1.5
1.0
0.5
0.0

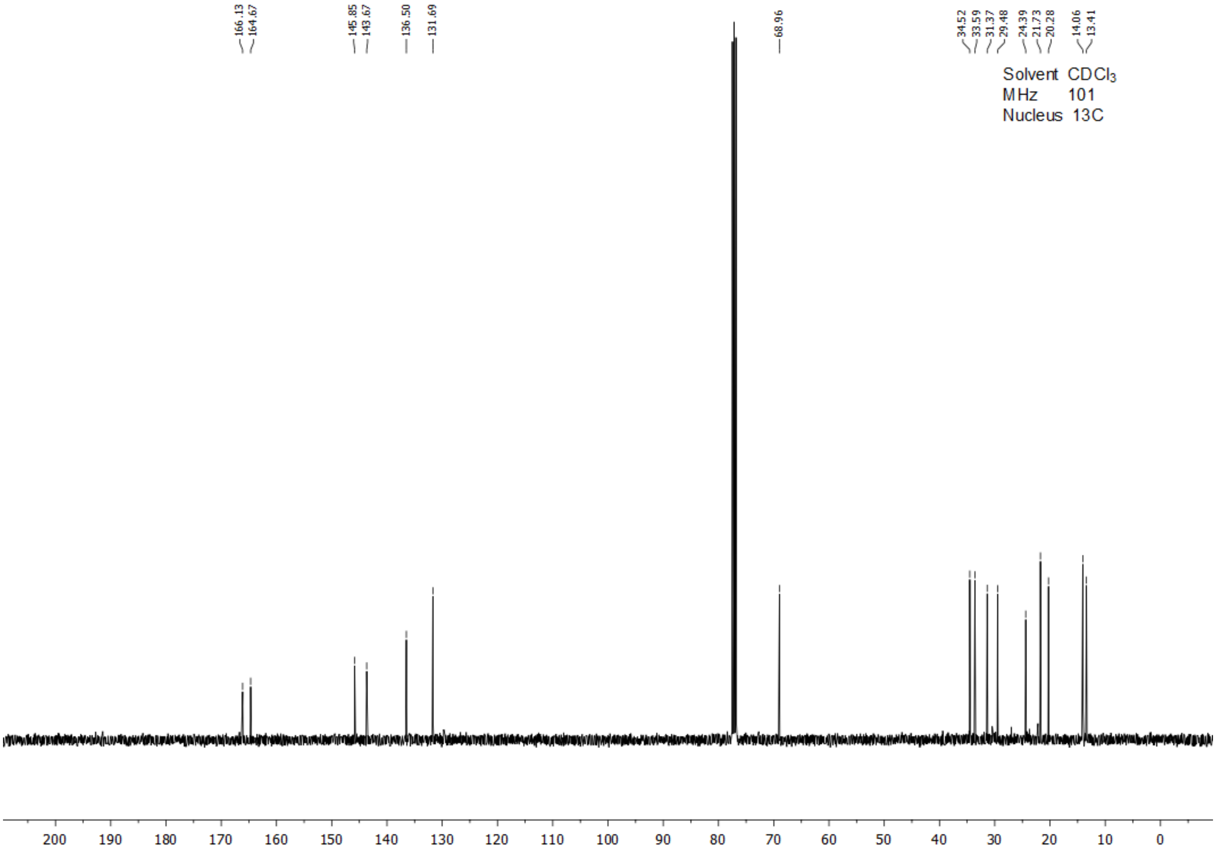
166.13
164.67
145.85
143.67
136.50
131.69
68.96
34.52
33.59
31.37
29.48
24.39
21.73
20.28
14.06
13.41
Solvent CDCl3
MHz 101
Nucleus 13C
200
190
180
170
160
150
140
130
120
110
100
90
80
70
60
50
40
30
20
10
0

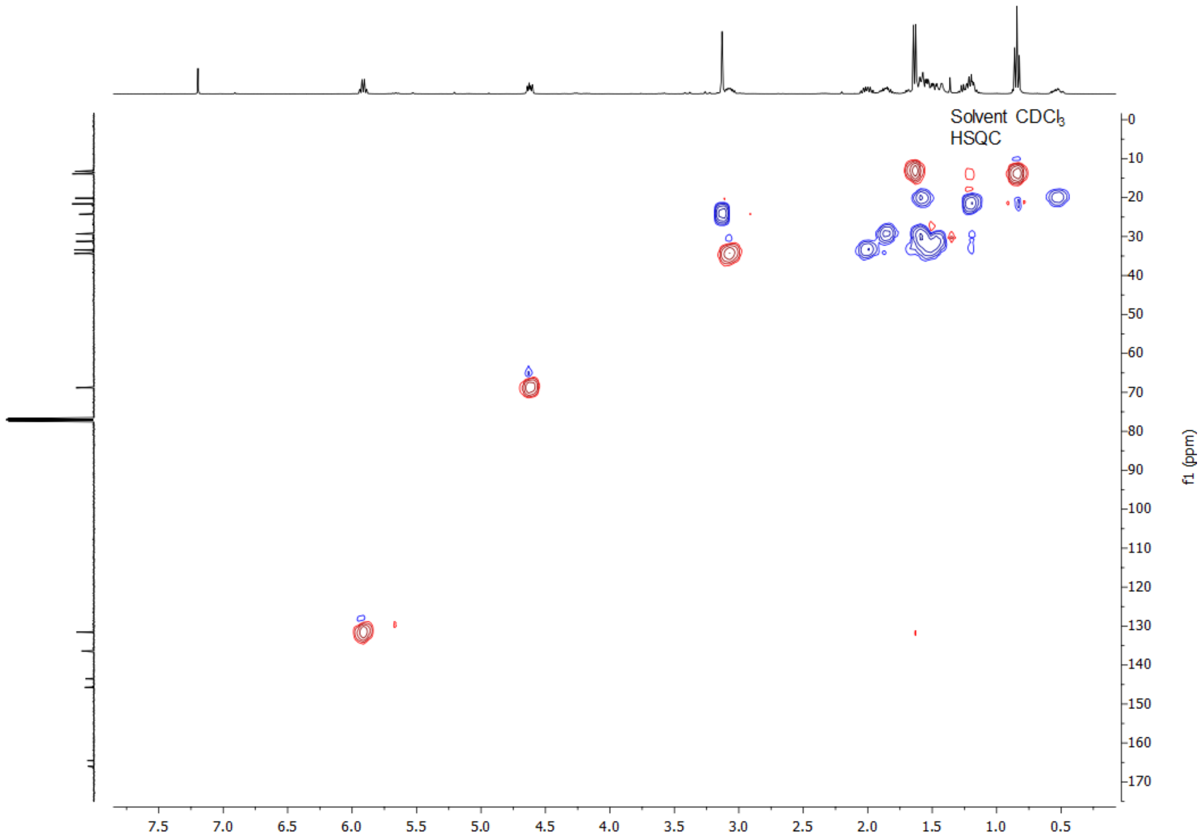
Solvent CDCl3
HSQC
f1 (ppm)

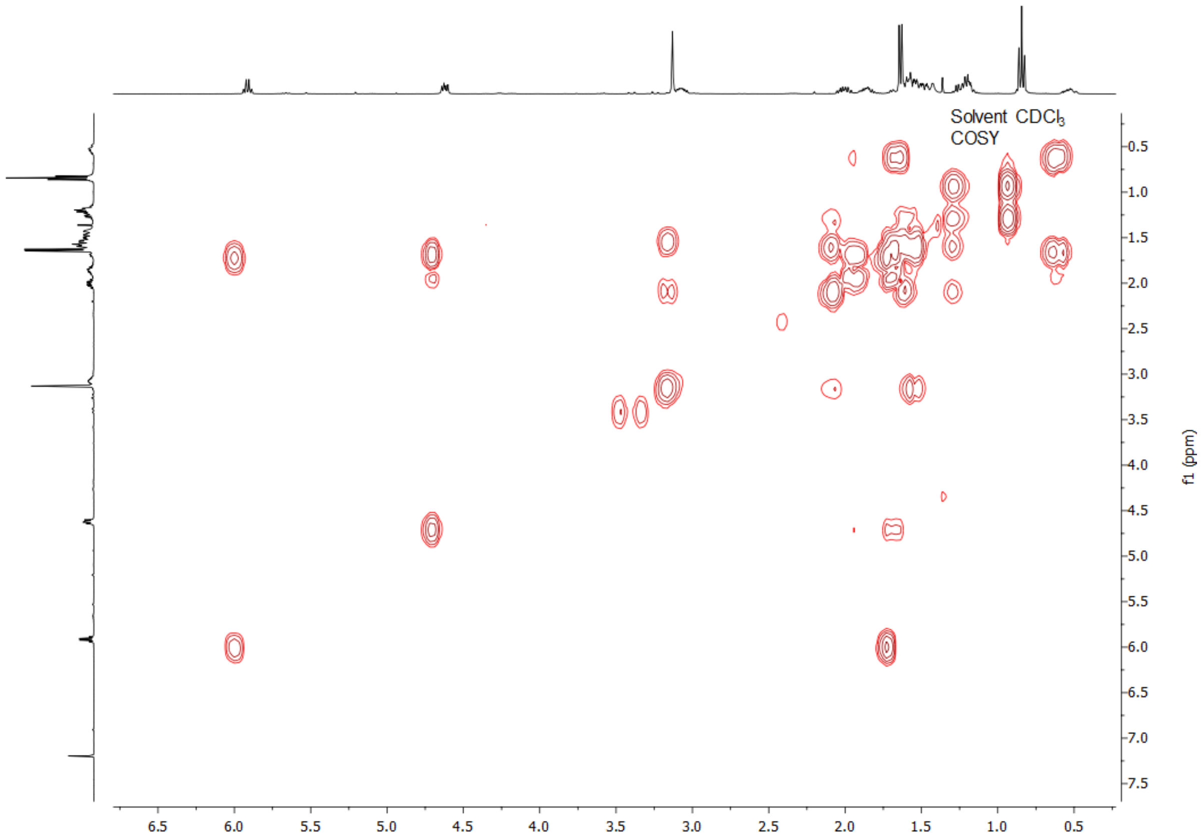
Solvent CDCl3
COSY
f1 (ppm)

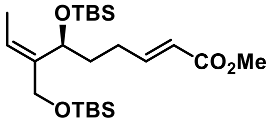
OTBS
CO2Me
OTBS

23

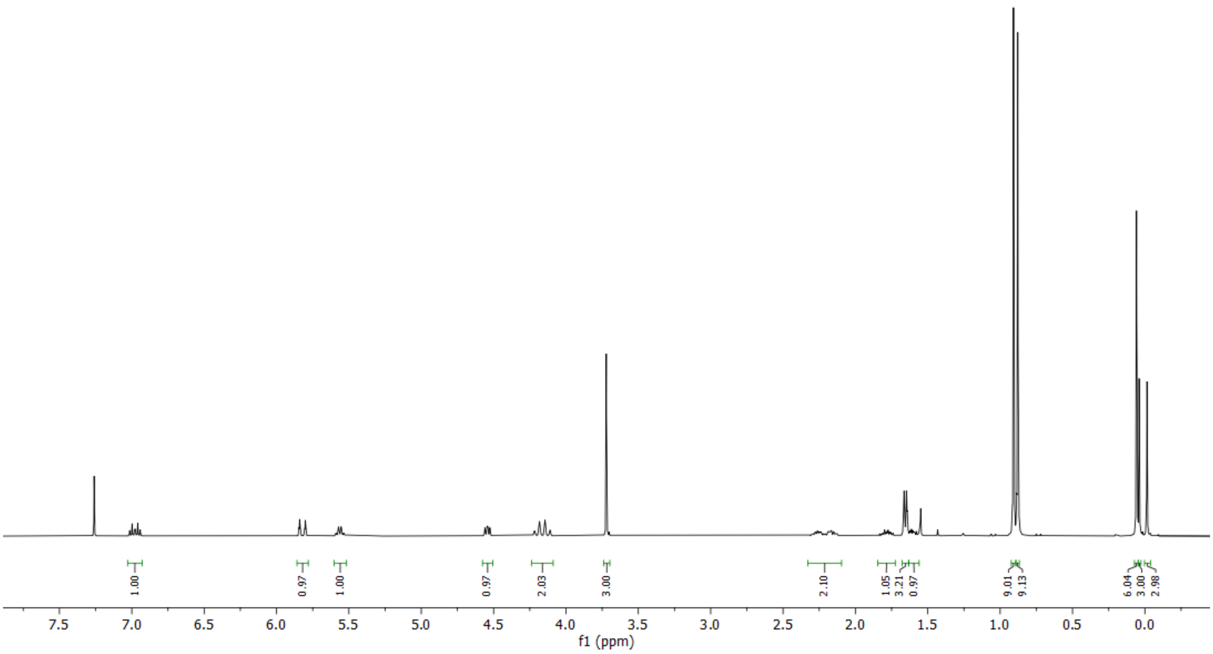
Solvent CDCl3
MHz 400
Nucleus 1H
f1 (ppm)

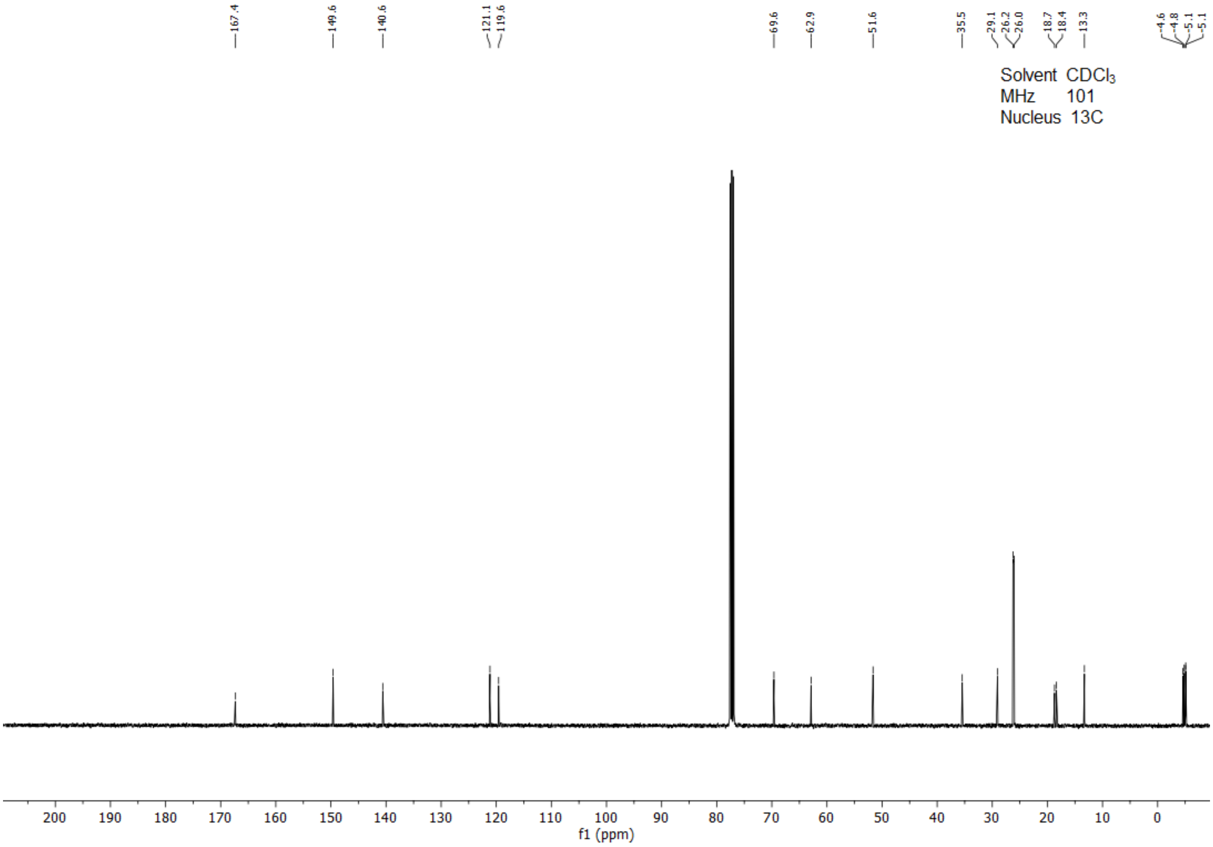
167.4
149.6
140.6
121.1
119.6
69.6
62.9
51.6
35.5
29.1
26.2
26.0
18.7
18.4
13.3
-4.6
-4.8
-5.1
-5.1
Solvent CDCl3
MHz 101
Nucleus 13C
f1 (ppm)

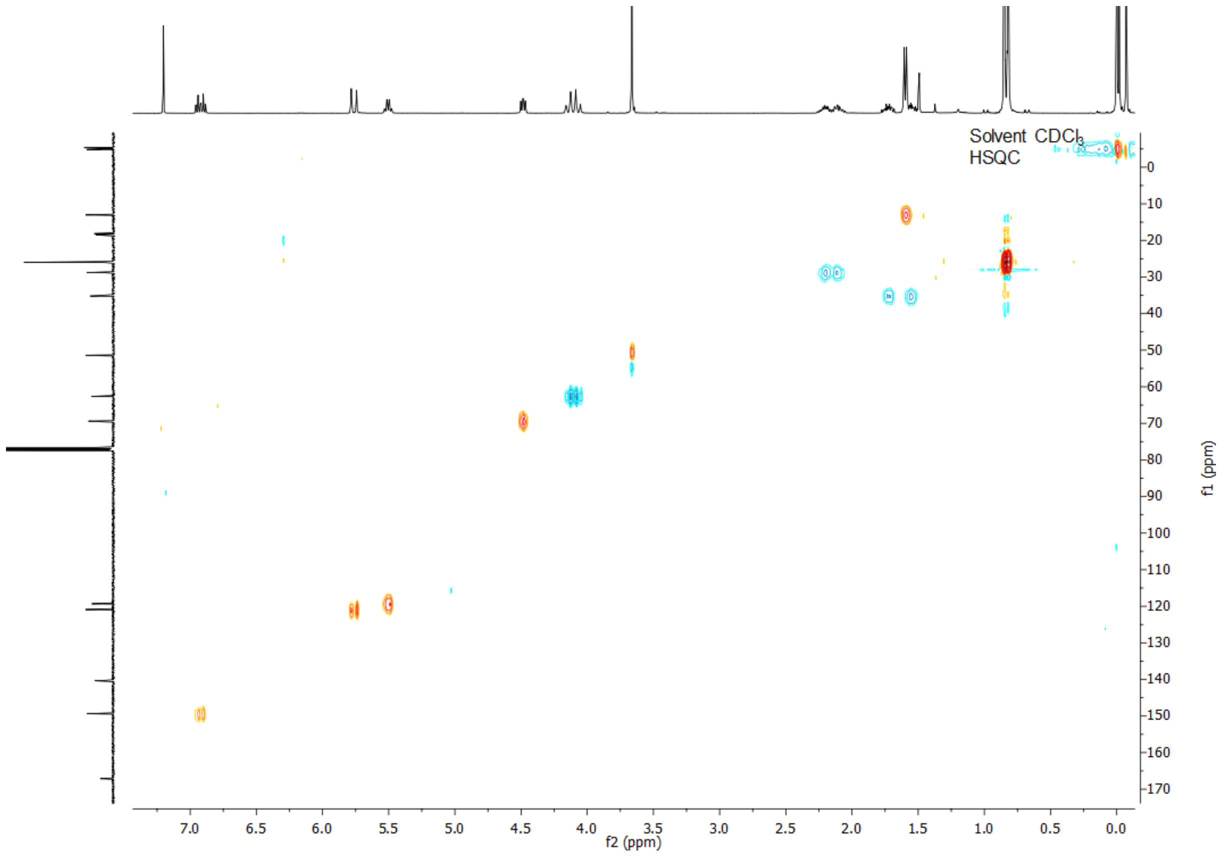
Solvent CDCl3
HSQC
f2 (ppm)
f1 (ppm)

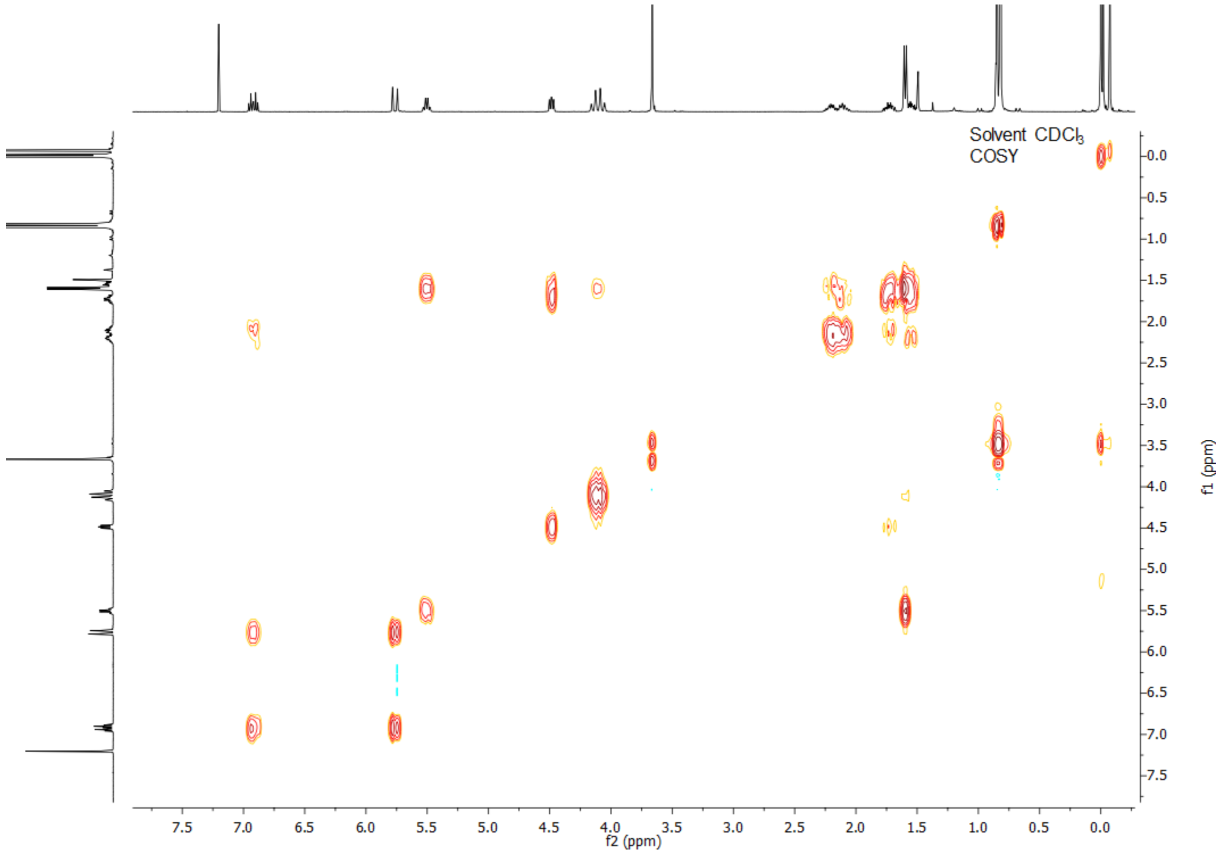
Solvent CDCl3
COSY
f2 (ppm)
f1 (ppm)

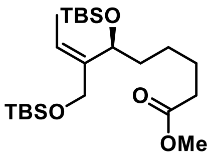
TBSO
TBSO
O
OMe

24

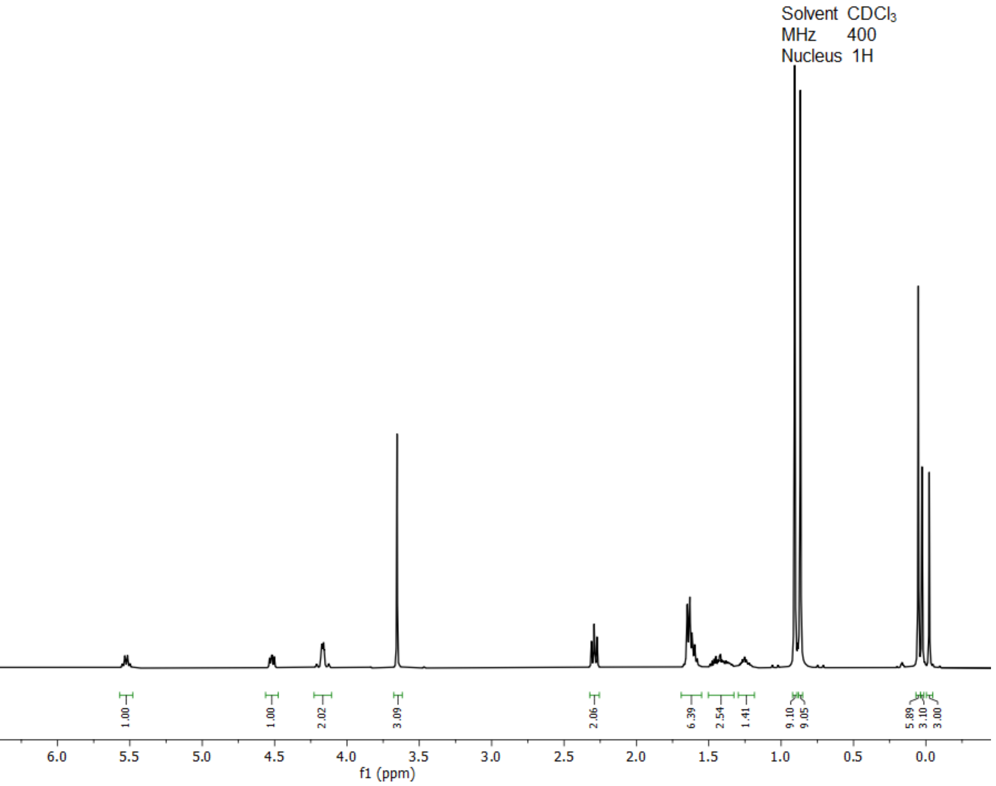
Solvent CDCl3
MHz 400
Nucleus 1H
1.00
1.00
2.02
3.09
2.06
6.39
2.54
1.41
9.10
9.05
5.89
3.10
3.00
7.5 7.0 6.5 6.0 5.5 5.0 4.5 4.0 3.5 3.0 2.5 2.0 1.5 1.0 0.5 0.0
f1 (ppm)

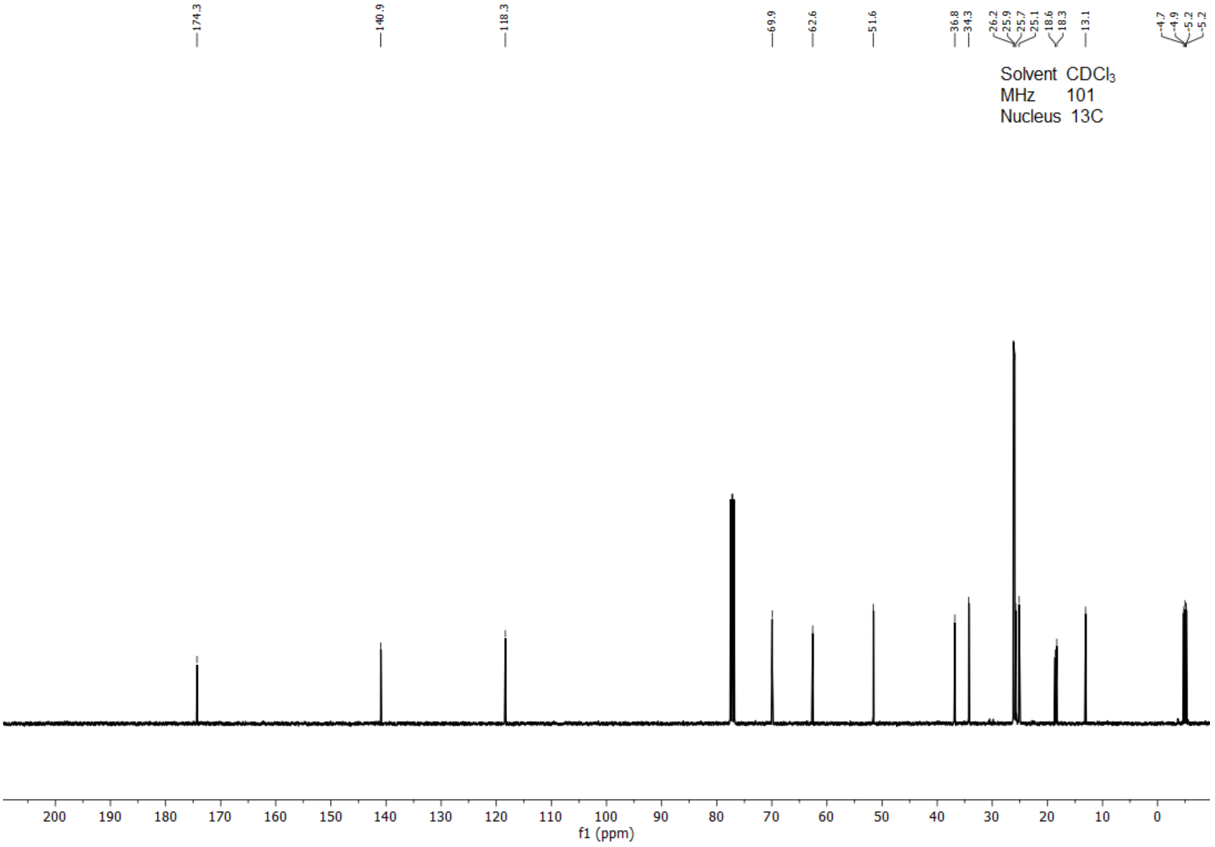
174.3
140.9
118.3
69.9
62.6
51.6
36.8
34.3
26.2
25.9
25.7
25.1
18.6
18.3
13.1
-4.7
-4.9
-5.2
-5.2
Solvent CDCl3
MHz 101
Nucleus 13C
200 190 180 170 160 150 140 130 120 110 100 90 80 70 60 50 40 30 20 10 0
f1 (ppm)

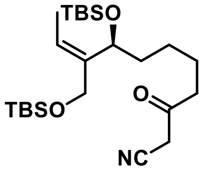
TBSO
TBSO
O
NC

21c

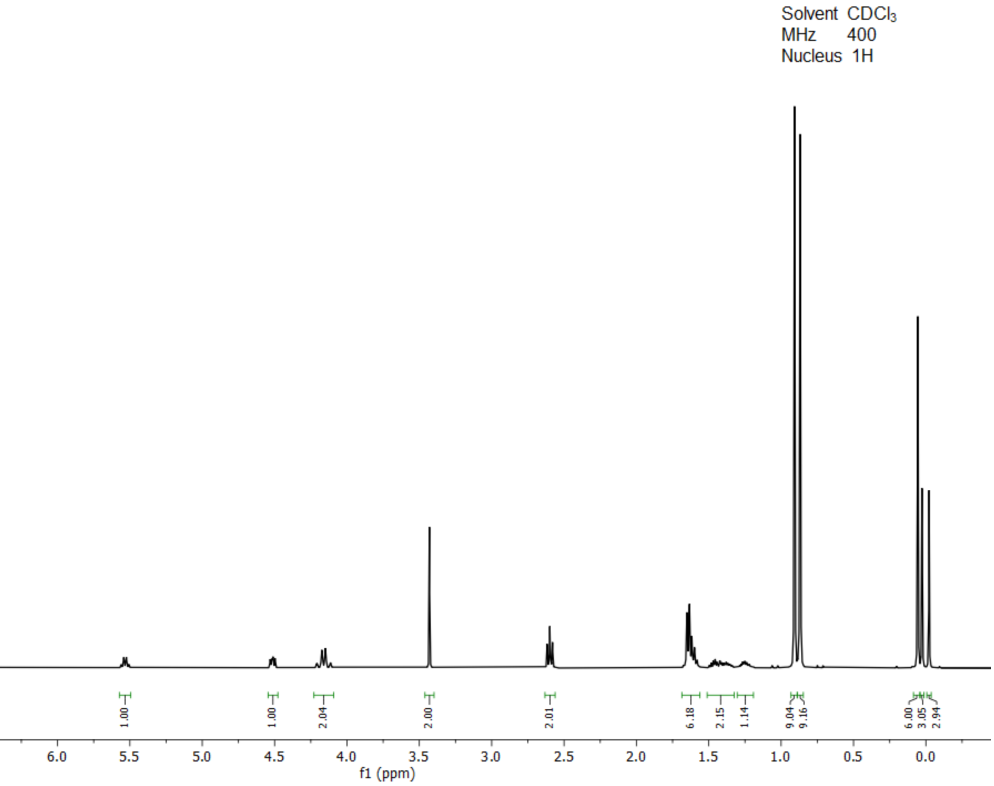
Solvent CDCl$_3$
MHz 400
Nucleus 1H
f1 (ppm)

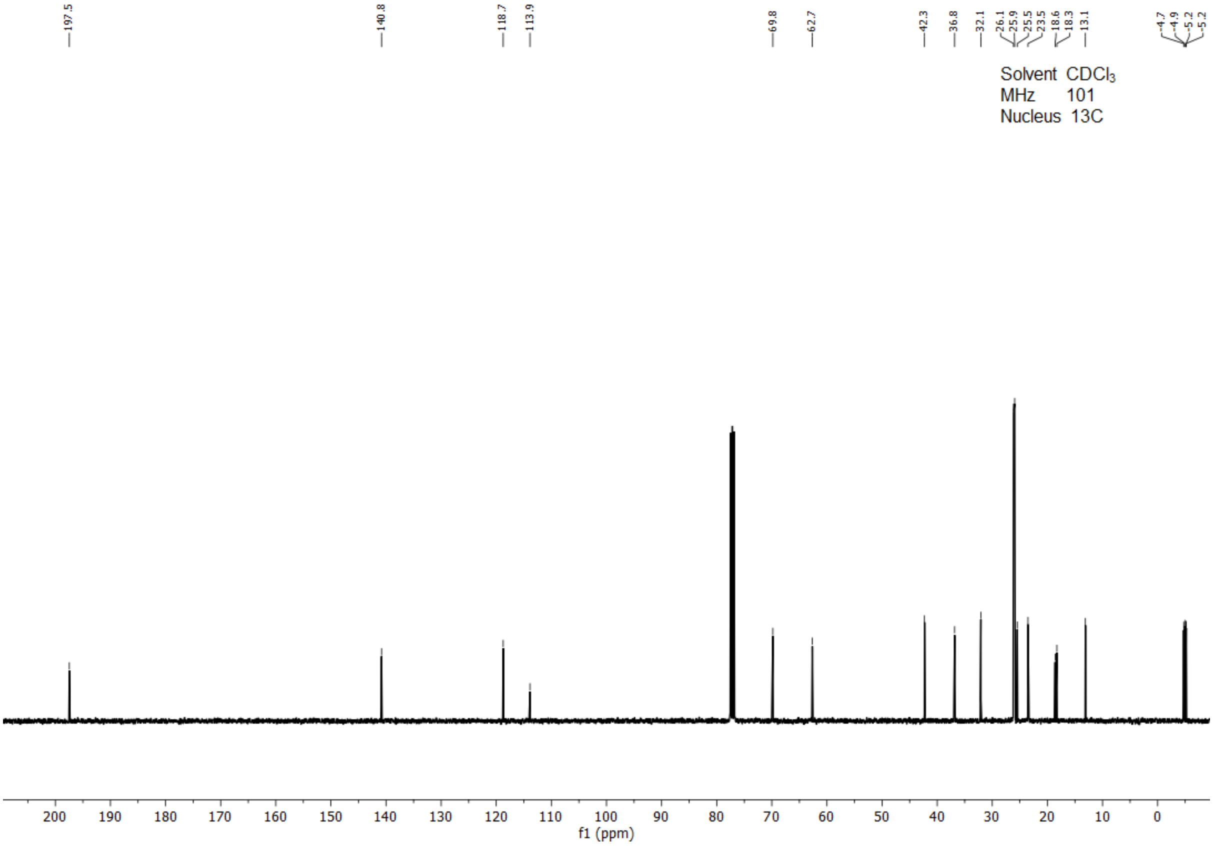
197.5
140.8
118.7
113.9
69.8
62.7
42.3
36.8
32.1
26.1
25.9
25.5
23.5
18.6
18.3
13.1
-4.7
-4.9
-5.2
-5.2
Solvent CDCl$_3$
MHz 101
Nucleus 13C
f1 (ppm)

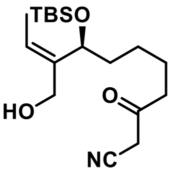

TBSO
HO
O
NC

25c

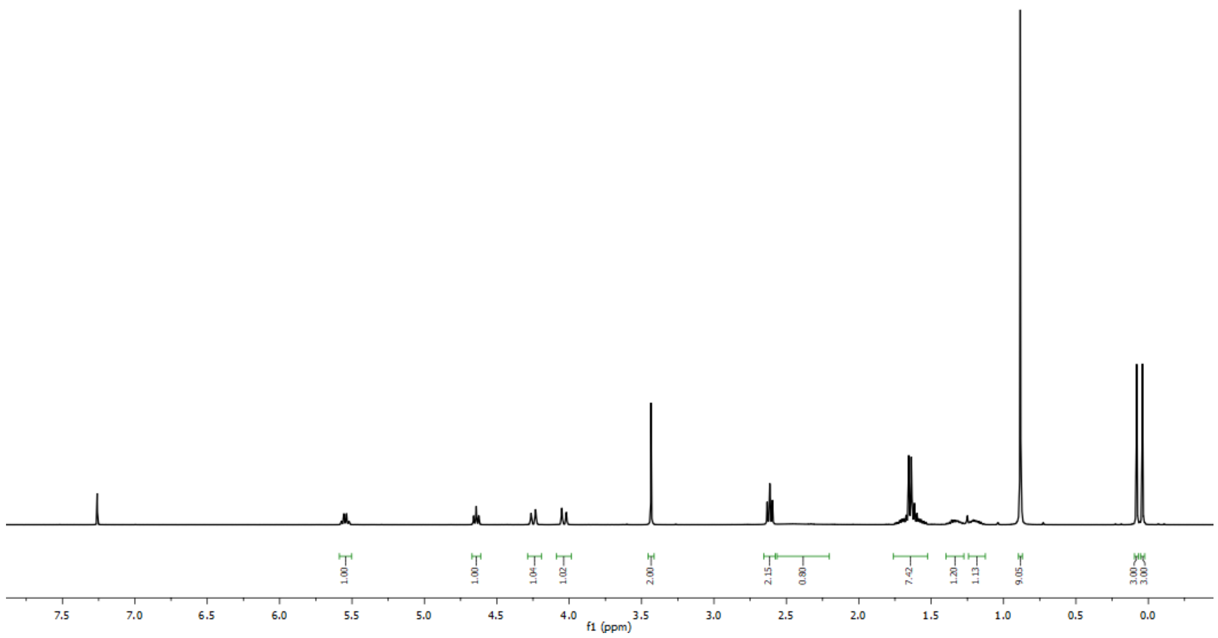

Solvent CDCl3
MHz 400
Nucleus 1H
1.00
1.00
1.04
1.02
2.00
2.15
0.80
7.42
1.20
1.13
9.05
3.00
3.00
f1 (ppm)

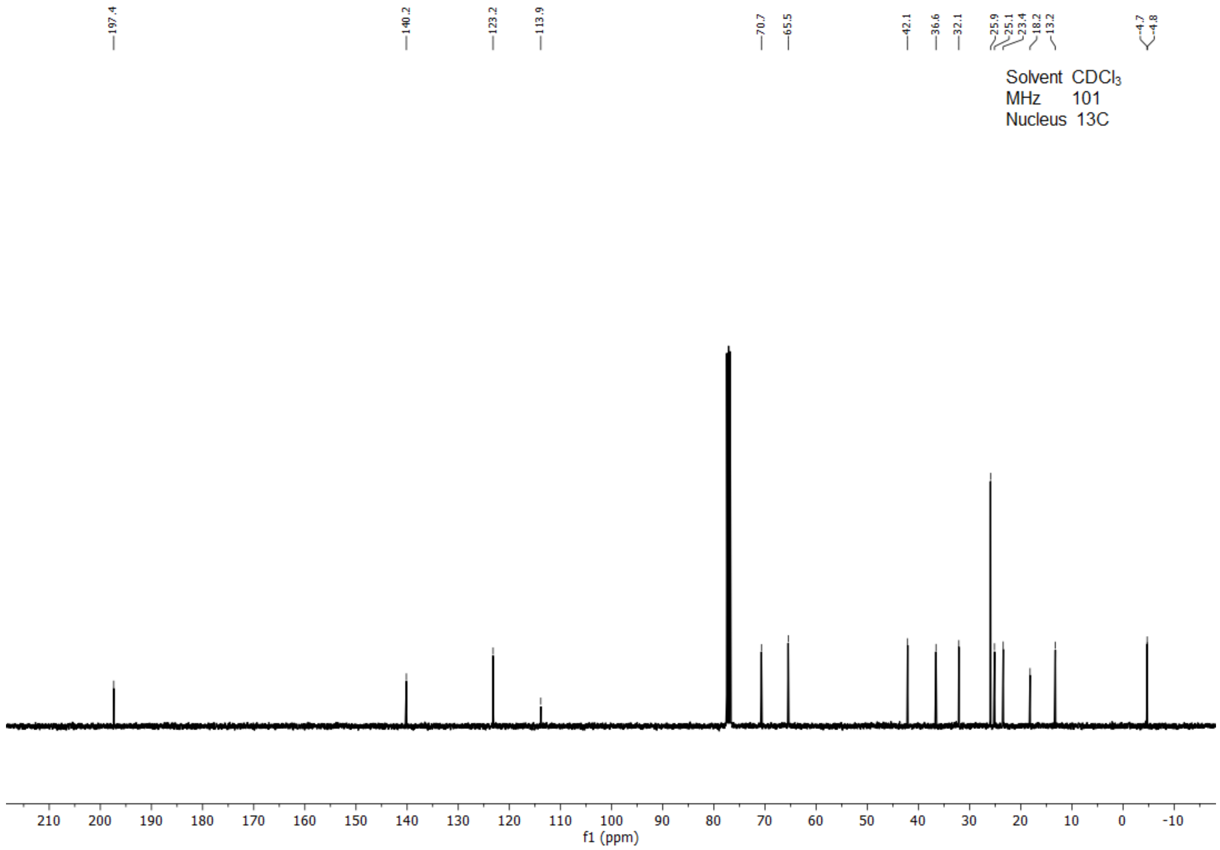

197.4
140.2
123.2
113.9
70.7
65.5
42.1
36.6
32.1
25.9
25.1
23.4
18.2
13.2
-4.7
-4.8
Solvent CDCl3
MHz 101
Nucleus 13C
f1 (ppm)

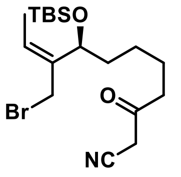

26c

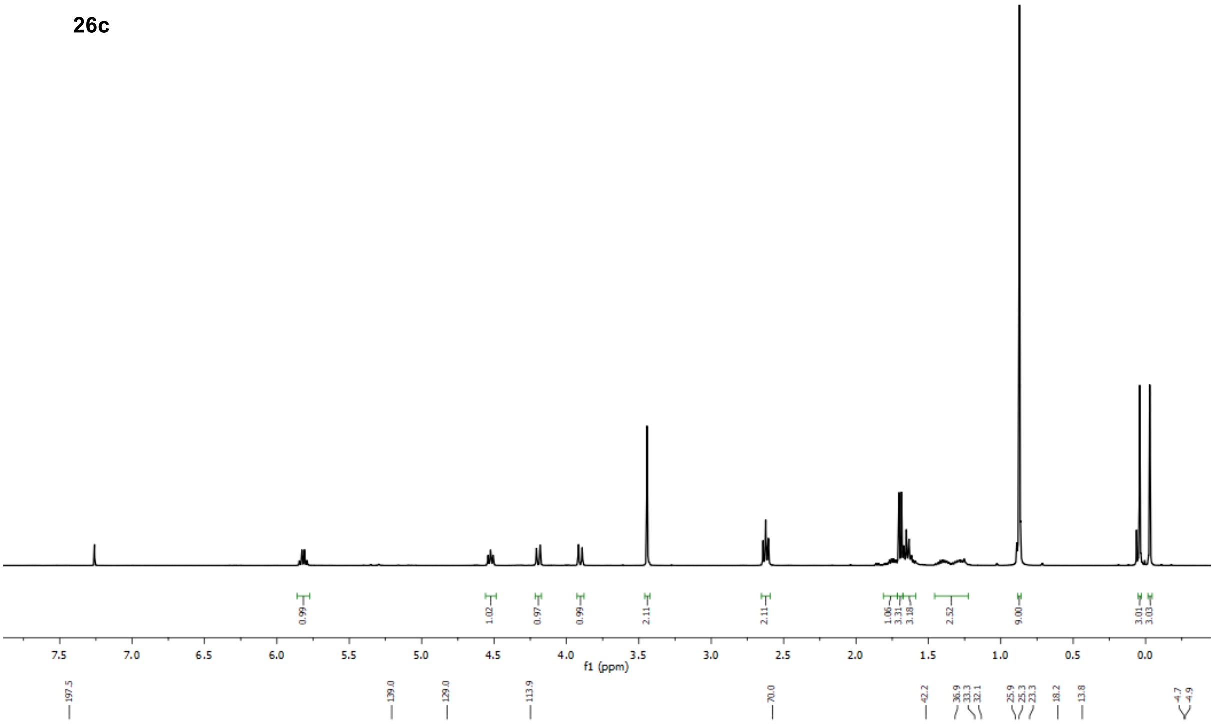

Solvent CDCl$_3$
MHz 101
Nucleus 13C

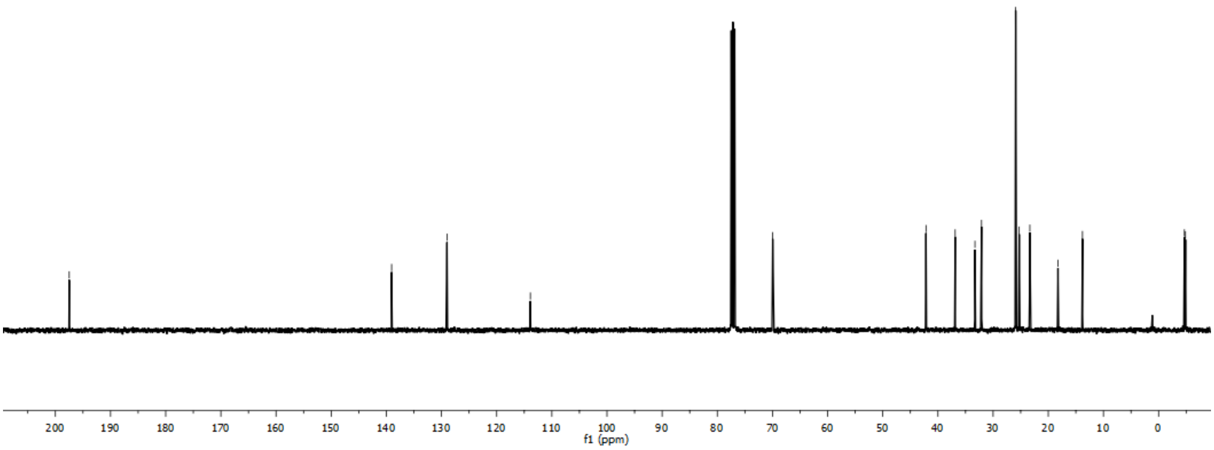

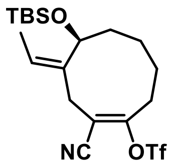
TBSO
NC
OTf

28c

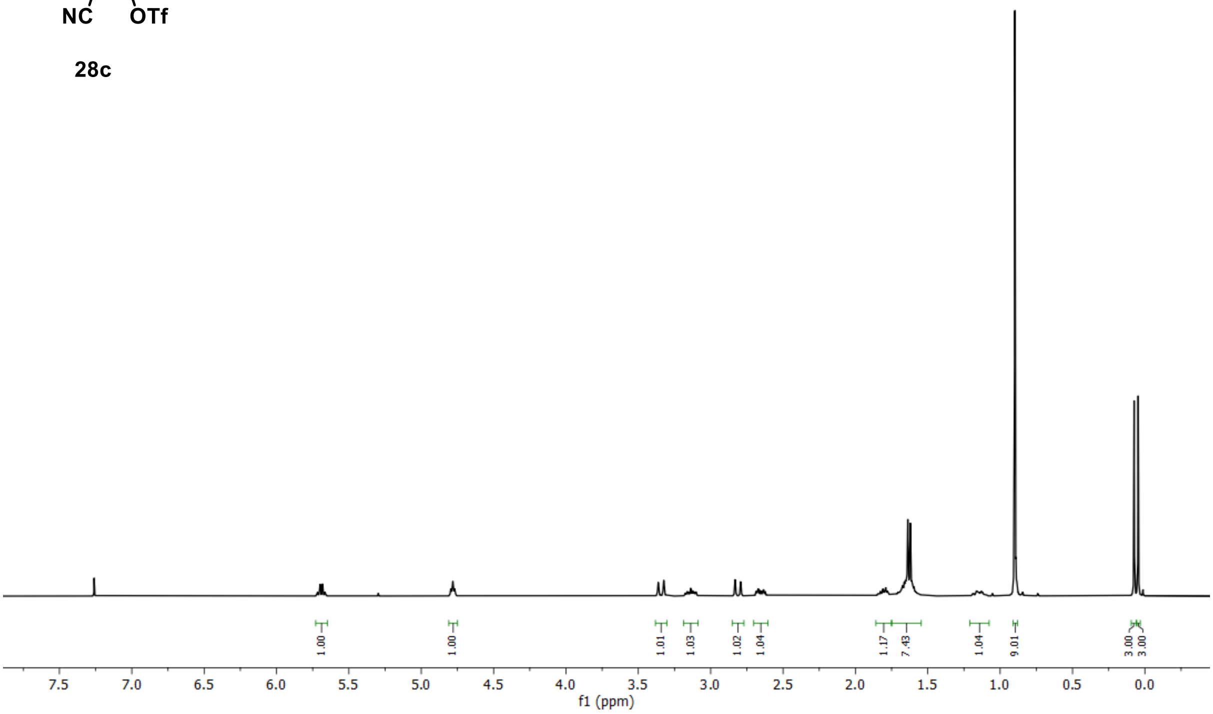
Solvent CDCl3
MHz 400
Nucleus 1H
1.00
1.00
1.01
1.03
1.02
1.04
1.17
7.43
1.04
9.01
3.00
3.00
f1 (ppm)

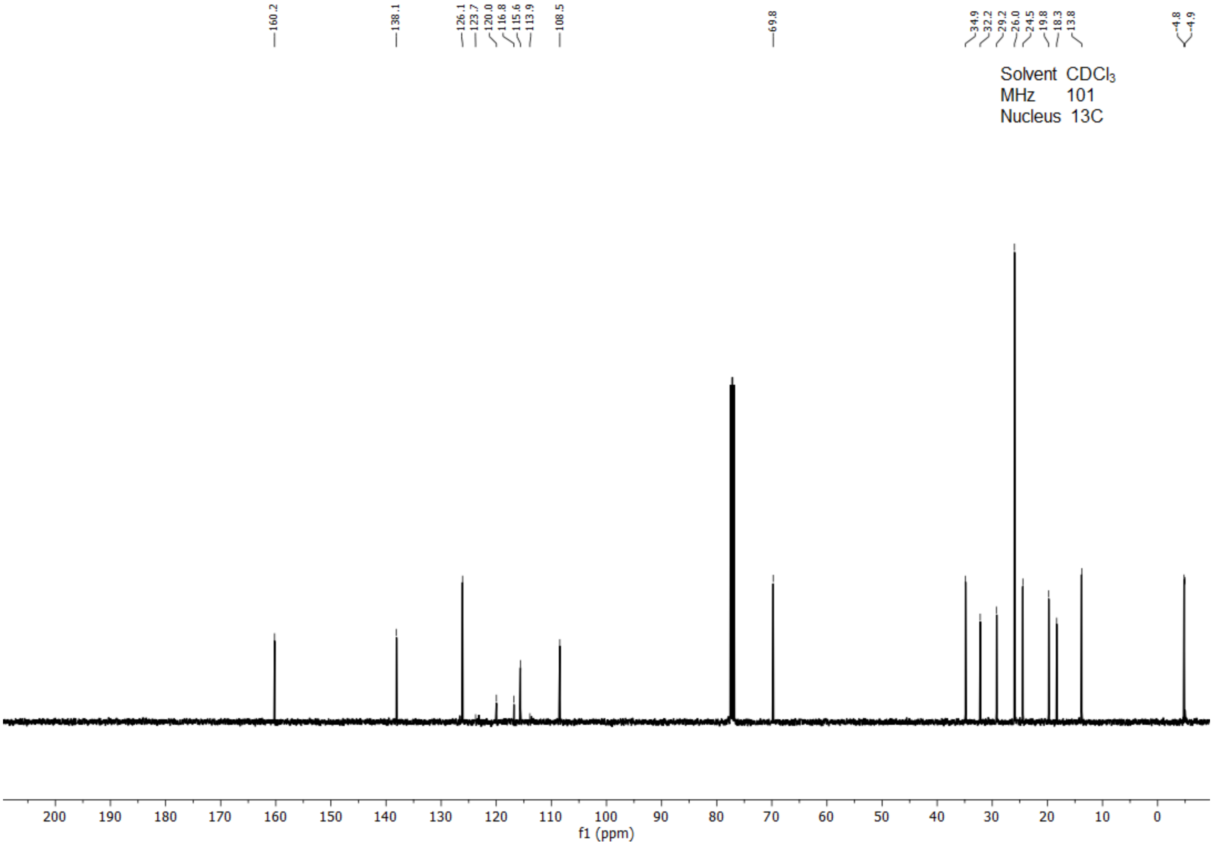
160.2
138.1
126.1
123.7
120.0
116.8
115.6
113.9
108.5
69.8
34.9
32.2
29.2
26.0
24.5
19.8
18.3
13.8
-4.8
-4.9
Solvent CDCl3
MHz 101
Nucleus 13C
f1 (ppm)

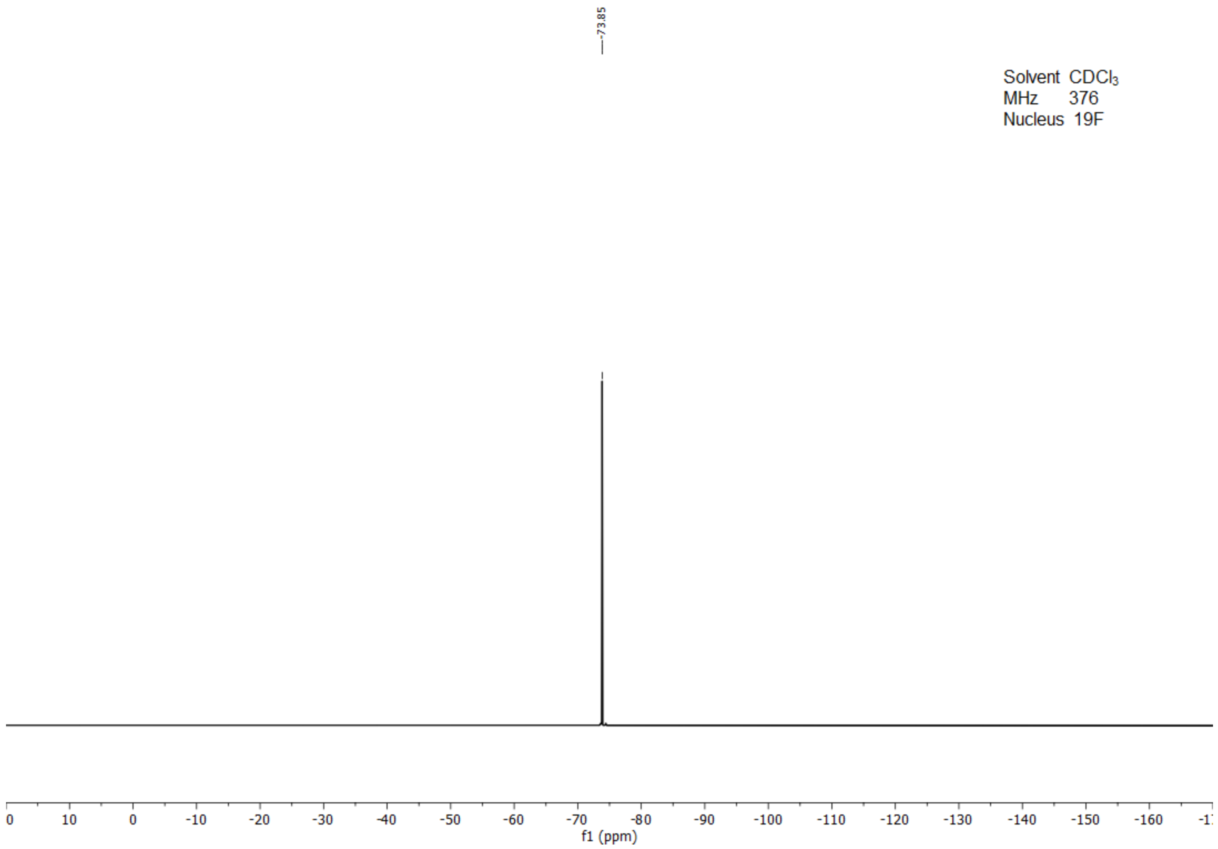

-73.85
Solvent CDCl3
MHz 376
Nucleus 19F
f1 (ppm)

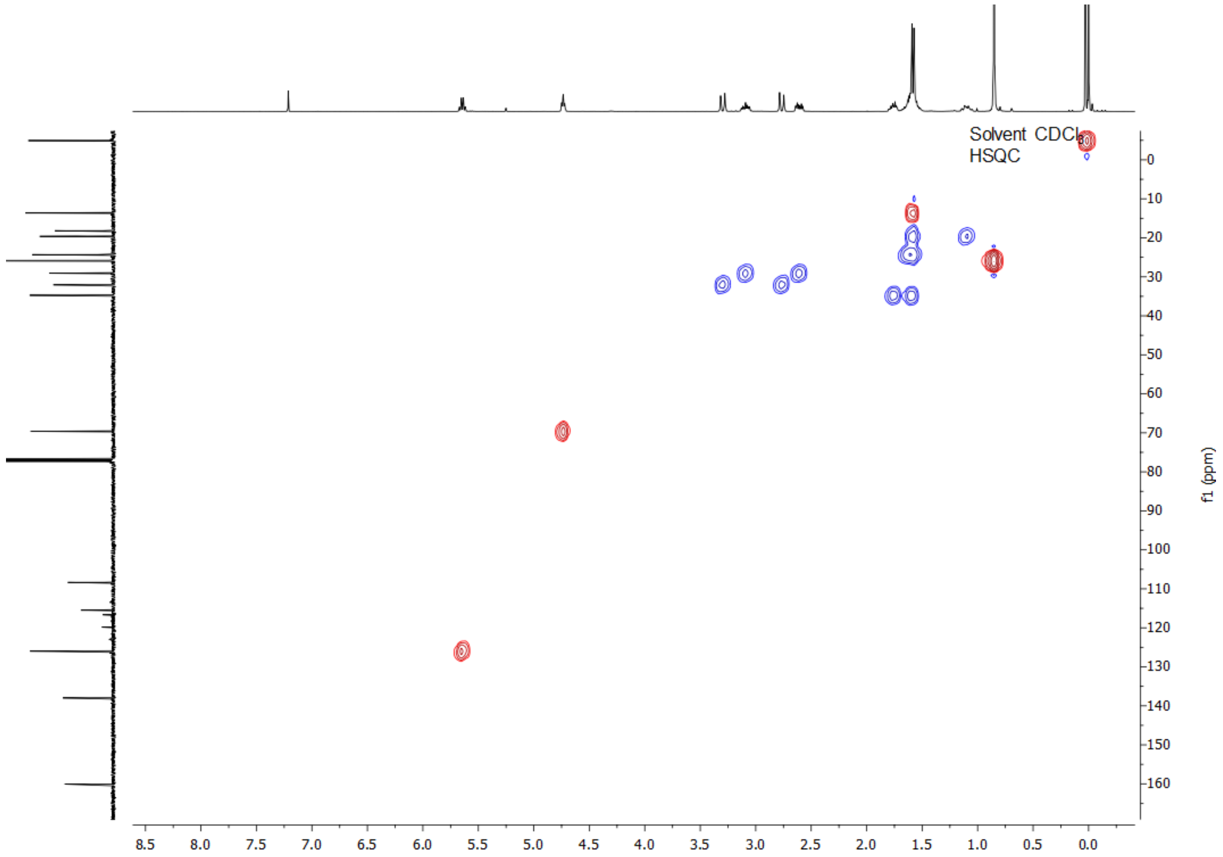

Solvent CDCl3
HSQC
f1 (ppm)

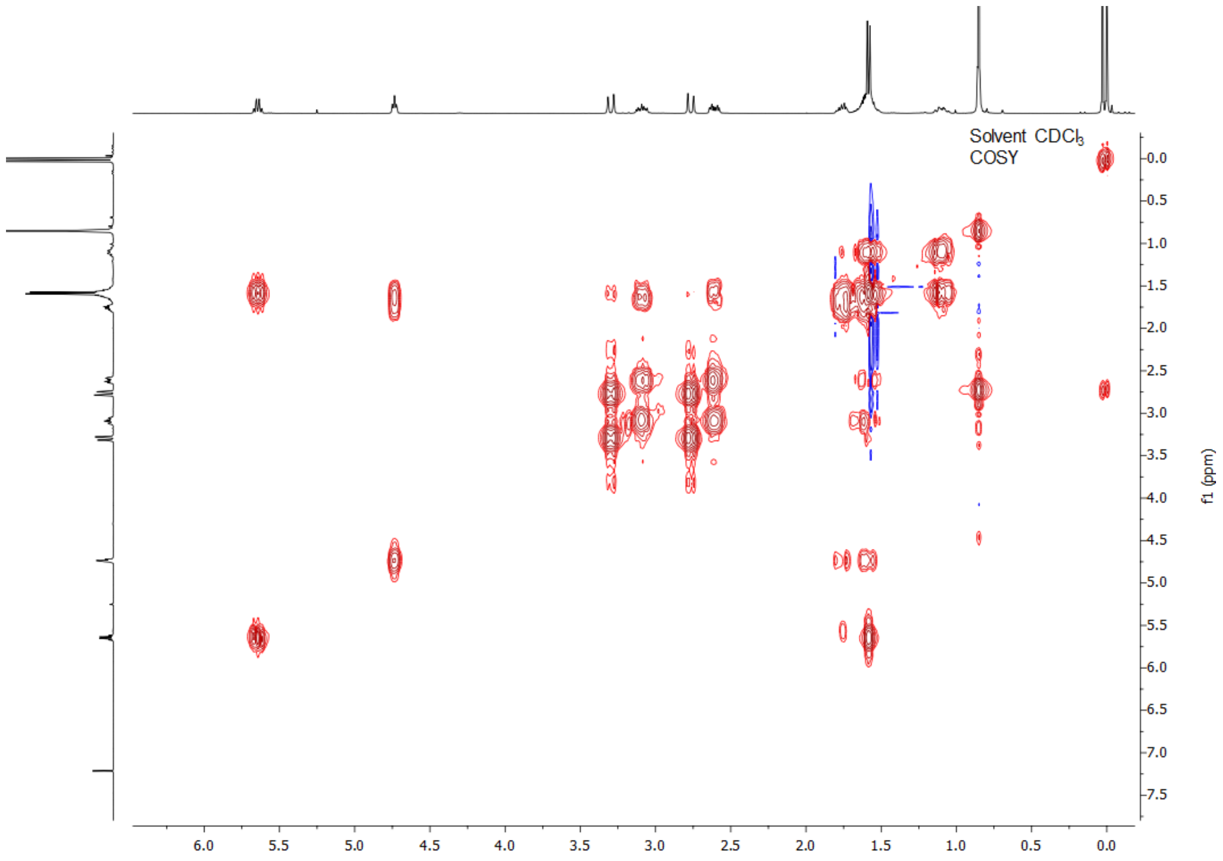
Solvent CDCl3
COSY
f1 (ppm)

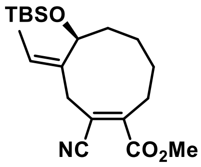
TBSO
NC
CO2Me

29c

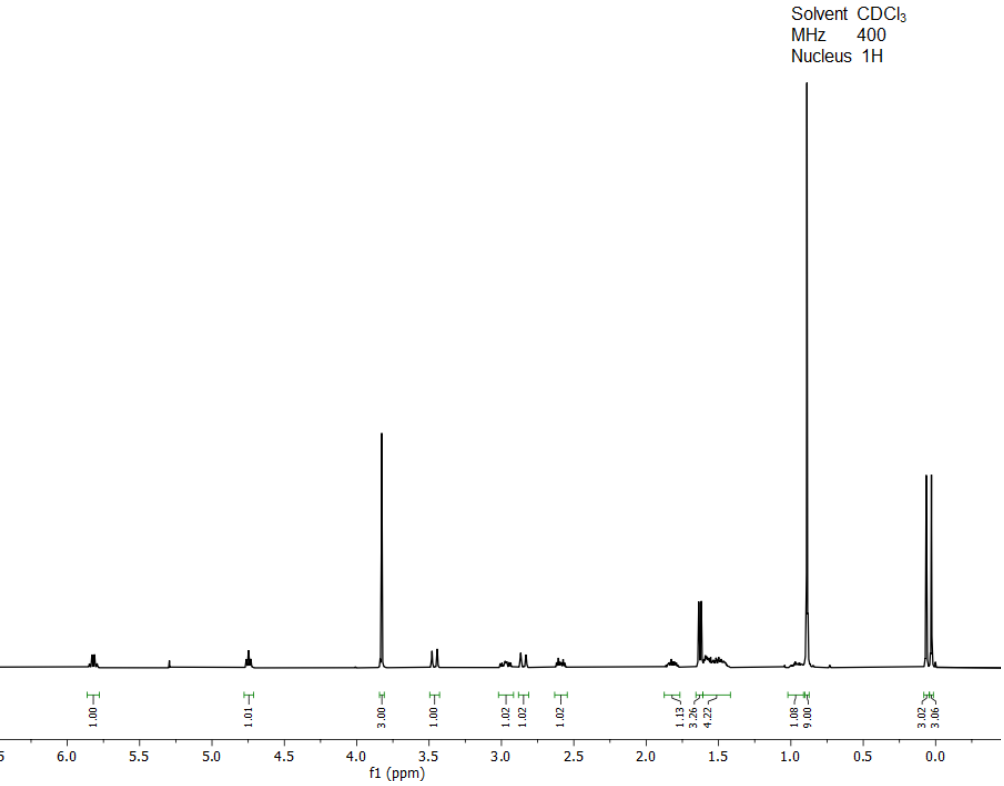
Solvent CDCl3
MHz 400
Nucleus 1H
1.00
1.01
3.00
1.00
1.02
1.02
1.02
1.13
3.26
4.22
1.08
9.00
3.02
3.06
7.5 7.0 6.5 6.0 5.5 5.0 4.5 4.0 3.5 3.0 2.5 2.0 1.5 1.0 0.5 0.0
f1 (ppm)

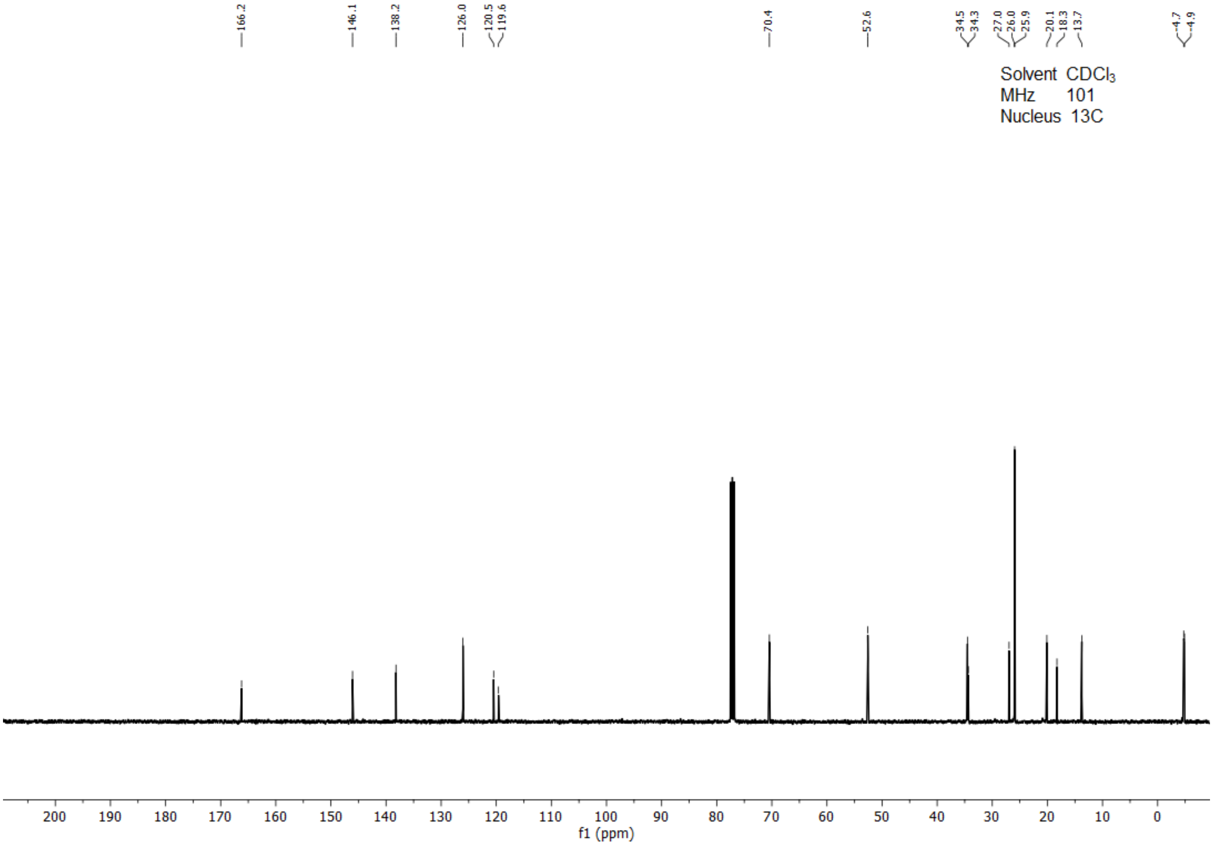
166.2
146.1
138.2
126.0
120.5
119.6
70.4
52.6
34.5
34.3
27.0
26.0
25.9
20.1
18.3
13.7
-4.7
-4.9
Solvent CDCl3
MHz 101
Nucleus 13C
200 190 180 170 160 150 140 130 120 110 100 90 80 70 60 50 40 30 20 10 0
f1 (ppm)

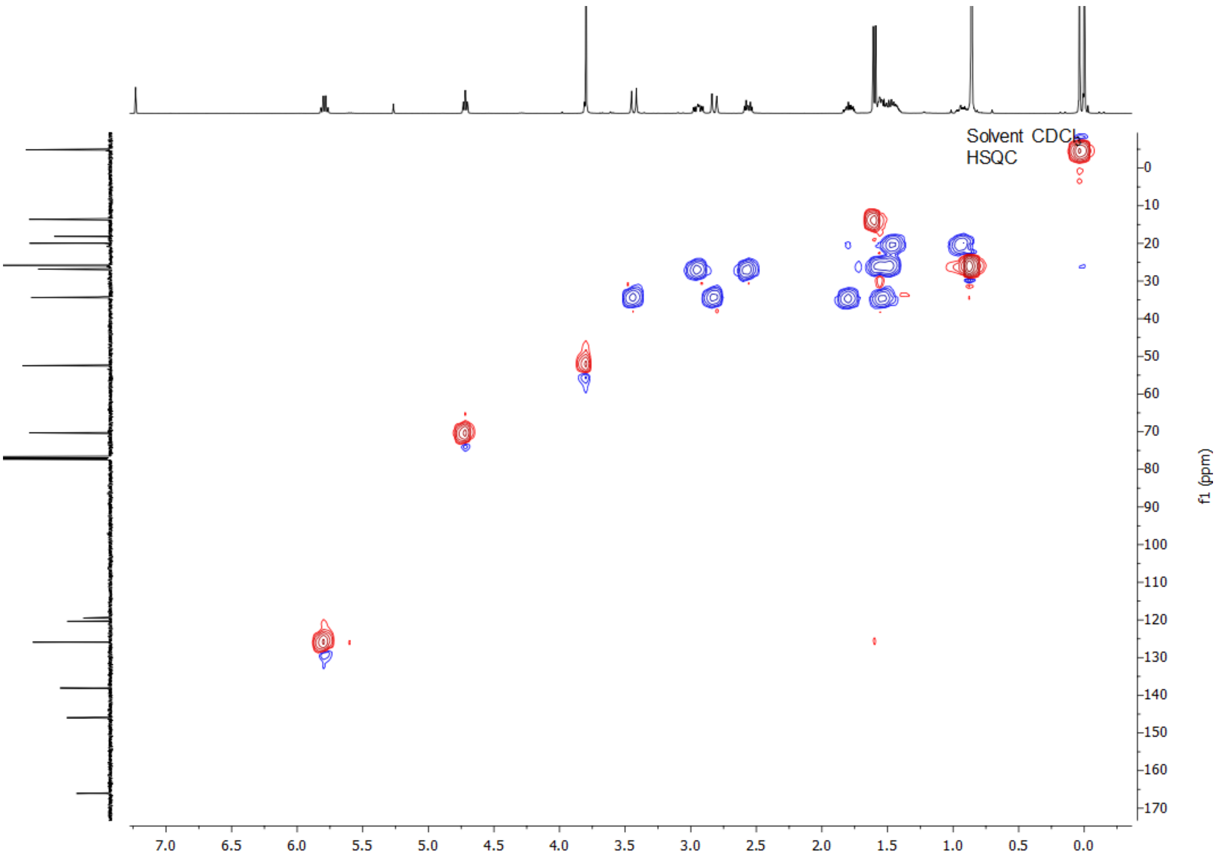
Solvent CDCl3
HSQC
f1 (ppm)

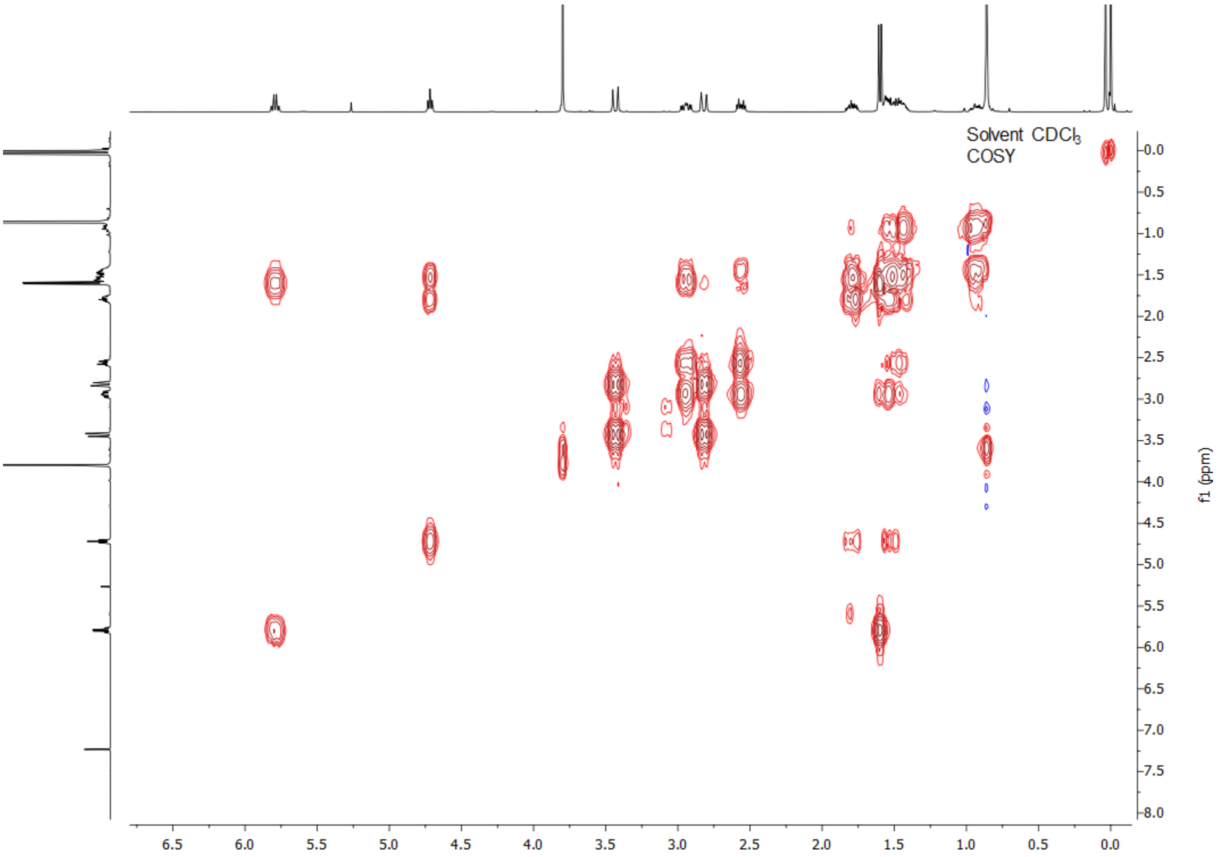
Solvent CDCl3
COSY
f1 (ppm)

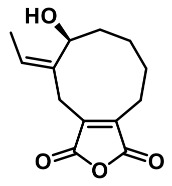
HO
O
O
O

10

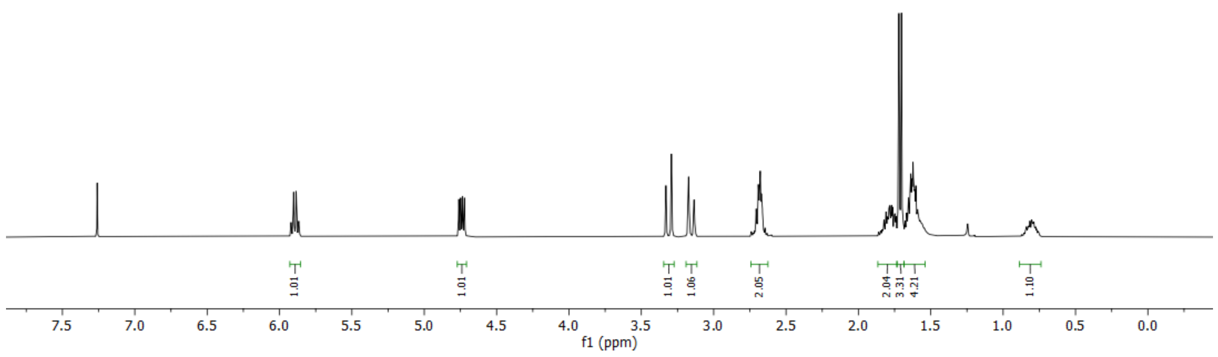
Solvent CDCl3
MHz 400
Nucleus 1H
1.01
1.01
1.01
1.06
2.05
2.04
3.31
4.21
1.10
7.5
7.0
6.5
6.0
5.5
5.0
4.5
4.0
3.5
3.0
2.5
2.0
1.5
1.0
0.5
0.0
f1 (ppm)

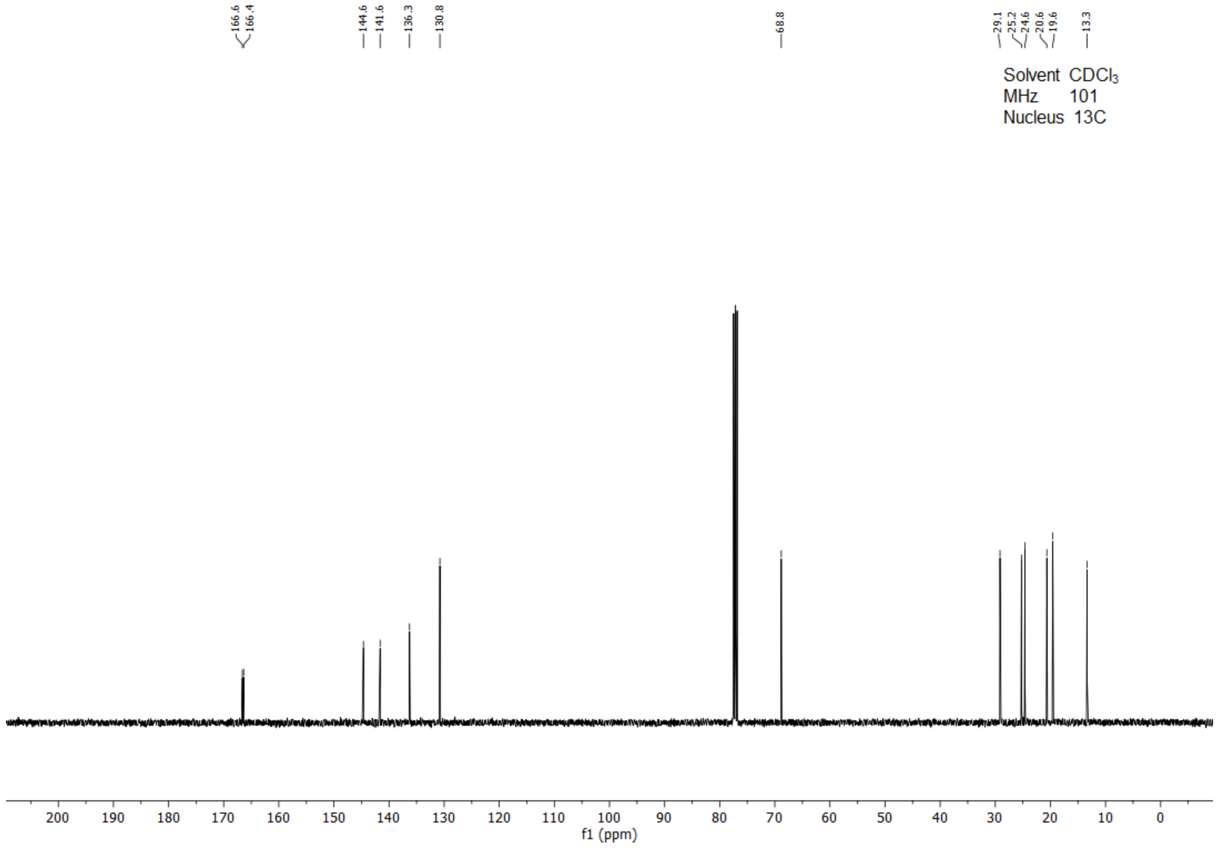
166.6
166.4
144.6
141.6
136.3
130.8
68.8
29.1
25.2
24.6
20.6
19.6
13.3
Solvent CDCl3
MHz 101
Nucleus 13C
200
190
180
170
160
150
140
130
120
110
100
90
80
70
60
50
40
30
20
10
0
f1 (ppm)

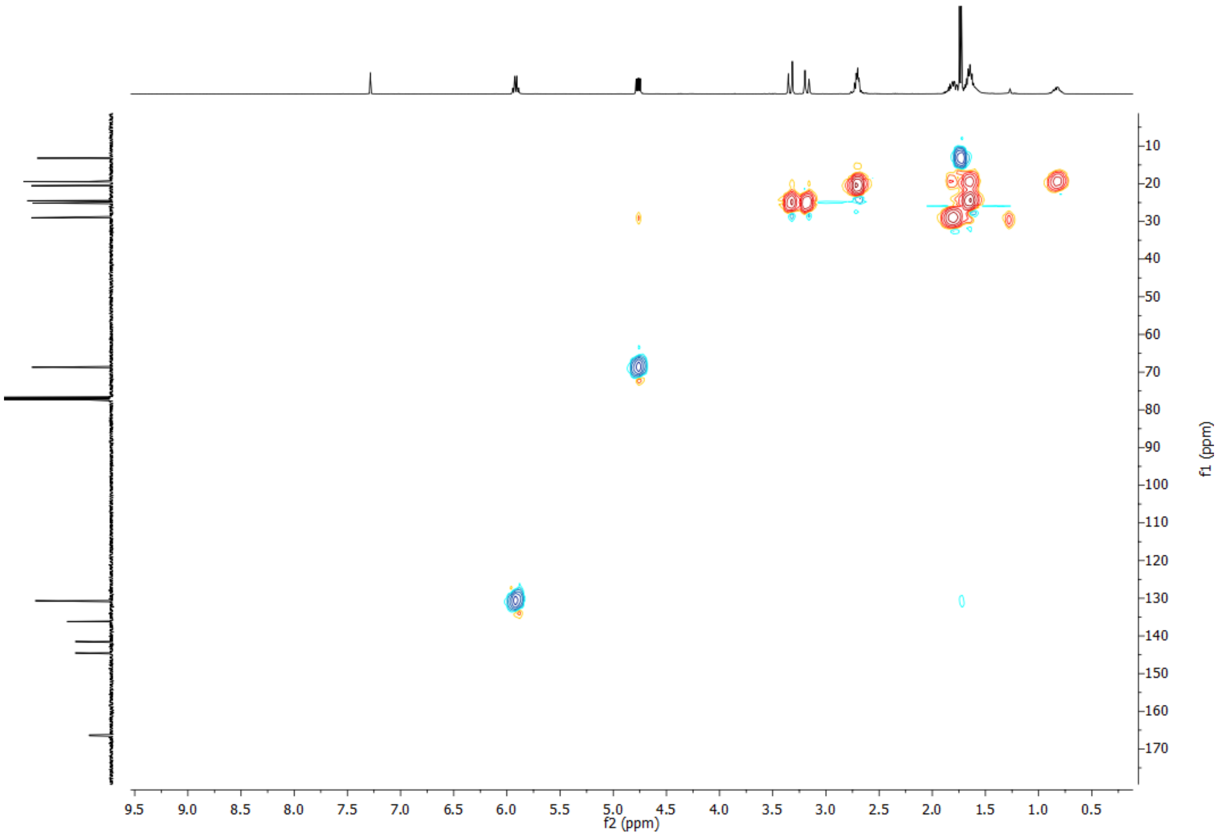
f2 (ppm)
f1 (ppm)

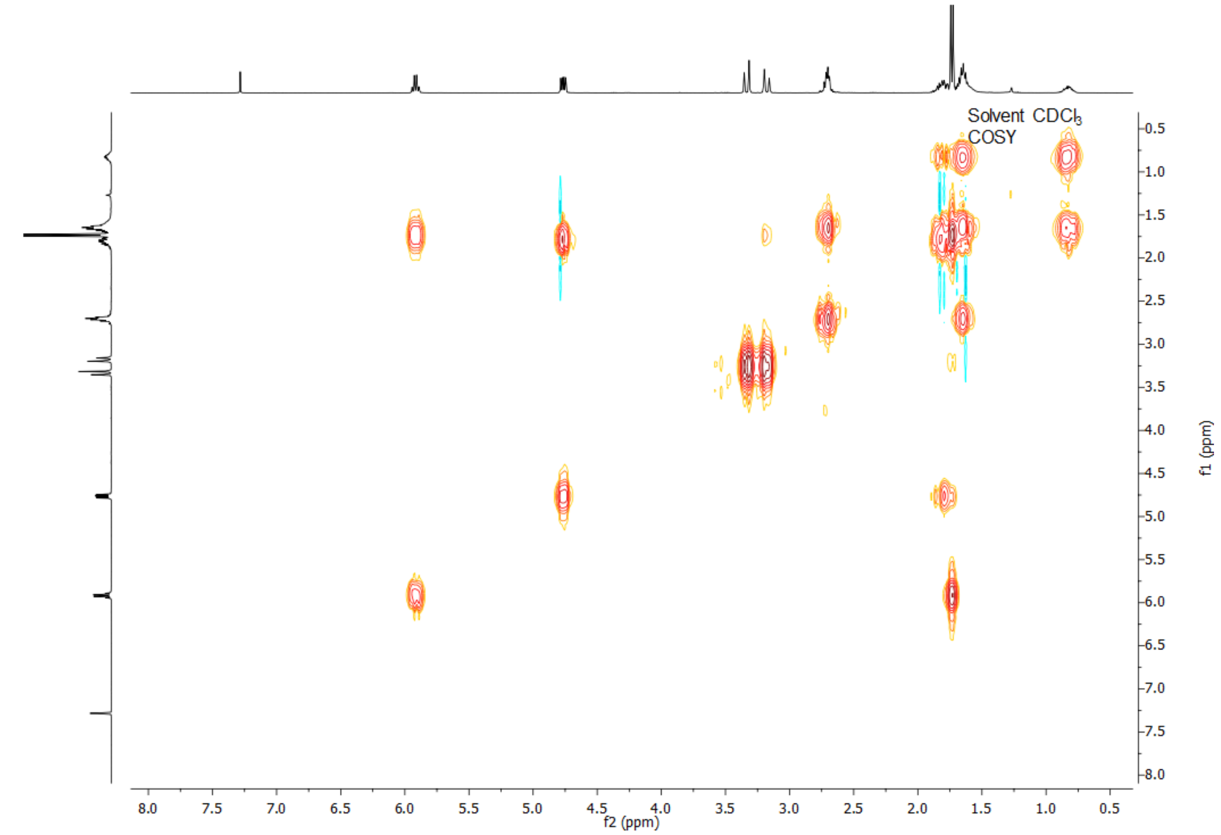
Solvent CDCl3
COSY
f2 (ppm)
f1 (ppm)
